# Analysis of the biodegradative and adaptive potential of the novel polychlorinated biphenyl degrader *Rhodococcus* sp. WAY2 revealed by its complete genome sequence

**DOI:** 10.1099/mgen.0.000363

**Published:** 2020-04-02

**Authors:** Daniel Garrido-Sanz, Paula Sansegundo-Lobato, Miguel Redondo-Nieto, Jachym Suman, Tomas Cajthaml, Esther Blanco-Romero, Marta Martin, Ondrej Uhlik, Rafael Rivilla

**Affiliations:** ^1^​ Departamento de Biología, Facultad de Ciencias, Universidad Autónoma de Madrid, C/ Darwin 2, 28049 Madrid, Spain; ^2^​ Department of Biochemistry and Microbiology, Faculty of Food and Biochemical Technology, University of Chemistry and Technology Prague, Technika 3, 16628 Prague, Czech Republic; ^3^​ Laboratory of Environmental Biotechnology, Institute of Microbiology, Czech Academy of Sciences v.v.i., Vídeňská 1083, 14200 Prague, Czech Republic

**Keywords:** *Rhodococcus*, biodegradation, PAH, PCB, hydrocarbons, complete genome

## Abstract

The complete genome sequence of *Rhodococcus* sp. WAY2 (WAY2) consists of a circular chromosome, three linear replicons and a small circular plasmid. The linear replicons contain typical actinobacterial invertron-type telomeres with the central CGTXCGC motif. Comparative phylogenetic analysis of the 16S rRNA gene along with phylogenomic analysis based on the genome-to-genome blast distance phylogeny (GBDP) algorithm and digital DNA–DNA hybridization (dDDH) with other *Rhodococcus* type strains resulted in a clear differentiation of WAY2, which is likely a new species. The genome of WAY2 contains five distinct clusters of *bph*, *etb* and *nah* genes, putatively involved in the degradation of several aromatic compounds. These clusters are distributed throughout the linear plasmids. The high sequence homology of the ring-hydroxylating subunits of these systems with other known enzymes has allowed us to model the range of aromatic substrates they could degrade. Further functional characterization revealed that WAY2 was able to grow with biphenyl, naphthalene and xylene as sole carbon and energy sources, and could oxidize multiple aromatic compounds, including ethylbenzene, phenanthrene, dibenzofuran and toluene. In addition, WAY2 was able to co-metabolize 23 polychlorinated biphenyl congeners, consistent with the five different ring-hydroxylating systems encoded by its genome. WAY2 could also use *n*-alkanes of various chain-lengths as a sole carbon source, probably due to the presence of *alkB* and *ladA* gene copies, which are only found in its chromosome. These results show that WAY2 has a potential to be used for the biodegradation of multiple organic compounds.

## Data Summary

The genome assembly of *Rhodococcus* sp. WAY2 has been submitted to the National Center for Biotechnology Information (NCBI), BioProject ID PRJNA580179, and it is accessible in the GenBank database under the accession numbers CP046572–CP046576.

**Impact Statement**

Members of the genus *Rhodococcus* are known for their ability to degrade many organic compounds. Their genomes provide a deep understanding of their catabolic abilities and environmental adaption strategies, which are essential for any biotechnological application. In this study, we report the complete multipartite genome sequence of the novel polychlorinated biphenyl (PCB) degrader *Rhodococcus* sp. strain WAY2. Comparative genomic analyses show that it is a potentially novel species. Multiple complementary catabolic pathways that are contained in its mega-plasmids allow its growth on multiple organic compounds, including aliphatic and aromatic hydrocarbons, and the co-metabolism of recalcitrant PCB congeners. The presence of several environmental adaption features could allow its survival during different nutritional states, and its ability to grow at a broad range of temperatures and in the presence of toxic compounds. This study provides a thorough characterization of the genomic features of *Rhodococcus* sp. WAY2 that shows its potential for application in bioremediation technologies.

## Introduction

The genus *Rhodococcus* comprises Gram-positive actinobacteria that are widely distributed in the environment, including in soil and water from tropical, desert and artic habitats [1–3]. Although some members of this genus are known pathogens of plants and animals [4, 5] or symbionts in the gut of insects [6], most of the species have been isolated from different polluted sites where they thrive along with other bacterial populations [7–9]. In fact, the ability to grow in polluted environments, including hydrocarbon-polluted soils [10], results from the presence of multiple catabolic pathways predominantly encoded by extrachromosomal replicons [11, 12], which allow rhodococci to degrade a wide range of chemical structures, including aliphatic hydrocarbons, polycyclic aromatic hydrocarbons (PAHs) and halogenated compounds such as polychlorinated biphenyls (PCBs), nitroaromatics, heterocyclic compounds and herbicides [8, 10, 13, 14]. The adaptation of rhodococci to various environments and nutritional states also relies on the use of storage compounds, such as glycogen, polyhydroxyalkanoates (PHAs) and triacylglycerols (TAGs), and the presence of cold shock proteins, osmotic and oxidative stress proteins and other factors involved in membrane/cell wall alteration [3, 15–18].

Genome analyses of *Rhodococcus* strains have shown their huge metabolic potential [12, 19]. It has been proposed that the catabolic abilities of rhodococci rely on a hyper-recombinant strategy that consists of the acquisition and storage of genes to be deployed for recombination, contributing to the dispersal of acquired DNA without the involvement of mobile genetic elements [20]. This results in large and complex multipartite genomes [21] consisting of a main chromosome, which can be circular or linear, and multiple linear replicons up to 1.1 Mbp in size, [12] together with small circular plasmids. Actinobacterial linear replicons usually show invertron-type telomeres, which are proposed to have evolved from bacteriophages [22]. The presence of these multiple extrachromosomal elements contributes to the overall rich repertoire of catabolic genes that appear in *Rhodococcus* genomes [19]. Among them, dioxygenase systems have been extensively studied in this genus because of their involvement in the degradation of several toxic aromatic compounds, including the *bph*, *etb* and *nah* gene clusters encoding, among others, the ring-hydroxylating dioxygenases that initiate the degradation of biphenyl and PCB congeners, ethylbenzene and naphthalene, respectively, in many strains [11, 14, 23–25]. Multiple studies have shown that these enzymes are versatile and the range of aromatic substrates they can utilize is wide, including biphenyl/PCBs, naphthalene, ethylbenzene, dibenzofuran, dibenzo-*p*-dioxins, phenanthrene, toluene and xylene, among others [24, 26–28].

The catabolic potential that *Rhodococcus* strains exhibit, along with extensive ecological adaptions to harsh and changing conditions, make this genus specifically suited for multiple environmental and industrial applications. Rhodococci have been successfully applied in the bioremediation of fuel-oil [2, 9], PAHs [29, 30] and herbicides [31], among other pollutants. Members of this genus have also been used as biocatalysts [32], including in the production of biofuel, degradation of pharma pollutants and production of acrylic acid [33–35]. Furthermore, *Rhodococcus* strains recently have been gaining attention in the discovery and production of drugs [36, 37], and in the production of electricity from organic waste in microbial fuel cells [38]. All these examples of biotechnological uses make the discovery and characterization of novel *Rhodococcus* strains essential for new or enhanced applications.

*Rhodococcus* sp. WAY2 was isolated from a bacterial rhizosphere consortium growing with biphenyl as the sole carbon and energy source in a previous metagenomic study [39]. In this work, we report the complete genome sequence of *Rhodococcus* sp. WAY2. Phylogenetic and phylogenomic comparisons with other *Rhodococcus* type strains were conducted to address its taxonomic status. In addition, further genome and functional analyses were performed to characterize its degradative potential.

## Methods

### Growth conditions and functional characterization

*Rhodococcus* sp. WAY2 was routinely grown in liquid LB or solid PCA (1.5 %, w/v, agar) media. The range of temperatures in which WAY2 was able to grow was tested on PCA plates at 5, 12, 20, 28, 37 and 40 °C. The ability of WAY2 to grow in LB liquid media supplemented with 1, 2, 3, 4 and 5 % NaCl or 1–5 % of toluene was determined by turbidity. Minimal salt medium (MM) [40] supplemented with 0.1 % (v/v) of phosphate-buffered mineral medium salts (PAS) [41] was used for growing WAY2 with different organic compounds as the sole carbon and energy sources at 1 g l^−1^ for biphenyl, naphthalene, *n*-pentadecane, *n*-heptadecane and *n*-tetracosane, and at 1 ml l^−1^ for xylene (*p*-, *m*- and *o*-xylene mixture) and toluene. Phenanthrene, dibenzofuran, ethylbenzene, *n*-hexane, 1-butanol, 2-butanol and methanol were added to the lid of solid MM+PAS media (1.5 %, w/v, agar) either as crystals or in sterile 200 µl microtubes for constant vapour release.

### Genome sequencing and assembly

Total DNA extraction for genome sequencing was performed using LB-grown WAY2 culture with the NucleoSpin microbial DNA kit (Macherey-Nagel), according to the manufacturer's specifications. The genome of *Rhodococcus* sp. WAY2 was sequenced by paired-end Illumina MiSeq 2×150 and PacBio RS II (Pacific Biosciences) technologies by the Parque Científico de Madrid (Spain) and Novogene Co., Ltd. (China), respectively. Illumina reads were quality-filtered with Trimmomatic software v0.38 [42]. Both Illumina and PacBio RS II reads were used for hybrid assembly with SPAdes software v1.12.0 [43] using read error correction and careful modes to correct and reduce the number of mismatches and short indels, respectively. PacBio RS II raw reads were separately quality filtered and assembled into contigs with canu software v1.7.1 [44] using a corrected ErrorRate parameter of 0.105 and an estimated genome size of 8.4 Mbp, with remaining parameters as the default values. The two assemblies were compared using blastn v2.2.31+ [45] to find overlapping contigs, which allowed genome closure and plasmid determination. Additionally, the topology of the pRWAY01, pRWAY02, pRWAY03 and pRWAY04 replicons was confirmed by PCR, the full description is in File S1 (available with the online version of this article). The overall assembly sequence coverage was calculated with the CollectWgsMetricsWithNonZeroCoverage tool within gatk software v1.0.11.0 [46] and using the mapped Illumina and PacBio reads against the final assembly using bowtie2 v2.3.4.2 [47] with processing by samtools v1.7 [48] to retain only the mapped reads.

### Genome annotation

The complete genome of *Rhodococcus* sp. WAY2 was annotated using the rast pipeline [49] and eggNOG-mapper v1.0.3 [50] against the bacNOG version 38 database. Annotations of regions of interest were additionally curated by blastn or blastp against the nucleotide (nt) or nonredundant (nr) National Center for Biotechnology Information (NCBI) databases, respectively. Noncoding RNA (ncRNA) genes were predicted using RNAspace online environment v1.2.1 [51], using blast searches of the Rfam 10.0 database and default parameters. Annotations were used to generate genome maps using DNAPlotter software [52].

The telomeric sequences of WAY2 linear replicons and those found in closely related genomes were identified by blastn against the NCBI nt database and aligned using multiple sequence comparison by log-expectation (muscle) [53]. The alignments were manually examined to identify the previously described CGTXCGC motif [54, 55] and inverted terminal repeats. Genomic islands (GIs) were predicted with the IslandViewer 4 online service [56], using the IslandPath-DIMOB [57] and SIGI-HMM [58] prediction methods. Small GIs contained in a bigger GI were removed. Partially overlapping GIs were combined.

### Phylogenetic analysis

The 16S rRNA sequences of all *Rhodococcus* type strains (according to the List of Prokaryotic Names with Standing in Nomenclature – http://www.bacterio.net, last accessed in April 2019), listed in Table S1, were downloaded and aligned with Clustal Omega [59]. *Rhodococcus obuensis* ATCC 44610^T^ was excluded from further analyses because its 16S rRNA sequence was truncated (505 nt). The resulting alignment was trimmed from position 108 to 1331, using a 16S rRNA sequence of WAY2 chromosome (position 732 059 to 733 282) to adjust the alignments to 1249 nt. The resulting alignment was filtered with Gblocks v0.91b [60] to remove poorly aligned positions and divergent regions, using a minimum block length of two nucleotides and allowing gap positions in all sequences. A maximum-likelihood (ML) phylogenetic tree was rebuilt using the Pthreads-parallelized RAxML package v8.2.10 [61], using the GTR (General Time Reversible) model of nucleotide substitution [62] combined with the gamma model of rate heterogeneity and optimization of substitution rates using the bfgs algorithm. Rapid bootstrapping and subsequent ML search combined with the autoMRE [63] criterion were applied. The phylogenetic tree was plotted and exported using mega v7 software [64].

### Phylogenomic analysis

Only sequenced *Rhodococcus* type strains were used for phylogenomic analysis. Type strain *Rhodococcus* genomes were downloaded from the NCBI ftp RefSeq database in March 2019, resulting in the 38 genomes listed in Table S2. *Nocardia brasiliensis* NCTC 11294^T^ was used as an outgroup. Genome-to-genome blast distance phylogeny (GBDP) [65] was used via the genome-to-genome distance calculator (ggdc) v2.1 (http://ggdc.dsmz.de/) web service. Resulting sets of intergenomic distances were used to rebuild the neighbour-joining phylogenomic tree with mega v7 software. Digital DNA–DNA hybridization (dDDH) values were calculated using the recommended settings of the ggdc 2.1 [65]. Additionally, the genome sequence of WAY2 was submitted to the Type (Strain) Genome Server (TYGS; https://tygs.dsmz.de/) platform for a whole genome-based taxonomic analysis [66], using default parameters, as described elsewhere [67].

### Ring-hydroxylating dioxygenase phylogeny

Amino acid sequences of the five alpha-subunits of ring-hydroxylating dioxygenases identified in the WAY2 genome were aligned using Clustal Omega and an ML phylogenetic tree was rebuilt using the Pthreads-parallelized RAxML package as specified above, except that the LG model of amino acid evolution [68] combined with gamma-distributed substitution rates and empirical amino acid frequencies were used instead. Rapid bootstrapping and subsequent search of the best-scoring ML tree combined with the autoMRE criterion were applied.

### Resting cell assays with PCBs

Resting cell assays to test the ability of *Rhodococcus* sp. WAY2 to degrade PCB congeners were assessed. *Rhodococcus jostii* RHA1 [69], *Paraburkholderia xenovorans* LB400 [70, 71] and *Pseudomonas alcaliphila* JAB1 [72] were also included for comparison purposes. Bacterial cultures at an OD_600_ of 0.5–1 in MM+PAS medium growing with biphenyl as a sole carbon source were filtered with a funnel filled with sterilized glass wool to remove biphenyl crystals, centrifuged at 4000 ***g*** for 10 min and washed twice with MM+PAS. Cells were resuspended in 200 ml MM+PAS to give a final OD_600_ of 1. Autoclaved cell suspensions (120 °C for 20 min) were used as controls. Three replicas of 20 ml active and autoclaved cells were incubated with 0.001 % (w/v) Delor 103 PCB mixture in 120 ml glass vials sealed with screw caps for48 h at 28 °C on a rotary shaker at 250 r.p.m. The samples were stored at −20 °C until analysed. Individual PCB congener depletion in the microcosms were determined using GC-MS (450-GC, 240 MS ion trap detector; Varian), as previously described [72, 73].

## Results and Discussion

### Genome anatomy

**Fig. 1.**
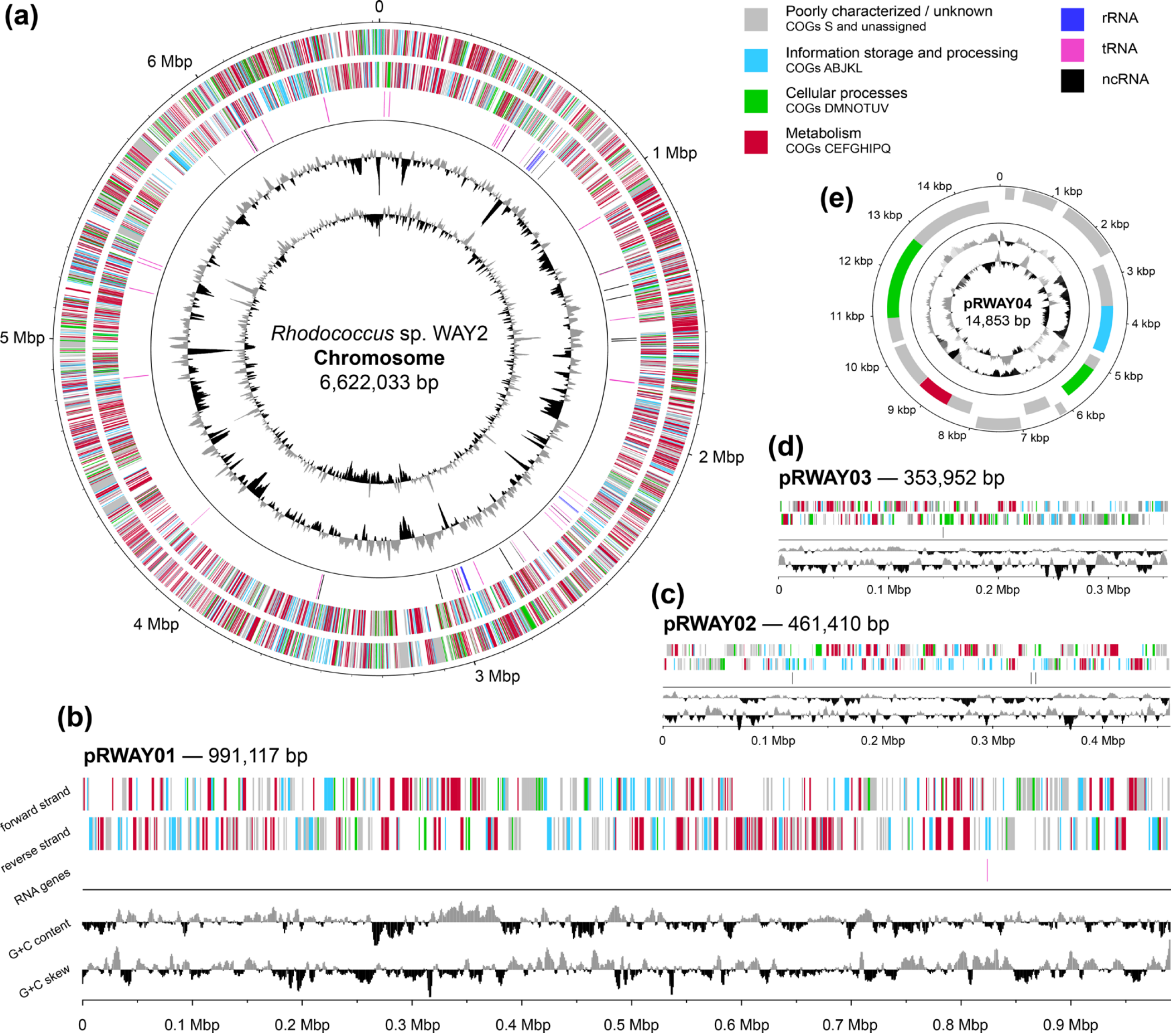
Genomic map of *Rhodococcus* sp. WAY2 replicons. Chromosome (a), linear mega-plasmids pRWAY01, pRWAY02 and pRWAY03 (b–d), and circular plasmid pRWAY04 (e). The outer/top two rings/rows represent coding sequences in the forward strands (first ring/row) and reverse strands (second ring/row), coloured according to the main COG types. The third circle/row represents RNA genes (dark blue, rRNA; purple, tRNA; black, ncRNA). The fourth circle/row represents deviation in G+C content, using a 10 000 bp window size and a 200 bp step size for the chromosome, a 3000 bp window size and 300 bp step size for the linear replicons, and a 200 bp window size and 5 bp step size for the pRWAY04 circular plasmid (grey/black represents above/below the mean G+C content, respectively). The fifth circle/row represents the G+C skew using a 10 000 bp window size and 500 bp step size for the chromosome, a 3000 bp window size and 200 bp step size for the three linear replicons, and a 200 bp window size and 5 bp step size for the pRWAY04 circular plasmid (grey/black represents above/below 1, respectively).

The multipartite genome architecture of *Rhodococcus* sp. WAY2 is composed of 8.44 Mbp distributed in five replicons: a circular chromosome of 6.62 Mbp, three linear mega-plasmids designated pRWAY01 (0.99 Mbp), pRWAY02 (0.46 Mbp) and pRWAY03 (0.35 Mbp), and a small circular plasmid designated pRWAY04 (14.85 kbp) (represented in Fig. 1). The final assembly yielded an overall mean coverage of 232×. The chromosome G+C content is 65.87 mol%, similar to that of the linear plasmids (65.04, 64.92 and 65.10 mol% respectively), while the small circular plasmid has a G+C content of 61.45 mol % (Table 1). The linearity of the pRWAY01, pRWAY02 and pRWAY03 mega-plasmids and the presence of the small circular pRWAY04 plasmid were also confirmed by PCR analyses (File S1).

**Table 1.** Summary of *Rhodococcus* sp. WAY2 genome characteristics across replicons

Characteristic	Replicon				
**Chromosome**	**pRWAY01**	**pRWAY02**	**pRWAY03**	**pRWAY04**
Total length (bp)	6 622 033	991 117	461 378	353 952	14 853
Topology	Circular	Linear	Linear	Linear	Circular
G+C mol%	65.86	65.04	64.92	65.10	61.45
Genes	6102	1066	497	393	20
Pseudogenes	102	16	29	11	0
Protein-encoding genes	6025	1065	494	392	20
rRNA genes	12	0	0	0	0
tRNA genes	51	1	0	0	0
ncRNA genes	14	0	3	1	0

The telomeres of the linear replicons of WAY2 are typical actinobacterial invertrons, with terminal inverted repeats (TIRs) and the central GCTXCGC motif, as described elsewhere [54, 55]. These telomeres are similar to those found in other linear replicons in rhodococci, such as *R. jostii* RHA1 and *Rhodococcus opacus* strains B4 and 1CP (File S2). WAY2 telomeric sequences share 88–93 % sequence identity along their first 500 bp, except for the pRWAY03 left end, which shares no homology with any other WAY2 telomeric sequence but has a 95 % sequence identity with the sequences of pRHL2 and pROB01 right ends from *R. jostii* RHA1 and *R. opacus* B4, respectively. Interestingly, the TIRs of the pRWAY03 left end contain two central GCTXCGC motifs with different short inverted repeats than those found in the remaining WAY2 telomeres (File. S2), which might suggest a different pRWAY03 origin compared to the other linear replicons. The three WAY2 linear mega-plasmids harbour distant homologous sequences of *tap* and *tpg* genes found in *Streptomyces* and other *Actinobacteria* [74–76], which encode putative telomerase-binding proteins (Tap) and terminal proteins (TPs), respectively (Table S3). Tap and TP encoded by the pRWAY03 right end share a 99 % amino acid sequence identity with other *Rhodococcus* Tap/TP proteins and between 33 and 37% sequence identity with *Arthrobacter* Tap/TP proteins characterized in its pAL1 linear plasmid [76]. Such poor Tap/TP sequence conservation has been previously reported within *Streptomyces* linear plasmids [75].

The WAY2 chromosome contains the *dnaA* chromosomal replication initiator gene, whereas several putative copies of the chromosome/plasmid partitioning and segregation *parAB* system are present in all WAY2 replicons (Table S3). Four *parA* homologues are present in the WAY2 chromosome and a single *parA* copy is found in each of the WAY2 plasmids. In contrast, *parB* copies are only found in the chromosome and in the pRWAY01 mega-plasmid, suggesting a dependency on the remaining replicons for stable maintenance during cell division. A similar *parAB* gene distribution is found in *R. jostii* RHA1 [12]. Amino acid sequence identity among ParAs of WAY2 is less than 50 %, but highly similar to those found in other rhodococcal genomes (>90 %). This finding suggests that the WAY2 chromosome and plasmids have different origins. The sequence dissimilarity between chromosomal ParAs is probably due to genetic exchange among extrachromosomal elements rather than duplication and speciation of *parA* genes, as previously suggested [77]. Nonetheless, these are probably recurring processes in rhodococci [12, 78, 79].

### Phylogenetic and phylogenomic analyses

**Fig. 2.**
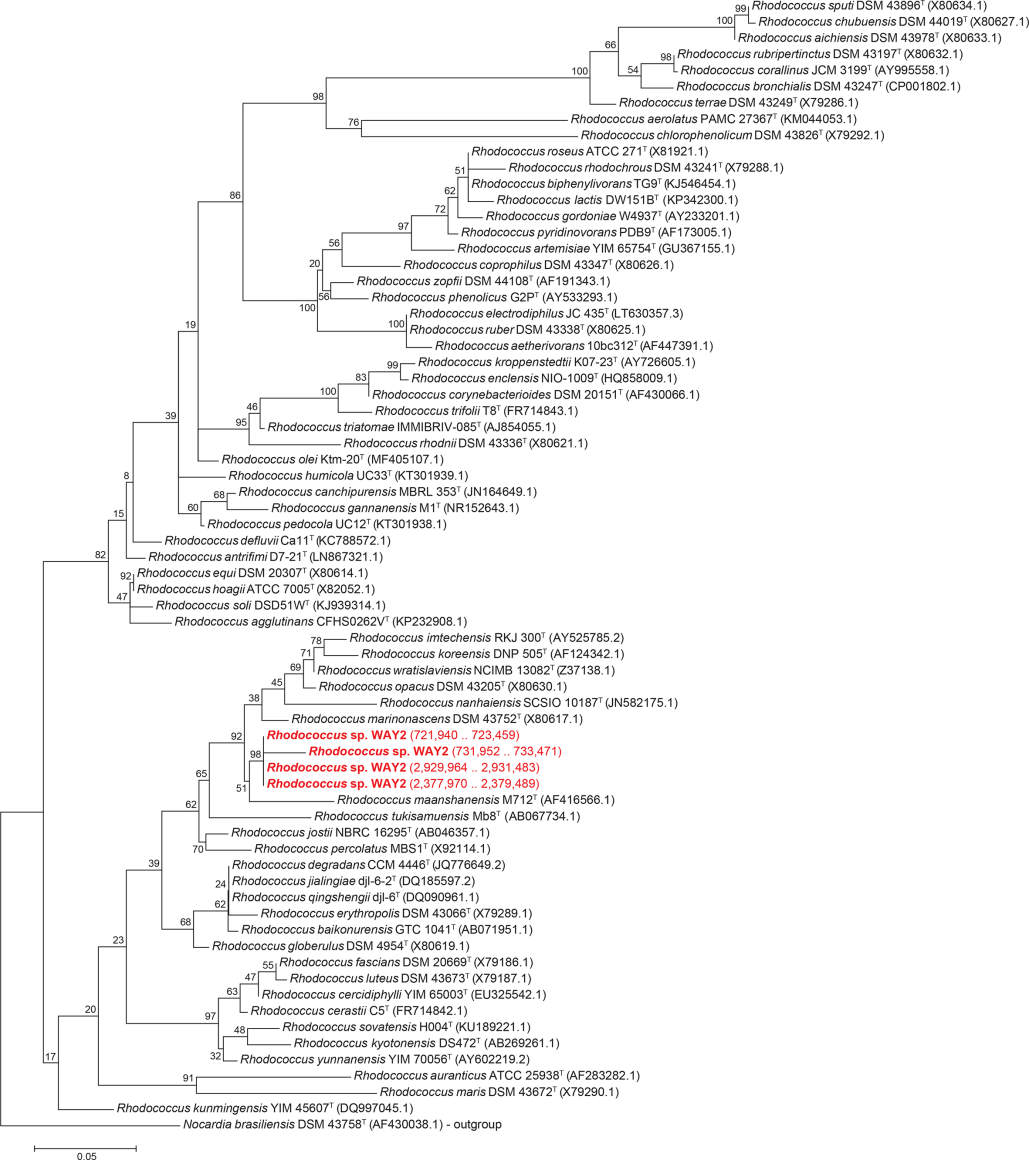
16S rRNA-based ML phylogeny of *Rhodococcus* type strains. Bootstrap values are specified in nodes. The four copies of *Rhodococcus* sp. WAY2 16S rRNA genes are highlighted in red type and coordinates are specified in parentheses. *N. brasiliensis* DSM 43758^T^ was used as an outgroup. Scale bar represents the number of substitutions per site.

**Fig. 3.**
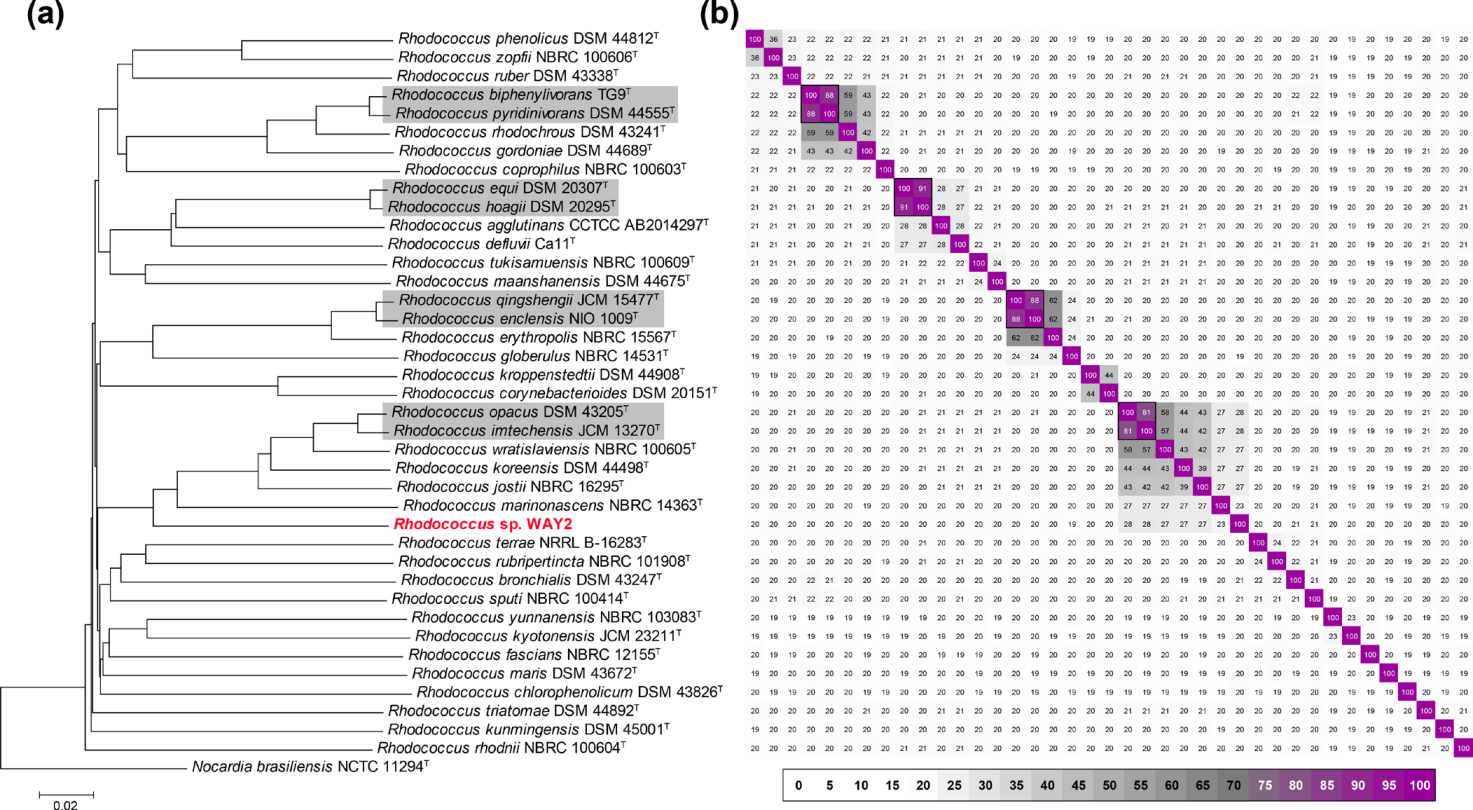
(a) Neighbour-joining phylogeny based on the GBDP intergenomic distances of the sequenced genomes of 38 *Rhodococcus* type strains and WAY2 (red type). Grey boxes denote those comparisons resulting in >70% dDDH. Scale bar represents evolutionary distance in the same units as those of the intergenomic distances used to infer the phylogenomic tree. (b) dDDH matrix of the genome comparisons.

As of October 2019, there were 66 validly named *Rhodococcus* species, of which 38 type strain genomes had been sequenced. Phylogenetic analyses based on the 16S rRNA gene sequences of all type strains and phylogenomic analyses based on intergenomic distances calculated with sequenced type strains and using the GBDP algorithm were carried out in order to evaluate the phyletic relationship of WAY2 within the genus *Rhodococcus *. The results show a clear distinction of WAY2 from the rest of the type strains (Figs 2 and 3). The four 16S rRNA genes of WAY2 form a single cluster separated from other type strains, with *Rhodococcus maanshanensis* M712^T^ being the closest relative. Other closely related 16S rRNA genes belong to known aromatic-degrading species, such as *R. jostii* and *R. opacus* (Fig. 2).

Regarding phylogenomic analyses, GBDP-based genome comparisons also show that WAY2 does not belong to any previously sequenced *Rhodococcus* species, since the dDDH value threshold for species delineation (≥70 %) is not achieved in any of the intergenomic comparisons (Fig. 3). The highest dDDH values were obtained in the comparison of WAY2 with *R. opacus* DSM 43205^T^ and *Rhodococcus imtechensis* JCM 13270^T^ (27.7 and 27.6% dDDH, respectively; Table S4), which are known to degrade different aromatic compounds [8, 80]. Surprisingly, four genome comparisons of validly named type strains resulted in dDDH values above the species threshold (Fig. 3), denoting that they are potentially the same species. These are *Rhodococcus biphenylivorans* TG9^T^ and *Rhodococcus pyridinivorans* DSM 44555^T^ (88.3 % dDDH), *Rhodococcus equi* DSM 20307^T^ and *Rhodococcus hoagii* DSM 20295^T^ (91 % dDDH), *Rhodococcus qingshengii* KCM 15477^T^ and *Rhodococcus enclensis* NIO 1009^T^ (88 % dDDH), and *R. opacus* DSM 43205^T^ and *R. imtechensis* JCM 13270^T^ (81.2 % dDDH; Fig. 3b). The taxonomic discrepancy concerning *R. equi* and *R. hoagii* is in agreement with a reclassification proposal reported elsewhere [81].

The phylogenetic and phylogenomic analyses show similar results and strongly support the status of *Rhodococcus* sp. WAY2 as a novel species, being clearly separated from any other named *Rhodococcus *. These results were additionally validated using the new TYGS [66] and are described in File S3.

### General functional content

The annotation of the *Rhodococcus* sp. WAY2 genome resulted in 8078 genes and 158 pseudogenes distributed across the replicons, with 7996 (99 %) genes being protein encoding, 2873 (35.6 %) of which putatively encode proteins of unknown function (Table 1). A total of 82 RNA genes were found in the WAY2 genome. The chromosome contains four rRNA operons, all ribosomal proteins (except for S21p) and 51 tRNA genes, except for a copy of the tRNA^Gly^_CCC_ located also in the pRWAY01 mega-plasmid (Fig. 1). This distribution is similar to that reported in other rhodococci [12]. In addition, 18 ncRNA genes have been identified, mainly in the chromosome (14), but also in the mega-plasmids pRWAY02 (3) and pRWAY03 (1), as shown in Table 1.

**Fig. 4.**
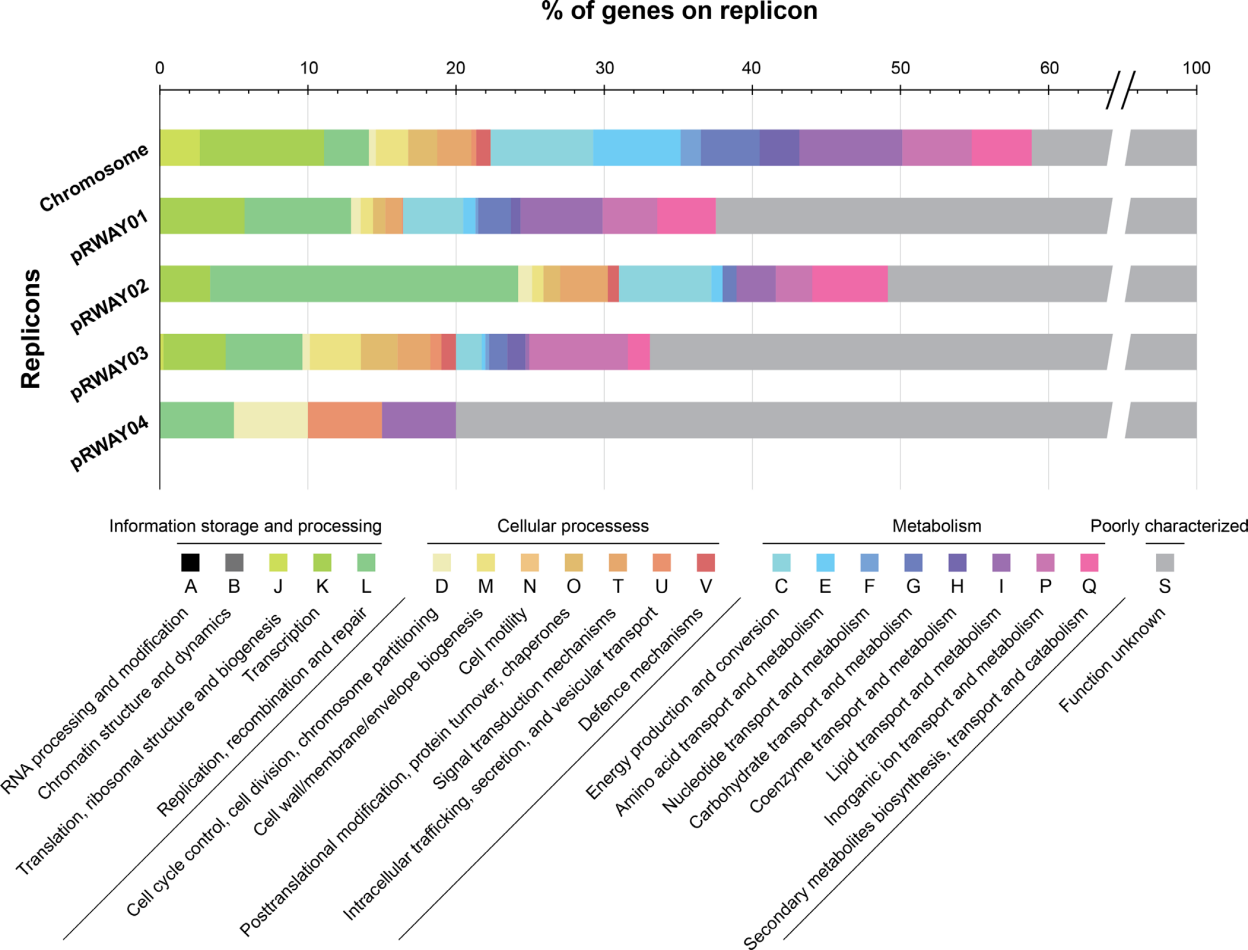
Functional distribution of COGs among the five *Rhodococcus* sp. WAY2 replicons. Proteins were classified into COGs using eggNOG-mapper. Proteins with no COG hit were included in the S category

The functional distribution of clusters of orthologous groups (COGs) across the WAY2 replicons was examined with eggNOG-mapper [50] and is summarized in Fig. 4. The chromosome of WAY2 contains most of genes associated with core cellular functions, such as translation, energy production and conversion; and amino acid, nucleotide and lipid transport and metabolism (COGs J, K, C, E, F, G). Conversely, all plasmids are enriched in replication, recombination and repair (COG L) genes compared to the WAY2 chromosome. This category represents 30.8 % of all COGs in the pRWAY02 mega-plasmid and around 10 % in the remaining ones. Yet, none of the genes found in the plasmids COG L category participate in core cellular functions. These genes are annotated as mobile elements, transposases, integrases, retron-type RNA-directed DNA polymerases and other integrative elements that suggest genetic transference events. Moreover, genes encoding the machinery for cell division, replication, transduction and translation are exclusively found in the WAY2 chromosome (Table S3). WAY2 mega-plasmids are rich in genes associated with metabolism, such as those included in the COGs categories C, I and P (energy production and conversion; lipid transport and metabolism; and inorganic ion transport and metabolism, respectively), representing between 4.1 and 6.7 %. However, categories related to cellular processes are scarce among the mega-plasmids, except for those of posttranscriptional modifications and signal transduction mechanisms (COGs O and T).

The distribution of COGs observed in the WAY2 replicons is consistent with a specialization of the mega-plasmids towards peripheral metabolism (see below). This functionality division between core functions encoded by the chromosome and secondary or novel adaptive functions harboured in the mega-plasmids is typical of multipartite genomes found in many different phyla [21, 82].

### Degradation capabilities of *Rhodococcus* sp. WAY2

The ability of WAY2 to use different carbon sources was addressed by growing WAY2 on several aromatic compounds, alkanes and alcohols as sole carbon and energy sources. The results are summarized in Table 2.

**Table 2.** Functional characterization of the aromatic degradation capabilities of *Rhodococcus* sp. WAY2

Characteristic	* Rhodococcus* sp. WAY2	Characteristic	* Rhodococcus* sp. WAY2
**Ranges of growth**		**Utilization/oxidation of:**	
Temperature (°C)	5–37	*n*-Alkanes	
Lowest pH	6	Hexane	−
Highest NaCl (%)	4	Pentadecane	+
**Utilization/oxidation of:**		Heptadecane	+
Aromatics		Tetracosane	+
Biphenyl	+	Alcohols
Naphthalene	+	1-butanol	+
Xylene	+	2-butanol	−
Ethylbenzene	o	Methanol	−
Phenanthrene	o	
Dibenzofuran	o	
Toluene*	o		

+, Growth; −, lack of growth; o, no growth observed but a change in the colour of the media was observed.

*Growth using toluene as sole carbon source was not detected, but resistance up to 1 % was observed.

Among the aromatic compounds tested, WAY2 can use biphenyl, naphthalene and xylene (*p*-, *m*- and *o*-xylene mixture) as sole carbon sources. Changes in media colour (to yellow-brown) were observed with ethylbenzene, phenanthrene, dibenzofuran and toluene, indicating partial transformation of these compounds into the respective products of extradiol dioxygenases [83–85]. WAY2 was also able to grow with various chain-length *n*-alkanes (pentadecane, heptadecane and tetracosane) as well as 1-butanol as sole carbon sources, but not with hexane, 2-butanol nor methanol. In addition, WAY2 was able to grow at between 5 and 37 °C, with the optimal growth temperature ranging between 20 and 37 °C, and to tolerate up to 4 % NaCl. This was not unexpected, as rhodococci are found in cold, tropical and even arid and desert environments [18, 86, 87], and thrive in marine environments [36, 88].

### Central metabolic pathways

The chromosome of *Rhodococcus* sp. WAY2 encodes genes involved in typical central carbohydrate metabolic pathways in bacteria, including the two glycolytic pathways Embden–Meyerhof–Parnas (EMP) and Entner–Duodoroff (ED) previously reported in other *Rhodococcus* [89], as well as gluconeogenesis, the pentose phosphate pathway and the tricarboxylic acid (TCA) cycle (Tables 3 and S5). The chromosome also encodes general pathways for purine and pyrimidine metabolism, the β-oxidation pathway, and metabolic pathways for amino-acids biosynthesis, degradation and their interconversion (Table 3).

**Table 3.** Central metabolic pathways identified in the *Rhodococcus* sp. WAY2 genome and the genes involved For additional information see Table S5.

Pathway	Replicon	Gene
GLY	Chromosome	*glkA*, *glk*, *gntk*, *ppgK*, *pgi1-2*, *pfkA*, *fruK*, *fba*, *tpiA*, *gap*, *pgk*, *gpmA1-2*, *eno*, *pyk*, *edd*, *edaK*, *zwf1-4*, *pgl*
	pRWAY01	*pgl*
GLN	Chromosome	*pgi1-2*, *fba*, *gap*, *pgk*, *gpmA1-2*, *eno*, *glpX*, *pckG*
	pRWAY01	*glpX*
	pRWAY02	*pckG*
PP	Chromosome	*pgi1-2*, *zwf-4*, *pgl*, *gnd1-4*, *rpe*, *rpiB*, *tal*, *tkt1-2*
	pRWAY01	*pgl*, *gnd*, *tal*
ACoA	Chromosome	*pdh*, *pdhA1-3*, *pdhB1-3*, *pdhC1-4*, *aceE1-2*
TCA	Chromosome	*citA*, *gltA*, *acnA*, *icd1-2*, *sucA*, *sucC*, *sucD*, *sdhA1-2*, *sdhB1-2*, *fumB*, *mqo*, *mdh1-4*
	pRWAY01	*citA*, *mdh1-2*
PU	Chromosome	*purF*, *purD*, *purN*, *purM*, *purL*, *purS*, *purQ*, *purK*, *purE*, *purC*, *purB*, *purH*, *purA*, *purB*, *adk1-2*, *ndk*, *gmk*, *guaA*, *guaB1-4*, *xdhA1-2*, *xdhB1-2*, *pucL*, *puuE*, *pucM*, *uraD*, *alc*
PY	Chromosome	*dnk*, *carA*, *carB*, *pyrC*, *pyrR*, *pyrD*, *pyrE*, *pyrF*, *pyrH*, *pyrG*, *nrdE*, *nrdF*, *nrdH*, *nrdI*, *dcd*, *dut*, *thyA*, *tmk1-2*
BO	Chromosome	*fadD1-17*, *acx*, *fadE1-26*, *acd*, *paaF1-3*, *echA1-15*, *hadH1-7*, *fadA1-15*, *atoB1-4*
	pRWAY01	*fadD*, *fadE*, *paaF*, *hadH*, *fadA1-2*
	pRWAY02	*fadA*, *atoB1-2*
AAM	Chromosome	*serA*, *serB1-2*, *serC*, *lysC*, *asd*, *hom*, *thrB*, *thrC*, *cysK1-2*, *cbs*, *cth*, *metK*, *pfs*, *metE*, *metH*, *achy*, *cdm*, *ilvB*, *ilvC*, *ilvD*, *ilvE1-3*, *leuB*, *leuC1-3*, *leuD1-3*, *leuA*, *lpd1-4*, *lpdA*, *dapA1-4*, *dapB*, *dapD*, *argD*, *dapC*, *dapE*, *dapF*, *lysA*, *argJ*, *argB*, *argC*, *argF*, *argG*, *argH*, *proB*, *proA*, *proC*, *hisG*, *hisE*, *hisI*, *hisA*, *hisF*, *hisB*, *hisC*, *hisD*, *hutH*, *hutU*, *hutI*, *hutG*, *trpC*, *trpD*, *trpE*, *trpF*, *trpG*, *trpB*, *trpA*, *csm*, *pheA*, *aspC*, *tyrA*, *tat1-2*, *hppD1-2*, *kynA*, *kynU*
BKA	Chromosome	*pcaG*, *pcaH*, *pcaB*, *pcaC*, *pcaD1-5, pcaI*, *pcaJ*, *catA*, *catB*, *catC*
CATM	pRWAY01	*catE*
	pRWAY02	*catE*
2HDP	Chromosome	*bphE1-3*, *bphF1-3*, *bphG1-3*
	pRWAY01	*bphE*, *bphF1-3*, *bphG1-2*
	pRWAY02	*bphE*, *bphF*, *bphG1*-2
BEN	Chromosome	*benA*, *benB*, *benC*, *benD*
GEN	Chromosome	*gdoA1-2*
	pRWAY01	*gdoA*
	pRWAY02	*gdoA*
HGEN	Chromosome	*hmgA*

GLY, Glycolysis; GLN, gluconeogenesis; PP, pentose phosphate; ACoA, acetyl-CoA synthesis; TCA, tricarboxylic acid cycle; PU, purine metabolism; PY, pyrimidine metabolism; BO, β-oxidation; AAM, amino acid metabolism; BKA, β-ketoadipate; CATM, catechol meta-cleavage; 2HDP, 2-hydroxypentadienoate metabolism; BEN, benzoate metabolism; GEN, gentisate metabolism; HGEN, homogentisate metabolism.

Regarding central aromatic metabolism, genes for β-ketoadipate (*pcaGHBCDIJ* and *catABC*), 2-hydroxypentadienoate (2HDP; *bphEFG*), benzoate (*benABCD*), gentisate (*gdoA*) and homogentisate (*hmgA*) metabolism are found predominantly in the WAY2 chromosome, although copies of genes involved in the 2HDP and gentisate metabolism are also found in the mega-plasmids pRWAY01 and pRWAY02. Two copies of the catechol 2,3-dioxygenase-encoding genes (*catE*) involved in catechol *meta*-cleavage are also found in these plasmids (Tables 3 and S5), probably related to specific catabolic pathways of aromatic compounds. Interestingly, genes involved in phenylacetate and homoprotocatechuate degradation previously identified in *R. opacus* PD300 and *R. jostii* RHA1 [90, 91] are missing in the genome of WAY2, which could suggest specialized metabolism of aromatic compounds.

### Peripheral metabolism

#### Degradation of aromatic compounds

**Fig. 5.**
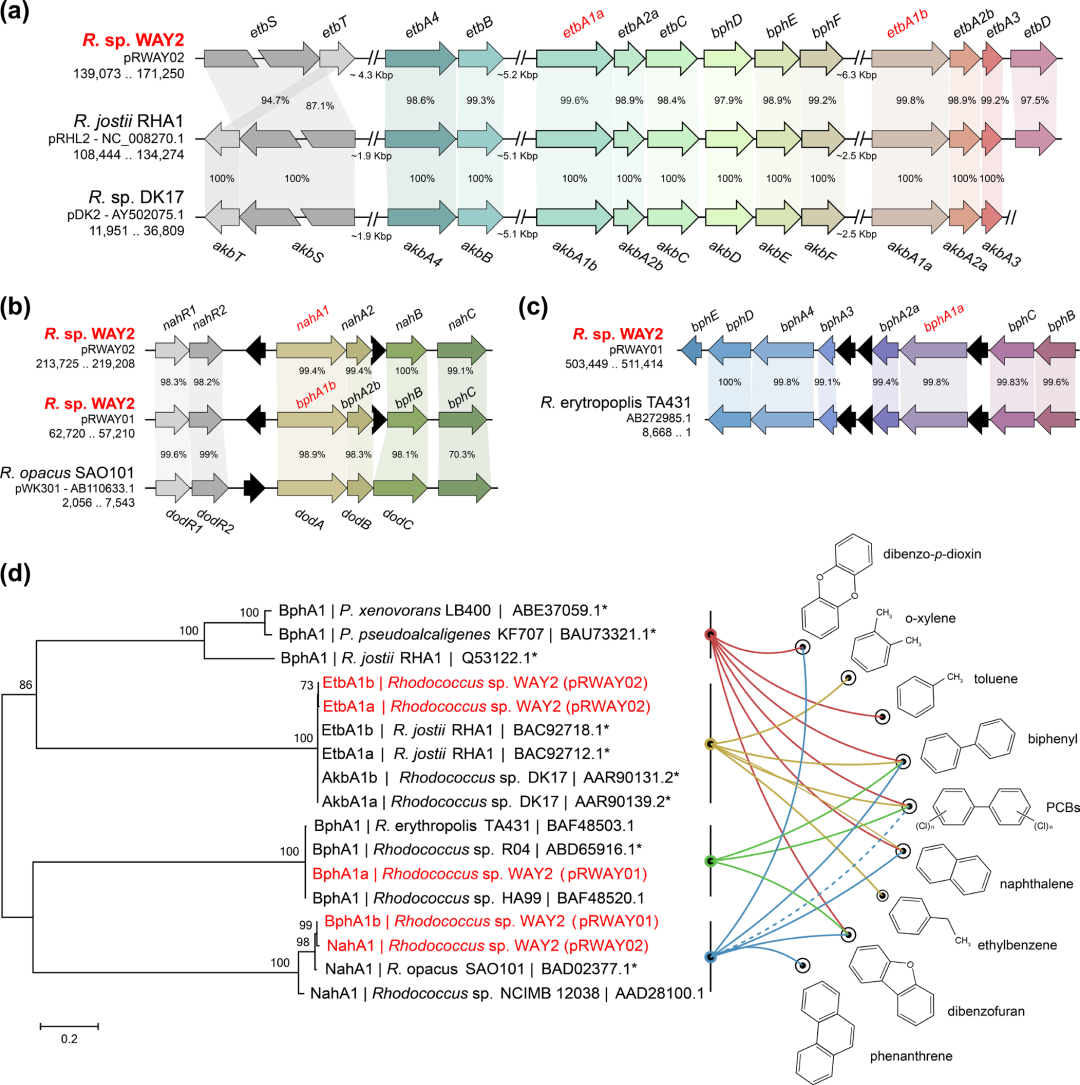
(a–c) Gene organization of *Rhodococcus* sp. WAY2 *etb*, *bph* and *nah* gene clusters and syntenic comparisons with homologous clusters in other rhodococci. Percentages indicate amino acid sequence identity. Replicons and positions of the regions shown are specified under the strain names. *etbS*/*akbS* not to scale. (d) ML phylogenetic tree based on the amino acid sequences of the ring-hydroxylating dioxygenase large subunits of *Rhodococcus* sp. WAY2 (red type) with putative involvement in degradation of aromatic compounds. Other well-characterized dioxygenases were included for comparison purposes. Bootstrap values are shown above the branches. Scale bar represents the number of substitutions per site. Validated substrates of these enzymes (marked with asterisks) are shown in the panel on the right connected with lines. The dashed line to PCBs indicates an assumed function for co-metabolism based on biphenyl degradation, but not direct evidence.

Rhodococci are known for their ability to degrade many organic aromatic compounds, usually by using ring-hydroxylating dioxygenases in the first step of their catabolism. The genome of *Rhodococcus* sp. WAY encodes 160 putative oxygenases: 67 dioxygenases and 93 monooxygenases. Among the oxygenases, there are 11 ring-hydroxylating dioxygenases distributed in the chromosome and the pRWAY01 and pRWAY02 replicons (6, 2 and 3, respectively). Of these dioxygenases, those present in the lineal replicons are clustered with other genes involved in the catabolism of biphenyl (*bph*), ethylbenzene (*etb*) and naphthalene (*nah*). These genetic clusters putatively involved in the degradation of aromatic compounds are summarized in Table 4 and Fig. 5, and are described in detail in Table S6.

**Table 4.** Gene clusters of *Rhodococcus* sp. WAY2 involved in the degradation of compounds

Gene cluster	Replicon (cluster coordinates)	Closest homologue (identity/cover.)*	Evidence of substrate specificity†
*bphA1aA2aA3A4BCDE*	pRWAY01 (503 862–510 599)	* Rhodococcus* sp. R04 ABD65916.1 (100 %/100 %)	Biphenyl/PCBs Dibenzofuran
*bphA1bA2bBD*	pRWAY01 (58 025–71 297)	* Rhodococcus opacus* SAO101 BAD02377.1 (98.9 %/100 %)	Biphenyl/PCBs Dibenzo-*p*-dioxin Naphthalene Dibenzofuran Phenanthrene
*etbA1aA2aA4BCDEFST*	pRWAY02 (139 073–161605)	* Rhodococcus jostii* RHA1 BAC92712.1 (99.6 %/99%)	Biphenyl/PCBs Naphthalene Ethylbenzene *o*-xylene
*etbA1bA2bA3D*	pRWAY02 (167 939–170 250)	* Rhodococcus jostii* RHA1 BAC92718.1 (99.8 %/99 %)	Biphenyl/PCBs Naphthalene Ethylbenzene *o*-xylene
*nahA1A2BCR1R2*	pRWAY02 (213 725–219 208)	* Rhodococcus opacus* SAO101 BAD02377.1 (98.3 %/100 %)	Biphenyl/PCBs Dibenzo-*p*-dioxin Naphthalene Dibenzofuran Phenanthrene

*Only large subunits (*bphA1*, *etbA1* and *narA1*) are considered. Percentages are based on blastp.

†See the main text for references.

The *bph* gene cluster (*bphEDA4A3A2aA1aCB*) is located in the pRWAY01 mega-plasmid. Additionally, this plasmid also harbours an incomplete *bph* cluster (*bphBA2bA1b+bphD*) and the 2HDP pathway (see above, Table 3). Similarly, the pRWAY02 replicon contains a complete *etb*/*bph* gene cluster (*etbA4B+etbA1aA2aC+bphDEF*), an incomplete *etb*/*bph* cluster (*etbA1bA2bA3D*) and a copy of the 2HDP pathway (see above, Table 3), which could also be involved in complete mineralization of ethylbenzene, biphenyl and other aromatic substrates, including PCBs and xylene, as reported in RHA1 [14, 28]. Finally, the pRWAY02 mega-plasmid also encodes the *nah* gene cluster (*nahR1R2A1A2CB*), putatively involved in naphthalene, biphenyl and different aromatic compound degradation [24]. Unlike RHA1, WAY2 is able to grow using naphthalene as a sole carbon and energy source, probably due to this *nah* gene cluster that is missing in the RHA1 genome [28]. These results evidence that pRWAY01 and pRWAY02 replicons have a predominant role in the degradation of aromatic compounds. Furthermore, compatible exchange of some subunits of the *bph*/*etb* hydroxylating dioxygenases in *R. jostii* RHA1 have also been reported [14, 23], suggesting that the combination of both *bph* and *etb* genetic clusters within the same organism could enhance the range of aromatic compounds to be degraded. The fact that WAY2 can grow using biphenyl and naphthalene as a sole carbon and energy source suggests that at least one of these *bph*/*etb*/*nah* genetic clusters is functional.

WAY2 can also use xylene as a carbon source for cell growth (Table 2). Previous studies have shown that the *bph* and *etb* gene clusters of RHA1 can metabolize *o*-xylene [28]. Furthermore, the *o*-xylene degradation *akb* gene cluster reported in *Rhodococcus* sp. DK17 [92] shares a 98 % overall amino acid sequence identity with the *etb* gene cluster of WAY2 and is identical to that of RHA1 (Fig. 5a), which strongly suggests the involvement of the WAY2 *etb* gene cluster in xylene utilization.

Altogether, WAY2 contains five large subunits of hydroxylating dioxygenases with the Rieske 2Fe-2S domain, *bphA1a*, *bphA1b*, *etbA1a*, *etbA1b* and *nahA1*, which are either clustered in the genome with complete or incomplete gene sets for the degradation of aromatic compounds in the WAY2 genome (Table 4). Phylogenetic analyses show that these dioxygenases belong to three distinct groups, with a 99 % overall sequence identity and strong syntenic conservation with previously well-characterized dioxygenase clusters (Fig. 5). While the two *etbA1* belong to the first cluster, together with their respective homologues in RHA1 and DK17, *bphA1a* and *bphA1b* belong to two distinct groups. The former is clustered with atypical *bphA1* enzymes found in *Rhodococcus* sp. strains HA99 and R04, and *Rhodococcus erythropolis* TA431 (Fig. 5c), previously described as being involved in biphenyl/PCBs and dibenzofuran degradation [25, 93]. The latter, *bphA1b,* clusters together with WAY2 *nahA1* and with known naphthalene dioxygenases including *nahA1* of *R. opacus* SA0101 [24], which has been shown to act in the dioxygenation of dibenzofuran, naphthalene, biphenyl/PCBs, dibenzo-*p*-dioxin and phenanthrene (Fig. 5b). None of these WAY2 enzymes clustered together with the typical and well-characterized biphenyl dioxygenases of RHA1, LB400 or KF707 strains (Fig. 5d). The repertory of ring-hydroxylating dioxygenases that are found in the WAY2 genome explains the wide range of aromatic degradation abilities the strain displays. Furthermore, redundancy of substrate specificity of these distinct enzymes can also enhance its degradation performance.

The pRWAY01 mega-plasmid also contains the cluster *tmoABCDEF* (Table S6) previously described as being responsible for the conversion of toluene to *p*-cresol [94]. Although WAY2 was unable to grow either with 0.5 % (v/v) toluene or its gaseous form as a sole carbon source, it can tolerate up to 1 % (v/v) toluene in LB growth medium. This was not unexpected, as it has been reported in other rhodococci [95]. The fact that the WAY2 genome does not contain any of the *pch* genes for *p*-cresol degradation [96] suggests that the *tmo* gene cluster is active in toluene detoxification. Furthermore, WAY2 incubated in MM+PAS medium with toluene (0.5 %, v/v) turned a light brown colour, suggesting partial degradation of toluene.

All the genetic systems for aromatic compound degradation that are present in the genome of *Rhodococcus* sp. WAY2 could allow its survival in aromatic hydrocarbon-polluted environments, either by metabolizing these substrates while obtaining carbon and energy as is the case of biphenyl, naphthalene and xylene, by transforming them into nontoxic by-products as is probably the case of toluene, or by co-metabolism as is the case of PCB congeners (see below).

#### Degradation of *n*-alkanes

Alkanes are the major fraction in the hydrocarbon mixtures used in fuels. Many micro-organisms are able to degrade alkanes using alkane monooxygenase enzymes for their initial aerobic hydroxylation [97]. The WAY2 chromosome putatively encodes an alkane 1-monooxygenase (*alkB*) and three long-chain alkane monooxygenases (*ladA*) involved in alkane degradation [98, 99]. The fact that WAY2 is able to grow with *n*-pentadecane, *n*-heptadecane and *n*-tetracosane as sole carbon and energy sources suggests that these enzymes are functional. This result is in agreement with previous reports that showed that rhodococci can degrade short-to-middle chain *n*-alkanes ranging from C_5_ to C_16_ [13, 100, 101] and also long-chain *n*-alkanes such as tetracosane, for which the role of *alkB* has been reported [102]. Additionally, the genome of WAY2 also harbours several clusters of genes for methane utilization (Table S6). Three of these systems are assigned to putative soluble methane monooxygenase systems (sMMO) encoded by *mmoXCYB* genes located in the chromosome, and *mmoXYBC* and *mmoX2Y2B2C2Z* genes located in the pRWAY02 replicon. The remaining cluster putatively encodes a particulate methane monooxygenase system (pMMO) encoded by *pmoBAC* genes, also located in the pRWAY02 mega-plasmid. Aside of methane oxidation, these monooxygenases have been reported to be involved in the degradation of other short-chain alkanes (up to five carbon atoms), such as propane, *n*-butane and *n*-pentane [103]. These substrates can be converted by sMMOs or pMMOs to their corresponding alcohols, which are finally transformed into formate and carbon dioxide by subsequent alcohol, formaldehyde and formate dehydrogenase reactions [97]. Previous reports show that *Rhodococcus* strains can grow using different alcohols, such as methanol, ethanol and butanol as carbon sources [104–106]. Surprisingly, our assays to test the ability of WAY2 to grow with methanol, either supplemented as a gas or in liquid (0.5 %, v/v), were unsuccessful, but WAY2 was able to use 1-butanol supplemented in vapour form as a sole carbon and energy source. However, further studies are required in order to test whether indeed these MMOs systems are active.

#### Co-metabolism of PCBs

**Fig. 6.**
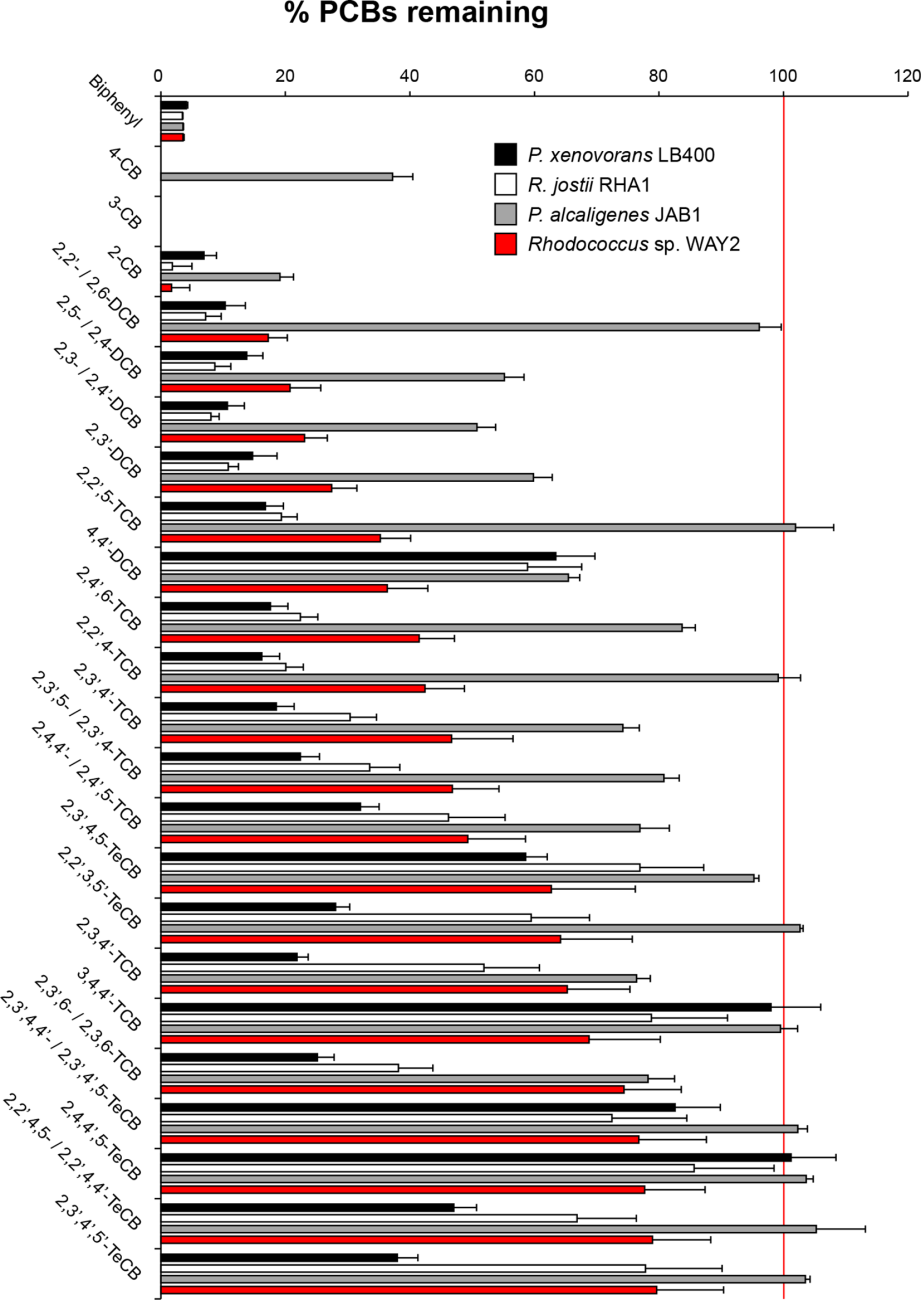
PCB congeners remaining in resting cell assays after 48 h of incubation with Delor 103 PCB mixture. Degradation abilities of *Rhodococcus* sp. WAY2, *R. jostii* RHA1, *Paraburkholderia xenovorans* LB400 and *Pseudomonas alcaligenes* JAB1 are shown compared with autoclaved controls (100%) and absolute values are specified in File S4. Mean values are represented in columns. Error bars indicate sd values from three replicates.

The ability of *Rhodococcus* sp. WAY2 to co-metabolize different PCB congeners was assessed in resting cell assays using Delor 103 as a PCB mixture. Two of the most powerful PCB degraders known to date, *R. jostii* RHA1 [69] and *Paraburkholderia xenovorans* LB400 [70, 71], as well as *Pseudomonas alcaliphila* JAB1 [72], whose *bph* gene cluster has been shown to be identical to that of *Pseudomonas pseudoalcaligenes* KF707, were also included in the assay for comparison purposes. WAY2 is able to effectively degrade 23 PCB congeners, with less than 80 % of mean PCB congener remaining after 48 h of incubation with Delor 103 (Fig. 6). Among these, WAY2 was highly effective against 14 congeners, resulting in less than 50 % of mean PCB congeners remaining after treatment. These include monochlorobiphenyls (CBs) 2-, 3- and 4-CB, dichlorobiphenyls (DCBs) 2,2′-/2,6-, 2,5-/ 2,4-, 2,3-/2,4′-, 2,3′- and 4,4′-DCB and the trichlorobiphenyls (TCBs) 2,2′,5-, 2,4′,6-, 2,2′,4-, 2,3′,4′-, 2,3′,5-/2,3′,4- and 2,4,4′-/2,4′,5-TCB (Fig. 6).

The strikingly similar congener specificity of WAY2 and RHA1 could be due to the high sequence homology of the *etb* gene cluster, which has been shown in RHA1 to have a predominant role in the degradation of highly chlorinated PCBs, preferably *ortho*-substituted [23]. Compared to JAB1, WAY2 performed better in all the PCB congeners tested (Fig. 6). This could be due to the three independent genetic systems (*bph*, *etb* and *nah*) that are putatively involved in the degradation of PCBs, although further analysis to test the implications of each system in the degradation of specific congeners should be properly addressed. Although there are 23 PCB congeners for which WAY2 has shown to be active and of similar congener spectrum as RHA1, it is important to note that RHA1 can effectively degrade 54 PCB congeners [69], suggesting that the *etb* gene cluster is only partially responsible for the degradation of certain congeners. Furthermore, WAY2 performed better than RHA1 and LB400 in the case of 4,4′-DCB, and better than LB400 in the case of 3,4,4′-TCB and 2,4,4′,5-TCB congeners (Fig. 6).

**Fig. 7.**
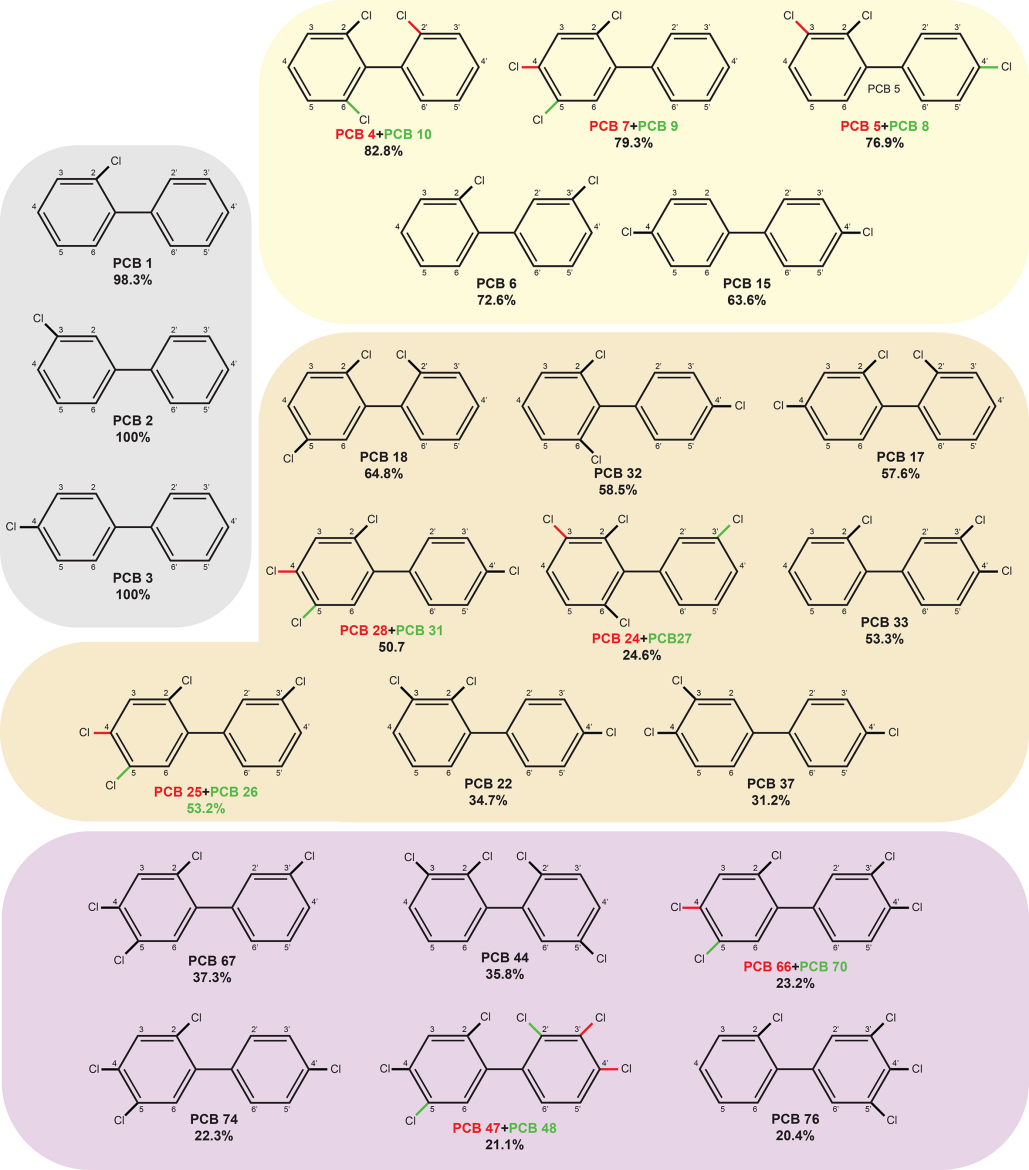
PCB congeners degraded by *Rhodococcus* sp. WAY2. Only congeners with more than 20% of degradation in resting cell assays are considered. The different coloured boxes indicate monochlorinated biphenyls (grey), dichlorinated biphenyls (yellow), trichlorinated biphenyls (orange) and tetrachlorinated biphenyls (violet). The name and percentage of degradation of each PCB congener is indicated under each one. Different colours of chlorine substitutions are indicated for those PCB congeners within the same retention times in GC-MS.

Our results also show that WAY2 has a preference for *ortho*- and *para*- substituted PCB congeners (Fig. 7). All the congeners degraded by WAY2 (except 3-CB) contain chlorine atoms in *ortho*- and/or *para*- positions. This result is congruent with the *etb* gene cluster being responsible for the degradation of most PCB congeners, as previously suggested [23].

Additionally, the *bphA1a* gene of WAY2, which is identical to that of the *Rhodococcus sp*. R04 strain (Table 4), could be responsible for the degradation of specific congeners, such as 3-CB, 4,4′-DCB and 2,4′,5-TCB, as reported elsewhere [93], although these are probably common substrates of different *bph*/*etb*/*nah* clusters in WAY2.

### Environmental adaption

Members of the genus *Rhodococcus* are known to accumulate diverse storage compounds, including glycogen, polyphosphate, TAGs and PHAs, which could be related to environmental adaptions to fluctuating nutritional states and metabolic balance [16, 17, 107, 108]. Genes for the biosynthesis and degradation of glycogen (*glgXBEPUCA* and *pgm*), polyphosphate metabolism (*ppk* and *ppx*) and wax ester synthase/acyl-CoA:diacylglycerol acyltransferase (WS/DGAT, *aft* gene) involved in TAG metabolism are also present in the WAY2 chromosome (Table S7). Unlike RHA1, none of these genes are found within the WAY2 mega-plasmids. Furthermore, WAY2 possesses half of *aft* genes (seven) compared to RHA1 [17]. Interestingly, although the chromosome of WAY2 contains five putative PHA synthases (*phaC*), no PHA depolymerase is found, raising questions whether WAY2 is able to use PHA as a storage compound.

However, lithoautotrophy has been previously described in several *Rhodococcus *, and the genes involving the production of energy and carbon intermediates from inorganic H_2_, CO and CO_2_ have been discovered [89, 109–111]. The chromosome of WAY2 contains two clusters of genes encoding putative NiFe hydrogenase systems (*hypABDC+hyaAB* and *hypDCEF*), suggesting that WAY2 could be able to oxidize H_2_ to obtain energy. Additionally, two major gene clusters for carbon monoxide utilization (*coxMLSED* and *coxEGDFLSMI*) are encoded by the WAY2 chromosome, while four carbonic anhydrases are present in the chromosome and one in the pRWAY03 mega-plasmid (Table S7). All these data suggest that *Rhodococcus* sp. WAY2 is adapted to nutritional starvation during environmental changes by using auxiliary mechanisms for energy production and carbon utilization and storage.

In addition to nutritional stress adaption, the genome of *Rhodococcus* sp. WAY2 contains multiple cold shock proteins, including six CspA paralogs and a single copy of CspC (Table S7), which are involved in the correct folding of proteins that have been putatively linked to the ability of other rhodococci to grow at subzero temperatures [18]. Furthermore, compatible osmolytes, such as glycine betaine, choline, ectoine and trehalose, play an important role at low temperatures by preventing ice formation and by helping to resist the osmotic pressures of subzero temperatures [112, 113]. The chromosome of WAY2 contains multiple glycine betaine and choline transporters (*proP* and *betT*) and the betaine, ectoine and trehalose biosynthetic genes (*betAB*, *ectABC* and *tresSZY*, respectively; Table S7). These genes are found predominantly in the WAY2 chromosome and could explain its ability to grow at 5 °C.

### GIs and heavy metal resistance

GIs are a common feature in bacterial genomes, and are involved in genome evolution by providing novel genes that are often related to antimicrobial and heavy metal resistance [114–116] or acquisition of entire metabolic pathways [117]. The genome of WAY2 was screened using the IslandPath-DIMOB and SIGI-HMM sequence composition-based prediction methods to identify GIs. WAY2 contains a total of 30 GIs, most of which are found within its chromosome (16), ranging from 4 to 43 kbp in length, while the linear replicon pRWAY01 contains 8 GIs ranging from 5 to 36 kbp, pRWAY02 contains 2 GIs of around 7 kbp each, and pRWAY03 contains 4 GIs, ranging from 5 to 8 kbp (Table 5 and S8). No GI was found in the small circular plasmid pRWAY04. The length of these GIs per replicon represents 4.4 % of the chromosome length and 11.6, 3 and 6.9 % of the linear replicon’s lengths, respectively. Similarly, GIs are also present in other rhodococci, representing the 7.4, 9.2 and 11 % of the genomes of *Rhodococcus rhodochrous* EP4, *R. jostii* RHA1 and *R. opacus* M213, respectively [118, 119]. Unlike in these rhodococci, whose GIs encode complete catabolic pathways or genes involved in the biodegradation of organic compounds, most of the genes included within WAY2 GIs do not have a predicted function or are poorly characterized. However, certain GIs present in all replicons of WAY2 contain clusters of genes involved in resistance to heavy metals and metalloids. For instance, the cluster of genes *arsCBR* involved in arsenate reduction is found in a chromosome GI, while a multicopper oxidase (*mco*), the copper resistance protein CopC and a mercuric ion reductase (*merA*) are present in pRWAY01 GIs (Table S8). One of the GIs of the pRWAY02 replicon also contains the tellurium-resistance protein TerD, and a GI of the pRWAY03 contains a copper chaperone (*copZ*), the copper-translocating P-type ATPase (*copA*) and the repressor CsoR of the *copZA* operon. Furthermore, the linear replicons of WAY2 contain many of these heavy metal resistance systems and others that are not associated with the predicted GIs. *Rhodococcus* strains have been proven useful for the removal of heavy metals by biosorption [120, 121], for which heavy metal resistance mechanisms are required in order to avoid its toxicity [122]. The presence of heavy metal resistance mechanisms along with the WAY2 ability to degrade several chain-length alkanes and PAHs could be of use for bioremediation of hydrocarbon-polluted environments in which heavy metals are also frequently present [123].

**Table 5.** GIs present in the genome of *Rhodococcus* sp. WAY2 See Table S8 for extended information.

Replicon	No. of GIs	Total size (bp)	Size range (kbp)	% replicon size	No. of genes
Chromosome	16	289 487	4.1–43.2	4.4	267
pRWAY01	8	115 699	5.1–35.8	11.6	133
pRWAY02	2	13 883	6.9–7	3	17
pRWAY03	4	24 573	5–8	6.9	33

The results show that a small fraction of novel genes for resistance of metal and metalloid toxicity could have been acquired by horizontal gene transfer, as they are found within GIs. However, the fact that none of the catabolic pathways for the degradation of aromatics and alkanes described herein are found in GIs, and that multiple metal and metalloid resistance proteins are also outside GIs, is congruent with the hyper-recombinant mechanism described in *Rhodococcus* genomes to acquire novel catabolic pathways [19, 20] that do not fall into GIs prediction.

In conclusion, the novel PCB degrader *Rhodococcus* sp. WAY2 possesses a multipartite genome in which there is a division of functions encoded by its chromosome and extrachromosomal replicons. While the chromosome harbours core cellular functions and central metabolic pathways, the linear replicons provide the machinery for the biodegradation of multiple aromatic compounds, including PCBs. The wide range of aromatic compounds WAY2 is able to degrade is explained by the presence of five distinct genetic clusters. These clusters also explain the number of PCB congeners degraded by WAY2 in the resting cell assay. In addition, WAY2 is able to grow with different chain-length alkanes as sole carbon source and harbours different heavy metals resistance mechanisms, some of which are found within GIs. This make this strain particularly suited for bioremediation of fuel-polluted soils where PAHs and alkanes are the major contaminants, often accompanied with heavy metals and metalloids. Furthermore, adaption strategies to nutritional starvation by using storage compounds and putative lithoautotrophic mechanisms, along with cold shock proteins and compatible osmolytes found predominantly in the chromosome of *Rhodococcus* sp. WAY2, show that this strain is adapted to a wide range of environmental conditions. The phylogenetic and phylogenomic analyses of *Rhodococcus* sp. WAY2 have provided insights into a putative new species of the genus *Rhodococcus *, which appears extraordinarily suited for bioremediation applications.

## Data Bibliography

*Rhodococcus* sp. WAY2 genome sequencing and assembly. National Center for Biotechnology Information (NCBI), BioProject ID PRJNA580179.

## Supplementary Data

Supplementary material 1
