# Supplementary material for: Analysis of the biodegradative and adaptive potential of the novel polychlorinated biphenyl degrader Rhodococcus sp. WAY2 revealed by its complete genome sequence
Source: Microb Genom. 2020 Apr 2;6(4):e000363. doi: 10.1099/mgen.0.000363 (PMC7276702; doi:10.1099/mgen.0.000363)
Supplement: Supplementary material 1 [file mgen-6-363-s001.pdf]

Supplementary material of: Analysis of the biodegradative and adaptive potential of the novel PCB degrader *Rhodococcus* sp. WAY2 revealed by its complete genome sequence

Daniel Garrido-Sanz[1], Paula Sansegundo-Lobato[1], Miguel Redondo-Nieto[1], Jachym Suman[2], Tomas Cajthaml[3], Esther Blanco-Romero[1], Marta Martin[1], Ondrej Uhlik[2] and Rafael Rivilla[1,*]

Supplementary File S1. Analysis of the *Rhodococcus* sp. WAY2 plasmids.

Methods

To test the topology of the pRWAY01, pRWAY02 and pRWAY03 replicons, different sets of primers were designed at least at 800 bp from each replicon end to avoid telomeric sequences. A reverse primer in each of the left ends of the three replicons (1L3, 2L3 and 3L3), was designed in order to amplify with its respective forward primer in the replicons right ends (1R1, 2R1, 3R1) only in case of circular topology. Additionally, forward primers in the left ends (1L2, 2L1 and 3L2) and reverse primers in the right ends (1R3, 2R2 and 3R3), were designed to be combined with the ones previously described as positive controls. The sequences of the primers, Tm, positions in each replicon and combinations used can be seen below and also a scheme of the priming sites. For the small circular plasmid pRWAY04, two sets of primers (4F1-4R3 and 4F2-4R4) were designed to amplify ~10 Kbps of overlapping fragments that covers the total plasmid length (14.8 Kbps). Melting temperature of the primers, absence of dimerization and harping formation and lack of secondary priming sites were assessed using the OligoAnalyzer tool available at https://eu.idtdna.com/calc/analyzer.

For pRWAY01, pRWAY02 and pRWAY03, PCR reactions were carried out in a total volume of 25 µL containing 2.5 µL of 10x PCB buffer $MgCl_2$ free, 1 µL $MgCl_2$ 50 mM, 0.5 µL of DMSO (dimethyl sulfoxide) at 10% (v/v), 0.5 µL dNTP mix 10 mM (2.5 mM each), 1 µL of each primer at 10 µM, 1 µL of *Taq* DNA polymerase 1 U/µL (Biotools) and 1 µL of *Rhodococcus* sp. WAY2 genomic DNA at a 30-50 ng/µL. The cycling conditions consisted in a first denaturation step at 95 ºC for 5 min followed by 27 cycles of amplification (1 min denaturation at 95 ºC, 45 s of primer annealing at 60 ºC and an elongation step at 72 ºC for 1.5 min) followed by a final elongation step at 72 ºC for 7 min. For pRWAY04, PCR reactions were carried out in a total volume of 25 µL containing 12.5 µL of Master Mix Q5' High Fidelity 2x (New England BioLabs), 1.25 µL of each primer at 10 µM, 0.5 µL of DMSO at 10% (v/v) and 9.5 µL of WAY2 genomic DNA at a 30-50 ng/uL. The cycling conditions consisted inn a first denaturation step at 98 ºC for 3 min followed by 35 cycles of amplification (10 s denaturation at 98 ºC, 30 s of primer annealing at 71 ºC and an elongation step at 72 ºC for 7 min) followed by a final elongation step at 72 ºC for 3 min. PCR products were electrophoretically separated in 0.8% (w/v) agarose gels and dyed with Gel Red.

Results

All the primer combinations to test the linear topology of the pRWAY01, pRWAY02 and pRWAY03 plasmids resulted in positive amplification of the controls (image), with amplicon sizes congruent with the theoretically expected, and negative amplification with the primer combinations designed to amplify only in case of circular topology (image: 1L3-1R1, 2L3-2R1 and 3L3-3R1, lanes 3, 6 and 9). These results validate the linear topology of the pRWAY01, pRWAY02 and pRWAY03 replicons of *Rhodococcus* sp. WAY2, as predicted by its genome sequencing. The two PCRs of the pRWAY04

circular small plasmid resulted in amplicon sizes of ~10 Kbps, congruent with the theoretically expected and validate the circular topology of this plasmid.

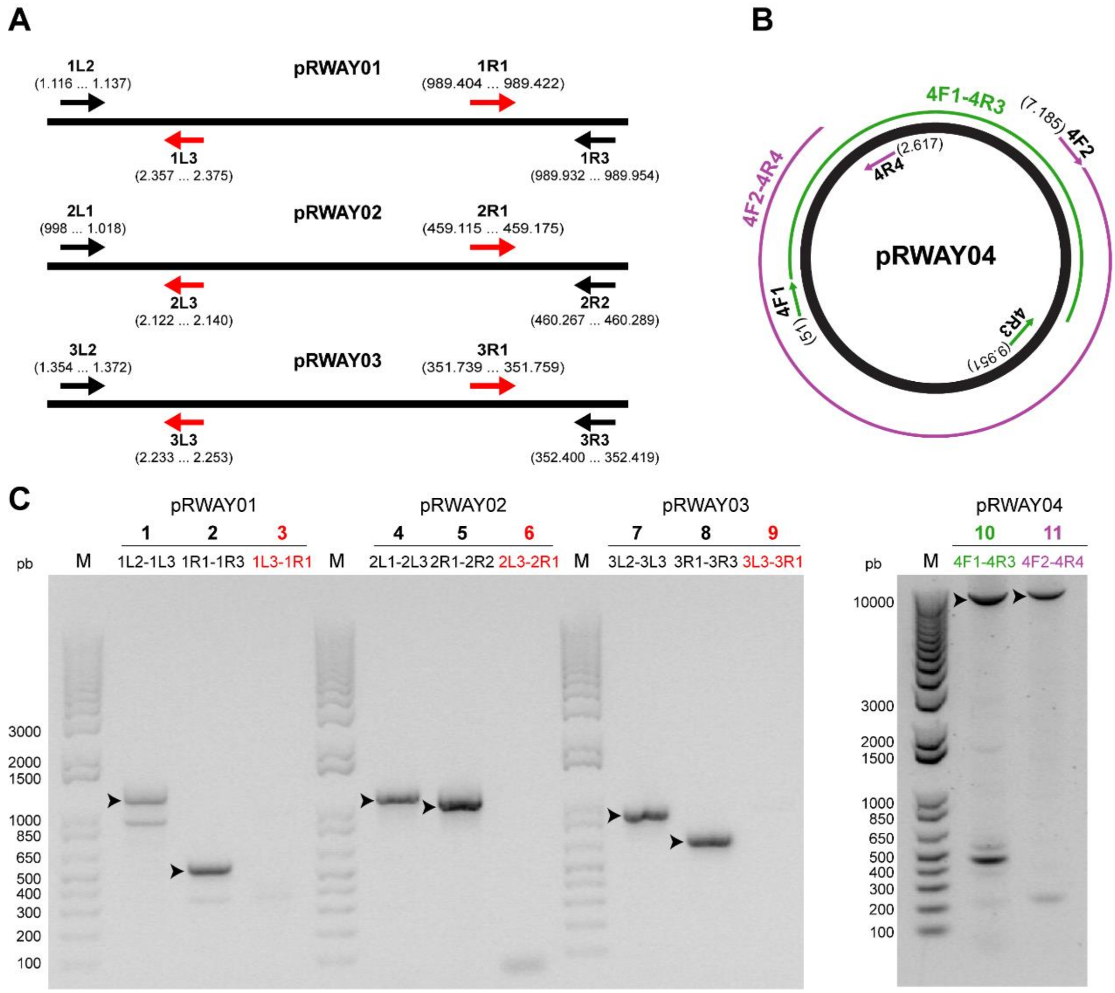

(**A**) Schematic representation of the primers designed to test the topology of the pRWAY01, pRWAY02 and pRWAY03 replicons of *Rhodococcus* sp. WAY2. Red arrows indicate the combination of primers which will result in positive amplification in case of circular topology. (**B**) Schematic representation of the primers designed to test the topology of the small pRWAY04 replicon. (**C**) PCR results in agarose gels at 0.8% (w/v). Black typing lanes show positive control amplicons (arrows), while red typing lanes (3, 6 and 9) show no amplification, which correspond with a linear topology of the pRWAY01, pRWAY02 and pRWAY03 replicons. Lanes 10 and 11 show the PCR products of the pRWAY04 small replicon, congruent with a circular topology.

Primers designed to test the topology of the *Rhodococcus* sp. WAY2 extrachromosomal replicons.

Replicon	Name	Forward (F) / Reverse (R)	Sequence (5' – 3')	Tm (°C)	Position
**pRWAY01**	1L2	F	CAAACTCAGATCCCGCCTCACC	60,0	1.116 .. 1.137
	1L3	R	CAGCCGCGAGTACGACCTC	60,4	2.357 .. 2.375
	1R1	F	GAACCGGAACCGCTGACCC	61,2	989.404 .. 989.422
	1R3	R	CGTTGGCTGCCTTCAAGTGGAAC	61,0	989.932 .. 989.954
**pRWAY02**	2L1	F	GCATGACGGTGTCGACGTGTC	60,7	998 .. 1.018
	2L3	R	CCCGAACTCTCGCCACAGC	61,1	2.122 .. 2.140
	2R1	F	GGAGGGTTCAGTGTTCGGCTG	60,4	459.155 .. 459.175
	2R2	R	CCGGTTCCTGAGAGGATGCGTTC	61,7	460.267 .. 460.289
**pRWAY03**	3L2	F	CGTCCTCCACCATGAGCCCC	60,6	1.354 .. 1.372
	3L3	R	CATTCCCGCAACCAGTGGACC	61,0	2.233 .. 2.253
	3R1	F	GTGTCGGTGACATCGAGTCGC	60,3	351.739 .. 351.759
	3R3	R	CGAAGGGGTGATCCGGGAAC	60,3	352.400 .. 352.419
**pRWAY04**	4F1	F	CTGTCTCCGGCTCGAGTGC	60,5	51 .. 69
	4F2	F	GGAGATGCTGCAGGAAACAGGC	60,8	7.185 .. 7.206
	4R3	R	GGGCGGTGCGTATTTACTTCGC	61,0	9.951 .. 9.972
	4R4	R	CTCCCAATCCCCGTACAGTCG	59,5	2.617 .. 2.637

Primer combinations used.

Replicon	Forward	Reverse	Expected amplicon length (bp)
**pRWAY01**	1L2	1L3	1.260
	1R1	1R3	551
	1L3	1R1	-
**pRWAY02**	2L1	2L3	1.143
	2R1	2R2	1.135
	2L3	2R1	-
**pRWAY03**	3L2	3L3	900
	3R1	3R3	681
	3L3	3R1	-
**pRWAY04**	4F1	4R3	9.922
	4F2	4R4	10.306

Supplementary File S2. Analysis of the invertron-type telomeric nucleotide sequences of *Rhodococcus* sp. WAY2 linear mega-plasmids and identification of Terminal Inverted Repeats (RITs).

A

```
                          10        20        30        40        50        60
pRWAY01_R      -CCGCGGCGCTGCTCCCGGTGCTCCGCACCGGGAGACGGGGGCGAGTCGCTCCGCTCCC-
pRWAY01_L      -CCGCGGCGCTGCTCCCGGCGCTCCGCGCCGGGAGGCGGAG-CGAGTCGCTTCGCTCCC-
pRWAY02_R      -CCGCGGCGCTGCTCCCGGCGCTCCGCGCCGGGAGACGGAG-CGAGTCGCTTCGCTCCC-
pRWAY02_L      -CCGCGGCGCTGCTCCCGGCGCTCCGCGCCGGGAGACGGGG-CGAGTCGCTGCGCTCCC-
pRWAY03_R      CCCGCGGCGCTGCTCCCGGCGCTCCGCGCCGGGAGGCGGGG-CGAGTCGCTTCGCTCCCA
RHA1_chr_R     -CCGCGGCGCTGCTCCCGGCGCTCCGCGCCGGGAGACGGGG-CGAGTCGCTGCGCTCCC-
RHA1_chr_L     -CCGCGGCGCTGCTCCCGGCGCTCCGCGCCGGGAGACGGGG-CGAGTCGCTGCGCTCCC-
pRHL1_R        -CCGCGGCGCTGCTCCCGGTGCTCCGCACCGGGAGACGGGGGCGAGTCGCTTCGCTCCC-
pRHL1_L        -CCGCGGCGCTGCTCCGGGTGCTCCGCACCCGGAGACGGGG-CGAGTCGCTTCGCTCCC-
pRHL2_L        -CCGCGGCGCTGCCCCCGACGCTCCGCGCCGGGAGACGGGG-CGAGTCGCTCCGCTCCC-
pRHL3_R        -CCGCGGCGCTGCCCCCGGCGCTCCGCGCCGTGAGACGGGG-CGAGTCGCTTCGCTCCCC
pRHL3_L        -CCGCGGCGCTGCTCCCGGCGCTCCGCGCCGGGAGACGGGG-CGAGTCGCTCCGCTCCC-
B4_chr_R       --CGCGGCGCTGCTCCCGGCGCTCCGCGCCGGGAGACGGGG-CGAGTCGCTGCGCTCCC-
B4_chr_L       ---GCGGCGCTGCTCCCGGCGCTCCGCGCCGGGAGACGGGG-CGAGTCGCTTCGCTCCC-
pROB01_L       ---GCGGCGCTGCTCCCGGCGCTCCGCGCCGGGAGACGGGG-CGAGTCGCTTCGCTCCC-
pROB02_R       --CGCGGCGCTGCTCCCGGCGCTCCGCGCCGGGAGACGGGG-CGAGTCGCTTCGCTCCC-
pROB02_L       ---GCGGCGCTGCTCCCGGTGCTCCGCACCGGGAGACGGGG-CGAGTCGCTGCGCTCCC-
pR1CP1_R       -CCGCGGCGCTGCTCCCGGCGCTCCGCGCCGGGAGACGGGG-CGAGTCGCTTCGCTCCC-
pR1CP1_L       --CGCGGCGCTGCTTCCGGCGCTCCGCGCCGGGAGACGGAG-CGAGTCGCTTCGCTCCC-
                  **********  * *  ******* **  *** *** * ********* *******

                          70        80        90       100       110       120
pRWAY01_R      CGCCAGGGAGCCGCTTGCGCGGCTCGCAGTGGGTCGATTCCCGTTGCCGTCGATCGAGTC
pRWAY01_L      CGCCAGGGAGCCGCT-GCGCGGCTCGCAGTGGGCCCATTCCCGTTGCCGTCGATCGAGTC
pRWAY02_R      CGCCAGGGAGCCGCT-GCGCGGCTCGCAGTGAGCT--GTCCCGTTGCCGTCGATCGAGTC
pRWAY02_L      CGCCAGGGAGCCGCT-GCGCGGCTCGCAGTGGGTCCATTCCCGTTGCCGTCGATCGAGTC
pRWAY03_R      CGCCAGGGAGCCGCT-GCGCGGCTCGCAGTGGGTCCATTCCCGTTGCCGTCGATCGAGTC
RHA1_chr_R     CGCCAGGGAGCCGCT-GCGCGGCTCGCAGTGGGTCGATTCCCGTTGCCGTCGATCGAGTC
RHA1_chr_L     CGCCAGGGAGCCGCT-GCGCGGCTCGCAGTGGGTCGATTCCCGTTGCCGTCGATCGAGTC
pRHL1_R        CGCCAGGGAGCCGCT-GCGCGCCTCTCAGTGGGTCCATTCCCGTTGCCGTCGATCGAGTC
pRHL1_L        CGCCAGGGAGCCGCT-GCGCGGCTCGCAGTGGGTCCATTTCCGTTGCCGTCGATCGAGTC
pRHL2_L        CGCCAGGGAGCCGCT-ACGCGGCTCGCAGTGGGTCGATTCCCGTTGCCGTCGATCGAGTC
pRHL3_R        CGCCAGGGAGCCGCT-GCGCGGCTCGCAGTGGGTCGATTCCCGTTGCCGTCGATCGAGTC
pRHL3_L        CGCCAGGGAGCCGCT-GCGCGGCTCGCAGTGGGTCCATTCCCGTTGCCGTCGATCGAGTC
B4_chr_R       CGCCAGGGAGCCGCT-GCGCGGCTCGCAGTGGGTCCACTCCCGTTGCCGACGATCGAGTC
B4_chr_L       CGCCAGGGAGCCGCT-GCGCGGCTCGCAGTGGGTCGATTCCCGTTGCCGTCGATCGAGTC
pROB01_L       CGCCAGGGAGCCGCT-GCGCGGCTCGCAGTGGGCCGATTCCCGTTGCCGTCGATCGAGTC
pROB02_R       CGCCAGGGAGCCGCT-GCGCGGCTCGCGGTGGGTCCATTCCCGTTGCCGACGATCGAGTC
pROB02_L       CGCCAGGGAGCCGCT-GCGCGGCTCGCAGTGGGTCAATTCCCGTTGCCGTCGATCGAGTC
pR1CP1_R       CGCCAGGGAGCCGCT-GCGCGGCTCGCAGTGGGTTGATTCCCGTTGCCGTCGATCGAGTC
pR1CP1_L       CGCCAGGGAGCCGCT-GCGCGGCTCGCAGTGGGTCCATTCCCGTTGCCGTCGATCGAGTC
               ***************  **** *** * *** *     * ********* **********

                         130       140       150       160       170       180
pRWAY01_R      GCTTCGCTCCTCTGAGTTT-CCGAGATTGCGTTCTCACCTGTGCTTTTACTCGTCACGTT
pRWAY01_L      GCTTCGCTCCTCTGAGTTT-CCAAAATTAGGCTCTCACCTGAGCCTTTAATCGCCCAGTT
pRWAY02_R      GCTTCGCTCCTCTGAGTTT-CCAAGATTAGGTTCTCACCTGCATCTTTCATCGTCCCGTT
pRWAY02_L      GCTTCGCTCCTCTGAATTT-CCGAGATTAGGCTCTCACCTGGGCTTTTCATTGGCATATT
pRWAY03_R      GCTTCTCTCCTCTGAGTTT-CCGAGATTAGGTTCTCACCTGCACTTTTACTCGTTCCGTC
RHA1_chr_R     GCTTCGCTCCTCTGAGTTT-CCGAGATTAGGTTCTCGCCTGCACTTTTCATCGTCCCGTT
RHA1_chr_L     GCTTCGCTCCTCTGAGTTT-CCGAGATTAGGTTCTCGCCTGCACTTTTCATCGTCCCGTT
pRHL1_R        GCTTCGCTCCTCTGAGTTT-CCGAGATTAGGTTCTCACCTGCACTTTTCATCGTCCCGTT
pRHL1_L        GCTTCGCTCCTCTGGGTTT-CCGAGATTAGGTTATTACCTGCACTTTTCATCGTCCAGAT
pRHL2_L        GCTTCGCTCCTCTGAGTTT-TCGAGATTAGGTTCTCACCTGCGCTTTTACTCGTCCCGTT
pRHL3_R        GCTTCGCTCCTCTGAGTTT-CGGAGATTAGGTTCTCACCTGTGCTTTTAGTCGTCCCGTT
pRHL3_L        GCTTCGCTCCTCCGAGTTT-CCGAGATTAGGTTCTCACCTGCGCTTTTACTCGTCCCGTT
B4_chr_R       GCTTCGCTCCTCTGAGTTT-CCGAGATTAGGCTCTCACCAAATATTTTCATCGGCACGTT
B4_chr_L       GCTTCGCTCCTCTGAGTTT-CCGAGATTGGGTTTTCACCTGCGCTTTTAATCGCACCGTT
pROB01_L       GCTTCGCTCCTCTGAGTTTTCCAAGATTAGGTTCTCACCTGCACTTTTACTCGGAACGTT
pROB02_R       GCTTCGCTCCTCTGAGTTT-CCGAGATTAGGCTCCCACCAAAGCTTTTCATCGGCACGTT
pROB02_L       GCTTCGCTCCTCTGAGTTT-CCGAAATTAGGTTCTCAACTGCTCTTTTCATCGGAAAGCT
pR1CP1_R       GCTTCGCTCCTCTGAGATT-CCGAGATTAAGTTCTCACCTGCCCTTTTCCTCGTCCCGTT
pR1CP1_L       GCTTCGCTCCTCTGAGTTT-CCGAAATTAGGCTCTCACCTGCGCTTTTACTCGTCCCGTT
               ***** ****** *   **    * ***  * *     *      ***  * *
```

(**A**)

Comparison of *Rhodococcus* sp. WAY2 linear mega-plasmids telomeres (except for pRWAY03 left end) with linear replicons of other rhodococci: *R. jostii* RHA1 chromosome and plasmids pRHL1, pRHL2 and pRHL3, *R. opacus* B4 chromosome and plasmids pROB01, pROB02 and *R. opacus* 1CP plasmid pR1CP1. Conserved nucleotides are indicated with asterisks. The two sets of inverted repeats are highlighted with blue boxes and the GCTXCGC central motif with red boxes and bold. Non-conserved nucleotides in these inverted repeats and central motif are red typed.

B

```
                     10        20        30        40        50        60
pRWAY03_L   CCGTCGGGGAGGGTACCCCGGGCTGCGCCCGGGGCCTGAACCGACTGGGCGGCAAGCCGC
pRHL2_R     CCGTCGGG-AGGGTACCC-GGGCTGCGCCCGGGGCCTGAACCGACTGGGCGGCAAGCCGC
pROB01_R    --GTCGGGGAGGGTACCCCGGGCTGCGCCCGGGGCCTGAACCGACTGGGCGGCAAGCCGC
              ****** ********* *****************************************

                     10        20        30        40        50        60
pRWAY03_L   CCAATAACGGAATTCACCCA-CGCGCTGCGCGCTGGGTCACGGGTTAGCTTGCGGTATAA
pRHL2_R     CCAATAACGGAATTCACCCAACGCGCTGCGCGCAGGGTCACGGGTTAGCTTGCTGCATAA
pROB01_R    CCAATAACGGAATTCACCCA-CGCGCTGCGCGCTGGGTCACGGGTTAGCTTGTTGCATAA
            ******************** ************ ******************  * ****

                     10        20        30        40        50        60
pRWAY03_L   CGGAATCTGGAACAAGCAAAATGATCACTTCGGAAGTGTTCATTACCAACGGGATTGGCG
pRHL2_R     CGGAATCCGGAAAAAGCAAAATGATCACTTCGGAAGTGTTCATTACCAACGGAATTGGCC
pROB01_R    CGGAATCCGGAGAAAACAAAATGATCACTTCGGAAGTCTTCATTACCAACGGGATTGGCC
            ******* ***  ** ********************* ************** ******
```

(**B**) Comparison of *Rhodococcus* sp. WAY2 linear mega-plasmids pRWAY03 left end with homologous telomeric sequences of *R. jostii* RHA1 plasmid pRHL2 right end and *R. opacus* B4 plasmid pROB01 right end. Conserved nucleotides are indicated with asterisks. The two sets of inverted repeats are highlighted with blue boxes and the GCTXCGC central motif with red boxes and bold. Non-conserved nucleotides in these inverted repeats and central motif are red typed.

Supplementary File S3. Whole genome-based taxonomic analysis of *Rhodococcus* sp. WAY2 using the Type (Strain) Genome Server (TYGS).

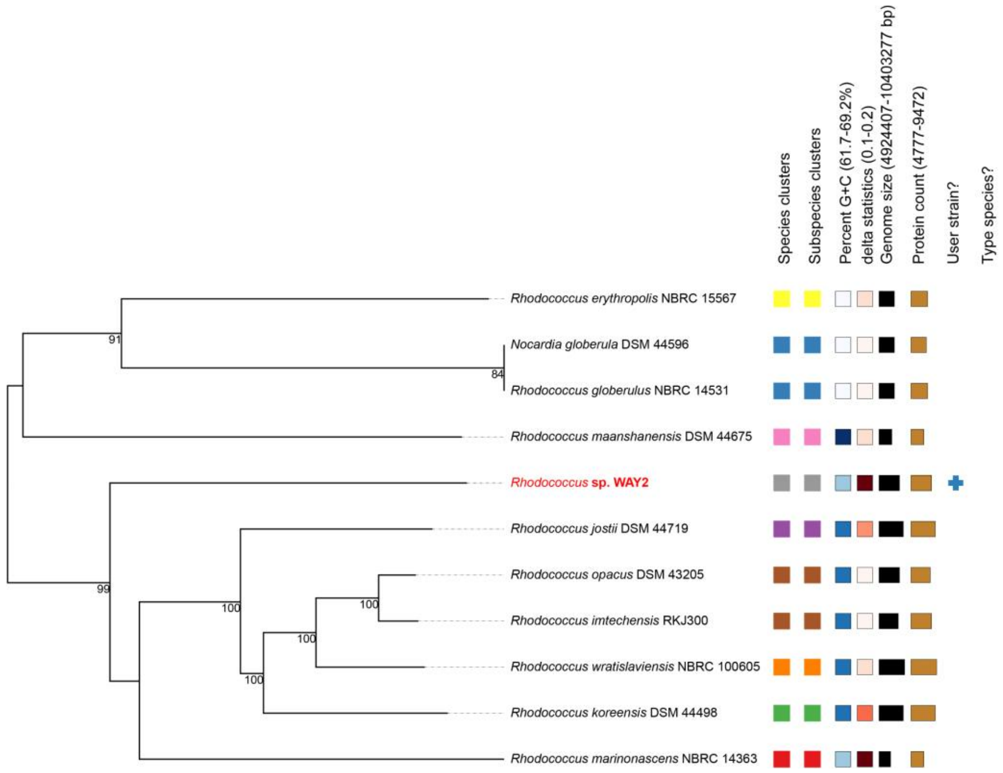

GBDP tree based in whole-genome sequences of the 10 closest type strain genomes to *R.* sp. WAY2 (red typing) inferred with FastME from GBDP distances. Tree was rooted at midpoint. Pseudo-bootstrap support values are shown below branches and were calculated over 100 replicates, with an average branch support of 91.5%.

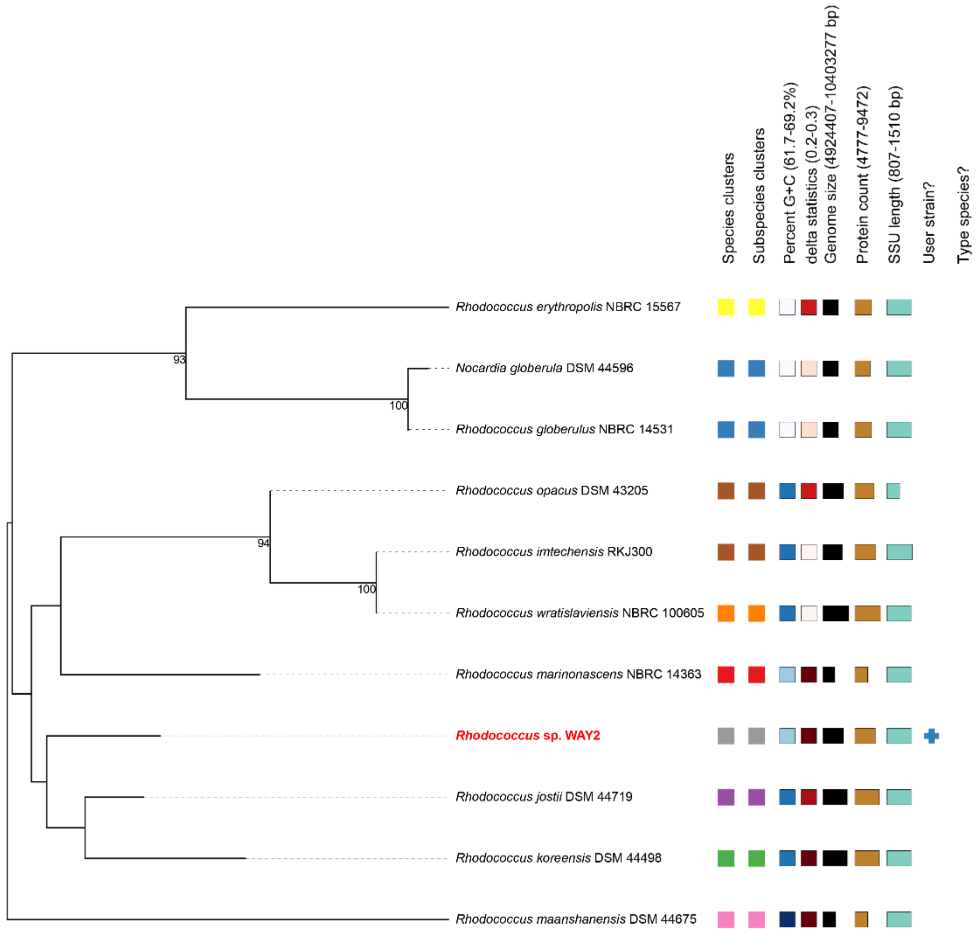

GBDP tree based in 16S rRNA gene sequences of the 10 closest type strain to *R.* sp. WAY2 (red typing) inferred with FastME from GBDP distances. Tree was rooted at midpoint. Pseudo-bootstrap support values are shown below branches and were calculated over 100 replicates, with an average branch support of 62.5%.

Pairwise comparisons of *Rhodococcus* sp. WAY2 against the 10 closest type strain genomes.

**Query strain**	**Subject strain**	**dDDH (d4, in %)**	**C.I. (d4, in %)**	**%GC content difference**
Rhodococcus sp. WAY2	*Rhodococcus opacus* DSM 43205	27.7	[25.4 - 30.2]	2.8
Rhodococcus sp. WAY2	*Rhodococcus imtechensis* RKJ300	27.6	[25.2 - 30.1]	2.75
Rhodococcus sp. WAY2	*Rhodococcus wratislaviensis* NBRC 100605	26.8	[24.4 - 29.2]	2.3
Rhodococcus sp. WAY2	*Rhodococcus jostii* DSM 44719	26.7	[24.4 - 29.2]	2.41
Rhodococcus sp. WAY2	*Rhodococcus koreensis* DSM 44498	26.5	[24.1 - 28.9]	2.9
Rhodococcus sp. WAY2	*Rhodococcus marinonascens* NBRC 14363	22.9	[20.6 - 25.3]	0.07
Rhodococcus sp. WAY2	*Rhodococcus maanshanensis* DSM 44675	20.2	[18.0 - 22.7]	4.69
Rhodococcus sp. WAY2	*Rhodococcus erythropolis* NBRC 15567	20.1	[17.9 - 22.5]	2.08
Rhodococcus sp. WAY2	*Rhodococcus globerulus* NBRC 14531	19.4	[17.2 - 21.8]	2.8
Rhodococcus sp. WAY2	*Nocardia globerula* DSM 44596	19.4	[17.2 - 21.8]	2.75

Type-based species clustering using a 70% dDDH threshold around each of the 10 type strains.

Species cluster	Name	Authority	Size (Mbp)	%GC	No. proteins	Assembly accession
1	*Rhodococcus marinonascens* NBRC 14363	Helmke and Weyland 1984	4.92	64.41	4777	GCA_001894885
2	*Rhodococcus globerulus* NBRC 14531	Goodfellow et al. 1985 emend. Nouioui et al. 2018	6.74	61.67	6262	GCA_001894805
2	*Nocardia globerula* DSM 44596	(Gray 1928) Waksman and Henrici 1948	6.48	61.72	6050	-
3	*Rhodococcus koreensis* DSM 44498	Yoon et al. 2000 emend. Nouioui et al. 2018	10.31	67.38	9444	GCA_900105905
4	*Rhodococcus jostii* DSM 44719	Takeuchi et al. 2002	9.91	66.88	9110	GCA_900105375
5	*Rhodococcus wratislaviensis* NBRC 100605	(Goodfellow et al. 1995) Goodfellow et al. 2002 emend. Nouioui et al. 2018	10.40	66.78	9472	GCA_000583735
6	*Rhodococcus erythropolis* NBRC 15567	(Gray and Thornton 1928) Goodfellow and Alderson 1979 emend. Nouioui et al. 2018	6.59	62.4	6211	GCA_001552595
7	*Rhodococcus imtechensis* RKJ300	Ghosh et al. 2006 emend. Nouioui et al. 2018	8.23	67.22	7681	GCA_000260815
7	*Rhodococcus opacus* DSM 43205	Klatte et al. 1995	8,53	67.28	7425	GCF_001646735
8	*Rhodococcus maanshanensis* DSM 44675	Zhang et al. 2002 emend. Nouioui et al. 2018	5,67	69.17	5173	GCA_900109405
9	*Rhodococcus* sp. WAY2	This work	8,44	64.47	7841	-

Supplementary File S4. Absolute values of PCB congeners degradation.

	sample	LB400.1	LB400.2	LB400.3	LB400.control1	LB400.control2	LB400.control3
**PCB congener**	**Chl.sust**	**[μg]**					
biphenyl	biphenyl	0.02	0.02	0.02	0.52	0.60	0.51
1	2-Chlorobiphenyl	0.01	0.03	0.03	0.33	0.35	0.31
2	3-Chlorobiphenyl	0.00	0.00	0.00	0.00	0.00	0.00
3	4-Chlorobiphenyl	0.00	0.00	0.00	0.15	0.15	0.14
4+10	2,2'; 2,6	0.22	0.42	0.39	3.30	3.54	3.11
5+8	2,3; 2,4'	2.02	3.39	3.01	26.74	27.67	24.76
6	2,3'	0.44	0.76	0.66	4.23	4.44	3.96
9+7	2,5; 2,4	0.06	0.08	0.07	0.51	0.52	0.48
15	4,4'	3.83	4.56	3.88	6.48	6.80	6.05
16	2,2',3	6.37	7.57	6.49	9.95	10.51	9.80
17	2,2',4	2.70	3.85	3.19	19.67	21.12	19.09
18	2,2',5	5.32	7.55	6.60	38.31	39.99	35.69
19	2,2',6	1.57	1.88	1.80	4.45	4.78	4.23
22	2,3,4'	3.18	3.75	3.49	15.43	16.40	15.21
26+25	2,3',5; 2,3',4	0.67	0.87	0.73	3.35	3.54	3.15
27+24	2,3',6; 2,3,6	0.47	0.57	0.56	2.12	2.19	2.04
28+31	2,4,4'; 2,4',5	13.14	15.66	13.73	44.09	44.65	42.06
32	2,4',6	1.11	1.53	1.35	7.56	7.87	7.00
33	2,3',4'	4.74	6.40	5.27	29.32	30.21	27.41
37	3,4,4'	11.26	12.88	11.18	11.92	12.22	11.52
40	2,2',3,3'	1.00	1.15	0.99	3.86	4.11	3.81
41+71	2,2',3,4; 2,3',4',6	1.97	2.27	1.98	3.61	3.76	3.48
42	2,2',3,4'	1.86	2.17	1.91	5.57	5.43	4.85
44	2,2',3,5'	3.90	4.55	4.07	14.49	15.43	14.34
45	2,2',3,6	2.38	2.71	2.46	4.14	4.23	3.73
46	2,2',3,6'	0.78	0.95	0.87	2.03	2.00	1.80
48+47	2,2',4,5; 2,2',4,4'	1.86	2.16	1.95	4.28	4.32	3.96
49	2,2',4,5'	3.15	3.53	3.21	10.11	10.62	9.49
51	2,2',4,6'	1.18	1.34	1.17	1.20	1.30	1.17
52	2,2',5,5'	3.29	3.80	3.36	11.07	11.59	10.66
53	2,2',5,6'	3.93	4.23	4.03	9.88	10.43	9.46
55	2,3,3',4	0.68	0.85	0.68	1.38	1.47	1.38
56+60	2,3,3',4'; 2,3,4,4'	3.59	3.90	3.47	4.33	4.43	4.16
63	2,3,4',5	0.49	0.55	0.49	0.65	0.66	0.65
66+70	2,3',4,4'; 2,3',4',5	5.26	6.08	5.22	6.57	6.77	6.51
67	2,3',4,5	0.34	0.38	0.34	0.62	0.63	0.57
72+64	2,3',5,5'; 2,3,4',6	3.44	3.95	3.43	3.94	4.20	3.82
74	2,4,4',5	5.93	6.72	5.98	6.21	6.27	5.86
76	2,3',4',5'	5.05	5.74	4.88	14.00	14.02	12.99
77	3,3',4,4'	1.07	1.27	1.09	1.01	1.05	1.03
82	2,2',3,3',4	0.68	0.74	0.63	0.76	0.78	0.83

84+92	2,2',3,3',6; 2,2',3,5,5'	0.23	0.26	0.25	0.36	0.39	0.34
87	2,2',3,4,5'	0.46	0.51	0.43	0.75	0.72	0.68
91	2,2',3,4',6	0.35	0.39	0.33	0.36	0.35	0.35
95	2,2',3,5',6	2.33	2.74	2.35	3.76	3.90	3.48
97	2,2',3,4',5'	0.50	0.57	0.49	0.62	0.65	0.62
99	2,2',4,4',5	0.76	0.83	0.77	0.90	0.96	0.88
101+113	2,2',4,5,5'; 2,3,3',5',6	0.37	0.39	0.35	0.60	0.63	0.62
105	2,3,3',4,4'	0.65	0.73	0.64	0.64	0.62	0.63
110	2,3,3',4',6	1.59	1.75	1.51	1.76	1.77	1.73
111	2,3,3',5,5'	0.55	0.62	0.52	0.54	0.55	0.55
118	2,3',4,4',5	0.87	1.02	0.87	0.86	0.90	0.88
138+163	2,2',3,4,4',5'; 2,3,3',4',5,6	0.06	0.06	0.06	0.05	0.07	0.06
148	2,2',3,4',5,6'	0.04	0.04	0.05	0.04	0.05	0.05
149	2,2',3,4',5',6	0.07	0.08	0.08	0.08	0.07	0.08
153	2,2',4,4',5,5'	0.06	0.06	0.06	0.05	0.06	0.05
180	2,2',3,4,4',5,5'	ND	ND	ND	ND	ND	ND
∑PCB	∑PCB	117.83	140.88	123.41	349.50	362.75	332.04

	sample	RHA1.1	RHA1.2	RHA1.3	RHA1.control.1	RHA1.control.2	RHA1.control.3
**PCB congener**	**Chl.sust**	**[µg]**					
biphenyl	biphenyl	0.02	0.02	0.02	0.57	0.51	0.56
1	2-Chlorobiphenyl	0.02	0.00	0.00	0.36	0.32	0.34
2	3-Chlorobiphenyl	0.00	0.00	0.00	0.00	0.00	0.00
3	4-Chlorobiphenyl	0.00	0.00	0.00	0.17	0.15	0.16
4+10	2,2'; 2,6	0.34	0.18	0.21	3.47	3.32	3.38
5+8	2,3; 2,4'	2.49	1.79	2.33	27.67	27.24	27.40
6	2,3'	0.52	0.39	0.49	4.42	4.19	4.37
9+7	2,5; 2,4	0.06	0.03	0.04	0.51	0.50	0.50
15	4,4'	3.33	4.46	3.77	6.69	6.38	6.57
16	2,2',3	3.63	5.03	4.28	10.48	10.05	10.39
17	2,2',4	3.46	4.03	4.59	20.71	20.17	19.34
18	2,2',5	6.61	7.65	8.61	40.49	38.50	39.17
19	2,2',6	2.58	3.18	2.81	4.68	4.63	4.56
22	2,3,4'	7.17	9.92	7.85	16.92	15.61	15.54
26+25	2,3',5; 2,3',4	0.97	1.28	1.24	3.69	3.32	3.38
27+24	2,3',6; 2,3,6	0.72	0.96	0.82	2.26	2.13	2.17
28+31	2,4,4'; 2,4',5	17.65	25.89	20.46	47.49	43.67	47.32
32	2,4',6	1.50	1.72	1.93	7.82	7.55	7.67
33	2,3',4'	7.68	10.00	9.73	30.76	29.48	29.88
37	3,4,4'	8.43	11.20	8.92	13.10	11.07	12.06
40	2,2',3,3'	2.54	3.37	2.71	4.29	3.79	3.95
41+71	2,2',3,4; 2,3',4',6	2.14	2.90	2.27	3.92	3.53	3.65
42	2,2',3,4'	3.09	4.17	3.54	5.52	4.71	5.25
44	2,2',3,5'	7.77	10.39	8.30	15.89	14.08	14.51

45	2,2',3,6	3.12	3.96	3.47	4.31	4.02	4.26
46	2,2',3,6'	1.48	1.92	1.63	2.14	1.91	2.04
48+47	2,2',4,5; 2,2',4,4'	2.66	3.49	2.89	5.45	3.89	4.19
49	2,2',4,5'	7.41	9.55	7.88	10.59	9.53	10.17
51	2,2',4,6'	0.97	1.19	1.01	1.29	1.22	1.20
52	2,2',5,5'	7.92	10.35	8.73	11.65	10.65	11.19
53	2,2',5,6'	7.88	9.63	8.36	10.33	9.93	10.64
55	2,3,3',4	1.12	1.43	1.07	1.53	1.19	1.29
56+60	2,3,3',4'; 2,3,4,4'	2.94	4.01	3.20	4.72	3.95	4.22
63	2,3,4',5	0.50	0.62	0.53	0.71	0.61	0.66
66+70	2,3',4,4'; 2,3',4',5	4.31	5.77	4.47	7.28	6.12	6.68
67	2,3',4,5	0.40	0.51	0.42	0.66	0.52	0.55
72+64	2,3',5,5'; 2,3,4',6	3.19	4.00	3.23	4.35	3.66	4.02
74	2,4,4',5	4.55	6.02	4.88	6.59	5.52	5.92
76	2,3',4',5'	9.34	12.51	9.96	14.90	12.46	13.52
77	3,3',4,4'	0.87	1.13	0.98	1.14	0.96	1.06
82	2,2',3,3',4	0.62	0.79	0.63	0.87	0.63	0.77
84+92	2,2',3,3',6; 2,2',3,5,5'	0.30	0.37	0.32	0.39	0.33	0.36
87	2,2',3,4,5'	0.58	0.75	0.57	0.75	0.61	0.59
91	2,2',3,4',6	0.28	0.39	0.32	0.36	0.35	0.32
95	2,2',3,5',6	3.03	3.87	3.21	4.02	3.37	3.66
97	2,2',3,4',5'	0.41	0.57	0.47	0.66	0.57	0.58
99	2,2',4,4',5	0.74	0.96	0.77	0.98	0.81	0.85
101+113	2,2',4,5,5'; 2,3,3',5',6	0.50	0.63	0.53	0.64	0.56	0.59
105	2,3,3',4,4'	0.53	0.70	0.58	0.64	0.52	0.57
110	2,3,3',4',6	1.45	1.86	1.57	1.89	1.54	1.64
111	2,3,3',5,5'	0.42	0.57	0.47	0.55	0.48	0.49
118	2,3',4,4',5	0.74	0.97	0.79	0.93	0.73	0.76
138+163	2,2',3,4,4',5'; 2,3,3',4',5,6	0.06	0.07	0.05	0.06	0.05	0.05
148	2,2',3,4',5,6'	0.04	0.04	0.04	0.05	0.05	0.04
149	2,2',3,4',5',6	0.07	0.09	0.06	0.07	0.07	0.07
153	2,2',4,4',5,5'	0.05	0.06	0.05	0.05	0.04	0.05
180	2,2',3,4,4',5,5'	ND	ND	ND	ND	ND	ND
∑PCB	∑PCB	151.20	197.35	168.05	372.41	341.79	355.10

	sample	WAY2.1	WAY2.2	WAY2.3	WAY2.control1	WAY2.control2	WAY2.control3
**PCB congener**	**Chl.sust**	**[µg]**					
biphenyl	biphenyl	0.02	0.02	0.02	0.13	0.69	0.64
1	2-Chlorobiphenyl	0.00	0.02	0.00	0.17	0.40	0.38
2	3-Chlorobiphenyl	0.00	0.00	0.00	0.00	0.00	0.00
3	4-Chlorobiphenyl	0.00	0.00	0.00	0.10	0.18	0.16
4+10	2,2'; 2,6	0.50	0.71	0.57	2.72	3.92	3.72
5+8	2,3; 2,4'	5.67	7.79	6.57	25.71	32.17	28.99
6	2,3'	1.06	1.43	1.24	4.02	5.05	4.53

9+7	2,5; 2,4	0.10	0.15	0.10	0.48	0.61	0.58
15	4,4'	2.15	3.09	2.78	6.98	7.98	7.07
16	2,2',3	9.41	11.49	11.43	10.96	13.66	11.48
17	2,2',4	7.88	10.62	9.76	20.73	24.14	21.75
18	2,2',5	13.60	17.99	16.36	41.20	50.85	44.06
19	2,2',6	4.14	4.90	4.99	4.51	5.39	4.96
22	2,3,4'	10.43	14.05	13.54	18.14	22.06	18.06
26+25	2,3',5; 2,3',4	1.55	2.13	1.99	3.87	4.35	3.88
27+24	2,3',6; 2,3,6	1.58	1.97	1.99	2.29	2.77	2.40
28+31	2,4,4'; 2,4',5	21.14	30.80	28.48	50.55	62.18	50.42
32	2,4',6	3.11	4.10	3.75	8.15	9.78	8.51
33	2,3',4'	12.63	19.46	17.16	33.17	38.37	33.95
37	3,4,4'	8.69	11.88	11.68	14.75	17.43	14.73
40	2,2',3,3'	3.83	4.59	4.59	4.63	5.44	4.73
41+71	2,2',3,4; 2,3',4',6	3.56	4.27	4.35	4.29	5.09	4.28
42	2,2',3,4'	4.80	5.49	5.75	5.99	7.51	6.16
44	2,2',3,5'	9.30	13.27	12.48	17.43	19.92	17.26
45	2,2',3,6	3.90	4.68	4.70	4.52	5.46	4.74
46	2,2',3,6'	1.79	2.24	2.32	2.16	2.62	2.32
48+47	2,2',4,5; 2,2',4,4'	3.69	4.54	4.60	5.39	5.91	4.94
49	2,2',4,5'	9.93	12.00	11.87	11.53	14.04	11.80
51	2,2',4,6'	1.16	1.42	1.43	1.38	1.55	1.39
52	2,2',5,5'	10.76	13.22	12.91	12.87	15.69	13.07
53	2,2',5,6'	9.61	11.60	11.24	11.23	13.56	11.56
55	2,3,3',4	1.41	1.84	1.77	1.65	2.06	1.82
56+60	2,3,3',4'; 2,3,4,4'	4.21	5.18	5.13	5.18	6.18	5.25
63	2,3,4',5	0.67	0.81	0.82	0.79	0.92	0.80
66+70	2,3',4,4'; 2,3',4',5	5.68	7.34	7.34	8.59	9.63	8.30
67	2,3',4,5	0.37	0.57	0.52	0.73	0.89	0.70
72+64	2,3',5,5'; 2,3,4',6	3.98	4.92	4.88	4.83	5.75	4.86
74	2,4,4',5	5.25	6.52	6.63	7.23	8.88	7.57
76	2,3',4',5'	11.99	15.44	15.16	16.75	20.00	16.73
77	3,3',4,4'	1.11	1.38	1.36	1.37	1.60	1.36
82	2,2',3,3',4	0.88	1.01	0.97	1.00	1.23	1.11
84+92	2,2',3,3',6; 2,2',3,5,5'	0.38	0.45	0.44	0.44	0.54	0.45
87	2,2',3,4,5'	0.71	0.96	0.85	0.89	0.99	0.85
91	2,2',3,4',6	0.38	0.43	0.44	0.41	0.50	0.44
95	2,2',3,5',6	3.93	4.62	4.50	4.56	5.55	4.51
97	2,2',3,4',5'	0.64	0.77	0.70	0.78	0.93	0.78
99	2,2',4,4',5	0.96	1.15	1.10	1.12	1.36	1.13
101+113	2,2',4,5,5'; 2,3,3',5',6	0.63	0.72	0.74	0.72	0.92	0.74
105	2,3,3',4,4'	0.66	0.81	0.78	0.78	0.91	0.79
110	2,3,3',4',6	1.89	2.19	2.16	2.12	2.54	2.19
111	2,3,3',5,5'	0.56	0.65	0.68	0.67	0.80	0.65
118	2,3',4,4',5	0.90	1.10	1.09	1.09	1.27	1.11
138+163	2,2',3,4,4',5'; 2,3,3',4',5,6	0.07	0.08	0.08	0.08	0.08	0.08

148	2,2',3,4',5,6'	0.04	0.05	0.05	0.05	0.06	0.06
149	2,2',3,4',5',6	0.08	0.09	0.09	0.10	0.12	0.09
153	2,2',4,4',5,5'	0.06	0.07	0.07	0.07	0.07	0.07
180	2,2',3,4,4',5,5'	ND	ND	ND	ND	ND	ND
∑PCB	∑PCB	213.44	279.06	266.98	392.04	472.57	404.97

	**sample**	**JAB1. 1**	**JAB1. 2**	**JAB1. 3**	**JAB1.control 1**	**JAB1.control 2**	**JAB1.control 3**
**PCB congener**	**Chl.sust**	**[μg]**					
biphenyl	biphenyl	0.02	0.02	0.02	0.67	0.68	0.56
1	2-Chlorobiphenyl	0.05	0.05	0.06	0.29	0.26	0.23
2	3-Chlorobiphenyl	0.00	0.00	0.00	0.00	0.00	0.00
3	4-Chlorobiphenyl	0.04	0.04	0.04	0.12	0.12	0.10
4+10	2,2'; 2,6	2.58	2.76	2.60	2.98	2.86	2.42
5+8	2,3; 2,4'	10.50	11.17	11.81	24.66	22.25	19.01
6	2,3'	1.94	2.07	2.15	3.68	3.60	3.00
9+7	2,5; 2,4	0.20	0.22	0.23	0.43	0.40	0.35
15	4,4'	3.18	3.36	3.31	5.53	5.06	4.46
16	2,2',3	7.89	8.17	7.65	8.68	8.21	7.04
17	2,2',4	15.89	17.05	16.29	18.33	16.50	14.82
18	2,2',5	29.97	33.68	31.10	34.30	31.77	26.88
19	2,2',6	3.81	4.01	3.83	4.11	3.93	3.47
22	2,3,4'	9.37	9.59	9.92	14.17	12.21	11.42
26+25	2,3',5; 2,3',4	2.12	2.20	2.25	2.97	2.72	2.45
27+24	2,3',6; 2,3,6	1.29	1.44	1.39	1.92	1.81	1.53
28+31	2,4,4'; 2,4',5	26.06	29.47	28.10	39.98	36.67	32.06
32	2,4',6	5.13	5.38	5.19	6.86	6.31	5.58
33	2,3',4'	16.98	17.48	18.23	26.66	22.42	21.96
37	3,4,4'	9.12	9.57	9.56	10.89	8.86	8.64
40	2,2',3,3'	3.21	3.18	3.12	3.84	3.29	3.17
41+71	2,2',3,4; 2,3',4',6	3.19	3.19	3.16	3.45	2.99	2.85
42	2,2',3,4'	4.23	4.95	4.93	4.71	4.63	3.88
44	2,2',3,5'	12.51	12.41	12.39	13.63	11.52	11.21
45	2,2',3,6	3.47	3.76	3.53	3.85	3.49	3.16
46	2,2',3,6'	1.61	1.68	1.64	1.84	1.56	1.50
48+47	2,2',4,5; 2,2',4,4'	4.08	3.60	3.58	4.00	3.42	3.27
49	2,2',4,5'	8.51	9.07	8.88	9.19	8.37	7.57
51	2,2',4,6'	1.02	1.11	1.09	1.14	1.02	0.95
52	2,2',5,5'	9.61	9.81	9.60	10.40	9.37	8.38
53	2,2',5,6'	8.38	8.74	8.43	9.54	8.45	7.56
55	2,3,3',4	1.16	1.07	1.13	1.39	1.08	1.12
56+60	2,3,3',4'; 2,3,4,4'	3.59	3.49	3.60	4.19	3.40	3.35
63	2,3,4',5	0.55	0.51	0.52	0.61	0.52	0.49
66+70	2,3',4,4'; 2,3',4',5	5.76	5.60	5.74	6.38	5.15	5.18
67	2,3',4,5	0.48	0.48	0.48	0.56	0.48	0.47

72+64	2,3',5,5'; 2,3,4',6	3.27	3.41	3.25	3.71	3.02	2.97
74	2,4,4',5	5.25	5.19	5.30	5.76	4.79	4.65
76	2,3',4',5'	11.79	11.62	11.75	12.66	10.72	10.59
77	3,3',4,4'	1.01	1.00	1.06	1.06	0.88	0.91
82	2,2',3,3',4	0.73	0.74	0.73	0.79	0.59	0.61
84+92	2,2',3,3',6; 2,2',3,5,5'	0.33	0.33	0.33	0.37	0.31	0.30
87	2,2',3,4,5'	0.61	0.64	0.64	0.57	0.51	0.54
91	2,2',3,4',6	0.33	0.33	0.34	0.35	0.31	0.30
95	2,2',3,5',6	3.47	3.40	3.34	3.70	2.96	2.94
97	2,2',3,4',5'	0.53	0.55	0.59	0.59	0.47	0.49
99	2,2',4,4',5	0.92	0.83	0.86	0.97	0.78	0.77
101+113	2,2',4,5,5'; 2,3,3',5',6	0.59	0.57	0.57	0.61	0.50	0.53
105	2,3,3',4,4'	0.66	0.61	0.63	0.64	0.50	0.58
110	2,3,3',4',6	1.74	1.54	1.65	1.77	1.38	1.42
111	2,3,3',5,5'	0.54	0.48	0.50	0.53	0.42	0.46
118	2,3',4,4',5	0.83	0.84	0.84	0.92	0.70	0.79
138+163	2,2',3,4,4',5'; 2,3,3',4',5,6	0.05	0.05	0.06	0.05	0.06	0.06
148	2,2',3,4',5,6'	0.04	0.04	0.04	0.05	0.04	0.05
149	2,2',3,4',5',6	0.08	0.08	0.08	0.09	0.07	0.07
153	2,2',4,4',5,5'	0.05	0.05	0.05	0.06	0.04	0.04
180	2,2',3,4,4',5,5'	ND	ND	ND	ND	ND	ND
∑PCB	∑PCB	250.32	262.68	258.15	321.17	284.42	259.15

Supplementary Table S1. *Rhodococcus* type strains 16S rRNA genes used in this study.

**Type strain name**	**Accs. no. 16S rRNA**	**Type strain name**	**Accs. no. 16S rRNA**
R. aerolatus PAMC 27367 [T]	KM044053.1	*R. koreensis* DNP505 [T]	AF124342.1
R. aetherivorans 10bc312 [T]	AF447391.1	*R. kroppenstedtii* K07-23 [T]	AY726605.1
R. agglutinans CFH S0262 [T]	KP232908.1	*R. kunmingensis* YIM 45607 [T]	DQ997045.1
R. aichiensis DSM 43978 [T]	X80633.1	*R. kyotonensis* DS472 [T]	AB269261.1
R. antrifimi D7-21 [T]	LN867321.1	*R. lactis* DW151B [T]	KP342300.1
R. artemisiae YIM 65754 [T]	GU367155.1	*R. luteus* DSM 43673 [T]	X79187.1
R. auranticus ATCC 25938 [T]	AF283282.1	*R. maanshanensis* M712 [T]	AF416566.1
R. baikonurensis GTC 1041 [T]	AB071951.1	*R. marinonascens* DSM 43752 [T]	X80617.1
R. biphenylivorans TG9 [T]	KJ546454.1	*R. maris* DSM 43672 [T]	X79290.1
R. bronchialis DSM 43247 [T]	CP001802.1	*R. nanhaiensis* SCSIO 10187 [T]	JN582175.1
R. canchipurensis MBRL 353 [T]	JN164649.1	*R. obuensis* ATCC 33610 [T] [a]	AY262329.1
R. cerastii C5 [T]	FR714842.1	*R. olei* Ktm-20 [T]	MF405107.1
R. cercidiphylli YIM 65003 [T]	EU325542.1	*R. opacus* DSM 43205 [T]	X80630.1
R. chlorophenolicum DSM 43826 [T]	X79292.1	*R. pedocola* UC12 [T]	KT301938.1
R. chubuensis DSM 44019 [T]	X80627.1	*R. percolatus* MBS1 [T]	X92114.1
R. coprophilus DSM 43347 [T]	X80626.1	*R. phenolicus* G2P [T]	AY533293.1
R. corallinus JCM 3199 [T]	AY995558.1	*R. pyridinovorans* PDB9 [T]	AF173005.1
R. corynebacterioides DSM 20151[T]	AF430066.1	*R. qingshengii* djl-6 [T]	DQ090961.1
R. defluvii Ca11 [T]	KC788572.1	*R. rhodnii* DSM 43336 [T]	X80621.1
R. degradans CCM 4446 [T]	JQ776649.2	*R. rhodochrous* DSM 43241 [T]	X79288.1
R. electrodiphilus JC435 [T]	LT630357.3	*R. roseus* ATCC 271 [T]	X81921.1
R. enclensis NIO-1009 [T]	HQ858009.1	*R. ruber* DSM 43338 [T]	X80625.1
R. equi DSM 20307 [T]	X80614.1	*R. rubripertinctus* DSM 43197 [T]	X80632.1
R. erythropolis DSM 43066 [T]	X79289.1	*R. soli* DSD51W [T]	KJ939314.1
R. fascians DSM 20669 [T]	X79186.1	*R. sovatensis* H004 [T]	KU189221.1
R. gannanensis M1 [T]	NR152643.1	*R. sputi* DSM 43896 [T]	X80634.1
R. globerulus DSM 4954 [T]	X80619.1	*R. terrae* DSM 43249 [T]	X79286.1
R. gordoniae W 4937 [T]	AY233201.1	*R. triatomae* IMMIB RIV-085 [T]	AJ854055.1
R. hoagii ATCC 7005 [T]	X82052.1	*R. trifolii* T8 [T]	FR714843.1
R. humicola UC33 [T]	KT301939.1	*R. tukisamuensis* Mb8 [T]	AB067734.1
R. imtechensis RKJ300 [T]	AY525785.2	*R. wratislaviensis* NCIMB 13082 [T]	Z37138.1
R. jialingiae djl-6-2 [T]	DQ185597.2	*R. yunnanensis* YIM 70056 [T]	AY602219.2
R. jostii NBRC 16295 [T]	AB046357.1	*R. zopfii* DSM 44108 [T]	AF191343.1

[a] *R. obuensis* ATCC 33610 [T] was excluded of further analysis because its 16S rRNA gene sequence was too small (505 nts)

Supplementary Table S2. Genomes of *Rhodococcus* type strains used in this study.

Type strain gene name	Assembly Accs. no.	Scaffolds	Size (Mbp)	GC%	Genes	Proteins
R. agglutinans CCTCC AB2014297 [T]	GCA_004011865.1	22	5.43	69.3	4.994	4.862
R. biphenylivorans TG9 [T]	GCA_003288095.1	1	5.03	68	4.7	4.373
R. bronchialis DSM 43247 [T]	GCA_000024785.1	-	5.29	67.1	4.934	4.601
R. chlorophenolicum DSM 43826 [T]	GCA_001044235.1	72	7.38	68.4	7.164	6.729
R. coprophilus NBRC 100603 [T]	GCA_001895045.1	30	4.55	66.9	4.206	4.079
R. corynebacterioides DSM 20151 [T]	GCA_001646675.1	180	3.9	70.2	3.651	3.445
R. defluvii Ca11 [T]	GCA_000738775.1	267	5.13	68.7	4.85	4.535
R. enclensis NIO-1009 [T]	GCA_900094765.1	76	7.48	62.3	7.098	6.826
R. equi DSM 20307 [T]	GCA_002094305.1	37	5.2	68.8	4.85	4.735
R. erythropolis NBRC 15567 [T]	GCA_001552595.1	67	6.59	62.4	6.147	5.981
R. fascians NBRC 12155 [T]	GCA_001894785.1	36	5.77	64.4	5.422	5.284
R. globerulus NBRC 14531 [T]	GCA_001894805.1	30	6.74	61.7	6.231	6.054
R. gordoniae DSM 44689 [T]	GCA_001646655.1	114	4.82	67.9	4.46	4.238
R. hoagii DSM 20295 [T]	GCA_001646645.1	279	4.97	68.8	4.757	4.427
R. imtechensis RKJ300 [T]	GCA_000260815.1	178	8.23	67.2	7.962	7.245
R. jostii NBRC 16295 [T]	GCA_001894825.1	286	9.73	66.9	8.983	8.358
R. koreensis DSM 44498 [T]	GCA_900105905.1	9	10.31	67.4	9.491	8.902
R. kroppenstedtii DSM 44908 [T]	GCA_900111805.1	30	4.08	70.1	3.789	3.652
R. kunmingensis DSM 45001 [T]	GCA_001646865.1	330	5.62	66.2	3.76	4.885
R. kyotonensis JCM 23211 [T]	GCA_900188125.1	50	6.31	64.2	5.86	5.698
R. maanshanensis DSM 44675 [T]	GCA_900109405.1	61	5.67	69.2	5.143	4.972
R. marinonascens NBRC 14363 [T]	GCA_001894885.1	156	4.92	64.4	3.28	4.269
R. maris DSM 43672 [T]	GCA_001630765.1	57	3.51	70.9	3.302	3.098
R. opacus DSM 43205 [T]	GCA_001646735.1	382	8.53	67.3	8.198	7.418
R. phenolicus DSM 44812 [T]	GCA_001646785.1	232	6.28	68.4	5.916	5.6
R. pyridinivorans DSM 44555 [T]	GCA_900105195.1	3	5.26	67.8	4.864	4.604
R. qingshengii JCM 15477 [T]	GCA_001646745.1	131	7.26	62.4	6.82	6.574
R. rhodnii NBRC 100604 [T]	GCA_001894925.1	70	4.46	69.7	4.265	4.08
R. rhodochrous DSM 43241 [T]	GCA_001646825.1	105	5.18	68.2	4.79	4.585
R. ruber DSM 43338 [T]	GCA_001646835.1	164	5.3	70.7	4.93	4.677
R. rubripertincta NBRC 101908 [T]	GCA_000327325.1	134	5.2	67.4	4.792	4.571
R. sputi NBRC 100414 [T]	GCA_000248055.2	158	4.95	65.4	4.642	4.592
R. terrae NRRL B-16283 [T]	GCA_003183825.1	-	5.71	67.8	5.109	4.951
R. triatomae DSM 44892 [T]	GCA_900099725.1	38	4.73	68.7	4.43	4.297
R. tukisamuensis NBRC 100609 [T]	GCA_001894985.1	66	5.49	69.9	4.97	4.779
R. wratislaviensis NBRC 100605[T]	GCA_000583735.1	151	10.4	66.8	9.514	8.883
R. yunnanensis NBRC 103083 [T]	GCA_001895005.1	68	6.37	63.9	5.847	5.65
R. zopfii NBRC 100606 [T]	GCA_001895025.1	146	6.3	68.2	5.848	5.575

Table S3. Genes involved in core cellular functions of *Rhodococcus* sp. WAY2

Replicon	Start	End	Strand	Gene name	Predicted function	Cellular function
Chromosome	1	1,587	+	*dnaA*	Chromosomal replication initiator protein	Replication
Chromosome	3,656,174	3,658,378	+	*dnaB*	Replicative DNA helicase	Replication
Chromosome	2602811	2604742	+	*dnaG*	DNA primase	Replication
Chromosome	2,799,978	2,803,514	+	*dnaE*	DNA polymerase III alpha subunit	Replication
Chromosome	2,247	3,431	+	*dnaN*	DNA polymerase III beta subunit	Replication
Chromosome	2,737,654	2,735,894	-	*dnaQ*	DNA polymerase III epsilon subunit	Replication
Chromosome	595,751	598,042	+	*dnaX*	DNA polymerase III gamma and tau subunits	Replication
Chromosome	2,552,031	2,553,008	+	*holA*	DNA polymerase III delta subunit	Replication
Chromosome	719,116	720,330	+	*holB*	DNA polymerase III delta prime subunit	Replication
Chromosome	17,206	19,728	+	*gyrA*	DNA gyrase subunit A	Replication
Chromosome	9,531	11,573	+	*gyrB*	DNA gyrase subunit B	Replication
Chromosome	4,508,290	4,509,321	+	*ligC*	ATP-dependent DNA ligase	Replication
Chromosome	716,139	719,063	+	*topA*	DNA topoisomerase I	Replication
Chromosome	2452962	2453426	+	*ssb*	Single-strand DNA-binding protein	Replication
Chromosome	3,654,343	3,654,864	+	*ssb*	Single-strand DNA-binding protein	Replication
Chromosome	3,496,401	3,494,383	-	*priA*	Primosomal protein n’	Replication
Chromosome	2,947,050	2,947,949	+	*parA1*	Chromosome (plasmid) partitioning protein	Replication
Chromosome	4,751,853	4,752,611	+	*parA2*	Chromosome (plasmid) partitioning protein	Replication
Chromosome	5,976,007	5,976,804	+	*parA3*	Chromosome (plasmid) partitioning protein	Replication
Chromosome	6,616,198	6,615,185	-	*parA4*	Chromosome (plasmid) partitioning protein	Replication
Chromosome	6,615,188	6,614,127	-	*parB*	Chromosome (plasmid) partitioning protein	Replication
pRWAY01	382,849	383,562	+	*parA*	Plasmid partitioning protein	Replication
pRWAY01	383,562	384,428	+	*parB1*	Chromosome (plasmid) partitioning protein	Replication
pRWAY01	446,841	445,951	-	*parB2*	Chromosome (plasmid) partitioning protein	Replication
pRWAY01	470,162	468,558	-	*parB3*	Chromosome (plasmid) partitioning protein	Replication
pRWAY02	86,990	87,772	+	*parA*	Chromosome (plasmid) partitioning protein	Replication
pRWAY03	339,700	340,668	+	*parA*	Chromosome (plasmid) partitioning protein	Replication
pRWAY04	5,889	5,116	-	*parA*	Chromosome (plasmid) partitioning protein	Replication
pRWAY01	106,121	104,127	-	*tap1*	Telomere-binding protein	Telomere stabilization
pRWAY01	572,964	571,006	-	*tap2*	Telomere-binding protein	Telomere stabilization
pRWAY01	734,553	733,756	-	*tpg*	Telomere terminal protein	Telomere stabilization
pRWAY01	736,526	734,553	-	*tap3*	Telomere-binding protein	Telomere stabilization

pRWAY02	105,788	107,872	+	*tap1*	Telomere-binding protein	Telomere stabilization
pRWAY02	107,872	108,669	+	*tpg*	Telomere terminal protein	Telomere stabilization
pRWAY02	164,253	162,955	-	*tap2*	Telomere-binding protein	Telomere stabilization
pRWAY02	454,926	458,255	+	*tap3*	Telomere-binding protein	Telomere stabilization
pRWAY02	460,071	458,329	-	*tap4*	Telomere-binding protein	Telomere stabilization
pRWAY03	241,118	239,262	-	*tap1*	Telomere-binding protein	Telomere stabilization
pRWAY03	342,324	347,702	+	*tap2*	Telomere-binding protein	Telomere stabilization
pRWAY03	347,699	348,433	+	*tpg*	Telomere terminal protein	Telomere stabilization
Chromosome	5,771,254	5,772,315	+	*rpoA*	DNA-directed RNA polymerase alpha subunit	Transcription
Chromosome	1,814,549	1,818,037	+	*rpoB*	DNA-directed RNA polymerase beta subtunit	Transcription
Chromosome	1,818,157	1,822,113	+	*rpoC*	DNA-directed RNA polymerase beta’ subunit	Transcription
Chromosome	6,343,022	6,341,646	-	*rpoD*	RNA polymerase sigma factor RpoD	Trnascription
Chromosome	4,194,213	4,194,719	+	*rpoN*	RNA polymerase sigma-54 factor RpoN	Transcription
Chromosome	3,499,415	3,499,110	-	*rpoZ*	DNA-directed RNA polymerase omega subunit	Transcription
Chromosome	1,089,695	1,088,799	-	*sigF1*	RNA polymerase sigma-70 factor	Transcription
Chromosome	1,411,251	1,410,331	-	*sigJ1*	RNA polymerase sigma-70 factor	Transcription
Chromosome	1,685,881	1,685,144	-	*sigF2*	RNA polymerase sigma-70 factor	Transcription
Chromosome	3,209,743	3,210,678	+	*sigX*	RNA polymerase sigma-70 factor	Transcription
Chromosome	3,304,884	3,305,681	+	*sigF3*	RNA polymerase sigma-70 factor	Transcription
Chromosome	3,811,818	3,812,057	+	*sig*	RNA polymerase sigma-70 factor	Transcription
Chromosome	3,927,675	3,926,941	-	*sigK1*	RNA polymerase sigma-70 factor	Transcription
Chromosome	4,125,707	4,125,036	-	*sigD1*	RNA polymerase sigma-70 factor	Transcription
Chromosome	4,600,503	4,600,982	+	*sigD2*	RNA polymerase sigma-70 factor	Transcription
Chromosome	4,890,674	4,891,333	+	*sigZ*	RNA polymerase sigma-70 factor	Transcription
Chromosome	4,913,325	4,912,345	-	*sig*	RNA polymerase sigma-70 factor	Transcription
Chromosome	5,229,528	5,228,305	-	*sig*	RNA polymerase sigma-70 factor	Transcription
Chromosome	5,554,709	5,555,359	+	*sigE*	RNA polymerase sigma-70 factor	Transcription
Chromosome	5,814,072	5,813,404	-	*sigK2*	RNA polymerase sigma-70 factor	Transcription
Chromosome	5,817,100	5,817,447	+	*sigD3*	RNA polymerase sigma-70 factor	Transcription
Chromosome	5,969,836	5,970,573	+	*sigH*	RNA polymerase sigma-70 factor	Transcription
Chromosome	6,332,642	6,331,671	-	*sigB*	RNA polymerase sigma-70 factor	Transcription
Chromosome	6,606,184	6,606,765	+	*sigM*	RNA polymerase sigma-70 factor	Transcription
Chromosome	3,211,552	3,211,214	-	*rbpA1*	RNA polymerase-binding protein	Transcription
Chromosome	721,940	723,459	+	*16S rRNA*	Small Subunit Ribosomal RNA	Translation
Chromosome	731,952	733,471	+	*16S rRNA*	Small Subunit Ribosomal RNA	Translation
Chromosome	2,377,970	2,379,489	+	*16S rRNA*	Small Subunit Ribosomal RNA	Translation
Chromosome	2,929,964	2,931,483	+	*16S rRNA*	Small Subunit Ribosomal RNA	Translation
Chromosome	723,795	726,930	+	*23S rRNA*	Large Subunit Ribosomal RNA	Translation

Chromosome	733,805	736,936	+	*23S rRNA*	Large Subunit Ribosomal RNA	Translation
Chromosome	2,379,825	2,382,960	+	*23S rRNA*	Large Subunit Ribosomal RNA	Translation
Chromosome	2,931,819	2,934,954	+	*23S rRNA*	Large Subunit Ribosomal RNA	Translation
Chromosome	727,095	727,215	+	*5S rRNA*	5S RNA	Translation
Chromosome	737,090	737,210	+	*5S rRNA*	5S RNA	Translation
Chromosome	2,383,114	2,383,234	+	*5S rRNA*	5S RNA	Translation
Chromosome	2,935,107	2,935,227	+	*5S rRNA*	5S RNA	Translation
Chromosome	2,890,126	2,891,613	+	*rpsA*	30S ribosomal protein S1	Translation
Chromosome	6,183,560	6,184,408	+	*rpsB*	30S ribosomal protein S2	Translation
Chromosome	5,759,342	5,760,148	+	*rpsC*	30S ribosomal protein S3	Translation
Chromosome	5,770,532	5,771,137	+	*rpsD*	30S ribosomal protein S4	Translation
Chromosome	5,764,275	5,764,931	+	*rpsE*	30S ribosomal protein S5	Translation
Chromosome	3,653,990	3,654,277	+	*rpsF*	30S ribosomal protein S6	Translation
Chromosome	1,856,786	1,857,256	+	*rpsG*	30S ribosomal protein S7	Translation
Chromosome	5,762,858	5,763,280	+	*rpsH*	30S ribosomal protein S8	Translation
Chromosome	5,792,720	5,793,256	+	*rpsI*	30S ribosomal protein S9	Translation
Chromosome	5,755,800	5,756,105	+	*rpsJ*	30S ribosomal protein S10	Translation
Chromosome	5,770,096	5,770,509	+	*rpsK*	30S ribosomal protein S11	Translation
Chromosome	1,856,412	1,856,786	+	*rpsL*	30S ribosomal protein S12	Translation
Chromosome	5,769,764	5,770,096	+	*rpsM*	30S ribosomal protein S13	Translation
Chromosome	5,201,297	5,200,992	-	*rpsN*	30S ribosomal protein S14	Translation
Chromosome	6,248,056	6,248,325	+	*rpsO*	30S ribosomal protein S15	Translation
Chromosome	6,165,774	6,166,235	+	*rpsP*	30S ribosomal protein S16	Translation
Chromosome	5,760,801	5,761,082	+	*rpSQ*	30S ribosomal protein S17	Translation
Chromosome	3,654,918	3,655,145	+	*rpsR1*	30S ribosomal protein S18	Translation
Chromosome	5,200,982	5,200,728	-	*rpsR2*	30S ribosomal protein S18	Translation
Chromosome	5,758,660	5,758,941	+	*rpsS*	30S ribosomal protein S19	Translation
Chromosome	2,553,338	2,553,078	-	*rpsT*	30S ribosomal protein S20	Translation
Chromosome	5,762,543	5,762,728	+	*rpsZ*	30S ribosomal protein S14 type Z	Translation
Chromosome	1,797,555	1,798,271	+	*rplA*	50S ribosomal protein L1	Translation
Chromosome	5,757,810	5,758,646	+	*rplB*	50S ribosomal protein L2	Translation
Chromosome	5,756,126	5,756,782	+	*rplC*	50S ribosomal protein L3	Translation
Chromosome	5,756,779	5,757,468	+	*rplD*	50S ribosomal protein L4	Translation
Chromosome	5,761,972	5,762,538	+	*rplE*	50S ribosomal protein L5	Translation
Chromosome	5,763,295	5,763,834	+	*rplF*	50S ribosomal protein L6	Translation
Chromosome	1,799,337	1,799,726	+	*rplL*	50S ribosomal protein L7/L12	Translation
Chromosome	3,655,163	3,655,618	+	*rplI*	50S ribosomal protein L9	Translation
Chromosome	1,798,701	1,799,258	+	*rplJ*	50S ribosomal protein L10	Translation

Chromosome	1,796,992	1,797,426	+	*rplK*	50S ribosomal protein L11	Translation
Chromosome	5,792,280	5,792,723	+	*rplM*	50S ribosomal protein L13	Translation
Chromosome	5,761,285	5,761,653	+	*rplN*	50S ribosomal protein L14	Translation
Chromosome	5,765,113	5,765,556	+	*rplO*	50S ribosomal protein L15	Translation
Chromosome	5,760,152	5,760,568	+	*rplP*	50S ribosomal protein L16	Translation
Chromosome	5,772,346	5,772,921	+	*rplQ*	50S ribosomal protein L17	Translation
Chromosome	5,763,836	5,764,243	+	*rplR*	50S ribosomal protein L18	Translation
Chromosome	6,171,274	6,171,615	+	*rplS*	50S ribosomal protein L19	Translation
Chromosome	2,907,916	2,908,305	+	*rplT*	50S ribosomal protein L20	Translation
Chromosome	2,523,861	2,524,172	+	*rplU*	50S ribosomal protein L21	Translation
Chromosome	5,758,938	5,759,342	+	*rplV*	50S ribosomal protein L22	Translation
Chromosome	5,757,465	5,757,770	+	*rplW*	50S ribosomal protein L23	Translation
Chromosome	5,761,653	5,761,970	+	*rplX*	50S ribosomal protein L24	Translation
Chromosome	5,275,581	5,274,967	-	*rplY*	50S ribosomal protein L25	Translation
Chromosome	2,524,282	2,524,479	+	*rpmA*	50S ribosomal protein L27	Translation
Chromosome	5,201,715	5,201,473	-	*rpmB*	50S ribosomal protein L28	Translation
Chromosome	5,760,568	5,760,804	+	*rpmC*	50S ribosomal protein L29	Translation
Chromosome	5,764,931	5,765,110	+	*rpmD*	50S ribosomal protein L30	Translation
Chromosome	2,362,720	2,362,962	+	*rpmE1*	50S ribosomal protein L31	Translation
Chromosome	5,203,078	5,203,332	+	*rpmE2*	50S ribosomal protein L31	Translation
Chromosome	5,203,394	5,203,567	+	*rpmF1*	50S ribosomal protein L32	Translation
Chromosome	6,149,124	6,149,237	+	*rpmF2*	50S ribosomal protein L32	Translation
Chromosome	1,794,071	1,794,238	+	*rpmG1*	50S ribosomal protein L33	Translation
Chromosome	5,201,473	5,201,309	-	*rpmG2*	50S ribosomal protein L33	Translation
Chromosome	6,621,327	6,621,184	-	*rpmH*	50S ribosomal protein L34	Translation
Chromosome	2,907,657	2,907,851	+	*rpmI*	50S ribosomal protein L35	Translation
Chromosome	5,769,373	5,769,486	+	*rpmJ*	50S ribosomal protein L36	Translation
Chromosome	5,218,862	5,218,937	+	$tRNA^{Ala}CGC$	tRNA-Ala-CGC	Translation
Chromosome	6,044,028	6,044,103	+	$tRNA^{Ala}GGC$	tRNA-Ala-GGC	Translation
Chromosome	21,380	21,452	+	$tRNA^{Ala}TGC$	tRNA-Ala-TGC	Translation
Chromosome	542,988	543,063	+	$tRNA^{Arg}ACG$	tRNA-Arg-ACG	Translation
Chromosome	2,349,107	2,349,032	-	$tRNA^{Arg}CCG$	tRNA-Arg-CCG	Translation
Chromosome	1,525,339	1,525,267	-	$tRNA^{Arg}CCT$	tRNA-Arg-CCT	Translation
Chromosome	2,450,226	2,450,154	-	$tRNA^{Arg}TCT$	tRNA-Arg-TCT	Translation
Chromosome	2,605,142	2,605,217	+	$tRNA^{Asn}GTT$	tRNA-Asn-GTT	Translation
Chromosome	1,312,751	1,312,824	+	$tRNA^{Asp}GTC$	tRNA-Asp-GTC	Translation
Chromosome	2,319,606	2,319,677	+	$tRNA^{Cys}GCA$	tRNA-Cys-GCA	Translation
Chromosome	6,392,827	6,392,757	-	$tRNA^{Cys}GCA$	tRNA-Cys-GCA	Translation

Chromosome	6,121,902	6,121,973	+	tRNA^{Gln}CTG	tRNA-Gln-CTG	Translation
Chromosome	5,331,738	5,331,665	-	tRNA^{Gln}TTG	tRNA-Gln-TTG	Translation
Chromosome	6,122,081	6,122,153	+	tRNA^{Glu}CTC	tRNA-Glu-CTC	Translation
Chromosome	1,312,567	1,312,639	+	tRNA^{Glu}TTC	tRNA-Glu-TTC	Translation
Chromosome	4,847,349	4,847,276	-	tRNA^{Gly}CCC	tRNA-Gly-CCC	Translation
pRWAY01	824,552	824,479	-	tRNA^{Gly}CCC	tRNA-Gly-CCC	Translation
Chromosome	6,392,612	6,392,537	-	tRNA^{Gly}GCC	tRNA-Gly-GCC	Translation
Chromosome	6,392,924	6,392,849	-	tRNA^{Gly}GCC	tRNA-Gly-GCC	Translation
Chromosome	2,476,000	2,475,927	-	tRNA^{Gly}TCC	tRNA-Gly-TCC	Translation
Chromosome	2,435,266	2,435,191	-	tRNA^{His}GTG	tRNA-His-GTG	Translation
Chromosome	21,186	21,259	+	tRNA^{Ile}GAT	tRNA-Ile-GAT	Translation
Chromosome	2,871,855	2,871,782	-	tRNA^{Leu}CAA	tRNA-Leu-CAA	Translation
Chromosome	43,364	43,449	+	tRNA^{Leu}CAG	tRNA-Leu-CAG	Translation
Chromosome	2,976,567	2,976,482	-	tRNA^{Leu}GAG	tRNA-Leu-GAG	Translation
Chromosome	5,348,842	5,348,915	+	tRNA^{Leu}TAA	tRNA-Leu-TAA	Translation
Chromosome	2,420,124	2,420,040	-	tRNA^{Leu}TAG	tRNA-Leu-TAG	Translation
Chromosome	2,433,614	2,433,542	-	tRNA^{Lys}CTT	tRNA-Lys-CTT	Translation
Chromosome	1,311,494	1,311,419	-	tRNA^{Lys}TTT	tRNA-Lys-TTT	Translation
Chromosome	1,793,937	1,794,009	+	tRNA^{Met}CAT	tRNA-Met-CAT	Translation
Chromosome	2,606,972	2,607,048	+	tRNA^{Met}CAT	tRNA-Met-CAT	Translation
Chromosome	3,564,444	3,564,368	-	tRNA^{Met}CAT	tRNA-Met-CAT	Translation
Chromosome	4,174,406	4,174,330	-	tRNA^{Met}CAT	tRNA-Met-CAT	Translation
Chromosome	6,026,484	6,026,560	+	tRNA^{Met}CAT	tRNA-Met-CAT	Translation
Chromosome	1,312,891	1,312,964	+	tRNA^{Phe}GAA	tRNA-Phe-GAA	Translation
Chromosome	681,526	681,453	-	tRNA^{Pro}CGG	tRNA-Pro-CGG	Translation
Chromosome	3,571,928	3,571,852	-	tRNA^{Pro}CGG	tRNA-Pro-CGG	Translation
Chromosome	2,952,883	2,952,959	+	tRNA^{Pro}GGG	tRNA-Pro-GGG	Translation
Chromosome	2,476,140	2,476,216	+	tRNA^{Pro}TGG	tRNA-Pro-TGG	Translation
Chromosome	556,756	556,846	+	tRNA^{Ser}CGA	tRNA-Ser-CGA	Translation
Chromosome	542,855	542,946	+	tRNA^{Ser}GCT	tRNA-Ser-GCT	Translation
Chromosome	585,846	585,759	-	tRNA^{Ser}GGA	tRNA-Ser-GGA	Translation
Chromosome	533,689	533,775	+	tRNA^{Ser}TGA	tRNA-Ser-TGA	Translation
Chromosome	720,362	720,434	+	tRNA^{Thr}CGT	tRNA-Thr-CGT	Translation
Chromosome	1,793,819	1,793,891	+	tRNA^{Thr}GGT	tRNA-Thr-GGT	Translation
Chromosome	1,088,492	1,088,566	+	tRNA^{Thr}TGT	tRNA-Thr-TGT	Translation
Chromosome	1,795,487	1,795,562	+	tRNA^{Trp}CCA	tRNA-Trp-CCA	Translation
Chromosome	1,793,533	1,793,615	+	tRNA^{Tyr}GTA	tRNA-Tyr-GTA	Translation
Chromosome	6,393,156	6,393,230	+	tRNA^{Val}CAC	tRNA-Val-CAC	Translation

Chromosome	6,392,521	6,392,447	-	$tRNA^{Val}GAC$	tRNA-Val-GAC	Translation
Chromosome	6,392,755	6,392,681	-	$tRNA^{Val}GAC$	tRNA-Val-GAC	Translation
Chromosome	2,672,752	2,672,826	+	$tRNA^{Val}TAC$	tRNA-Val-TAC	Translation
Chromosome	768,635	770,899	+	*ftsH1*	Cell division protein FtsH	Cell division
Chromosome	1,196,871	1,197,113	+	*ftsH2*	Cell division protein FtsH	Cell division
Chromosome	1,197,110	1,198,015	+	*ftsH3*	Cell division protein FtsH	Cell division
Chromosome	2,476,278	2,477,654	+	*tig*	Cell division trigger factor	Cell division
Chromosome	2,708,947	2,708,057	-	*yfhF1*	Cell division inhibitor	Cell division
Chromosome	3,930,915	3,932,267	+	*ytfH2*	Cell division inhibitor	Cell division
Chromosome	2,768,737	2,769,168	+	*mraZ*	Cell division protein MraZ	Cell division
Chromosome	2,770,506	2,771,060	+	*ftsL*	Cell division protein FtsL	Cell division
Chromosome	2,771,147	2,772,907	+	*ftsI*	Cell division protein FtsI	Cell division
Chromosome	2,779,155	2,780,501	+	*ftsW*	Cell division protein FtsW	Cell division
Chromosome	2,783,343	2,784,026	+	*ftsQ*	Cell division protein FtsQ	Cell division
Chromosome	2,784,237	2,785,433	+	*ftsZ*	Cell division protein FtsZ	Cell division
Chromosome	6,035,272	6,035,961	+	*ftsE*	Cell division transporter, ATP-binding protein FtsE	Cell division
Chromosome	6,036,025	6,036,930	+	*ftsX*	Cell division protein FtsX	Cell division
Chromosome	6,157,007	6,158,422	+	*ftsY*	Signal recognition particle receptor protein FtsY	Cell division
Chromosome	6,263,572	6,266,208	+	*ftsK*	Cell division protein FtsK	Cell division

Supplementary Table S4. GGDC comparisons of *Rhodococcus* type strains sequenced genomes.

Query	Subject	DDH	Model C.I.	Distance	Prob. DDH >= 70%	G+C difference
000_Nocardia_brasiliensis_NCTC_11294_T	001_Rhodococcus_sp._WAY2	20.50	[18.3 - 22.9%]	0.21410	0.000	2.570
000_Nocardia_brasiliensis_NCTC_11294_T	002_Rhodococcus_agglutinans_CCTCC_AB2014297_T	20.50	[18.3 - 23%]	0.21400	0.000	1.010
000_Nocardia_brasiliensis_NCTC_11294_T	003_Rhodococcus_biphenylivorans_TG9_T	20.20	[18 - 22.6%]	0.21730	0.000	0.190
000_Nocardia_brasiliensis_NCTC_11294_T	004_Rhodococcus_bronchialis_DSM_43247_T	19.80	[17.6 - 22.2%]	0.22240	0.000	1.200
000_Nocardia_brasiliensis_NCTC_11294_T	005_Rhodococcus_chlorophenolicum_DSM_43826_T	19.70	[17.5 - 22.1%]	0.22310	0.000	0.190
000_Nocardia_brasiliensis_NCTC_11294_T	006_Rhodococcus_coprophilus_NBRC_100603_T	20.50	[18.3 - 22.9%]	0.21440	0.000	1.360
000_Nocardia_brasiliensis_NCTC_11294_T	007_Rhodococcus_corynebacterioides_DSM_20151_T	20.80	[18.6 - 23.2%]	0.21110	0.000	2.010
000_Nocardia_brasiliensis_NCTC_11294_T	008_Rhodococcus_defluvii_Ca11_T	20.40	[18.1 - 22.8%]	0.21590	0.000	0.480
000_Nocardia_brasiliensis_NCTC_11294_T	009_Rhodococcus_enclensis_NIO_1009_T	20.30	[18 - 22.7%]	0.21690	0.000	5.930
000_Nocardia_brasiliensis_NCTC_11294_T	010_Rhodococcus_equi_DSM_20307_T	20.60	[18.3 - 23%]	0.21360	0.000	0.550
000_Nocardia_brasiliensis_NCTC_11294_T	011_Rhodococcus_erythropolis_NBRC_15567_T	20.20	[18 - 22.7%]	0.21710	0.000	5.840
000_Nocardia_brasiliensis_NCTC_11294_T	012_Rhodococcus_fascians_NBRC_12155_T	20.40	[18.2 - 22.8%]	0.21510	0.000	3.790
000_Nocardia_brasiliensis_NCTC_11294_T	013_Rhodococcus_globerulus_NBRC_14531_T	20.60	[18.4 - 23%]	0.21310	0.000	6.570
000_Nocardia_brasiliensis_NCTC_11294_T	014_Rhodococcus_gordoniae_DSM_44689_T	20.00	[17.8 - 22.4%]	0.21930	0.000	0.290
000_Nocardia_brasiliensis_NCTC_11294_T	015_Rhodococcus_hoagii_DSM_20295_T	20.70	[18.4 - 23.1%]	0.21250	0.000	0.530
000_Nocardia_brasiliensis_NCTC_11294_T	016_Rhodococcus_imtechensis_JCM_13270_T	20.20	[17.9 - 22.6%]	0.21800	0.000	1.020
000_Nocardia_brasiliensis_NCTC_11294_T	017_Rhodococcus_jostii_NBRC_16295_T	20.30	[18.1 - 22.8%]	0.21600	0.000	1.340
000_Nocardia_brasiliensis_NCTC_11294_T	018_Rhodococcus_koreensis_DSM_44498_T	20.10	[17.9 - 22.5%]	0.21890	0.000	0.860
000_Nocardia_brasiliensis_NCTC_11294_T	019_Rhodococcus_kroppenstedtii_DSM_44908_T	21.20	[19 - 23.7%]	0.20680	0.000	1.830
000_Nocardia_brasiliensis_NCTC_11294_T	020_Rhodococcus_kunmingensis_DSM_45001_T	20.70	[18.5 - 23.2%]	0.21190	0.000	2.040
000_Nocardia_brasiliensis_NCTC_11294_T	021_Rhodococcus_kyotonensis_JCM_23211_T	19.80	[17.6 - 22.2%]	0.22200	0.000	4.080
000_Nocardia_brasiliensis_NCTC_11294_T	022_Rhodococcus_maanshanensis_DSM_44675_T	20.20	[17.9 - 22.6%]	0.21800	0.000	0.930
000_Nocardia_brasiliensis_NCTC_11294_T	023_Rhodococcus_marinonascens_NBRC_14363_T	20.50	[18.2 - 22.9%]	0.21470	0.000	3.830
000_Nocardia_brasiliensis_NCTC_11294_T	024_Rhodococcus_maris_DSM_43672_T	20.50	[18.3 - 22.9%]	0.21450	0.000	2.620
000_Nocardia_brasiliensis_NCTC_11294_T	025_Rhodococcus_opacus_DSM_43205_T	20.10	[17.9 - 22.6%]	0.21810	0.000	0.960
000_Nocardia_brasiliensis_NCTC_11294_T	026_Rhodococcus_phenolicus_DSM_44812_T	20.30	[18 - 22.7%]	0.21690	0.000	0.120

000_Nocardia_brasiliensis_NCTC_11294_T	027_Rhodococcus_pyridinivorans_DSM_44555_T	19.90	[17.7 - 22.3%]	0.22040	0.000	0.390
000_Nocardia_brasiliensis_NCTC_11294_T	028_Rhodococcus_qingshengii_JCM_15477_T	20.40	[18.2 - 22.8%]	0.21520	0.000	5.870
000_Nocardia_brasiliensis_NCTC_11294_T	029_Rhodococcus_rhodnii_NBRC_100604_T	21.20	[19 - 23.6%]	0.20690	0.000	1.430
000_Nocardia_brasiliensis_NCTC_11294_T	030_Rhodococcus_rhodochrous_DSM_43241_T	20.60	[18.4 - 23%]	0.21300	0.000	0.010
000_Nocardia_brasiliensis_NCTC_11294_T	031_Rhodococcus_ruber_DSM_43338_T	20.50	[18.3 - 23%]	0.21390	0.000	2.430
000_Nocardia_brasiliensis_NCTC_11294_T	032_Rhodococcus_rubripertincta_NBRC_101908_T	20.20	[18 - 22.6%]	0.21760	0.000	0.860
000_Nocardia_brasiliensis_NCTC_11294_T	033_Rhodococcus_sputi_NBRC_100414_T	20.30	[18.1 - 22.7%]	0.21680	0.000	2.890
000_Nocardia_brasiliensis_NCTC_11294_T	034_Rhodococcus_terrae_NRRL_B_16283_T	20.30	[18.1 - 22.7%]	0.21650	0.000	0.430
000_Nocardia_brasiliensis_NCTC_11294_T	035_Rhodococcus_triatomae_DSM_44892_T	20.60	[18.4 - 23%]	0.21330	0.000	0.490
000_Nocardia_brasiliensis_NCTC_11294_T	036_Rhodococcus_tukisamuensis_NBRC_100609_T	20.50	[18.3 - 22.9%]	0.21450	0.000	1.610
000_Nocardia_brasiliensis_NCTC_11294_T	037_Rhodococcus_wratislaviensis_NBRC_100605_T	20.10	[17.9 - 22.5%]	0.21880	0.000	1.460
000_Nocardia_brasiliensis_NCTC_11294_T	038_Rhodococcus_yunnanensis_NBRC_103083_T	20.10	[17.9 - 22.6%]	0.21820	0.000	4.340
000_Nocardia_brasiliensis_NCTC_11294_T	039_Rhodococcus_zopfii_NBRC_100606_T	19.80	[17.6 - 22.2%]	0.22200	0.000	0.000
001_Rhodococcus_sp._WAY2	002_Rhodococcus_agglutinans_CCTCC_AB2014297_T	19.90	[17.7 - 22.3%]	0.22070	0	3.58
001_Rhodococcus_sp._WAY2	003_Rhodococcus_biphenylivorans_TG9_T	20.30	[18 - 22.7%]	0.21690	0	2.38
001_Rhodococcus_sp._WAY2	004_Rhodococcus_bronchialis_DSM_43247_T	20.70	[18.5 - 23.2%]	0.21180	0	1.37
001_Rhodococcus_sp._WAY2	005_Rhodococcus_chlorophenolicum_DSM_43826_T	19.70	[17.5 - 22.1%]	0.22320	0	2.76
001_Rhodococcus_sp._WAY2	006_Rhodococcus_coprophilus_NBRC_100603_T	19.80	[17.6 - 22.2%]	0.22230	0	1.21
001_Rhodococcus_sp._WAY2	007_Rhodococcus_corynebacterioides_DSM_20151_T	20.30	[18.1 - 22.8%]	0.21590	0	4.58
001_Rhodococcus_sp._WAY2	008_Rhodococcus_defluvii_Ca11_T	20.10	[17.9 - 22.6%]	0.21820	0	3.05
001_Rhodococcus_sp._WAY2	009_Rhodococcus_enclensis_NIO_1009_T	19.90	[17.7 - 22.3%]	0.22080	0	3.36
001_Rhodococcus_sp._WAY2	010_Rhodococcus_equi_DSM_20307_T	20.10	[17.8 - 22.5%]	0.21910	0	3.12
001_Rhodococcus_sp._WAY2	011_Rhodococcus_erythropolis_NBRC_15567_T	20.10	[17.9 - 22.5%]	0.21860	0	3.27
001_Rhodococcus_sp._WAY2	012_Rhodococcus_fascians_NBRC_12155_T	19.50	[17.3 - 21.9%]	0.22520	0	1.22
001_Rhodococcus_sp._WAY2	013_Rhodococcus_globerulus_NBRC_14531_T	19.40	[17.2 - 21.8%]	0.22690	0	4
001_Rhodococcus_sp._WAY2	014_Rhodococcus_gordoniae_DSM_44689_T	19.80	[17.6 - 22.2%]	0.22200	0	2.28
001_Rhodococcus_sp._WAY2	015_Rhodococcus_hoagii_DSM_20295_T	20.30	[18 - 22.7%]	0.21690	0	3.1
001_Rhodococcus_sp._WAY2	016_Rhodococcus_imtechensis_JCM_13270_T	27.60	[25.2 - 30.1%]	0.15630	0.04	1.55
001_Rhodococcus_sp._WAY2	017_Rhodococcus_jostii_NBRC_16295_T	26.70	[24.3 - 29.1%]	0.16240	0.02	1.23

001_Rhodococcus_sp._WAY2	018_Rhodococcus_koreensis_DSM_44498_T	26.50	[24.1 - 28.9%]	0.16370	0.02	1.71
001_Rhodococcus_sp._WAY2	019_Rhodococcus_kroppenstedtii_DSM_44908_T	20.10	[17.9 - 22.5%]	0.21840	0	4.39
001_Rhodococcus_sp._WAY2	020_Rhodococcus_kunmingensis_DSM_45001_T	20.10	[17.8 - 22.5%]	0.21920	0	0.53
001_Rhodococcus_sp._WAY2	021_Rhodococcus_kyotonensis_JCM_23211_T	19.50	[17.3 - 21.9%]	0.22490	0	1.51
001_Rhodococcus_sp._WAY2	022_Rhodococcus_maanshanensis_DSM_44675_T	20.20	[18 - 22.7%]	0.21710	0	3.5
001_Rhodococcus_sp._WAY2	023_Rhodococcus_marinonascens_NBRC_14363_T	22.90	[20.6 - 25.3%]	0.19140	0	1.26
001_Rhodococcus_sp._WAY2	024_Rhodococcus_maris_DSM_43672_T	20.30	[18.1 - 22.7%]	0.21680	0	5.19
001_Rhodococcus_sp._WAY2	025_Rhodococcus_opacus_DSM_43205_T	27.70	[25.4 - 30.2%]	0.15540	0.04	1.61
001_Rhodococcus_sp._WAY2	026_Rhodococcus_phenolicus_DSM_44812_T	19.70	[17.5 - 22.2%]	0.22260	0	2.69
001_Rhodococcus_sp._WAY2	027_Rhodococcus_pyridinivorans_DSM_44555_T	20.20	[17.9 - 22.6%]	0.21810	0	2.18
001_Rhodococcus_sp._WAY2	028_Rhodococcus_qingshengii_JCM_15477_T	20.20	[18 - 22.6%]	0.21730	0	3.3
001_Rhodococcus_sp._WAY2	029_Rhodococcus_rhodnii_NBRC_100604_T	19.70	[17.5 - 22.1%]	0.22300	0	4
001_Rhodococcus_sp._WAY2	030_Rhodococcus_rhodochrous_DSM_43241_T	20.20	[18 - 22.6%]	0.21780	0	2.56
001_Rhodococcus_sp._WAY2	031_Rhodococcus_ruber_DSM_43338_T	20.40	[18.2 - 22.8%]	0.21530	0	5
001_Rhodococcus_sp._WAY2	032_Rhodococcus_rubripertincta_NBRC_101908_T	19.90	[17.7 - 22.3%]	0.22050	0	1.71
001_Rhodococcus_sp._WAY2	033_Rhodococcus_sputi_NBRC_100414_T	20.60	[18.3 - 23%]	0.21360	0	0.32
001_Rhodococcus_sp._WAY2	034_Rhodococcus_terrae_NRRL_B_16283_T	20.00	[17.8 - 22.4%]	0.22000	0	2.14
001_Rhodococcus_sp._WAY2	035_Rhodococcus_triatomae_DSM_44892_T	20.10	[17.9 - 22.5%]	0.21850	0	3.06
001_Rhodococcus_sp._WAY2	036_Rhodococcus_tukisamuensis_NBRC_100609_T	20.20	[18 - 22.6%]	0.21770	0	4.18
001_Rhodococcus_sp._WAY2	037_Rhodococcus_wratislaviensis_NBRC_100605_T	26.80	[24.4 - 29.2%]	0.16170	0.02	1.11
001_Rhodococcus_sp._WAY2	038_Rhodococcus_yunnanensis_NBRC_103083_T	19.70	[17.5 - 22.1%]	0.22340	0	1.77
001_Rhodococcus_sp._WAY2	039_Rhodococcus_zopfii_NBRC_100606_T	20.00	[17.8 - 22.4%]	0.22010	0	2.57
002_Rhodococcus_agglutinans_CCTCC_AB2014297_T	003_Rhodococcus_biphenylivorans_TG9_T	20.40	[18.2 - 22.8%]	0.21570	0	1.2
002_Rhodococcus_agglutinans_CCTCC_AB2014297_T	004_Rhodococcus_bronchialis_DSM_43247_T	19.60	[17.4 - 22%]	0.22400	0	2.2
002_Rhodococcus_agglutinans_CCTCC_AB2014297_T	005_Rhodococcus_chlorophenolicum_DSM_43826_T	19.50	[17.3 - 21.9%]	0.22580	0	0.82
002_Rhodococcus_agglutinans_CCTCC_AB2014297_T	006_Rhodococcus_coprophilus_NBRC_100603_T	19.80	[17.6 - 22.2%]	0.22190	0	2.36
002_Rhodococcus_agglutinans_CCTCC_AB2014297_T	007_Rhodococcus_corynebacterioides_DSM_20151_T	20.00	[17.8 - 22.5%]	0.21920	0	1
002_Rhodococcus_agglutinans_CCTCC_AB2014297_T	008_Rhodococcus_defluvii_Ca11_T	27.80	[25.4 - 30.3%]	0.15520	0.04	0.53
002_Rhodococcus_agglutinans_CCTCC_AB2014297_T	009_Rhodococcus_enclensis_NIO_1009_T	19.90	[17.7 - 22.3%]	0.22110	0	6.93

002_Rhodococcus_agglutinans_CCTCC_AB2014297_T	010_Rhodococcus_equi_DSM_20307_T	27.70	[25.3 - 30.2%]	0.15570	0.04	0.46
002_Rhodococcus_agglutinans_CCTCC_AB2014297_T	011_Rhodococcus_erythropolis_NBRC_15567_T	20.00	[17.8 - 22.4%]	0.22020	0	6.85
002_Rhodococcus_agglutinans_CCTCC_AB2014297_T	012_Rhodococcus_fascians_NBRC_12155_T	19.40	[17.2 - 21.8%]	0.22620	0	4.8
002_Rhodococcus_agglutinans_CCTCC_AB2014297_T	013_Rhodococcus_globerulus_NBRC_14531_T	19.60	[17.4 - 22%]	0.22440	0	7.57
002_Rhodococcus_agglutinans_CCTCC_AB2014297_T	014_Rhodococcus_gordoniae_DSM_44689_T	20.40	[18.1 - 22.8%]	0.21580	0	1.3
002_Rhodococcus_agglutinans_CCTCC_AB2014297_T	015_Rhodococcus_hoagii_DSM_20295_T	27.90	[25.5 - 30.4%]	0.15430	0.04	0.48
002_Rhodococcus_agglutinans_CCTCC_AB2014297_T	016_Rhodococcus_imtechensis_JCM_13270_T	20.50	[18.3 - 22.9%]	0.21440	0	2.02
002_Rhodococcus_agglutinans_CCTCC_AB2014297_T	017_Rhodococcus_jostii_NBRC_16295_T	20.70	[18.5 - 23.2%]	0.21190	0	2.35
002_Rhodococcus_agglutinans_CCTCC_AB2014297_T	018_Rhodococcus_koreensis_DSM_44498_T	20.60	[18.4 - 23.1%]	0.21290	0	1.87
002_Rhodococcus_agglutinans_CCTCC_AB2014297_T	019_Rhodococcus_kroppenstedtii_DSM_44908_T	20.30	[18.1 - 22.8%]	0.21610	0	0.82
002_Rhodococcus_agglutinans_CCTCC_AB2014297_T	020_Rhodococcus_kunmingensis_DSM_45001_T	20.20	[18 - 22.6%]	0.21790	0	3.05
002_Rhodococcus_agglutinans_CCTCC_AB2014297_T	021_Rhodococcus_kyotonensis_JCM_23211_T	19.40	[17.2 - 21.8%]	0.22670	0	5.09
002_Rhodococcus_agglutinans_CCTCC_AB2014297_T	022_Rhodococcus_maanshanensis_DSM_44675_T	21.40	[19.2 - 23.8%]	0.20510	0	0.08
002_Rhodococcus_agglutinans_CCTCC_AB2014297_T	023_Rhodococcus_marinonascens_NBRC_14363_T	20.00	[17.8 - 22.4%]	0.21960	0	4.84
002_Rhodococcus_agglutinans_CCTCC_AB2014297_T	024_Rhodococcus_maris_DSM_43672_T	19.50	[17.3 - 21.9%]	0.22540	0	1.61
002_Rhodococcus_agglutinans_CCTCC_AB2014297_T	025_Rhodococcus_opacus_DSM_43205_T	20.60	[18.3 - 23%]	0.21380	0	1.97
002_Rhodococcus_agglutinans_CCTCC_AB2014297_T	026_Rhodococcus_phenolicus_DSM_44812_T	20.70	[18.5 - 23.1%]	0.21230	0	0.89
002_Rhodococcus_agglutinans_CCTCC_AB2014297_T	027_Rhodococcus_pyridinivorans_DSM_44555_T	20.10	[17.9 - 22.6%]	0.21820	0	1.4
002_Rhodococcus_agglutinans_CCTCC_AB2014297_T	028_Rhodococcus_qingshengii_JCM_15477_T	20.00	[17.8 - 22.5%]	0.21920	0	6.88
002_Rhodococcus_agglutinans_CCTCC_AB2014297_T	029_Rhodococcus_rhodnii_NBRC_100604_T	20.40	[18.2 - 22.8%]	0.21530	0	0.43
002_Rhodococcus_agglutinans_CCTCC_AB2014297_T	030_Rhodococcus_rhodochrous_DSM_43241_T	20.70	[18.4 - 23.1%]	0.21250	0	1.02
002_Rhodococcus_agglutinans_CCTCC_AB2014297_T	031_Rhodococcus_ruber_DSM_43338_T	20.80	[18.6 - 23.2%]	0.21110	0	1.42
002_Rhodococcus_agglutinans_CCTCC_AB2014297_T	032_Rhodococcus_rubripertincta_NBRC_101908_T	20.00	[17.8 - 22.4%]	0.21980	0	1.86
002_Rhodococcus_agglutinans_CCTCC_AB2014297_T	033_Rhodococcus_sputi_NBRC_100414_T	20.40	[18.2 - 22.8%]	0.21540	0	3.9
002_Rhodococcus_agglutinans_CCTCC_AB2014297_T	034_Rhodococcus_terrae_NRRL_B_16283_T	20.20	[18 - 22.6%]	0.21790	0	1.44
002_Rhodococcus_agglutinans_CCTCC_AB2014297_T	035_Rhodococcus_triatomae_DSM_44892_T	20.50	[18.2 - 22.9%]	0.21480	0	0.52
002_Rhodococcus_agglutinans_CCTCC_AB2014297_T	036_Rhodococcus_tukisamuensis_NBRC_100609_T	21.90	[19.7 - 24.4%]	0.20010	0	0.61
002_Rhodococcus_agglutinans_CCTCC_AB2014297_T	037_Rhodococcus_wratislaviensis_NBRC_100605_T	20.40	[18.2 - 22.9%]	0.21490	0	2.47
002_Rhodococcus_agglutinans_CCTCC_AB2014297_T	038_Rhodococcus_yunnanensis_NBRC_103083_T	19.90	[17.7 - 22.3%]	0.22110	0	5.35

002_Rhodococcus_agglutinans_CCTCC_AB2014297_T	039_Rhodococcus_zopfii_NBRC_100606_T	20.70	[18.5 - 23.1%]	0.21210	0	1
003_Rhodococcus_biphenylivorans_TG9_T	004_Rhodococcus_bronchialis_DSM_43247_T	21.70	[19.4 - 24.1%]	0.20240	0	1
003_Rhodococcus_biphenylivorans_TG9_T	005_Rhodococcus_chlorophenolicum_DSM_43826_T	19.60	[17.4 - 22%]	0.22410	0	0.38
003_Rhodococcus_biphenylivorans_TG9_T	006_Rhodococcus_coprophilus_NBRC_100603_T	22.00	[19.8 - 24.5%]	0.19920	0	1.16
003_Rhodococcus_biphenylivorans_TG9_T	007_Rhodococcus_corynebacterioides_DSM_20151_T	20.20	[17.9 - 22.6%]	0.21800	0	2.2
003_Rhodococcus_biphenylivorans_TG9_T	008_Rhodococcus_defluvii_Ca11_T	20.20	[18 - 22.6%]	0.21760	0	0.67
003_Rhodococcus_biphenylivorans_TG9_T	009_Rhodococcus_enclensis_NIO_1009_T	19.70	[17.5 - 22.1%]	0.22300	0	5.73
003_Rhodococcus_biphenylivorans_TG9_T	010_Rhodococcus_equi_DSM_20307_T	20.20	[18 - 22.6%]	0.21780	0	0.74
003_Rhodococcus_biphenylivorans_TG9_T	011_Rhodococcus_erythropolis_NBRC_15567_T	19.70	[17.5 - 22.1%]	0.22350	0	5.65
003_Rhodococcus_biphenylivorans_TG9_T	012_Rhodococcus_fascians_NBRC_12155_T	19.60	[17.4 - 22%]	0.22430	0	3.6
003_Rhodococcus_biphenylivorans_TG9_T	013_Rhodococcus_globerulus_NBRC_14531_T	19.70	[17.5 - 22.1%]	0.22330	0	6.37
003_Rhodococcus_biphenylivorans_TG9_T	014_Rhodococcus_gordoniae_DSM_44689_T	42.70	[40.2 - 45.3%]	0.09090	5.12	0.1
003_Rhodococcus_biphenylivorans_TG9_T	015_Rhodococcus_hoagii_DSM_20295_T	20.50	[18.3 - 22.9%]	0.21460	0	0.72
003_Rhodococcus_biphenylivorans_TG9_T	016_Rhodococcus_imtechensis_JCM_13270_T	20.10	[17.9 - 22.5%]	0.21830	0	0.82
003_Rhodococcus_biphenylivorans_TG9_T	017_Rhodococcus_jostii_NBRC_16295_T	19.80	[17.6 - 22.2%]	0.22230	0	1.15
003_Rhodococcus_biphenylivorans_TG9_T	018_Rhodococcus_koreensis_DSM_44498_T	20.10	[17.9 - 22.5%]	0.21840	0	0.67
003_Rhodococcus_biphenylivorans_TG9_T	019_Rhodococcus_kroppenstedtii_DSM_44908_T	20.20	[18 - 22.6%]	0.21750	0	2.02
003_Rhodococcus_biphenylivorans_TG9_T	020_Rhodococcus_kunmingensis_DSM_45001_T	19.90	[17.7 - 22.3%]	0.22060	0	1.85
003_Rhodococcus_biphenylivorans_TG9_T	021_Rhodococcus_kyotonensis_JCM_23211_T	19.10	[16.9 - 21.5%]	0.22990	0	3.89
003_Rhodococcus_biphenylivorans_TG9_T	022_Rhodococcus_maanshanensis_DSM_44675_T	19.80	[17.6 - 22.2%]	0.22200	0	1.12
003_Rhodococcus_biphenylivorans_TG9_T	023_Rhodococcus_marinonascens_NBRC_14363_T	20.00	[17.8 - 22.4%]	0.21960	0	3.64
003_Rhodococcus_biphenylivorans_TG9_T	024_Rhodococcus_maris_DSM_43672_T	20.00	[17.8 - 22.4%]	0.22020	0	2.81
003_Rhodococcus_biphenylivorans_TG9_T	025_Rhodococcus_opacus_DSM_43205_T	20.10	[17.9 - 22.5%]	0.21840	0	0.77
003_Rhodococcus_biphenylivorans_TG9_T	026_Rhodococcus_phenolicus_DSM_44812_T	22.00	[19.8 - 24.5%]	0.19910	0	0.31
003_Rhodococcus_biphenylivorans_TG9_T	027_Rhodococcus_pyridinivorans_DSM_44555_T	88.30	[85.9 - 90.4%]	0.01390	95.2	0.2
003_Rhodococcus_biphenylivorans_TG9_T	028_Rhodococcus_qingshengii_JCM_15477_T	20.00	[17.8 - 22.4%]	0.21990	0	5.68
003_Rhodococcus_biphenylivorans_TG9_T	029_Rhodococcus_rhodnii_NBRC_100604_T	20.10	[17.9 - 22.5%]	0.21870	0	1.63
003_Rhodococcus_biphenylivorans_TG9_T	030_Rhodococcus_rhodochrous_DSM_43241_T	58.80	[56 - 61.6%]	0.05370	48.34	0.18
003_Rhodococcus_biphenylivorans_TG9_T	031_Rhodococcus_ruber_DSM_43338_T	21.60	[19.4 - 24.1%]	0.20300	0	2.62

003_Rhodococcus_biphenylivorans_TG9_T	032_Rhodococcus_rubripertincta_NBRC_101908_T	20.10	[17.9 - 22.5%]	0.21850	0	0.66
003_Rhodococcus_biphenylivorans_TG9_T	033_Rhodococcus_sputi_NBRC_100414_T	22.00	[19.8 - 24.5%]	0.19910	0	2.7
003_Rhodococcus_biphenylivorans_TG9_T	034_Rhodococcus_terrae_NRRL_B_16283_T	20.10	[17.9 - 22.5%]	0.21830	0	0.24
003_Rhodococcus_biphenylivorans_TG9_T	035_Rhodococcus_triatomae_DSM_44892_T	20.30	[18.1 - 22.7%]	0.21660	0	0.68
003_Rhodococcus_biphenylivorans_TG9_T	036_Rhodococcus_tukisamuensis_NBRC_100609_T	20.30	[18.1 - 22.7%]	0.21680	0	1.81
003_Rhodococcus_biphenylivorans_TG9_T	037_Rhodococcus_wratislaviensis_NBRC_100605_T	20.10	[17.9 - 22.5%]	0.21900	0	1.27
003_Rhodococcus_biphenylivorans_TG9_T	038_Rhodococcus_yunnanensis_NBRC_103083_T	19.30	[17.1 - 21.7%]	0.22800	0	4.15
003_Rhodococcus_biphenylivorans_TG9_T	039_Rhodococcus_zopfii_NBRC_100606_T	21.80	[19.6 - 24.3%]	0.20080	0	0.2
004_Rhodococcus_bronchialis_DSM_43247_T	005_Rhodococcus_chlorophenolicum_DSM_43826_T	19.60	[17.4 - 22%]	0.22470	0	1.38
004_Rhodococcus_bronchialis_DSM_43247_T	006_Rhodococcus_coprophilus_NBRC_100603_T	19.50	[17.3 - 21.8%]	0.22600	0	0.16
004_Rhodococcus_bronchialis_DSM_43247_T	007_Rhodococcus_corynebacterioides_DSM_20151_T	19.60	[17.4 - 22%]	0.22470	0	3.2
004_Rhodococcus_bronchialis_DSM_43247_T	008_Rhodococcus_defluvii_Ca11_T	19.80	[17.6 - 22.2%]	0.22240	0	1.67
004_Rhodococcus_bronchialis_DSM_43247_T	009_Rhodococcus_enclensis_NIO_1009_T	20.20	[18 - 22.6%]	0.21730	0	4.73
004_Rhodococcus_bronchialis_DSM_43247_T	010_Rhodococcus_equi_DSM_20307_T	19.90	[17.7 - 22.3%]	0.22120	0	1.75
004_Rhodococcus_bronchialis_DSM_43247_T	011_Rhodococcus_erythropolis_NBRC_15567_T	20.00	[17.8 - 22.4%]	0.21990	0	4.64
004_Rhodococcus_bronchialis_DSM_43247_T	012_Rhodococcus_fascians_NBRC_12155_T	19.80	[17.6 - 22.2%]	0.22190	0	2.6
004_Rhodococcus_bronchialis_DSM_43247_T	013_Rhodococcus_globerulus_NBRC_14531_T	19.80	[17.6 - 22.2%]	0.22240	0	5.37
004_Rhodococcus_bronchialis_DSM_43247_T	014_Rhodococcus_gordoniae_DSM_44689_T	20.30	[18.1 - 22.7%]	0.21650	0	0.9
004_Rhodococcus_bronchialis_DSM_43247_T	015_Rhodococcus_hoagii_DSM_20295_T	19.90	[17.7 - 22.3%]	0.22070	0	1.72
004_Rhodococcus_bronchialis_DSM_43247_T	016_Rhodococcus_imtechensis_JCM_13270_T	20.20	[18 - 22.6%]	0.21750	0	0.18
004_Rhodococcus_bronchialis_DSM_43247_T	017_Rhodococcus_jostii_NBRC_16295_T	19.30	[17.2 - 21.7%]	0.22730	0	0.14
004_Rhodococcus_bronchialis_DSM_43247_T	018_Rhodococcus_koreensis_DSM_44498_T	19.40	[17.2 - 21.8%]	0.22630	0	0.33
004_Rhodococcus_bronchialis_DSM_43247_T	019_Rhodococcus_kroppenstedtii_DSM_44908_T	19.80	[17.6 - 22.2%]	0.22200	0	3.02
004_Rhodococcus_bronchialis_DSM_43247_T	020_Rhodococcus_kunmingensis_DSM_45001_T	19.90	[17.7 - 22.3%]	0.22050	0	0.85
004_Rhodococcus_bronchialis_DSM_43247_T	021_Rhodococcus_kyotonensis_JCM_23211_T	19.60	[17.4 - 22%]	0.22440	0	2.88
004_Rhodococcus_bronchialis_DSM_43247_T	022_Rhodococcus_maanshanensis_DSM_44675_T	19.70	[17.5 - 22.1%]	0.22310	0	2.13
004_Rhodococcus_bronchialis_DSM_43247_T	023_Rhodococcus_marinonascens_NBRC_14363_T	20.30	[18.1 - 22.8%]	0.21610	0	2.63
004_Rhodococcus_bronchialis_DSM_43247_T	024_Rhodococcus_maris_DSM_43672_T	19.30	[17.1 - 21.7%]	0.22790	0	3.81
004_Rhodococcus_bronchialis_DSM_43247_T	025_Rhodococcus_opacus_DSM_43205_T	20.00	[17.8 - 22.4%]	0.21940	0	0.24

004_Rhodococcus_bronchialis_DSM_43247_T	026_Rhodococcus_phenolicus_DSM_44812_T	19.60	[17.4 - 22%]	0.22470	0	1.32
004_Rhodococcus_bronchialis_DSM_43247_T	027_Rhodococcus_pyridinivorans_DSM_44555_T	20.50	[18.3 - 23%]	0.21380	0	0.8
004_Rhodococcus_bronchialis_DSM_43247_T	028_Rhodococcus_qingshengii_JCM_15477_T	20.30	[18.1 - 22.7%]	0.21610	0	4.67
004_Rhodococcus_bronchialis_DSM_43247_T	029_Rhodococcus_rhodnii_NBRC_100604_T	20.00	[17.7 - 22.4%]	0.22030	0	2.63
004_Rhodococcus_bronchialis_DSM_43247_T	030_Rhodococcus_rhodochrous_DSM_43241_T	19.60	[17.4 - 22%]	0.22460	0	1.19
004_Rhodococcus_bronchialis_DSM_43247_T	031_Rhodococcus_ruber_DSM_43338_T	20.00	[17.8 - 22.4%]	0.22010	0	3.62
004_Rhodococcus_bronchialis_DSM_43247_T	032_Rhodococcus_rubripertincta_NBRC_101908_T	21.70	[19.5 - 24.1%]	0.20210	0	0.34
004_Rhodococcus_bronchialis_DSM_43247_T	033_Rhodococcus_sputi_NBRC_100414_T	21.30	[19 - 23.7%]	0.20620	0	1.7
004_Rhodococcus_bronchialis_DSM_43247_T	034_Rhodococcus_terrae_NRRL_B_16283_T	21.50	[19.3 - 24%]	0.20380	0	0.76
004_Rhodococcus_bronchialis_DSM_43247_T	035_Rhodococcus_triatomae_DSM_44892_T	19.60	[17.4 - 22%]	0.22460	0	1.69
004_Rhodococcus_bronchialis_DSM_43247_T	036_Rhodococcus_tukisamuensis_NBRC_100609_T	19.50	[17.3 - 21.9%]	0.22550	0	2.81
004_Rhodococcus_bronchialis_DSM_43247_T	037_Rhodococcus_wratislaviensis_NBRC_100605_T	19.70	[17.5 - 22.1%]	0.22280	0	0.27
004_Rhodococcus_bronchialis_DSM_43247_T	038_Rhodococcus_yunnanensis_NBRC_103083_T	19.80	[17.6 - 22.2%]	0.22200	0	3.15
004_Rhodococcus_bronchialis_DSM_43247_T	039_Rhodococcus_zopfii_NBRC_100606_T	20.30	[18.1 - 22.7%]	0.21620	0	1.2
005_Rhodococcus_chlorophenolicum_DSM_43826_T	006_Rhodococcus_coprophilus_NBRC_100603_T	19.30	[17.1 - 21.7%]	0.22820	0	1.55
005_Rhodococcus_chlorophenolicum_DSM_43826_T	007_Rhodococcus_corynebacterioides_DSM_20151_T	19.10	[16.9 - 21.5%]	0.23030	0	1.82
005_Rhodococcus_chlorophenolicum_DSM_43826_T	008_Rhodococcus_defluvii_Ca11_T	19.60	[17.4 - 22%]	0.22480	0	0.29
005_Rhodococcus_chlorophenolicum_DSM_43826_T	009_Rhodococcus_enclensis_NIO_1009_T	19.40	[17.2 - 21.8%]	0.22700	0	6.12
005_Rhodococcus_chlorophenolicum_DSM_43826_T	010_Rhodococcus_equi_DSM_20307_T	19.50	[17.3 - 21.9%]	0.22530	0	0.36
005_Rhodococcus_chlorophenolicum_DSM_43826_T	011_Rhodococcus_erythropolis_NBRC_15567_T	19.90	[17.6 - 22.3%]	0.22140	0	6.03
005_Rhodococcus_chlorophenolicum_DSM_43826_T	012_Rhodococcus_fascians_NBRC_12155_T	19.30	[17.1 - 21.7%]	0.22740	0	3.98
005_Rhodococcus_chlorophenolicum_DSM_43826_T	013_Rhodococcus_globerulus_NBRC_14531_T	19.10	[17 - 21.5%]	0.22960	0	6.76
005_Rhodococcus_chlorophenolicum_DSM_43826_T	014_Rhodococcus_gordoniae_DSM_44689_T	19.20	[17 - 21.6%]	0.22910	0	0.48
005_Rhodococcus_chlorophenolicum_DSM_43826_T	015_Rhodococcus_hoagii_DSM_20295_T	19.50	[17.3 - 21.9%]	0.22510	0	0.34
005_Rhodococcus_chlorophenolicum_DSM_43826_T	016_Rhodococcus_imtechensis_JCM_13270_T	19.00	[16.8 - 21.4%]	0.23160	0	1.2
005_Rhodococcus_chlorophenolicum_DSM_43826_T	017_Rhodococcus_jostii_NBRC_16295_T	19.40	[17.2 - 21.8%]	0.22640	0	1.53
005_Rhodococcus_chlorophenolicum_DSM_43826_T	018_Rhodococcus_koreensis_DSM_44498_T	19.30	[17.2 - 21.7%]	0.22720	0	1.05
005_Rhodococcus_chlorophenolicum_DSM_43826_T	019_Rhodococcus_kroppenstedtii_DSM_44908_T	19.30	[17.1 - 21.7%]	0.22800	0	1.64
005_Rhodococcus_chlorophenolicum_DSM_43826_T	020_Rhodococcus_kunmingensis_DSM_45001_T	19.40	[17.2 - 21.8%]	0.22620	0	2.23

005_Rhodococcus_chlorophenolicum_DSM_43826_T	021_Rhodococcus_kyotonensis_JCM_23211_T	19.00	[16.8 - 21.4%]	0.23110	0	4.27
005_Rhodococcus_chlorophenolicum_DSM_43826_T	022_Rhodococcus_maanshanensis_DSM_44675_T	19.40	[17.2 - 21.8%]	0.22620	0	0.74
005_Rhodococcus_chlorophenolicum_DSM_43826_T	023_Rhodococcus_marinonascens_NBRC_14363_T	19.10	[16.9 - 21.5%]	0.22980	0	4.02
005_Rhodococcus_chlorophenolicum_DSM_43826_T	024_Rhodococcus_maris_DSM_43672_T	19.10	[16.9 - 21.5%]	0.22990	0	2.43
005_Rhodococcus_chlorophenolicum_DSM_43826_T	025_Rhodococcus_opacus_DSM_43205_T	19.20	[17 - 21.6%]	0.22860	0	1.15
005_Rhodococcus_chlorophenolicum_DSM_43826_T	026_Rhodococcus_phenolicus_DSM_44812_T	19.50	[17.3 - 21.9%]	0.22550	0	0.07
005_Rhodococcus_chlorophenolicum_DSM_43826_T	027_Rhodococcus_pyridinivorans_DSM_44555_T	19.70	[17.5 - 22.1%]	0.22310	0	0.58
005_Rhodococcus_chlorophenolicum_DSM_43826_T	028_Rhodococcus_qingshengii_JCM_15477_T	19.70	[17.5 - 22.1%]	0.22330	0	6.06
005_Rhodococcus_chlorophenolicum_DSM_43826_T	029_Rhodococcus_rhodnii_NBRC_100604_T	19.50	[17.3 - 21.9%]	0.22560	0	1.25
005_Rhodococcus_chlorophenolicum_DSM_43826_T	030_Rhodococcus_rhodochrous_DSM_43241_T	19.60	[17.4 - 22%]	0.22450	0	0.2
005_Rhodococcus_chlorophenolicum_DSM_43826_T	031_Rhodococcus_ruber_DSM_43338_T	19.40	[17.2 - 21.8%]	0.22710	0	2.24
005_Rhodococcus_chlorophenolicum_DSM_43826_T	032_Rhodococcus_rubripertincta_NBRC_101908_T	19.30	[17.1 - 21.7%]	0.22810	0	1.04
005_Rhodococcus_chlorophenolicum_DSM_43826_T	033_Rhodococcus_sputi_NBRC_100414_T	19.90	[17.7 - 22.3%]	0.22120	0	3.08
005_Rhodococcus_chlorophenolicum_DSM_43826_T	034_Rhodococcus_terrae_NRRL_B_16283_T	19.30	[17.1 - 21.7%]	0.22800	0	0.62
005_Rhodococcus_chlorophenolicum_DSM_43826_T	035_Rhodococcus_triatomae_DSM_44892_T	19.80	[17.6 - 22.2%]	0.22250	0	0.3
005_Rhodococcus_chlorophenolicum_DSM_43826_T	036_Rhodococcus_tukisamuensis_NBRC_100609_T	19.20	[17 - 21.5%]	0.22940	0	1.42
005_Rhodococcus_chlorophenolicum_DSM_43826_T	037_Rhodococcus_wratislaviensis_NBRC_100605_T	19.30	[17.1 - 21.7%]	0.22780	0	1.65
005_Rhodococcus_chlorophenolicum_DSM_43826_T	038_Rhodococcus_yunnanensis_NBRC_103083_T	19.50	[17.3 - 21.9%]	0.22590	0	4.53
005_Rhodococcus_chlorophenolicum_DSM_43826_T	039_Rhodococcus_zopfii_NBRC_100606_T	19.40	[17.2 - 21.8%]	0.22640	0	0.18
006_Rhodococcus_coprophilus_NBRC_100603_T	007_Rhodococcus_corynebacterioides_DSM_20151_T	19.50	[17.3 - 21.9%]	0.22550	0	3.37
006_Rhodococcus_coprophilus_NBRC_100603_T	008_Rhodococcus_defluvii_Ca11_T	19.70	[17.5 - 22.1%]	0.22270	0	1.83
006_Rhodococcus_coprophilus_NBRC_100603_T	009_Rhodococcus_enclensis_NIO_1009_T	19.20	[17.1 - 21.6%]	0.22840	0	4.57
006_Rhodococcus_coprophilus_NBRC_100603_T	010_Rhodococcus_equi_DSM_20307_T	19.90	[17.7 - 22.3%]	0.22070	0	1.91
006_Rhodococcus_coprophilus_NBRC_100603_T	011_Rhodococcus_erythropolis_NBRC_15567_T	19.70	[17.5 - 22.1%]	0.22360	0	4.48
006_Rhodococcus_coprophilus_NBRC_100603_T	012_Rhodococcus_fascians_NBRC_12155_T	19.50	[17.3 - 21.9%]	0.22570	0	2.44
006_Rhodococcus_coprophilus_NBRC_100603_T	013_Rhodococcus_globerulus_NBRC_14531_T	19.10	[16.9 - 21.4%]	0.23080	0	5.21
006_Rhodococcus_coprophilus_NBRC_100603_T	014_Rhodococcus_gordoniae_DSM_44689_T	22.00	[19.7 - 24.4%]	0.19970	0	1.07
006_Rhodococcus_coprophilus_NBRC_100603_T	015_Rhodococcus_hoagii_DSM_20295_T	20.10	[17.9 - 22.5%]	0.21840	0	1.89
006_Rhodococcus_coprophilus_NBRC_100603_T	016_Rhodococcus_imtechensis_JCM_13270_T	19.80	[17.6 - 22.2%]	0.22250	0	0.34

006_Rhodococcus_coprophilus_NBRC_100603_T	017_Rhodococcus_jostii_NBRC_16295_T	19.60	[17.4 - 22%]	0.22450	0	0.02
006_Rhodococcus_coprophilus_NBRC_100603_T	018_Rhodococcus_koreensis_DSM_44498_T	19.50	[17.3 - 21.9%]	0.22590	0	0.49
006_Rhodococcus_coprophilus_NBRC_100603_T	019_Rhodococcus_kroppenstedtii_DSM_44908_T	18.90	[16.8 - 21.3%]	0.23220	0	3.18
006_Rhodococcus_coprophilus_NBRC_100603_T	020_Rhodococcus_kunmingensis_DSM_45001_T	19.60	[17.5 - 22%]	0.22370	0	0.69
006_Rhodococcus_coprophilus_NBRC_100603_T	021_Rhodococcus_kyotonensis_JCM_23211_T	18.80	[16.6 - 21.2%]	0.23380	0	2.72
006_Rhodococcus_coprophilus_NBRC_100603_T	022_Rhodococcus_maanshanensis_DSM_44675_T	19.90	[17.7 - 22.3%]	0.22120	0	2.29
006_Rhodococcus_coprophilus_NBRC_100603_T	023_Rhodococcus_marinonascens_NBRC_14363_T	19.50	[17.3 - 21.9%]	0.22570	0	2.47
006_Rhodococcus_coprophilus_NBRC_100603_T	024_Rhodococcus_maris_DSM_43672_T	19.60	[17.4 - 22%]	0.22450	0	3.98
006_Rhodococcus_coprophilus_NBRC_100603_T	025_Rhodococcus_opacus_DSM_43205_T	19.70	[17.5 - 22.1%]	0.22260	0	0.4
006_Rhodococcus_coprophilus_NBRC_100603_T	026_Rhodococcus_phenolicus_DSM_44812_T	21.30	[19.1 - 23.8%]	0.20590	0	1.48
006_Rhodococcus_coprophilus_NBRC_100603_T	027_Rhodococcus_pyridinivorans_DSM_44555_T	21.90	[19.6 - 24.4%]	0.20020	0	0.97
006_Rhodococcus_coprophilus_NBRC_100603_T	028_Rhodococcus_qingshengii_JCM_15477_T	19.40	[17.2 - 21.8%]	0.22650	0	4.51
006_Rhodococcus_coprophilus_NBRC_100603_T	029_Rhodococcus_rhodnii_NBRC_100604_T	19.70	[17.5 - 22.1%]	0.22310	0	2.79
006_Rhodococcus_coprophilus_NBRC_100603_T	030_Rhodococcus_rhodochrous_DSM_43241_T	22.00	[19.8 - 24.5%]	0.19910	0	1.35
006_Rhodococcus_coprophilus_NBRC_100603_T	031_Rhodococcus_ruber_DSM_43338_T	21.00	[18.8 - 23.4%]	0.20920	0	3.78
006_Rhodococcus_coprophilus_NBRC_100603_T	032_Rhodococcus_rubripertincta_NBRC_101908_T	19.40	[17.2 - 21.8%]	0.22670	0	0.5
006_Rhodococcus_coprophilus_NBRC_100603_T	033_Rhodococcus_sputi_NBRC_100414_T	20.40	[18.2 - 22.8%]	0.21560	0	1.54
006_Rhodococcus_coprophilus_NBRC_100603_T	034_Rhodococcus_terrae_NRRL_B_16283_T	20.20	[18 - 22.7%]	0.21700	0	0.92
006_Rhodococcus_coprophilus_NBRC_100603_T	035_Rhodococcus_triatomae_DSM_44892_T	20.00	[17.8 - 22.4%]	0.22000	0	1.85
006_Rhodococcus_coprophilus_NBRC_100603_T	036_Rhodococcus_tukisamuensis_NBRC_100609_T	20.10	[17.9 - 22.6%]	0.21820	0	2.97
006_Rhodococcus_coprophilus_NBRC_100603_T	037_Rhodococcus_wratislaviensis_NBRC_100605_T	19.70	[17.5 - 22.1%]	0.22260	0	0.1
006_Rhodococcus_coprophilus_NBRC_100603_T	038_Rhodococcus_yunnanensis_NBRC_103083_T	18.80	[16.6 - 21.2%]	0.23400	0	2.99
006_Rhodococcus_coprophilus_NBRC_100603_T	039_Rhodococcus_zopfii_NBRC_100606_T	21.10	[18.9 - 23.6%]	0.20770	0	1.36
007_Rhodococcus_corynebacterioides_DSM_20151_T	008_Rhodococcus_defluvii_Ca11_T	20.30	[18.1 - 22.8%]	0.21590	0	1.53
007_Rhodococcus_corynebacterioides_DSM_20151_T	009_Rhodococcus_enclensis_NIO_1009_T	20.00	[17.8 - 22.4%]	0.21930	0	7.94
007_Rhodococcus_corynebacterioides_DSM_20151_T	010_Rhodococcus_equi_DSM_20307_T	20.30	[18.1 - 22.7%]	0.21670	0	1.46
007_Rhodococcus_corynebacterioides_DSM_20151_T	011_Rhodococcus_erythropolis_NBRC_15567_T	20.10	[17.8 - 22.5%]	0.21910	0	7.85
007_Rhodococcus_corynebacterioides_DSM_20151_T	012_Rhodococcus_fascians_NBRC_12155_T	19.70	[17.5 - 22.1%]	0.22270	0	5.8
007_Rhodococcus_corynebacterioides_DSM_20151_T	013_Rhodococcus_globerulus_NBRC_14531_T	19.90	[17.7 - 22.3%]	0.22110	0	8.58

007_Rhodococcus_corynebacterioides_DSM_20151_T	014_Rhodococcus_gordoniae_DSM_44689_T	19.70	[17.5 - 22.1%]	0.22320	0	2.3
007_Rhodococcus_corynebacterioides_DSM_20151_T	015_Rhodococcus_hoagii_DSM_20295_T	20.50	[18.2 - 22.9%]	0.21480	0	1.48
007_Rhodococcus_corynebacterioides_DSM_20151_T	016_Rhodococcus_imtechensis_JCM_13270_T	19.90	[17.7 - 22.3%]	0.22130	0	3.03
007_Rhodococcus_corynebacterioides_DSM_20151_T	017_Rhodococcus_jostii_NBRC_16295_T	19.80	[17.6 - 22.2%]	0.22220	0	3.35
007_Rhodococcus_corynebacterioides_DSM_20151_T	018_Rhodococcus_koreensis_DSM_44498_T	19.90	[17.7 - 22.3%]	0.22090	0	2.87
007_Rhodococcus_corynebacterioides_DSM_20151_T	019_Rhodococcus_kroppenstedtii_DSM_44908_T	43.60	[41 - 46.1%]	0.08830	6.16	0.18
007_Rhodococcus_corynebacterioides_DSM_20151_T	020_Rhodococcus_kunmingensis_DSM_45001_T	20.10	[17.9 - 22.5%]	0.21830	0	4.05
007_Rhodococcus_corynebacterioides_DSM_20151_T	021_Rhodococcus_kyotonensis_JCM_23211_T	19.70	[17.5 - 22.1%]	0.22280	0	6.09
007_Rhodococcus_corynebacterioides_DSM_20151_T	022_Rhodococcus_maanshanensis_DSM_44675_T	19.80	[17.6 - 22.2%]	0.22160	0	1.08
007_Rhodococcus_corynebacterioides_DSM_20151_T	023_Rhodococcus_marinonascens_NBRC_14363_T	19.80	[17.6 - 22.2%]	0.22190	0	5.84
007_Rhodococcus_corynebacterioides_DSM_20151_T	024_Rhodococcus_maris_DSM_43672_T	19.50	[17.3 - 21.9%]	0.22520	0	0.61
007_Rhodococcus_corynebacterioides_DSM_20151_T	025_Rhodococcus_opacus_DSM_43205_T	19.90	[17.7 - 22.3%]	0.22040	0	2.97
007_Rhodococcus_corynebacterioides_DSM_20151_T	026_Rhodococcus_phenolicus_DSM_44812_T	19.40	[17.2 - 21.8%]	0.22650	0	1.89
007_Rhodococcus_corynebacterioides_DSM_20151_T	027_Rhodococcus_pyridinivorans_DSM_44555_T	20.00	[17.8 - 22.4%]	0.21950	0	2.4
007_Rhodococcus_corynebacterioides_DSM_20151_T	028_Rhodococcus_qingshengii_JCM_15477_T	20.20	[18 - 22.6%]	0.21790	0	7.88
007_Rhodococcus_corynebacterioides_DSM_20151_T	029_Rhodococcus_rhodnii_NBRC_100604_T	19.60	[17.4 - 22%]	0.22450	0	0.57
007_Rhodococcus_corynebacterioides_DSM_20151_T	030_Rhodococcus_rhodochrous_DSM_43241_T	19.90	[17.7 - 22.3%]	0.22140	0	2.02
007_Rhodococcus_corynebacterioides_DSM_20151_T	031_Rhodococcus_ruber_DSM_43338_T	19.80	[17.6 - 22.2%]	0.22200	0	0.42
007_Rhodococcus_corynebacterioides_DSM_20151_T	032_Rhodococcus_rubripertincta_NBRC_101908_T	19.90	[17.7 - 22.4%]	0.22040	0	2.86
007_Rhodococcus_corynebacterioides_DSM_20151_T	033_Rhodococcus_sputi_NBRC_100414_T	19.50	[17.3 - 21.9%]	0.22510	0	4.9
007_Rhodococcus_corynebacterioides_DSM_20151_T	034_Rhodococcus_terrae_NRRL_B_16283_T	19.80	[17.6 - 22.2%]	0.22150	0	2.44
007_Rhodococcus_corynebacterioides_DSM_20151_T	035_Rhodococcus_triatomae_DSM_44892_T	20.00	[17.8 - 22.4%]	0.22010	0	1.52
007_Rhodococcus_corynebacterioides_DSM_20151_T	036_Rhodococcus_tukisamuensis_NBRC_100609_T	20.00	[17.8 - 22.5%]	0.21920	0	0.4
007_Rhodococcus_corynebacterioides_DSM_20151_T	037_Rhodococcus_wratislaviensis_NBRC_100605_T	20.00	[17.8 - 22.4%]	0.22010	0	3.47
007_Rhodococcus_corynebacterioides_DSM_20151_T	038_Rhodococcus_yunnanensis_NBRC_103083_T	19.60	[17.4 - 22%]	0.22380	0	6.35
007_Rhodococcus_corynebacterioides_DSM_20151_T	039_Rhodococcus_zopfii_NBRC_100606_T	19.60	[17.4 - 22%]	0.22450	0	2
008_Rhodococcus_defluvii_Ca11_T	009_Rhodococcus_enclensis_NIO_1009_T	20.10	[17.9 - 22.6%]	0.21820	0	6.4
008_Rhodococcus_defluvii_Ca11_T	010_Rhodococcus_equi_DSM_20307_T	26.90	[24.6 - 29.4%]	0.16060	0.03	0.08
008_Rhodococcus_defluvii_Ca11_T	011_Rhodococcus_erythropolis_NBRC_15567_T	20.10	[17.9 - 22.5%]	0.21900	0	6.32

008_Rhodococcus_defluvii_Ca11_T	012_Rhodococcus_fascians_NBRC_12155_T	19.40	[17.2 - 21.8%]	0.22660	0	4.27
008_Rhodococcus_defluvii_Ca11_T	013_Rhodococcus_globerulus_NBRC_14531_T	19.80	[17.6 - 22.2%]	0.22210	0	7.04
008_Rhodococcus_defluvii_Ca11_T	014_Rhodococcus_gordoniae_DSM_44689_T	20.50	[18.3 - 22.9%]	0.21450	0	0.77
008_Rhodococcus_defluvii_Ca11_T	015_Rhodococcus_hoagii_DSM_20295_T	27.00	[24.7 - 29.5%]	0.16000	0.03	0.05
008_Rhodococcus_defluvii_Ca11_T	016_Rhodococcus_imtechensis_JCM_13270_T	20.60	[18.4 - 23%]	0.21330	0	1.49
008_Rhodococcus_defluvii_Ca11_T	017_Rhodococcus_jostii_NBRC_16295_T	20.70	[18.5 - 23.1%]	0.21200	0	1.81
008_Rhodococcus_defluvii_Ca11_T	018_Rhodococcus_koreensis_DSM_44498_T	20.60	[18.4 - 23%]	0.21340	0	1.34
008_Rhodococcus_defluvii_Ca11_T	019_Rhodococcus_kroppenstedtii_DSM_44908_T	20.20	[18 - 22.6%]	0.21780	0	1.35
008_Rhodococcus_defluvii_Ca11_T	020_Rhodococcus_kunmingensis_DSM_45001_T	20.30	[18.1 - 22.8%]	0.21610	0	2.52
008_Rhodococcus_defluvii_Ca11_T	021_Rhodococcus_kyotonensis_JCM_23211_T	19.60	[17.4 - 22%]	0.22390	0	4.56
008_Rhodococcus_defluvii_Ca11_T	022_Rhodococcus_maanshanensis_DSM_44675_T	21.20	[18.9 - 23.6%]	0.20750	0	0.45
008_Rhodococcus_defluvii_Ca11_T	023_Rhodococcus_marinonascens_NBRC_14363_T	19.90	[17.7 - 22.3%]	0.22130	0	4.31
008_Rhodococcus_defluvii_Ca11_T	024_Rhodococcus_maris_DSM_43672_T	19.90	[17.7 - 22.3%]	0.22130	0	2.14
008_Rhodococcus_defluvii_Ca11_T	025_Rhodococcus_opacus_DSM_43205_T	20.60	[18.4 - 23%]	0.21300	0	1.44
008_Rhodococcus_defluvii_Ca11_T	026_Rhodococcus_phenolicus_DSM_44812_T	20.50	[18.3 - 22.9%]	0.21450	0	0.36
008_Rhodococcus_defluvii_Ca11_T	027_Rhodococcus_pyridinivorans_DSM_44555_T	20.00	[17.8 - 22.4%]	0.21950	0	0.87
008_Rhodococcus_defluvii_Ca11_T	028_Rhodococcus_qingshengii_JCM_15477_T	20.20	[18 - 22.6%]	0.21770	0	6.35
008_Rhodococcus_defluvii_Ca11_T	029_Rhodococcus_rhodnii_NBRC_100604_T	20.80	[18.6 - 23.2%]	0.21140	0	0.96
008_Rhodococcus_defluvii_Ca11_T	030_Rhodococcus_rhodochrous_DSM_43241_T	20.70	[18.5 - 23.1%]	0.21190	0	0.48
008_Rhodococcus_defluvii_Ca11_T	031_Rhodococcus_ruber_DSM_43338_T	20.60	[18.4 - 23%]	0.21310	0	1.95
008_Rhodococcus_defluvii_Ca11_T	032_Rhodococcus_rubripertincta_NBRC_101908_T	20.60	[18.4 - 23%]	0.21350	0	1.33
008_Rhodococcus_defluvii_Ca11_T	033_Rhodococcus_sputi_NBRC_100414_T	20.60	[18.4 - 23%]	0.21310	0	3.37
008_Rhodococcus_defluvii_Ca11_T	034_Rhodococcus_terrae_NRRL_B_16283_T	19.90	[17.7 - 22.3%]	0.22040	0	0.91
008_Rhodococcus_defluvii_Ca11_T	035_Rhodococcus_triatomae_DSM_44892_T	20.50	[18.2 - 22.9%]	0.21460	0	0.01
008_Rhodococcus_defluvii_Ca11_T	036_Rhodococcus_tukisamuensis_NBRC_100609_T	21.80	[19.5 - 24.2%]	0.20140	0	1.14
008_Rhodococcus_defluvii_Ca11_T	037_Rhodococcus_wratislaviensis_NBRC_100605_T	20.50	[18.3 - 22.9%]	0.21430	0	1.94
008_Rhodococcus_defluvii_Ca11_T	038_Rhodococcus_yunnanensis_NBRC_103083_T	19.70	[17.5 - 22.1%]	0.22300	0	4.82
008_Rhodococcus_defluvii_Ca11_T	039_Rhodococcus_zopfii_NBRC_100606_T	20.70	[18.4 - 23.1%]	0.21270	0	0.47
009_Rhodococcus_enclensis_NIO_1009_T	010_Rhodococcus_equi_DSM_20307_T	19.60	[17.4 - 22%]	0.22460	0	6.48

009_Rhodococcus_enclensis_NIO_1009_T	011_Rhodococcus_erythropolis_NBRC_15567_T	62.20	[59.3 - 65%]	0.04790	59.34	0.09
009_Rhodococcus_enclensis_NIO_1009_T	012_Rhodococcus_fascians_NBRC_12155_T	19.40	[17.3 - 21.8%]	0.22610	0	2.13
009_Rhodococcus_enclensis_NIO_1009_T	013_Rhodococcus_globerulus_NBRC_14531_T	24.00	[21.7 - 26.4%]	0.18230	0	0.64
009_Rhodococcus_enclensis_NIO_1009_T	014_Rhodococcus_gordoniae_DSM_44689_T	19.70	[17.5 - 22.1%]	0.22340	0	5.64
009_Rhodococcus_enclensis_NIO_1009_T	015_Rhodococcus_hoagii_DSM_20295_T	19.60	[17.4 - 22%]	0.22390	0	6.46
009_Rhodococcus_enclensis_NIO_1009_T	016_Rhodococcus_imtechensis_JCM_13270_T	20.20	[18 - 22.6%]	0.21770	0	4.91
009_Rhodococcus_enclensis_NIO_1009_T	017_Rhodococcus_jostii_NBRC_16295_T	20.10	[17.9 - 22.5%]	0.21830	0	4.59
009_Rhodococcus_enclensis_NIO_1009_T	018_Rhodococcus_koreensis_DSM_44498_T	20.20	[17.9 - 22.6%]	0.21800	0	5.06
009_Rhodococcus_enclensis_NIO_1009_T	019_Rhodococcus_kroppenstedtii_DSM_44908_T	20.70	[18.4 - 23.1%]	0.21270	0	7.75
009_Rhodococcus_enclensis_NIO_1009_T	020_Rhodococcus_kunmingensis_DSM_45001_T	19.70	[17.5 - 22.1%]	0.22260	0	3.88
009_Rhodococcus_enclensis_NIO_1009_T	021_Rhodococcus_kyotonensis_JCM_23211_T	19.50	[17.3 - 21.9%]	0.22570	0	1.85
009_Rhodococcus_enclensis_NIO_1009_T	022_Rhodococcus_maanshanensis_DSM_44675_T	19.90	[17.7 - 22.3%]	0.22100	0	6.86
009_Rhodococcus_enclensis_NIO_1009_T	023_Rhodococcus_marinonascens_NBRC_14363_T	19.80	[17.6 - 22.2%]	0.22230	0	2.1
009_Rhodococcus_enclensis_NIO_1009_T	024_Rhodococcus_maris_DSM_43672_T	18.90	[16.7 - 21.2%]	0.23310	0	8.55
009_Rhodococcus_enclensis_NIO_1009_T	025_Rhodococcus_opacus_DSM_43205_T	20.50	[18.3 - 22.9%]	0.21420	0	4.97
009_Rhodococcus_enclensis_NIO_1009_T	026_Rhodococcus_phenolicus_DSM_44812_T	19.60	[17.4 - 22%]	0.22400	0	6.05
009_Rhodococcus_enclensis_NIO_1009_T	027_Rhodococcus_pyridinivorans_DSM_44555_T	19.70	[17.5 - 22.1%]	0.22300	0	5.54
009_Rhodococcus_enclensis_NIO_1009_T	028_Rhodococcus_qingshengii_JCM_15477_T	88.00	[85.5 - 90.1%]	0.01430	95.06	0.06
009_Rhodococcus_enclensis_NIO_1009_T	029_Rhodococcus_rhodnii_NBRC_100604_T	19.80	[17.6 - 22.2%]	0.22150	0	7.36
009_Rhodococcus_enclensis_NIO_1009_T	030_Rhodococcus_rhodochrous_DSM_43241_T	19.60	[17.4 - 22%]	0.22450	0	5.92
009_Rhodococcus_enclensis_NIO_1009_T	031_Rhodococcus_ruber_DSM_43338_T	19.70	[17.5 - 22.1%]	0.22260	0	8.36
009_Rhodococcus_enclensis_NIO_1009_T	032_Rhodococcus_rubripertincta_NBRC_101908_T	19.80	[17.6 - 22.2%]	0.22260	0	5.07
009_Rhodococcus_enclensis_NIO_1009_T	033_Rhodococcus_sputi_NBRC_100414_T	20.00	[17.8 - 22.4%]	0.22020	0	3.03
009_Rhodococcus_enclensis_NIO_1009_T	034_Rhodococcus_terrae_NRRL_B_16283_T	19.60	[17.4 - 22%]	0.22440	0	5.49
009_Rhodococcus_enclensis_NIO_1009_T	035_Rhodococcus_triatomae_DSM_44892_T	19.70	[17.5 - 22.1%]	0.22350	0	6.42
009_Rhodococcus_enclensis_NIO_1009_T	036_Rhodococcus_tukisamuensis_NBRC_100609_T	20.00	[17.7 - 22.4%]	0.22030	0	7.54
009_Rhodococcus_enclensis_NIO_1009_T	037_Rhodococcus_wratislaviensis_NBRC_100605_T	20.20	[18 - 22.6%]	0.21790	0	4.47
009_Rhodococcus_enclensis_NIO_1009_T	038_Rhodococcus_yunnanensis_NBRC_103083_T	19.70	[17.5 - 22.1%]	0.22320	0	1.58
009_Rhodococcus_enclensis_NIO_1009_T	039_Rhodococcus_zopfii_NBRC_100606_T	19.50	[17.3 - 21.9%]	0.22510	0	5.93

010_Rhodococcus_equi_DSM_20307_T	011_Rhodococcus_erythropolis_NBRC_15567_T	19.90	[17.7 - 22.3%]	0.22070	0	6.39
010_Rhodococcus_equi_DSM_20307_T	012_Rhodococcus_fascians_NBRC_12155_T	19.50	[17.3 - 21.9%]	0.22590	0	4.35
010_Rhodococcus_equi_DSM_20307_T	013_Rhodococcus_globerulus_NBRC_14531_T	19.80	[17.6 - 22.3%]	0.22140	0	7.12
010_Rhodococcus_equi_DSM_20307_T	014_Rhodococcus_gordoniae_DSM_44689_T	20.10	[17.9 - 22.6%]	0.21810	0	0.84
010_Rhodococcus_equi_DSM_20307_T	015_Rhodococcus_hoagii_DSM_20295_T	91.00	[88.8 - 92.8%]	0.01100	96.12	0.02
010_Rhodococcus_equi_DSM_20307_T	016_Rhodococcus_imtechensis_JCM_13270_T	20.20	[18 - 22.6%]	0.21760	0	1.57
010_Rhodococcus_equi_DSM_20307_T	017_Rhodococcus_jostii_NBRC_16295_T	20.20	[18 - 22.6%]	0.21790	0	1.89
010_Rhodococcus_equi_DSM_20307_T	018_Rhodococcus_koreensis_DSM_44498_T	20.20	[18 - 22.6%]	0.21730	0	1.42
010_Rhodococcus_equi_DSM_20307_T	019_Rhodococcus_kroppenstedtii_DSM_44908_T	20.00	[17.8 - 22.4%]	0.21950	0	1.27
010_Rhodococcus_equi_DSM_20307_T	020_Rhodococcus_kunmingensis_DSM_45001_T	20.00	[17.8 - 22.4%]	0.22010	0	2.6
010_Rhodococcus_equi_DSM_20307_T	021_Rhodococcus_kyotonensis_JCM_23211_T	19.40	[17.2 - 21.8%]	0.22700	0	4.63
010_Rhodococcus_equi_DSM_20307_T	022_Rhodococcus_maanshanensis_DSM_44675_T	20.70	[18.5 - 23.2%]	0.21180	0	0.38
010_Rhodococcus_equi_DSM_20307_T	023_Rhodococcus_marinonascens_NBRC_14363_T	20.20	[18 - 22.6%]	0.21790	0	4.38
010_Rhodococcus_equi_DSM_20307_T	024_Rhodococcus_maris_DSM_43672_T	19.40	[17.2 - 21.8%]	0.22640	0	2.07
010_Rhodococcus_equi_DSM_20307_T	025_Rhodococcus_opacus_DSM_43205_T	20.40	[18.2 - 22.8%]	0.21510	0	1.51
010_Rhodococcus_equi_DSM_20307_T	026_Rhodococcus_phenolicus_DSM_44812_T	20.70	[18.5 - 23.1%]	0.21230	0	0.43
010_Rhodococcus_equi_DSM_20307_T	027_Rhodococcus_pyridinivorans_DSM_44555_T	20.10	[17.9 - 22.5%]	0.21840	0	0.94
010_Rhodococcus_equi_DSM_20307_T	028_Rhodococcus_qingshengii_JCM_15477_T	19.80	[17.6 - 22.2%]	0.22240	0	6.42
010_Rhodococcus_equi_DSM_20307_T	029_Rhodococcus_rhodnii_NBRC_100604_T	20.60	[18.4 - 23%]	0.21310	0	0.88
010_Rhodococcus_equi_DSM_20307_T	030_Rhodococcus_rhodochrous_DSM_43241_T	20.80	[18.5 - 23.2%]	0.21160	0	0.56
010_Rhodococcus_equi_DSM_20307_T	031_Rhodococcus_ruber_DSM_43338_T	20.50	[18.2 - 22.9%]	0.21470	0	1.88
010_Rhodococcus_equi_DSM_20307_T	032_Rhodococcus_rubripertincta_NBRC_101908_T	20.40	[18.2 - 22.8%]	0.21520	0	1.41
010_Rhodococcus_equi_DSM_20307_T	033_Rhodococcus_sputi_NBRC_100414_T	20.20	[18 - 22.7%]	0.21710	0	3.45
010_Rhodococcus_equi_DSM_20307_T	034_Rhodococcus_terrae_NRRL_B_16283_T	20.40	[18.1 - 22.8%]	0.21580	0	0.99
010_Rhodococcus_equi_DSM_20307_T	035_Rhodococcus_triatomae_DSM_44892_T	20.10	[17.9 - 22.5%]	0.21850	0	0.06
010_Rhodococcus_equi_DSM_20307_T	036_Rhodococcus_tukisamuensis_NBRC_100609_T	21.40	[19.1 - 23.8%]	0.20530	0	1.06
010_Rhodococcus_equi_DSM_20307_T	037_Rhodococcus_wratislaviensis_NBRC_100605_T	20.30	[18.1 - 22.8%]	0.21610	0	2.01
010_Rhodococcus_equi_DSM_20307_T	038_Rhodococcus_yunnanensis_NBRC_103083_T	19.20	[17 - 21.6%]	0.22860	0	4.9
010_Rhodococcus_equi_DSM_20307_T	039_Rhodococcus_zopfii_NBRC_100606_T	20.40	[18.2 - 22.8%]	0.21530	0	0.55

011_Rhodococcus_erythropolis_NBRC_15567_T	012_Rhodococcus_fascians_NBRC_12155_T	19.60	[17.4 - 22%]	0.22440	0	2.05
011_Rhodococcus_erythropolis_NBRC_15567_T	013_Rhodococcus_globerulus_NBRC_14531_T	24.10	[21.8 - 26.6%]	0.18130	0.01	0.73
011_Rhodococcus_erythropolis_NBRC_15567_T	014_Rhodococcus_gordoniae_DSM_44689_T	19.70	[17.5 - 22.1%]	0.22310	0	5.55
011_Rhodococcus_erythropolis_NBRC_15567_T	015_Rhodococcus_hoagii_DSM_20295_T	19.80	[17.6 - 22.2%]	0.22170	0	6.37
011_Rhodococcus_erythropolis_NBRC_15567_T	016_Rhodococcus_imtechensis_JCM_13270_T	20.20	[18 - 22.6%]	0.21790	0	4.82
011_Rhodococcus_erythropolis_NBRC_15567_T	017_Rhodococcus_jostii_NBRC_16295_T	20.30	[18.1 - 22.7%]	0.21630	0	4.5
011_Rhodococcus_erythropolis_NBRC_15567_T	018_Rhodococcus_koreensis_DSM_44498_T	20.20	[18 - 22.6%]	0.21760	0	4.98
011_Rhodococcus_erythropolis_NBRC_15567_T	019_Rhodococcus_kroppenstedtii_DSM_44908_T	20.10	[17.9 - 22.5%]	0.21860	0	7.67
011_Rhodococcus_erythropolis_NBRC_15567_T	020_Rhodococcus_kunmingensis_DSM_45001_T	19.70	[17.5 - 22.1%]	0.22290	0	3.8
011_Rhodococcus_erythropolis_NBRC_15567_T	021_Rhodococcus_kyotonensis_JCM_23211_T	19.30	[17.1 - 21.6%]	0.22830	0	1.76
011_Rhodococcus_erythropolis_NBRC_15567_T	022_Rhodococcus_maanshanensis_DSM_44675_T	19.90	[17.7 - 22.3%]	0.22040	0	6.77
011_Rhodococcus_erythropolis_NBRC_15567_T	023_Rhodococcus_marinonascens_NBRC_14363_T	19.60	[17.4 - 22%]	0.22400	0	2.01
011_Rhodococcus_erythropolis_NBRC_15567_T	024_Rhodococcus_maris_DSM_43672_T	19.30	[17.1 - 21.7%]	0.22790	0	8.46
011_Rhodococcus_erythropolis_NBRC_15567_T	025_Rhodococcus_opacus_DSM_43205_T	20.40	[18.2 - 22.9%]	0.21490	0	4.88
011_Rhodococcus_erythropolis_NBRC_15567_T	026_Rhodococcus_phenolicus_DSM_44812_T	19.60	[17.4 - 22%]	0.22480	0	5.96
011_Rhodococcus_erythropolis_NBRC_15567_T	027_Rhodococcus_pyridinivorans_DSM_44555_T	19.40	[17.2 - 21.8%]	0.22640	0	5.45
011_Rhodococcus_erythropolis_NBRC_15567_T	028_Rhodococcus_qingshengii_JCM_15477_T	62.20	[59.3 - 65%]	0.04790	59.38	0.03
011_Rhodococcus_erythropolis_NBRC_15567_T	029_Rhodococcus_rhodnii_NBRC_100604_T	19.80	[17.6 - 22.2%]	0.22240	0	7.27
011_Rhodococcus_erythropolis_NBRC_15567_T	030_Rhodococcus_rhodochrous_DSM_43241_T	19.60	[17.4 - 22%]	0.22470	0	5.83
011_Rhodococcus_erythropolis_NBRC_15567_T	031_Rhodococcus_ruber_DSM_43338_T	19.70	[17.5 - 22.1%]	0.22270	0	8.27
011_Rhodococcus_erythropolis_NBRC_15567_T	032_Rhodococcus_rubripertincta_NBRC_101908_T	20.00	[17.8 - 22.4%]	0.21950	0	4.98
011_Rhodococcus_erythropolis_NBRC_15567_T	033_Rhodococcus_sputi_NBRC_100414_T	19.70	[17.5 - 22.1%]	0.22260	0	2.95
011_Rhodococcus_erythropolis_NBRC_15567_T	034_Rhodococcus_terrae_NRRL_B_16283_T	19.80	[17.6 - 22.2%]	0.22200	0	5.41
011_Rhodococcus_erythropolis_NBRC_15567_T	035_Rhodococcus_triatomae_DSM_44892_T	19.90	[17.7 - 22.3%]	0.22070	0	6.33
011_Rhodococcus_erythropolis_NBRC_15567_T	036_Rhodococcus_tukisamuensis_NBRC_100609_T	20.20	[18 - 22.6%]	0.21770	0	7.45
011_Rhodococcus_erythropolis_NBRC_15567_T	037_Rhodococcus_wratislaviensis_NBRC_100605_T	20.00	[17.8 - 22.4%]	0.21990	0	4.38
011_Rhodococcus_erythropolis_NBRC_15567_T	038_Rhodococcus_yunnanensis_NBRC_103083_T	19.80	[17.6 - 22.2%]	0.22230	0	1.5
011_Rhodococcus_erythropolis_NBRC_15567_T	039_Rhodococcus_zopfii_NBRC_100606_T	19.50	[17.3 - 21.9%]	0.22520	0	5.84
012_Rhodococcus_fascians_NBRC_12155_T	013_Rhodococcus_globerulus_NBRC_14531_T	19.70	[17.5 - 22.1%]	0.22290	0	2.77

012_Rhodococcus_fascians_NBRC_12155_T	014_Rhodococcus_gordoniae_DSM_44689_T	19.10	[16.9 - 21.5%]	0.22980	0	3.5
012_Rhodococcus_fascians_NBRC_12155_T	015_Rhodococcus_hoagii_DSM_20295_T	19.60	[17.4 - 22%]	0.22390	0	4.32
012_Rhodococcus_fascians_NBRC_12155_T	016_Rhodococcus_imtechensis_JCM_13270_T	19.20	[17 - 21.6%]	0.22930	0	2.78
012_Rhodococcus_fascians_NBRC_12155_T	017_Rhodococcus_jostii_NBRC_16295_T	19.40	[17.2 - 21.7%]	0.22720	0	2.46
012_Rhodococcus_fascians_NBRC_12155_T	018_Rhodococcus_koreensis_DSM_44498_T	19.60	[17.4 - 22%]	0.22440	0	2.93
012_Rhodococcus_fascians_NBRC_12155_T	019_Rhodococcus_kroppenstedtii_DSM_44908_T	20.00	[17.8 - 22.4%]	0.22000	0	5.62
012_Rhodococcus_fascians_NBRC_12155_T	020_Rhodococcus_kunmingensis_DSM_45001_T	19.70	[17.5 - 22.1%]	0.22290	0	1.75
012_Rhodococcus_fascians_NBRC_12155_T	021_Rhodococcus_kyotonensis_JCM_23211_T	20.00	[17.8 - 22.4%]	0.22020	0	0.29
012_Rhodococcus_fascians_NBRC_12155_T	022_Rhodococcus_maanshanensis_DSM_44675_T	19.70	[17.5 - 22.1%]	0.22270	0	4.72
012_Rhodococcus_fascians_NBRC_12155_T	023_Rhodococcus_marinonascens_NBRC_14363_T	19.50	[17.3 - 21.9%]	0.22560	0	0.04
012_Rhodococcus_fascians_NBRC_12155_T	024_Rhodococcus_maris_DSM_43672_T	19.70	[17.5 - 22.1%]	0.22290	0	6.41
012_Rhodococcus_fascians_NBRC_12155_T	025_Rhodococcus_opacus_DSM_43205_T	19.50	[17.3 - 21.9%]	0.22540	0	2.83
012_Rhodococcus_fascians_NBRC_12155_T	026_Rhodococcus_phenolicus_DSM_44812_T	20.00	[17.8 - 22.5%]	0.21930	0	3.91
012_Rhodococcus_fascians_NBRC_12155_T	027_Rhodococcus_pyridinivorans_DSM_44555_T	19.50	[17.3 - 21.9%]	0.22600	0	3.4
012_Rhodococcus_fascians_NBRC_12155_T	028_Rhodococcus_qingshengii_JCM_15477_T	19.60	[17.4 - 22%]	0.22460	0	2.08
012_Rhodococcus_fascians_NBRC_12155_T	029_Rhodococcus_rhodnii_NBRC_100604_T	19.70	[17.5 - 22.1%]	0.22270	0	5.23
012_Rhodococcus_fascians_NBRC_12155_T	030_Rhodococcus_rhodochrous_DSM_43241_T	19.70	[17.5 - 22.1%]	0.22360	0	3.79
012_Rhodococcus_fascians_NBRC_12155_T	031_Rhodococcus_ruber_DSM_43338_T	19.20	[17 - 21.5%]	0.22950	0	6.22
012_Rhodococcus_fascians_NBRC_12155_T	032_Rhodococcus_rubripertincta_NBRC_101908_T	19.80	[17.6 - 22.2%]	0.22200	0	2.94
012_Rhodococcus_fascians_NBRC_12155_T	033_Rhodococcus_sputi_NBRC_100414_T	19.80	[17.6 - 22.2%]	0.22170	0	0.9
012_Rhodococcus_fascians_NBRC_12155_T	034_Rhodococcus_terrae_NRRL_B_16283_T	20.20	[18 - 22.6%]	0.21720	0	3.36
012_Rhodococcus_fascians_NBRC_12155_T	035_Rhodococcus_triatomae_DSM_44892_T	19.30	[17.1 - 21.7%]	0.22820	0	4.28
012_Rhodococcus_fascians_NBRC_12155_T	036_Rhodococcus_tukisamuensis_NBRC_100609_T	19.40	[17.2 - 21.8%]	0.22630	0	5.41
012_Rhodococcus_fascians_NBRC_12155_T	037_Rhodococcus_wratislaviensis_NBRC_100605_T	19.50	[17.3 - 21.9%]	0.22570	0	2.33
012_Rhodococcus_fascians_NBRC_12155_T	038_Rhodococcus_yunnanensis_NBRC_103083_T	20.00	[17.8 - 22.4%]	0.21980	0	0.55
012_Rhodococcus_fascians_NBRC_12155_T	039_Rhodococcus_zopfii_NBRC_100606_T	19.10	[16.9 - 21.5%]	0.23010	0	3.8
013_Rhodococcus_globerulus_NBRC_14531_T	014_Rhodococcus_gordoniae_DSM_44689_T	19.30	[17.1 - 21.6%]	0.22830	0	6.28
013_Rhodococcus_globerulus_NBRC_14531_T	015_Rhodococcus_hoagii_DSM_20295_T	19.70	[17.5 - 22.1%]	0.22270	0	7.1
013_Rhodococcus_globerulus_NBRC_14531_T	016_Rhodococcus_imtechensis_JCM_13270_T	19.80	[17.6 - 22.2%]	0.22170	0	5.55

013_Rhodococcus_globerulus_NBRC_14531_T	017_Rhodococcus_jostii_NBRC_16295_T	20.00	[17.8 - 22.4%]	0.22020	0	5.23
013_Rhodococcus_globerulus_NBRC_14531_T	018_Rhodococcus_koreensis_DSM_44498_T	19.60	[17.4 - 22%]	0.22380	0	5.7
013_Rhodococcus_globerulus_NBRC_14531_T	019_Rhodococcus_kroppenstedtii_DSM_44908_T	19.60	[17.4 - 22%]	0.22440	0	8.39
013_Rhodococcus_globerulus_NBRC_14531_T	020_Rhodococcus_kunmingensis_DSM_45001_T	20.40	[18.2 - 22.8%]	0.21570	0	4.52
013_Rhodococcus_globerulus_NBRC_14531_T	021_Rhodococcus_kyotonensis_JCM_23211_T	19.00	[16.9 - 21.4%]	0.23090	0	2.49
013_Rhodococcus_globerulus_NBRC_14531_T	022_Rhodococcus_maanshanensis_DSM_44675_T	20.40	[18.1 - 22.8%]	0.21580	0	7.5
013_Rhodococcus_globerulus_NBRC_14531_T	023_Rhodococcus_marinonascens_NBRC_14363_T	19.70	[17.5 - 22.1%]	0.22360	0	2.74
013_Rhodococcus_globerulus_NBRC_14531_T	024_Rhodococcus_maris_DSM_43672_T	19.50	[17.3 - 21.9%]	0.22530	0	9.19
013_Rhodococcus_globerulus_NBRC_14531_T	025_Rhodococcus_opacus_DSM_43205_T	19.90	[17.7 - 22.3%]	0.22070	0	5.61
013_Rhodococcus_globerulus_NBRC_14531_T	026_Rhodococcus_phenolicus_DSM_44812_T	19.00	[16.8 - 21.4%]	0.23110	0	6.69
013_Rhodococcus_globerulus_NBRC_14531_T	027_Rhodococcus_pyridinivorans_DSM_44555_T	19.70	[17.5 - 22.1%]	0.22290	0	6.18
013_Rhodococcus_globerulus_NBRC_14531_T	028_Rhodococcus_qingshengii_JCM_15477_T	24.20	[21.9 - 26.7%]	0.18030	0.01	0.7
013_Rhodococcus_globerulus_NBRC_14531_T	029_Rhodococcus_rhodnii_NBRC_100604_T	20.00	[17.8 - 22.4%]	0.21950	0	8
013_Rhodococcus_globerulus_NBRC_14531_T	030_Rhodococcus_rhodochrous_DSM_43241_T	19.70	[17.5 - 22.1%]	0.22370	0	6.56
013_Rhodococcus_globerulus_NBRC_14531_T	031_Rhodococcus_ruber_DSM_43338_T	19.30	[17.1 - 21.7%]	0.22810	0	9
013_Rhodococcus_globerulus_NBRC_14531_T	032_Rhodococcus_rubripertincta_NBRC_101908_T	19.70	[17.5 - 22.1%]	0.22260	0	5.71
013_Rhodococcus_globerulus_NBRC_14531_T	033_Rhodococcus_sputi_NBRC_100414_T	20.00	[17.8 - 22.5%]	0.21930	0	3.67
013_Rhodococcus_globerulus_NBRC_14531_T	034_Rhodococcus_terrae_NRRL_B_16283_T	20.00	[17.8 - 22.4%]	0.21950	0	6.13
013_Rhodococcus_globerulus_NBRC_14531_T	035_Rhodococcus_triatomae_DSM_44892_T	19.50	[17.3 - 21.9%]	0.22540	0	7.06
013_Rhodococcus_globerulus_NBRC_14531_T	036_Rhodococcus_tukisamuensis_NBRC_100609_T	20.30	[18.1 - 22.7%]	0.21650	0	8.18
013_Rhodococcus_globerulus_NBRC_14531_T	037_Rhodococcus_wratislaviensis_NBRC_100605_T	19.80	[17.6 - 22.2%]	0.22220	0	5.11
013_Rhodococcus_globerulus_NBRC_14531_T	038_Rhodococcus_yunnanensis_NBRC_103083_T	20.10	[17.9 - 22.5%]	0.21900	0	2.22
013_Rhodococcus_globerulus_NBRC_14531_T	039_Rhodococcus_zopfii_NBRC_100606_T	19.70	[17.5 - 22.1%]	0.22360	0	6.57
014_Rhodococcus_gordoniae_DSM_44689_T	015_Rhodococcus_hoagii_DSM_20295_T	20.60	[18.3 - 23%]	0.21370	0	0.82
014_Rhodococcus_gordoniae_DSM_44689_T	016_Rhodococcus_imtechensis_JCM_13270_T	19.70	[17.5 - 22.1%]	0.22330	0	0.73
014_Rhodococcus_gordoniae_DSM_44689_T	017_Rhodococcus_jostii_NBRC_16295_T	19.90	[17.7 - 22.3%]	0.22090	0	1.05
014_Rhodococcus_gordoniae_DSM_44689_T	018_Rhodococcus_koreensis_DSM_44498_T	19.80	[17.6 - 22.2%]	0.22200	0	0.57
014_Rhodococcus_gordoniae_DSM_44689_T	019_Rhodococcus_kroppenstedtii_DSM_44908_T	19.60	[17.4 - 22%]	0.22410	0	2.12
014_Rhodococcus_gordoniae_DSM_44689_T	020_Rhodococcus_kunmingensis_DSM_45001_T	19.90	[17.7 - 22.3%]	0.22140	0	1.75

014_Rhodococcus_gordoniae_DSM_44689_T	021_Rhodococcus_kyotonensis_JCM_23211_T	19.10	[17 - 21.5%]	0.22980	0	3.79
014_Rhodococcus_gordoniae_DSM_44689_T	022_Rhodococcus_maanshanensis_DSM_44675_T	19.90	[17.7 - 22.3%]	0.22130	0	1.22
014_Rhodococcus_gordoniae_DSM_44689_T	023_Rhodococcus_marinonascens_NBRC_14363_T	19.40	[17.2 - 21.8%]	0.22660	0	3.54
014_Rhodococcus_gordoniae_DSM_44689_T	024_Rhodococcus_maris_DSM_43672_T	19.50	[17.3 - 21.9%]	0.22580	0	2.91
014_Rhodococcus_gordoniae_DSM_44689_T	025_Rhodococcus_opacus_DSM_43205_T	19.70	[17.5 - 22.1%]	0.22350	0	0.67
014_Rhodococcus_gordoniae_DSM_44689_T	026_Rhodococcus_phenolicus_DSM_44812_T	22.00	[19.7 - 24.5%]	0.19930	0	0.41
014_Rhodococcus_gordoniae_DSM_44689_T	027_Rhodococcus_pyridinivorans_DSM_44555_T	43.10	[40.6 - 45.7%]	0.08970	5.58	0.1
014_Rhodococcus_gordoniae_DSM_44689_T	028_Rhodococcus_qingshengii_JCM_15477_T	19.50	[17.3 - 21.9%]	0.22560	0	5.58
014_Rhodococcus_gordoniae_DSM_44689_T	029_Rhodococcus_rhodnii_NBRC_100604_T	20.00	[17.8 - 22.4%]	0.21950	0	1.73
014_Rhodococcus_gordoniae_DSM_44689_T	030_Rhodococcus_rhodochrous_DSM_43241_T	41.70	[39.2 - 44.3%]	0.09380	4.12	0.28
014_Rhodococcus_gordoniae_DSM_44689_T	031_Rhodococcus_ruber_DSM_43338_T	21.40	[19.1 - 23.8%]	0.20560	0	2.72
014_Rhodococcus_gordoniae_DSM_44689_T	032_Rhodococcus_rubripertincta_NBRC_101908_T	20.00	[17.8 - 22.4%]	0.22000	0	0.56
014_Rhodococcus_gordoniae_DSM_44689_T	033_Rhodococcus_sputi_NBRC_100414_T	20.10	[17.9 - 22.6%]	0.21820	0	2.6
014_Rhodococcus_gordoniae_DSM_44689_T	034_Rhodococcus_terrae_NRRL_B_16283_T	20.00	[17.8 - 22.4%]	0.22000	0	0.14
014_Rhodococcus_gordoniae_DSM_44689_T	035_Rhodococcus_triatomae_DSM_44892_T	20.50	[18.3 - 22.9%]	0.21420	0	0.78
014_Rhodococcus_gordoniae_DSM_44689_T	036_Rhodococcus_tukisamuensis_NBRC_100609_T	20.40	[18.1 - 22.8%]	0.21580	0	1.9
014_Rhodococcus_gordoniae_DSM_44689_T	037_Rhodococcus_wratislaviensis_NBRC_100605_T	19.90	[17.7 - 22.3%]	0.22110	0	1.17
014_Rhodococcus_gordoniae_DSM_44689_T	038_Rhodococcus_yunnanensis_NBRC_103083_T	19.40	[17.3 - 21.8%]	0.22610	0	4.05
014_Rhodococcus_gordoniae_DSM_44689_T	039_Rhodococcus_zopfii_NBRC_100606_T	21.90	[19.6 - 24.3%]	0.20080	0	0.3
015_Rhodococcus_hoagii_DSM_20295_T	016_Rhodococcus_imtechensis_JCM_13270_T	20.50	[18.3 - 22.9%]	0.21410	0	1.55
015_Rhodococcus_hoagii_DSM_20295_T	017_Rhodococcus_jostii_NBRC_16295_T	20.30	[18.1 - 22.8%]	0.21600	0	1.87
015_Rhodococcus_hoagii_DSM_20295_T	018_Rhodococcus_koreensis_DSM_44498_T	20.50	[18.3 - 22.9%]	0.21460	0	1.39
015_Rhodococcus_hoagii_DSM_20295_T	019_Rhodococcus_kroppenstedtii_DSM_44908_T	20.30	[18.1 - 22.7%]	0.21660	0	1.3
015_Rhodococcus_hoagii_DSM_20295_T	020_Rhodococcus_kunmingensis_DSM_45001_T	20.40	[18.1 - 22.8%]	0.21590	0	2.57
015_Rhodococcus_hoagii_DSM_20295_T	021_Rhodococcus_kyotonensis_JCM_23211_T	19.50	[17.3 - 21.8%]	0.22600	0	4.61
015_Rhodococcus_hoagii_DSM_20295_T	022_Rhodococcus_maanshanensis_DSM_44675_T	20.90	[18.7 - 23.4%]	0.20960	0	0.4
015_Rhodococcus_hoagii_DSM_20295_T	023_Rhodococcus_marinonascens_NBRC_14363_T	20.20	[18 - 22.6%]	0.21750	0	4.36
015_Rhodococcus_hoagii_DSM_20295_T	024_Rhodococcus_maris_DSM_43672_T	19.50	[17.3 - 21.8%]	0.22600	0	2.09
015_Rhodococcus_hoagii_DSM_20295_T	025_Rhodococcus_opacus_DSM_43205_T	20.70	[18.5 - 23.1%]	0.21220	0	1.49

015_Rhodococcus_hoagii_DSM_20295_T	026_Rhodococcus_phenolicus_DSM_44812_T	21.10	[18.9 - 23.5%]	0.20800	0	0.41
015_Rhodococcus_hoagii_DSM_20295_T	027_Rhodococcus_pyridinivorans_DSM_44555_T	20.40	[18.2 - 22.8%]	0.21520	0	0.92
015_Rhodococcus_hoagii_DSM_20295_T	028_Rhodococcus_qingshengii_JCM_15477_T	19.70	[17.5 - 22.1%]	0.22300	0	6.4
015_Rhodococcus_hoagii_DSM_20295_T	029_Rhodococcus_rhodnii_NBRC_100604_T	20.50	[18.3 - 22.9%]	0.21420	0	0.91
015_Rhodococcus_hoagii_DSM_20295_T	030_Rhodococcus_rhodochrous_DSM_43241_T	21.00	[18.8 - 23.4%]	0.20900	0	0.54
015_Rhodococcus_hoagii_DSM_20295_T	031_Rhodococcus_ruber_DSM_43338_T	20.70	[18.4 - 23.1%]	0.21260	0	1.9
015_Rhodococcus_hoagii_DSM_20295_T	032_Rhodococcus_rubripertincta_NBRC_101908_T	20.60	[18.4 - 23%]	0.21330	0	1.38
015_Rhodococcus_hoagii_DSM_20295_T	033_Rhodococcus_sputi_NBRC_100414_T	20.40	[18.2 - 22.8%]	0.21550	0	3.42
015_Rhodococcus_hoagii_DSM_20295_T	034_Rhodococcus_terrae_NRRL_B_16283_T	20.60	[18.4 - 23%]	0.21320	0	0.96
015_Rhodococcus_hoagii_DSM_20295_T	035_Rhodococcus_triatomae_DSM_44892_T	20.20	[17.9 - 22.6%]	0.21800	0	0.04
015_Rhodococcus_hoagii_DSM_20295_T	036_Rhodococcus_tukisamuensis_NBRC_100609_T	21.50	[19.2 - 23.9%]	0.20450	0	1.08
015_Rhodococcus_hoagii_DSM_20295_T	037_Rhodococcus_wratislaviensis_NBRC_100605_T	20.50	[18.3 - 22.9%]	0.21400	0	1.99
015_Rhodococcus_hoagii_DSM_20295_T	038_Rhodococcus_yunnanensis_NBRC_103083_T	19.40	[17.2 - 21.8%]	0.22620	0	4.87
015_Rhodococcus_hoagii_DSM_20295_T	039_Rhodococcus_zopfii_NBRC_100606_T	20.70	[18.4 - 23.1%]	0.21260	0	0.52
016_Rhodococcus_imtechensis_JCM_13270_T	017_Rhodococcus_jostii_NBRC_16295_T	41.60	[39.1 - 44.2%]	0.09410	4.03	0.32
016_Rhodococcus_imtechensis_JCM_13270_T	018_Rhodococcus_koreensis_DSM_44498_T	44.30	[41.8 - 46.9%]	0.08610	7.23	0.15
016_Rhodococcus_imtechensis_JCM_13270_T	019_Rhodococcus_kroppenstedtii_DSM_44908_T	19.70	[17.5 - 22.1%]	0.22290	0	2.84
016_Rhodococcus_imtechensis_JCM_13270_T	020_Rhodococcus_kunmingensis_DSM_45001_T	19.90	[17.7 - 22.3%]	0.22140	0	1.03
016_Rhodococcus_imtechensis_JCM_13270_T	021_Rhodococcus_kyotonensis_JCM_23211_T	19.40	[17.2 - 21.8%]	0.22620	0	3.06
016_Rhodococcus_imtechensis_JCM_13270_T	022_Rhodococcus_maanshanensis_DSM_44675_T	20.60	[18.4 - 23%]	0.21350	0	1.95
016_Rhodococcus_imtechensis_JCM_13270_T	023_Rhodococcus_marinonascens_NBRC_14363_T	27.10	[24.7 - 29.5%]	0.15970	0.03	2.81
016_Rhodococcus_imtechensis_JCM_13270_T	024_Rhodococcus_maris_DSM_43672_T	19.30	[17.1 - 21.7%]	0.22740	0	3.64
016_Rhodococcus_imtechensis_JCM_13270_T	025_Rhodococcus_opacus_DSM_43205_T	81.20	[78.3 - 83.8%]	0.02190	91.47	0.06
016_Rhodococcus_imtechensis_JCM_13270_T	026_Rhodococcus_phenolicus_DSM_44812_T	20.00	[17.8 - 22.5%]	0.21930	0	1.14
016_Rhodococcus_imtechensis_JCM_13270_T	027_Rhodococcus_pyridinivorans_DSM_44555_T	20.00	[17.8 - 22.4%]	0.21960	0	0.63
016_Rhodococcus_imtechensis_JCM_13270_T	028_Rhodococcus_qingshengii_JCM_15477_T	20.20	[18 - 22.6%]	0.21750	0	4.85
016_Rhodococcus_imtechensis_JCM_13270_T	029_Rhodococcus_rhodnii_NBRC_100604_T	19.70	[17.5 - 22.1%]	0.22280	0	2.45
016_Rhodococcus_imtechensis_JCM_13270_T	030_Rhodococcus_rhodochrous_DSM_43241_T	19.90	[17.7 - 22.3%]	0.22090	0	1.01
016_Rhodococcus_imtechensis_JCM_13270_T	031_Rhodococcus_ruber_DSM_43338_T	20.40	[18.2 - 22.9%]	0.21500	0	3.44

016_Rhodococcus_imtechensis_JCM_13270_T	032_Rhodococcus_rubripertincta_NBRC_101908_T	19.50	[17.3 - 21.9%]	0.22540	0	0.16
016_Rhodococcus_imtechensis_JCM_13270_T	033_Rhodococcus_sputi_NBRC_100414_T	20.30	[18 - 22.7%]	0.21700	0	1.88
016_Rhodococcus_imtechensis_JCM_13270_T	034_Rhodococcus_terrae_NRRL_B_16283_T	19.90	[17.7 - 22.3%]	0.22060	0	0.58
016_Rhodococcus_imtechensis_JCM_13270_T	035_Rhodococcus_triatomae_DSM_44892_T	20.80	[18.5 - 23.2%]	0.21150	0	1.51
016_Rhodococcus_imtechensis_JCM_13270_T	036_Rhodococcus_tukisamuensis_NBRC_100609_T	20.90	[18.7 - 23.3%]	0.20990	0	2.63
016_Rhodococcus_imtechensis_JCM_13270_T	037_Rhodococcus_wratislaviensis_NBRC_100605_T	57.40	[54.6 - 60.1%]	0.05630	43.39	0.44
016_Rhodococcus_imtechensis_JCM_13270_T	038_Rhodococcus_yunnanensis_NBRC_103083_T	19.20	[17 - 21.6%]	0.22930	0	3.33
016_Rhodococcus_imtechensis_JCM_13270_T	039_Rhodococcus_zopfii_NBRC_100606_T	20.10	[17.9 - 22.5%]	0.21840	0	1.02
017_Rhodococcus_jostii_NBRC_16295_T	018_Rhodococcus_koreensis_DSM_44498_T	38.80	[36.4 - 41.4%]	0.10340	2.02	0.47
017_Rhodococcus_jostii_NBRC_16295_T	019_Rhodococcus_kroppenstedtii_DSM_44908_T	19.70	[17.5 - 22.1%]	0.22340	0	3.16
017_Rhodococcus_jostii_NBRC_16295_T	020_Rhodococcus_kunmingensis_DSM_45001_T	20.00	[17.8 - 22.4%]	0.22020	0	0.71
017_Rhodococcus_jostii_NBRC_16295_T	021_Rhodococcus_kyotonensis_JCM_23211_T	19.10	[16.9 - 21.5%]	0.23040	0	2.74
017_Rhodococcus_jostii_NBRC_16295_T	022_Rhodococcus_maanshanensis_DSM_44675_T	20.60	[18.4 - 23.1%]	0.21280	0	2.27
017_Rhodococcus_jostii_NBRC_16295_T	023_Rhodococcus_marinonascens_NBRC_14363_T	26.90	[24.6 - 29.4%]	0.16070	0.03	2.49
017_Rhodococcus_jostii_NBRC_16295_T	024_Rhodococcus_maris_DSM_43672_T	19.20	[17.1 - 21.6%]	0.22850	0	3.96
017_Rhodococcus_jostii_NBRC_16295_T	025_Rhodococcus_opacus_DSM_43205_T	42.50	[40 - 45.1%]	0.09140	4.94	0.38
017_Rhodococcus_jostii_NBRC_16295_T	026_Rhodococcus_phenolicus_DSM_44812_T	20.10	[17.9 - 22.6%]	0.21820	0	1.46
017_Rhodococcus_jostii_NBRC_16295_T	027_Rhodococcus_pyridinivorans_DSM_44555_T	19.70	[17.5 - 22.1%]	0.22320	0	0.95
017_Rhodococcus_jostii_NBRC_16295_T	028_Rhodococcus_qingshengii_JCM_15477_T	20.20	[18 - 22.6%]	0.21720	0	4.53
017_Rhodococcus_jostii_NBRC_16295_T	029_Rhodococcus_rhodnii_NBRC_100604_T	19.50	[17.3 - 21.9%]	0.22490	0	2.77
017_Rhodococcus_jostii_NBRC_16295_T	030_Rhodococcus_rhodochrous_DSM_43241_T	19.80	[17.6 - 22.2%]	0.22230	0	1.33
017_Rhodococcus_jostii_NBRC_16295_T	031_Rhodococcus_ruber_DSM_43338_T	20.30	[18.1 - 22.7%]	0.21620	0	3.77
017_Rhodococcus_jostii_NBRC_16295_T	032_Rhodococcus_rubripertincta_NBRC_101908_T	19.50	[17.3 - 21.9%]	0.22580	0	0.48
017_Rhodococcus_jostii_NBRC_16295_T	033_Rhodococcus_sputi_NBRC_100414_T	19.60	[17.4 - 22%]	0.22410	0	1.56
017_Rhodococcus_jostii_NBRC_16295_T	034_Rhodococcus_terrae_NRRL_B_16283_T	19.50	[17.3 - 21.9%]	0.22500	0	0.9
017_Rhodococcus_jostii_NBRC_16295_T	035_Rhodococcus_triatomae_DSM_44892_T	20.50	[18.2 - 22.9%]	0.21470	0	1.83
017_Rhodococcus_jostii_NBRC_16295_T	036_Rhodococcus_tukisamuensis_NBRC_100609_T	20.80	[18.6 - 23.3%]	0.21080	0	2.95
017_Rhodococcus_jostii_NBRC_16295_T	037_Rhodococcus_wratislaviensis_NBRC_100605_T	41.90	[39.4 - 44.5%]	0.09320	4.3	0.12
017_Rhodococcus_jostii_NBRC_16295_T	038_Rhodococcus_yunnanensis_NBRC_103083_T	19.40	[17.2 - 21.8%]	0.22660	0	3.01

017_Rhodococcus_jostii_NBRC_16295_T	039_Rhodococcus_zopfii_NBRC_100606_T	20.10	[17.9 - 22.5%]	0.21880	0	1.34
018_Rhodococcus_koreensis_DSM_44498_T	019_Rhodococcus_kroppenstedtii_DSM_44908_T	19.80	[17.6 - 22.2%]	0.22180	0.00	2.69
018_Rhodococcus_koreensis_DSM_44498_T	020_Rhodococcus_kunmingensis_DSM_45001_T	20.10	[17.9 - 22.6%]	0.21810	0.00	1.18
018_Rhodococcus_koreensis_DSM_44498_T	021_Rhodococcus_kyotonensis_JCM_23211_T	19.20	[17 - 21.6%]	0.22910	0.00	3.22
018_Rhodococcus_koreensis_DSM_44498_T	022_Rhodococcus_maanshanensis_DSM_44675_T	20.60	[18.4 - 23%]	0.21340	0.00	1.79
018_Rhodococcus_koreensis_DSM_44498_T	023_Rhodococcus_marinonascens_NBRC_14363_T	26.60	[24.2 - 29.1%]	0.16300	0.02	2.97
018_Rhodococcus_koreensis_DSM_44498_T	024_Rhodococcus_maris_DSM_43672_T	19.40	[17.2 - 21.8%]	0.22620	0.00	3.48
018_Rhodococcus_koreensis_DSM_44498_T	025_Rhodococcus_opacus_DSM_43205_T	44.00	[41.4 - 46.5%]	0.08710	6.7	0.1
018_Rhodococcus_koreensis_DSM_44498_T	026_Rhodococcus_phenolicus_DSM_44812_T	20.10	[17.9 - 22.5%]	0.21830	0.00	0.98
018_Rhodococcus_koreensis_DSM_44498_T	027_Rhodococcus_pyridinivorans_DSM_44555_T	19.80	[17.6 - 22.2%]	0.22180	0.00	0.47
018_Rhodococcus_koreensis_DSM_44498_T	028_Rhodococcus_qingshengii_JCM_15477_T	20.40	[18.2 - 22.8%]	0.21550	0.00	5.01
018_Rhodococcus_koreensis_DSM_44498_T	029_Rhodococcus_rhodnii_NBRC_100604_T	19.80	[17.6 - 22.2%]	0.22170	0.00	2.3
018_Rhodococcus_koreensis_DSM_44498_T	030_Rhodococcus_rhodochrous_DSM_43241_T	20.00	[17.8 - 22.4%]	0.21950	0.00	0.85
018_Rhodococcus_koreensis_DSM_44498_T	031_Rhodococcus_ruber_DSM_43338_T	20.60	[18.4 - 23%]	0.21310	0.00	3.29
018_Rhodococcus_koreensis_DSM_44498_T	032_Rhodococcus_rubripertincta_NBRC_101908_T	19.70	[17.5 - 22.1%]	0.22340	0.00	0.01
018_Rhodococcus_koreensis_DSM_44498_T	033_Rhodococcus_sputi_NBRC_100414_T	20.60	[18.4 - 23.1%]	0.21290	0.00	2.03
018_Rhodococcus_koreensis_DSM_44498_T	034_Rhodococcus_terrae_NRRL_B_16283_T	19.70	[17.5 - 22.1%]	0.22310	0.00	0.43
018_Rhodococcus_koreensis_DSM_44498_T	035_Rhodococcus_triatomae_DSM_44892_T	20.30	[18.1 - 22.8%]	0.21600	0.00	1.35
018_Rhodococcus_koreensis_DSM_44498_T	036_Rhodococcus_tukisamuensis_NBRC_100609_T	20.80	[18.6 - 23.2%]	0.21100	0.00	2.48
018_Rhodococcus_koreensis_DSM_44498_T	037_Rhodococcus_wratislaviensis_NBRC_100605_T	43.30	[40.7 - 45.8%]	0.08920	5.77	0.6
018_Rhodococcus_koreensis_DSM_44498_T	038_Rhodococcus_yunnanensis_NBRC_103083_T	19.50	[17.3 - 21.9%]	0.22590	0.00	3.48
018_Rhodococcus_koreensis_DSM_44498_T	039_Rhodococcus_zopfii_NBRC_100606_T	20.10	[17.9 - 22.5%]	0.21870	0.00	0.87
019_Rhodococcus_kroppenstedtii_DSM_44908_T	020_Rhodococcus_kunmingensis_DSM_45001_T	19.80	[17.6 - 22.2%]	0.22250	0.00	3.87
019_Rhodococcus_kroppenstedtii_DSM_44908_T	021_Rhodococcus_kyotonensis_JCM_23211_T	19.90	[17.7 - 22.3%]	0.22080	0.00	5.91
019_Rhodococcus_kroppenstedtii_DSM_44908_T	022_Rhodococcus_maanshanensis_DSM_44675_T	20.00	[17.8 - 22.5%]	0.21930	0.00	0.9
019_Rhodococcus_kroppenstedtii_DSM_44908_T	023_Rhodococcus_marinonascens_NBRC_14363_T	20.10	[17.9 - 22.5%]	0.21900	0.00	5.66
019_Rhodococcus_kroppenstedtii_DSM_44908_T	024_Rhodococcus_maris_DSM_43672_T	18.80	[16.6 - 21.2%]	0.23390	0.00	0.79
019_Rhodococcus_kroppenstedtii_DSM_44908_T	025_Rhodococcus_opacus_DSM_43205_T	19.70	[17.5 - 22.1%]	0.22290	0.00	2.79
019_Rhodococcus_kroppenstedtii_DSM_44908_T	026_Rhodococcus_phenolicus_DSM_44812_T	19.40	[17.2 - 21.8%]	0.22620	0.00	1.71

019_Rhodococcus_kroppenstedtii_DSM_44908_T	027_Rhodococcus_pyridinivorans_DSM_44555_T	20.20	[18 - 22.6%]	0.21760	0.00	2.22
019_Rhodococcus_kroppenstedtii_DSM_44908_T	028_Rhodococcus_qingshengii_JCM_15477_T	20.30	[18.1 - 22.7%]	0.21620	0.00	7.7
019_Rhodococcus_kroppenstedtii_DSM_44908_T	029_Rhodococcus_rhodnii_NBRC_100604_T	19.70	[17.5 - 22.1%]	0.22350	0.00	0.39
019_Rhodococcus_kroppenstedtii_DSM_44908_T	030_Rhodococcus_rhodochrous_DSM_43241_T	20.00	[17.8 - 22.4%]	0.21980	0.00	1.83
019_Rhodococcus_kroppenstedtii_DSM_44908_T	031_Rhodococcus_ruber_DSM_43338_T	19.80	[17.6 - 22.2%]	0.22150	0.00	0.6
019_Rhodococcus_kroppenstedtii_DSM_44908_T	032_Rhodococcus_rubripertincta_NBRC_101908_T	19.80	[17.6 - 22.2%]	0.22220	0.00	2.68
019_Rhodococcus_kroppenstedtii_DSM_44908_T	033_Rhodococcus_sputi_NBRC_100414_T	19.70	[17.5 - 22.1%]	0.22280	0.00	4.72
019_Rhodococcus_kroppenstedtii_DSM_44908_T	034_Rhodococcus_terrae_NRRL_B_16283_T	20.20	[17.9 - 22.6%]	0.21800	0.00	2.26
019_Rhodococcus_kroppenstedtii_DSM_44908_T	035_Rhodococcus_triatomae_DSM_44892_T	20.10	[17.8 - 22.5%]	0.21920	0.00	1.34
019_Rhodococcus_kroppenstedtii_DSM_44908_T	036_Rhodococcus_tukisamuensis_NBRC_100609_T	20.00	[17.8 - 22.4%]	0.21940	0.00	0.21
019_Rhodococcus_kroppenstedtii_DSM_44908_T	037_Rhodococcus_wratislaviensis_NBRC_100605_T	19.60	[17.4 - 22%]	0.22450	0.00	3.29
019_Rhodococcus_kroppenstedtii_DSM_44908_T	038_Rhodococcus_yunnanensis_NBRC_103083_T	19.90	[17.7 - 22.3%]	0.22120	0.00	6.17
019_Rhodococcus_kroppenstedtii_DSM_44908_T	039_Rhodococcus_zopfii_NBRC_100606_T	19.40	[17.2 - 21.8%]	0.22640	0.00	1.82
020_Rhodococcus_kunmingensis_DSM_45001_T	021_Rhodococcus_kyotonensis_JCM_23211_T	19.40	[17.2 - 21.8%]	0.22680	0.00	2.04
020_Rhodococcus_kunmingensis_DSM_45001_T	022_Rhodococcus_maanshanensis_DSM_44675_T	19.90	[17.7 - 22.4%]	0.22030	0.00	2.97
020_Rhodococcus_kunmingensis_DSM_45001_T	023_Rhodococcus_marinonascens_NBRC_14363_T	19.00	[16.9 - 21.4%]	0.23080	0.00	1.79
020_Rhodococcus_kunmingensis_DSM_45001_T	024_Rhodococcus_maris_DSM_43672_T	19.30	[17.2 - 21.7%]	0.22730	0.00	4.66
020_Rhodococcus_kunmingensis_DSM_45001_T	025_Rhodococcus_opacus_DSM_43205_T	19.70	[17.5 - 22.1%]	0.22280	0.00	1.08
020_Rhodococcus_kunmingensis_DSM_45001_T	026_Rhodococcus_phenolicus_DSM_44812_T	19.40	[17.2 - 21.8%]	0.22660	0.00	2.16
020_Rhodococcus_kunmingensis_DSM_45001_T	027_Rhodococcus_pyridinivorans_DSM_44555_T	19.50	[17.3 - 21.9%]	0.22550	0.00	1.65
020_Rhodococcus_kunmingensis_DSM_45001_T	028_Rhodococcus_qingshengii_JCM_15477_T	19.70	[17.5 - 22.1%]	0.22260	0.00	3.83
020_Rhodococcus_kunmingensis_DSM_45001_T	029_Rhodococcus_rhodnii_NBRC_100604_T	20.30	[18.1 - 22.8%]	0.21600	0.00	3.48
020_Rhodococcus_kunmingensis_DSM_45001_T	030_Rhodococcus_rhodochrous_DSM_43241_T	19.80	[17.6 - 22.2%]	0.22240	0.00	2.04
020_Rhodococcus_kunmingensis_DSM_45001_T	031_Rhodococcus_ruber_DSM_43338_T	20.00	[17.8 - 22.4%]	0.21990	0.00	4.47
020_Rhodococcus_kunmingensis_DSM_45001_T	032_Rhodococcus_rubripertincta_NBRC_101908_T	19.90	[17.7 - 22.4%]	0.22040	0.00	1.19
020_Rhodococcus_kunmingensis_DSM_45001_T	033_Rhodococcus_sputi_NBRC_100414_T	20.10	[17.9 - 22.5%]	0.21870	0.00	0.85
020_Rhodococcus_kunmingensis_DSM_45001_T	034_Rhodococcus_terrae_NRRL_B_16283_T	20.10	[17.9 - 22.5%]	0.21880	0.00	1.61
020_Rhodococcus_kunmingensis_DSM_45001_T	035_Rhodococcus_triatomae_DSM_44892_T	20.30	[18.1 - 22.7%]	0.21660	0.00	2.53
020_Rhodococcus_kunmingensis_DSM_45001_T	036_Rhodococcus_tukisamuensis_NBRC_100609_T	20.00	[17.8 - 22.4%]	0.21950	0.00	3.66

020_Rhodococcus_kunmingensis_DSM_45001_T	037_Rhodococcus_wratislaviensis_NBRC_100605_T	19.70	[17.5 - 22.1%]	0.22280	0.00	0.58
020_Rhodococcus_kunmingensis_DSM_45001_T	038_Rhodococcus_yunnanensis_NBRC_103083_T	20.00	[17.8 - 22.4%]	0.21970	0.00	2.3
020_Rhodococcus_kunmingensis_DSM_45001_T	039_Rhodococcus_zopfii_NBRC_100606_T	19.90	[17.7 - 22.3%]	0.22110	0.00	2.05
021_Rhodococcus_kyotonensis_JCM_23211_T	022_Rhodococcus_maanshanensis_DSM_44675_T	18.90	[16.8 - 21.3%]	0.23210	0.00	5.01
021_Rhodococcus_kyotonensis_JCM_23211_T	023_Rhodococcus_marinonascens_NBRC_14363_T	19.30	[17.1 - 21.7%]	0.22790	0.00	0.25
021_Rhodococcus_kyotonensis_JCM_23211_T	024_Rhodococcus_maris_DSM_43672_T	19.50	[17.3 - 21.9%]	0.22550	0.00	6.7
021_Rhodococcus_kyotonensis_JCM_23211_T	025_Rhodococcus_opacus_DSM_43205_T	19.40	[17.2 - 21.8%]	0.22690	0.00	3.12
021_Rhodococcus_kyotonensis_JCM_23211_T	026_Rhodococcus_phenolicus_DSM_44812_T	19.30	[17.2 - 21.7%]	0.22730	0.00	4.2
021_Rhodococcus_kyotonensis_JCM_23211_T	027_Rhodococcus_pyridinivorans_DSM_44555_T	18.90	[16.7 - 21.3%]	0.23240	0.00	3.69
021_Rhodococcus_kyotonensis_JCM_23211_T	028_Rhodococcus_qingshengii_JCM_15477_T	19.50	[17.3 - 21.9%]	0.22590	0.00	1.79
021_Rhodococcus_kyotonensis_JCM_23211_T	029_Rhodococcus_rhodnii_NBRC_100604_T	19.00	[16.8 - 21.4%]	0.23150	0.00	5.52
021_Rhodococcus_kyotonensis_JCM_23211_T	030_Rhodococcus_rhodochrous_DSM_43241_T	19.30	[17.1 - 21.7%]	0.22800	0.00	4.07
021_Rhodococcus_kyotonensis_JCM_23211_T	031_Rhodococcus_ruber_DSM_43338_T	18.90	[16.8 - 21.3%]	0.23230	0.00	6.51
021_Rhodococcus_kyotonensis_JCM_23211_T	032_Rhodococcus_rubripertincta_NBRC_101908_T	20.10	[17.9 - 22.5%]	0.21870	0.00	3.23
021_Rhodococcus_kyotonensis_JCM_23211_T	033_Rhodococcus_sputi_NBRC_100414_T	19.80	[17.6 - 22.2%]	0.22240	0.00	1.19
021_Rhodococcus_kyotonensis_JCM_23211_T	034_Rhodococcus_terrae_NRRL_B_16283_T	19.50	[17.3 - 21.9%]	0.22550	0.00	3.65
021_Rhodococcus_kyotonensis_JCM_23211_T	035_Rhodococcus_triatomae_DSM_44892_T	19.50	[17.4 - 21.9%]	0.22490	0.00	4.57
021_Rhodococcus_kyotonensis_JCM_23211_T	036_Rhodococcus_tukisamuensis_NBRC_100609_T	19.60	[17.4 - 22%]	0.22370	0.00	5.69
021_Rhodococcus_kyotonensis_JCM_23211_T	037_Rhodococcus_wratislaviensis_NBRC_100605_T	19.20	[17 - 21.6%]	0.22910	0.00	2.62
021_Rhodococcus_kyotonensis_JCM_23211_T	038_Rhodococcus_yunnanensis_NBRC_103083_T	22.60	[20.3 - 25%]	0.19390	0.00	0.26
021_Rhodococcus_kyotonensis_JCM_23211_T	039_Rhodococcus_zopfii_NBRC_100606_T	19.00	[16.8 - 21.3%]	0.23190	0.00	4.09
022_Rhodococcus_maanshanensis_DSM_44675_T	023_Rhodococcus_marinonascens_NBRC_14363_T	20.10	[17.9 - 22.5%]	0.21850	0.00	4.76
022_Rhodococcus_maanshanensis_DSM_44675_T	024_Rhodococcus_maris_DSM_43672_T	19.60	[17.4 - 22%]	0.22450	0.00	1.69
022_Rhodococcus_maanshanensis_DSM_44675_T	025_Rhodococcus_opacus_DSM_43205_T	20.60	[18.4 - 23%]	0.21310	0.00	1.89
022_Rhodococcus_maanshanensis_DSM_44675_T	026_Rhodococcus_phenolicus_DSM_44812_T	20.30	[18 - 22.7%]	0.21680	0.00	0.81
022_Rhodococcus_maanshanensis_DSM_44675_T	027_Rhodococcus_pyridinivorans_DSM_44555_T	19.60	[17.4 - 22%]	0.22390	0.00	1.32
022_Rhodococcus_maanshanensis_DSM_44675_T	028_Rhodococcus_qingshengii_JCM_15477_T	19.90	[17.7 - 22.3%]	0.22050	0.00	6.8
022_Rhodococcus_maanshanensis_DSM_44675_T	029_Rhodococcus_rhodnii_NBRC_100604_T	19.90	[17.7 - 22.3%]	0.22110	0.00	0.51
022_Rhodococcus_maanshanensis_DSM_44675_T	030_Rhodococcus_rhodochrous_DSM_43241_T	20.30	[18.1 - 22.7%]	0.21680	0.00	0.94

022_Rhodococcus_maanshanensis_DSM_44675_T	031_Rhodococcus_ruber_DSM_43338_T	20.30	[18.1 - 22.7%]	0.21640	0.00	1.5
022_Rhodococcus_maanshanensis_DSM_44675_T	032_Rhodococcus_rubripertincta_NBRC_101908_T	19.90	[17.7 - 22.3%]	0.22140	0.00	1.78
022_Rhodococcus_maanshanensis_DSM_44675_T	033_Rhodococcus_sputi_NBRC_100414_T	20.00	[17.8 - 22.4%]	0.21970	0.00	3.82
022_Rhodococcus_maanshanensis_DSM_44675_T	034_Rhodococcus_terrae_NRRL_B_16283_T	19.90	[17.7 - 22.3%]	0.22100	0.00	1.36
022_Rhodococcus_maanshanensis_DSM_44675_T	035_Rhodococcus_triatomae_DSM_44892_T	20.40	[18.2 - 22.8%]	0.21510	0.00	0.44
022_Rhodococcus_maanshanensis_DSM_44675_T	036_Rhodococcus_tukisamuensis_NBRC_100609_T	24.20	[21.9 - 26.6%]	0.18060	0.01	0.68
022_Rhodococcus_maanshanensis_DSM_44675_T	037_Rhodococcus_wratislaviensis_NBRC_100605_T	20.50	[18.3 - 22.9%]	0.21440	0.00	2.39
022_Rhodococcus_maanshanensis_DSM_44675_T	038_Rhodococcus_yunnanensis_NBRC_103083_T	19.10	[16.9 - 21.5%]	0.23040	0.00	5.27
022_Rhodococcus_maanshanensis_DSM_44675_T	039_Rhodococcus_zopfii_NBRC_100606_T	20.20	[18 - 22.6%]	0.21740	0.00	0.92
023_Rhodococcus_marinonascens_NBRC_14363_T	024_Rhodococcus_maris_DSM_43672_T	19.30	[17.1 - 21.7%]	0.22820	0.00	6.45
023_Rhodococcus_marinonascens_NBRC_14363_T	025_Rhodococcus_opacus_DSM_43205_T	27.20	[24.8 - 29.7%]	0.15900	0.03	2.87
023_Rhodococcus_marinonascens_NBRC_14363_T	026_Rhodococcus_phenolicus_DSM_44812_T	19.60	[17.4 - 22%]	0.22410	0.00	3.95
023_Rhodococcus_marinonascens_NBRC_14363_T	027_Rhodococcus_pyridinivorans_DSM_44555_T	20.00	[17.8 - 22.4%]	0.22000	0.00	3.44
023_Rhodococcus_marinonascens_NBRC_14363_T	028_Rhodococcus_qingshengii_JCM_15477_T	20.00	[17.8 - 22.4%]	0.22010	0.00	2.04
023_Rhodococcus_marinonascens_NBRC_14363_T	029_Rhodococcus_rhodnii_NBRC_100604_T	19.80	[17.6 - 22.2%]	0.22150	0.00	5.26
023_Rhodococcus_marinonascens_NBRC_14363_T	030_Rhodococcus_rhodochrous_DSM_43241_T	19.90	[17.7 - 22.3%]	0.22130	0.00	3.82
023_Rhodococcus_marinonascens_NBRC_14363_T	031_Rhodococcus_ruber_DSM_43338_T	19.80	[17.6 - 22.2%]	0.22250	0.00	6.26
023_Rhodococcus_marinonascens_NBRC_14363_T	032_Rhodococcus_rubripertincta_NBRC_101908_T	19.60	[17.5 - 22%]	0.22370	0.00	2.97
023_Rhodococcus_marinonascens_NBRC_14363_T	033_Rhodococcus_sputi_NBRC_100414_T	20.60	[18.4 - 23%]	0.21340	0.00	0.94
023_Rhodococcus_marinonascens_NBRC_14363_T	034_Rhodococcus_terrae_NRRL_B_16283_T	19.90	[17.7 - 22.3%]	0.22100	0.00	3.4
023_Rhodococcus_marinonascens_NBRC_14363_T	035_Rhodococcus_triatomae_DSM_44892_T	20.20	[17.9 - 22.6%]	0.21800	0.00	4.32
023_Rhodococcus_marinonascens_NBRC_14363_T	036_Rhodococcus_tukisamuensis_NBRC_100609_T	20.10	[17.9 - 22.5%]	0.21890	0.00	5.44
023_Rhodococcus_marinonascens_NBRC_14363_T	037_Rhodococcus_wratislaviensis_NBRC_100605_T	27.20	[24.9 - 29.7%]	0.15860	0.03	2.37
023_Rhodococcus_marinonascens_NBRC_14363_T	038_Rhodococcus_yunnanensis_NBRC_103083_T	19.60	[17.4 - 22%]	0.22420	0.00	0.51
023_Rhodococcus_marinonascens_NBRC_14363_T	039_Rhodococcus_zopfii_NBRC_100606_T	19.50	[17.4 - 21.9%]	0.22490	0.00	3.83
024_Rhodococcus_maris_DSM_43672_T	025_Rhodococcus_opacus_DSM_43205_T	19.80	[17.6 - 22.2%]	0.22160	0.00	3.58
024_Rhodococcus_maris_DSM_43672_T	026_Rhodococcus_phenolicus_DSM_44812_T	19.40	[17.2 - 21.8%]	0.22630	0.00	2.5
024_Rhodococcus_maris_DSM_43672_T	027_Rhodococcus_pyridinivorans_DSM_44555_T	19.80	[17.6 - 22.2%]	0.22190	0.00	3.01
024_Rhodococcus_maris_DSM_43672_T	028_Rhodococcus_qingshengii_JCM_15477_T	19.60	[17.4 - 22%]	0.22420	0.00	8.49

024_Rhodococcus_maris_DSM_43672_T	029_Rhodococcus_rhodnii_NBRC_100604_T	19.20	[17 - 21.6%]	0.22930	0.00	1.18
024_Rhodococcus_maris_DSM_43672_T	030_Rhodococcus_rhodochrous_DSM_43241_T	19.60	[17.4 - 21.9%]	0.22480	0.00	2.63
024_Rhodococcus_maris_DSM_43672_T	031_Rhodococcus_ruber_DSM_43338_T	19.70	[17.5 - 22.1%]	0.22350	0.00	0.19
024_Rhodococcus_maris_DSM_43672_T	032_Rhodococcus_rubripertincta_NBRC_101908_T	20.40	[18.1 - 22.8%]	0.21580	0.00	3.47
024_Rhodococcus_maris_DSM_43672_T	033_Rhodococcus_sputi_NBRC_100414_T	20.30	[18.1 - 22.7%]	0.21630	0.00	5.51
024_Rhodococcus_maris_DSM_43672_T	034_Rhodococcus_terrae_NRRL_B_16283_T	19.50	[17.3 - 21.9%]	0.22580	0.00	3.05
024_Rhodococcus_maris_DSM_43672_T	035_Rhodococcus_triatomae_DSM_44892_T	19.40	[17.2 - 21.8%]	0.22680	0.00	2.13
024_Rhodococcus_maris_DSM_43672_T	036_Rhodococcus_tukisamuensis_NBRC_100609_T	19.40	[17.2 - 21.8%]	0.22630	0.00	1.01
024_Rhodococcus_maris_DSM_43672_T	037_Rhodococcus_wratislaviensis_NBRC_100605_T	19.90	[17.7 - 22.3%]	0.22080	0.00	4.08
024_Rhodococcus_maris_DSM_43672_T	038_Rhodococcus_yunnanensis_NBRC_103083_T	19.20	[17 - 21.6%]	0.22920	0.00	6.96
024_Rhodococcus_maris_DSM_43672_T	039_Rhodococcus_zopfii_NBRC_100606_T	19.50	[17.4 - 21.9%]	0.22490	0.00	2.61
025_Rhodococcus_opacus_DSM_43205_T	026_Rhodococcus_phenolicus_DSM_44812_T	20.20	[18 - 22.6%]	0.21740	0.00	1.08
025_Rhodococcus_opacus_DSM_43205_T	027_Rhodococcus_pyridinivorans_DSM_44555_T	20.10	[17.9 - 22.5%]	0.21850	0.00	0.57
025_Rhodococcus_opacus_DSM_43205_T	028_Rhodococcus_qingshengii_JCM_15477_T	20.40	[18.2 - 22.8%]	0.21560	0.00	4.91
025_Rhodococcus_opacus_DSM_43205_T	029_Rhodococcus_rhodnii_NBRC_100604_T	19.80	[17.6 - 22.2%]	0.22240	0.00	2.39
025_Rhodococcus_opacus_DSM_43205_T	030_Rhodococcus_rhodochrous_DSM_43241_T	20.10	[17.9 - 22.5%]	0.21870	0.00	0.95
025_Rhodococcus_opacus_DSM_43205_T	031_Rhodococcus_ruber_DSM_43338_T	20.50	[18.3 - 22.9%]	0.21460	0.00	3.39
025_Rhodococcus_opacus_DSM_43205_T	032_Rhodococcus_rubripertincta_NBRC_101908_T	19.70	[17.5 - 22.1%]	0.22340	0.00	0.1
025_Rhodococcus_opacus_DSM_43205_T	033_Rhodococcus_sputi_NBRC_100414_T	20.40	[18.2 - 22.8%]	0.21570	0.00	1.93
025_Rhodococcus_opacus_DSM_43205_T	034_Rhodococcus_terrae_NRRL_B_16283_T	20.10	[17.8 - 22.5%]	0.21920	0.00	0.53
025_Rhodococcus_opacus_DSM_43205_T	035_Rhodococcus_triatomae_DSM_44892_T	20.50	[18.3 - 22.9%]	0.21440	0.00	1.45
025_Rhodococcus_opacus_DSM_43205_T	036_Rhodococcus_tukisamuensis_NBRC_100609_T	20.80	[18.6 - 23.3%]	0.21080	0.00	2.57
025_Rhodococcus_opacus_DSM_43205_T	037_Rhodococcus_wratislaviensis_NBRC_100605_T	58.20	[55.4 - 60.9%]	0.05490	46.13	0.5
025_Rhodococcus_opacus_DSM_43205_T	038_Rhodococcus_yunnanensis_NBRC_103083_T	19.30	[17.1 - 21.7%]	0.22790	0.00	3.38
025_Rhodococcus_opacus_DSM_43205_T	039_Rhodococcus_zopfii_NBRC_100606_T	20.30	[18.1 - 22.8%]	0.21600	0.00	0.96
026_Rhodococcus_phenolicus_DSM_44812_T	027_Rhodococcus_pyridinivorans_DSM_44555_T	21.90	[19.7 - 24.4%]	0.19980	0.00	0.51
026_Rhodococcus_phenolicus_DSM_44812_T	028_Rhodococcus_qingshengii_JCM_15477_T	19.70	[17.5 - 22.1%]	0.22290	0.00	5.99
026_Rhodococcus_phenolicus_DSM_44812_T	029_Rhodococcus_rhodnii_NBRC_100604_T	19.70	[17.5 - 22.1%]	0.22280	0.00	1.31
026_Rhodococcus_phenolicus_DSM_44812_T	030_Rhodococcus_rhodochrous_DSM_43241_T	22.00	[19.7 - 24.5%]	0.19930	0.00	0.13

026_Rhodococcus_phenolicus_DSM_44812_T	031_Rhodococcus_ruber_DSM_43338_T	22.60	[20.4 - 25.1%]	0.19360	0.00	2.31
026_Rhodococcus_phenolicus_DSM_44812_T	032_Rhodococcus_rubripertincta_NBRC_101908_T	20.10	[17.9 - 22.5%]	0.21850	0.00	0.98
026_Rhodococcus_phenolicus_DSM_44812_T	033_Rhodococcus_sputi_NBRC_100414_T	20.10	[17.9 - 22.5%]	0.21850	0.00	3.01
026_Rhodococcus_phenolicus_DSM_44812_T	034_Rhodococcus_terrae_NRRL_B_16283_T	19.90	[17.7 - 22.4%]	0.22040	0.00	0.55
026_Rhodococcus_phenolicus_DSM_44812_T	035_Rhodococcus_triatomae_DSM_44892_T	20.10	[17.9 - 22.5%]	0.21890	0.00	0.37
026_Rhodococcus_phenolicus_DSM_44812_T	036_Rhodococcus_tukisamuensis_NBRC_100609_T	20.60	[18.3 - 23%]	0.21360	0.00	1.49
026_Rhodococcus_phenolicus_DSM_44812_T	037_Rhodococcus_wratislaviensis_NBRC_100605_T	20.10	[17.9 - 22.5%]	0.21880	0.00	1.58
026_Rhodococcus_phenolicus_DSM_44812_T	038_Rhodococcus_yunnanensis_NBRC_103083_T	19.70	[17.5 - 22.1%]	0.22340	0.00	4.46
026_Rhodococcus_phenolicus_DSM_44812_T	039_Rhodococcus_zopfii_NBRC_100606_T	36.40	[34 - 39%]	0.11230	1.03	0.12
027_Rhodococcus_pyridinivorans_DSM_44555_T	028_Rhodococcus_qingshengii_JCM_15477_T	19.90	[17.7 - 22.3%]	0.22070	0.00	5.48
027_Rhodococcus_pyridinivorans_DSM_44555_T	029_Rhodococcus_rhodnii_NBRC_100604_T	20.00	[17.8 - 22.4%]	0.21950	0.00	1.83
027_Rhodococcus_pyridinivorans_DSM_44555_T	030_Rhodococcus_rhodochrous_DSM_43241_T	58.60	[55.8 - 61.4%]	0.05410	47.65	0.38
027_Rhodococcus_pyridinivorans_DSM_44555_T	031_Rhodococcus_ruber_DSM_43338_T	21.50	[19.3 - 24%]	0.20380	0.00	2.82
027_Rhodococcus_pyridinivorans_DSM_44555_T	032_Rhodococcus_rubripertincta_NBRC_101908_T	20.30	[18 - 22.7%]	0.21700	0.00	0.46
027_Rhodococcus_pyridinivorans_DSM_44555_T	033_Rhodococcus_sputi_NBRC_100414_T	21.50	[19.2 - 23.9%]	0.20420	0.00	2.5
027_Rhodococcus_pyridinivorans_DSM_44555_T	034_Rhodococcus_terrae_NRRL_B_16283_T	20.00	[17.8 - 22.5%]	0.21930	0.00	0.04
027_Rhodococcus_pyridinivorans_DSM_44555_T	035_Rhodococcus_triatomae_DSM_44892_T	20.20	[18 - 22.7%]	0.21710	0.00	0.88
027_Rhodococcus_pyridinivorans_DSM_44555_T	036_Rhodococcus_tukisamuensis_NBRC_100609_T	20.00	[17.8 - 22.4%]	0.21970	0.00	2
027_Rhodococcus_pyridinivorans_DSM_44555_T	037_Rhodococcus_wratislaviensis_NBRC_100605_T	19.90	[17.7 - 22.3%]	0.22140	0.00	1.07
027_Rhodococcus_pyridinivorans_DSM_44555_T	038_Rhodococcus_yunnanensis_NBRC_103083_T	19.20	[17 - 21.6%]	0.22940	0.00	3.95
027_Rhodococcus_pyridinivorans_DSM_44555_T	039_Rhodococcus_zopfii_NBRC_100606_T	22.10	[19.8 - 24.5%]	0.19870	0.00	0.4
028_Rhodococcus_qingshengii_JCM_15477_T	029_Rhodococcus_rhodnii_NBRC_100604_T	19.90	[17.7 - 22.3%]	0.22110	0.00	7.3
028_Rhodococcus_qingshengii_JCM_15477_T	030_Rhodococcus_rhodochrous_DSM_43241_T	19.60	[17.4 - 22%]	0.22440	0.00	5.86
028_Rhodococcus_qingshengii_JCM_15477_T	031_Rhodococcus_ruber_DSM_43338_T	19.80	[17.6 - 22.2%]	0.22220	0.00	8.3
028_Rhodococcus_qingshengii_JCM_15477_T	032_Rhodococcus_rubripertincta_NBRC_101908_T	20.00	[17.8 - 22.5%]	0.21920	0.00	5.01
028_Rhodococcus_qingshengii_JCM_15477_T	033_Rhodococcus_sputi_NBRC_100414_T	20.30	[18.1 - 22.7%]	0.21660	0.00	2.98
028_Rhodococcus_qingshengii_JCM_15477_T	034_Rhodococcus_terrae_NRRL_B_16283_T	20.10	[17.9 - 22.5%]	0.21820	0.00	5.44
028_Rhodococcus_qingshengii_JCM_15477_T	035_Rhodococcus_triatomae_DSM_44892_T	19.90	[17.7 - 22.3%]	0.22100	0.00	6.36
028_Rhodococcus_qingshengii_JCM_15477_T	036_Rhodococcus_tukisamuensis_NBRC_100609_T	20.10	[17.9 - 22.6%]	0.21820	0.00	7.48

028_Rhodococcus_qingshengii_JCM_15477_T	037_Rhodococcus_wratislaviensis_NBRC_100605_T	20.20	[18 - 22.6%]	0.21760	0.00	4.41
028_Rhodococcus_qingshengii_JCM_15477_T	038_Rhodococcus_yunnanensis_NBRC_103083_T	19.90	[17.7 - 22.3%]	0.22140	0.00	1.53
028_Rhodococcus_qingshengii_JCM_15477_T	039_Rhodococcus_zopfii_NBRC_100606_T	19.40	[17.3 - 21.8%]	0.22610	0.00	5.87
029_Rhodococcus_rhodnii_NBRC_100604_T	030_Rhodococcus_rhodochrous_DSM_43241_T	20.00	[17.8 - 22.4%]	0.21980	0.00	1.44
029_Rhodococcus_rhodnii_NBRC_100604_T	031_Rhodococcus_ruber_DSM_43338_T	19.80	[17.6 - 22.2%]	0.22150	0.00	0.99
029_Rhodococcus_rhodnii_NBRC_100604_T	032_Rhodococcus_rubripertincta_NBRC_101908_T	19.80	[17.6 - 22.2%]	0.22200	0.00	2.29
029_Rhodococcus_rhodnii_NBRC_100604_T	033_Rhodococcus_sputi_NBRC_100414_T	20.30	[18.1 - 22.7%]	0.21660	0.00	4.33
029_Rhodococcus_rhodnii_NBRC_100604_T	034_Rhodococcus_terrae_NRRL_B_16283_T	19.70	[17.5 - 22.1%]	0.22260	0.00	1.87
029_Rhodococcus_rhodnii_NBRC_100604_T	035_Rhodococcus_triatomae_DSM_44892_T	20.50	[18.2 - 22.9%]	0.21480	0.00	0.94
029_Rhodococcus_rhodnii_NBRC_100604_T	036_Rhodococcus_tukisamuensis_NBRC_100609_T	20.00	[17.7 - 22.4%]	0.22030	0.00	0.18
029_Rhodococcus_rhodnii_NBRC_100604_T	037_Rhodococcus_wratislaviensis_NBRC_100605_T	19.90	[17.7 - 22.3%]	0.22100	0.00	2.9
029_Rhodococcus_rhodnii_NBRC_100604_T	038_Rhodococcus_yunnanensis_NBRC_103083_T	19.40	[17.2 - 21.8%]	0.22650	0.00	5.78
029_Rhodococcus_rhodnii_NBRC_100604_T	039_Rhodococcus_zopfii_NBRC_100606_T	19.70	[17.5 - 22.1%]	0.22310	0.00	1.43
030_Rhodococcus_rhodochrous_DSM_43241_T	031_Rhodococcus_ruber_DSM_43338_T	21.70	[19.4 - 24.1%]	0.20260	0.00	2.44
030_Rhodococcus_rhodochrous_DSM_43241_T	032_Rhodococcus_rubripertincta_NBRC_101908_T	20.00	[17.8 - 22.5%]	0.21920	0.00	0.85
030_Rhodococcus_rhodochrous_DSM_43241_T	033_Rhodococcus_sputi_NBRC_100414_T	20.20	[18 - 22.6%]	0.21770	0.00	2.89
030_Rhodococcus_rhodochrous_DSM_43241_T	034_Rhodococcus_terrae_NRRL_B_16283_T	20.10	[17.9 - 22.5%]	0.21840	0.00	0.43
030_Rhodococcus_rhodochrous_DSM_43241_T	035_Rhodococcus_triatomae_DSM_44892_T	20.30	[18.1 - 22.7%]	0.21640	0.00	0.5
030_Rhodococcus_rhodochrous_DSM_43241_T	036_Rhodococcus_tukisamuensis_NBRC_100609_T	20.50	[18.2 - 22.9%]	0.21470	0.00	1.62
030_Rhodococcus_rhodochrous_DSM_43241_T	037_Rhodococcus_wratislaviensis_NBRC_100605_T	19.80	[17.6 - 22.2%]	0.22170	0.00	1.45
030_Rhodococcus_rhodochrous_DSM_43241_T	038_Rhodococcus_yunnanensis_NBRC_103083_T	19.30	[17.2 - 21.7%]	0.22730	0.00	4.33
030_Rhodococcus_rhodochrous_DSM_43241_T	039_Rhodococcus_zopfii_NBRC_100606_T	21.90	[19.6 - 24.3%]	0.20060	0.00	0.01
031_Rhodococcus_ruber_DSM_43338_T	032_Rhodococcus_rubripertincta_NBRC_101908_T	20.10	[17.9 - 22.5%]	0.21830	0.00	3.28
031_Rhodococcus_ruber_DSM_43338_T	033_Rhodococcus_sputi_NBRC_100414_T	20.60	[18.4 - 23%]	0.21330	0.00	5.32
031_Rhodococcus_ruber_DSM_43338_T	034_Rhodococcus_terrae_NRRL_B_16283_T	19.70	[17.5 - 22.1%]	0.22270	0.00	2.86
031_Rhodococcus_ruber_DSM_43338_T	035_Rhodococcus_triatomae_DSM_44892_T	20.10	[17.9 - 22.5%]	0.21860	0.00	1.94
031_Rhodococcus_ruber_DSM_43338_T	036_Rhodococcus_tukisamuensis_NBRC_100609_T	20.60	[18.4 - 23.1%]	0.21280	0.00	0.82
031_Rhodococcus_ruber_DSM_43338_T	037_Rhodococcus_wratislaviensis_NBRC_100605_T	20.50	[18.3 - 22.9%]	0.21450	0.00	3.89
031_Rhodococcus_ruber_DSM_43338_T	038_Rhodococcus_yunnanensis_NBRC_103083_T	19.20	[17 - 21.5%]	0.22950	0.00	6.77

031_Rhodococcus_ruber_DSM_43338_T	039_Rhodococcus_zopfii_NBRC_100606_T	22.80	[20.5 - 25.3%]	0.19190	0.00	2.42
032_Rhodococcus_rubripertincta_NBRC_101908_T	033_Rhodococcus_sputi_NBRC_100414_T	20.80	[18.6 - 23.2%]	0.21100	0.00	2.04
032_Rhodococcus_rubripertincta_NBRC_101908_T	034_Rhodococcus_terrae_NRRL_B_16283_T	23.80	[21.5 - 26.3%]	0.18360	0.00	0.42
032_Rhodococcus_rubripertincta_NBRC_101908_T	035_Rhodococcus_triatomae_DSM_44892_T	19.90	[17.7 - 22.3%]	0.22090	0.00	1.35
032_Rhodococcus_rubripertincta_NBRC_101908_T	036_Rhodococcus_tukisamuensis_NBRC_100609_T	19.70	[17.5 - 22.1%]	0.22320	0.00	2.47
032_Rhodococcus_rubripertincta_NBRC_101908_T	037_Rhodococcus_wratislaviensis_NBRC_100605_T	19.60	[17.4 - 22%]	0.22460	0.00	0.61
032_Rhodococcus_rubripertincta_NBRC_101908_T	038_Rhodococcus_yunnanensis_NBRC_103083_T	19.30	[17.1 - 21.7%]	0.22790	0.00	3.49
032_Rhodococcus_rubripertincta_NBRC_101908_T	039_Rhodococcus_zopfii_NBRC_100606_T	20.00	[17.8 - 22.4%]	0.21950	0.00	0.86
033_Rhodococcus_sputi_NBRC_100414_T	034_Rhodococcus_terrae_NRRL_B_16283_T	21.10	[18.8 - 23.5%]	0.20830	0.00	2.46
033_Rhodococcus_sputi_NBRC_100414_T	035_Rhodococcus_triatomae_DSM_44892_T	20.20	[18 - 22.6%]	0.21750	0.00	3.38
033_Rhodococcus_sputi_NBRC_100414_T	036_Rhodococcus_tukisamuensis_NBRC_100609_T	20.20	[18 - 22.6%]	0.21740	0.00	4.51
033_Rhodococcus_sputi_NBRC_100414_T	037_Rhodococcus_wratislaviensis_NBRC_100605_T	20.30	[18 - 22.7%]	0.21700	0.00	1.43
033_Rhodococcus_sputi_NBRC_100414_T	038_Rhodococcus_yunnanensis_NBRC_103083_T	19.30	[17.1 - 21.7%]	0.22750	0.00	1.45
033_Rhodococcus_sputi_NBRC_100414_T	039_Rhodococcus_zopfii_NBRC_100606_T	20.60	[18.4 - 23.1%]	0.21290	0.00	2.9
034_Rhodococcus_terrae_NRRL_B_16283_T	035_Rhodococcus_triatomae_DSM_44892_T	20.00	[17.8 - 22.4%]	0.21990	0.00	0.92
034_Rhodococcus_terrae_NRRL_B_16283_T	036_Rhodococcus_tukisamuensis_NBRC_100609_T	19.80	[17.6 - 22.2%]	0.22210	0.00	2.05
034_Rhodococcus_terrae_NRRL_B_16283_T	037_Rhodococcus_wratislaviensis_NBRC_100605_T	19.70	[17.5 - 22.1%]	0.22320	0.00	1.03
034_Rhodococcus_terrae_NRRL_B_16283_T	038_Rhodococcus_yunnanensis_NBRC_103083_T	19.70	[17.5 - 22.1%]	0.22370	0.00	3.91
034_Rhodococcus_terrae_NRRL_B_16283_T	039_Rhodococcus_zopfii_NBRC_100606_T	19.60	[17.4 - 22%]	0.22440	0.00	0.44
035_Rhodococcus_triatomae_DSM_44892_T	036_Rhodococcus_tukisamuensis_NBRC_100609_T	20.20	[18 - 22.6%]	0.21710	0.00	1.12
035_Rhodococcus_triatomae_DSM_44892_T	037_Rhodococcus_wratislaviensis_NBRC_100605_T	20.50	[18.3 - 22.9%]	0.21450	0.00	1.95
035_Rhodococcus_triatomae_DSM_44892_T	038_Rhodococcus_yunnanensis_NBRC_103083_T	19.40	[17.2 - 21.8%]	0.22710	0.00	4.83
035_Rhodococcus_triatomae_DSM_44892_T	039_Rhodococcus_zopfii_NBRC_100606_T	20.20	[18 - 22.6%]	0.21770	0.00	0.48
036_Rhodococcus_tukisamuensis_NBRC_100609_T	037_Rhodococcus_wratislaviensis_NBRC_100605_T	20.90	[18.7 - 23.4%]	0.20970	0.00	3.07
036_Rhodococcus_tukisamuensis_NBRC_100609_T	038_Rhodococcus_yunnanensis_NBRC_103083_T	19.70	[17.5 - 22.1%]	0.22350	0.00	5.96
036_Rhodococcus_tukisamuensis_NBRC_100609_T	039_Rhodococcus_zopfii_NBRC_100606_T	20.50	[18.3 - 22.9%]	0.21420	0.00	1.61
037_Rhodococcus_wratislaviensis_NBRC_100605_T	038_Rhodococcus_yunnanensis_NBRC_103083_T	19.30	[17.1 - 21.7%]	0.22740	0.00	2.88
037_Rhodococcus_wratislaviensis_NBRC_100605_T	039_Rhodococcus_zopfii_NBRC_100606_T	19.90	[17.7 - 22.4%]	0.22040	0.00	1.47
038_Rhodococcus_yunnanensis_NBRC_103083_T	039_Rhodococcus_zopfii_NBRC_100606_T	19.40	[17.2 - 21.8%]	0.22650	0.00	4.35

Table S5. Genes involved in the central metabolism of *Rhodococcus* sp. WAY2

Replicon	Start	End	Strand	Gene name	Predicted function	Function, pathway
Chromosome	654,458	655,462	+	*glkA*	Glucokinase	Glycolysis
Chromosome	2,747,596	2,748,597	+	*glk*	Glucokinase	Glycolysis
Chromosome	4,167,043	4,167,567	+	*gntk*	Glucokinase thermoresistant	Glycolysis
Chromosome	6,343,896	6,343,144	-	*ppgK*	Polyphosphate glucokinase	Glycolysis
Chromosome	227,909	229,564	+	*pgi1*	Glucose-6-phosphate isomerase	Glycolysis, gluconeogenesis, pentose phosphate pathway
Chromosome	5,115,499	5,114,198	-	*pgi2*	Glucose-6-phosphate isomerase	Glycolysis, gluconeogenesis, pentose phosphate pathway
Chromosome	6,102,346	6,103,377	+	*pfkA*	6-phosphofructokinase	Glycolysis
Chromosome	6,297,174	6,296,194	-	*fruK*	1-phosphofructokinase	Glycolysis
Chromosome	5,083,954	5,084,988	+	*fba*	Fructose-bisphosphate aldolase	Glycolysis, gluconeogenesis
Chromosome	3,474,588	3,473,803	-	*tpiA*	Triose-phosphate isomerase	Glycolysis
Chromosome	3,476,836	3,475,817	-	*gap*	Glyceraldehyde 3-phosphate dehydrogenase	Glycolysis, gluconeogenesis
Chromosome	3,475,745	3,474,588	-	*pgk*	Phosphoglycerate kinase	Glycolysis, gluconeogenesis
Chromosome	1,728,074	1,728,829	+	*gpmA1*	Phosphoglycerate mutase	Glycolysis, gluconeogenesis
Chromosome	5,975,937	5,975,323	-	*gpmA2*	Phosphoglycerate mutase	Glycolysis, gluconeogenesis
Chromosome	5,345,257	5,346,543	+	*eno*	Phosphopyruvate hydratase / enolase	Glycolysis, gluconeogenesis
Chromosome	2,865,568	2,866,986	+	*pyk*	Pyruvate kinase	Glycolysis, purine metabolism
Chromosome	4,159,399	4,161,252	+	*edd*	Phosphogluconate dehydratase	Glycolysis (ED)
Chromosome	4,161,265	4,161,891	+	*eda*	4-hydroxy-2-oxoglutarate aldolase	Glycolysis (ED)
Chromosome	5,389,671	5,388,631	-	*glpX*	Fructose-1,6-biphosphatase class II	Gluconeogenesis
pRWAY01	292,410	293,354	+	*glpX*	Fructose-1,6-bisphosphatase	Gluconeogenesis
Chromosome	4,553,850	4,555,679	+	*pckG*	Phosphoenolpyruvate carboxykinase	Gluconeogenesis
prWAY02	385,109	383,205	-	*pckG*	Phosphoenolpyruvate carboxykinase	Gluconeogenesis
Chromosome	241,101	238,750	-	*pdh*	Pyruvate dehydrogenase E1 component	Acetyl-CoA synthesis
Chromosome	2,029,413	2,030,396	+	*pdhA1*	Pyruvate dehydrogenase E1 component, alpha subunit	Acetyl-CoA synthesis
Chromosome	2,295,245	2,294,148	-	*pdhA2*	Pyruvate dehydrogenase E1 component, alpha subunit	Acetyl-CoA synthesis
Chromosome	3,673,818	3,672,730	-	*pdhA3*	Pyruvate dehydrogenase E1 component, alpha subunit	Acetyl-CoA synthesis
Chromosome	2,030,393	2,031,412	+	*pdhB1*	Branched-chain alpha-keto acid dehydrogenase, E1 component, beta subunit	Acetyl-CoA synthesis
Chromosome	2,294,151	2,293,168	-	*pdhB2*	Pyruvate dehydrogenase E1 component beta subunit	Acetyl-CoA synthesis
Chromosome	3,672,733	3,671,741	-	*pdhB3*	Pyruvate dehydrogenase E1 component beta subunit	Acetyl-CoA synthesis

Chromosome	2,031,440	2,032,741	+	*pdhC1*	Dihydrolipoamide acyltransferase component of branched-chain alpha-keto acid dehydrogenase complex	Acetyl-CoA synthesis
Chromosome	2,293,124	2,291,898	-	*pdhC2*	Dihydrolipoamide acetyltransferase component of pyruvate dehydrogenase complex	Acetyl-CoA synthesis
Chromosome	3,671,719	3,670,391	-	*pdhC3*	Dihydrolipoamide acetyltransferase component of pyruvate dehydrogenase complex	Acetyl-CoA synthesis
Chromosome	3,744,757	3,743,195	-	*pdhC4*	Dihydrolipoamide acetyltransferase component of pyruvate dehydrogenase complex	Acetyl-CoA synthesis
Chromosome	2,670,866	2,668,014	-	*aceE1*	Pyruvate dehydrogenase E1 component [a]	Acetyl-CoA synthesis
Chromosome	3,153,897	3,151,561	-	*aceE2*	Pyruvate dehydrogenase E1 component [a]	Acetyl-CoA synthesis
Chromosome	1,458,004	1,456,874	-	*citA*	Citrate synthase (si)	TCA cycle
pRWAY01	707,924	709,246	+	*citA*	Citrate synthase (si)	TCA cycle
Chromosome	1,461,733	1,462,974	+	*gltA*	Citrate synthase (si)	TCA cycle
Chromosome	3,439,278	3,442,082	+	*acnA*	Aconitate hydratase	TCA cycle
Chromosome	1,484,368	1,486,602	+	*icd1*	Isocitrate dehydrogenase (NADP+)	TCA cycle
Chromosome	5,857,767	5,858,990	+	*icd2*	Isocitrate dehydrogenase (NADP+)	TCA cycle
Chromosome	5,591,157	5,587,378	-	*sucA*	2-Oxoglutarate dehydrogenase, E1 and E2 components	TCA cycle
Chromosome	5,124,137	5,125,306	+	*sucC*	Succinyl-CoA ligase (ADP-forming) beta chain	TCA cycle
Chromosome	5,125,323	5,126,225	+	*sucD*	Succinyl-CoA ligase (ADP-forming) alpha chain	TCA cycle
Chromosome	2,824,678	2,826,609	+	*sdhA1*	Succinate dehydrogenase flavoprotein subunit	TCA cycle
Chromosome	5,870,475	5,868,724	-	*sdhA2*	Succinate dehydrogenase flavoprotein subunit	TCA cycle
Chromosome	2,826,611	2,827,360	+	*sdhB1*	Succinate dehydrogenase iron-sulfur protein	TCA cycle
Chromosome	5,868,724	5,867,945	-	*sdhB2*	Succinate dehydrogenase iron-sulfur protein	TCA cycle
Chromosome	5,495,768	5,494,071	-	*fumB*	Fumarate hydratase class I	TCA cycle
Chromosome	6,208,259	6,209,806	+	*mqo*	Malate dehydrogenase (quinone oxidoreductase)	TCA cycle
Chromosome	1,941,117	1,942,310	+	*mdh1*	Malate dehydrogenase	TCA cycle
Chromosome	2,149,115	2,150,170	+	*mdh2*	Malate dehydrogenase	TCA cycle
Chromosome	4,840,775	4,841,710	+	*mdh3*	Malate dehydrogenase	TCA cycle
Chromosome	5,577,218	5,578,411	+	*mdh4*	Malate dehydrogenase	TCA cycle
pRWAY01	27,337	28,536	+	*mdh1*	Malate dehydrogenase	TCA cycle
pRWAY01	632,113	630,920	-	*mdh2*	Malate dehydrogenase	TCA cycle
Chromosome	3,467,019	3,468,557	+	*zwf1*	Glucose-6-phosphate 1-dehydrogenase	Pentose phosphate pathway, glycolysis (ED)
Chromosome	4,157,906	4,159,402	+	*zwf2*	Glucose-6-phosphate 1-dehydrogenase	Pentose phosphate pathway, glycolysis (ED)
Chromosome	5,223,741	5,222,395	-	*zwf3*	Glucose-6-phosphate 1-dehydrogenase	Pentose phosphate pathway, glycolysis (ED)

Chromosome	6,488,580	6,490,118	+	*zwf4*	Glucose-6-phosphate 1-dehydrogenase	Pentose phosphate pathway, glycolysis (ED)
Chromosome	3,469,462	3,470,205	+	*pgl*	6-phosphogluconolactonase	Pentose phosphate pathway, glycolysis (ED)
pRWAY01	276,334	275,534	-	*pgl*	6-phosphogluconolactonase	Pentose phosphate pathway, glycolysis (ED)
Chromosome	3,495	4,406	+	*gnd1*	6-Phosphogluconate dehydrogenase, decarboxylating	Pentose phosphate pathway
Chromosome	226,045	226,911	+	*gnd2*	6-Phosphogluconate dehydrogenase, decarboxylating	Pentose phosphate pathway
Chromosome	3,355,733	3,357,181	+	*gnd3*	6-Phosphogluconate dehydrogenase, decarboxylating	Pentose phosphate pathway
Chromosome	6,488,387	6,487,479	-	*gnd4*	6-Phosphogluconate dehydrogenase, decarboxylating	Pentose phosphate pathway
pRWAY01	291,430	292,347	+	*gnd*	6-Phosphogluconate dehydrogenase, decarboxylating	Pentose phosphate pathway
Chromosome	3,486,888	3,486,205	-	*rpe*	Ribulose-phosphate 3-epimerase	Pentose phosphate pathway
Chromosome	2,473,583	2,474,056	+	*rpiB*	Ribose 5-phosphate isomerase B	Pentose phosphate pathway
Chromosome	3,465,885	3,467,009	+	*tal*	Transaldolase	Pentose phosphate pathway
pRWAY01	296,705	297,859	+	*tal*	Transaldolase	Pentose phosphate pathway
Chromosome	294,587	296,695	+	*tkt1*	Transketolase	Pentose phosphate pathway
Chromosome	3,463,767	3,465,875	+	*tkt2*	Transketolase	Pentose phosphate pathway
Chromosome	1,276,787	1,278,370	+	*purF*	Amidophosphoribosyltransferase	Purine metabolism
Chromosome	1,231,769	1,233,022	+	*purD*	Phosphoribosylamine-glycine ligase	Purine metabolism
Chromosome	5,130,043	5,130,483	+	*purN*	Phosphoribosylglycinamide formyltransferase	Purine metabolism
Chromosome	1,278,489	1,279,568	+	*purM*	Phosphoribosylformylglycinamidine cyclo-ligase	Purine metabolism
Chromosome	1,271,293	1,273,578	+	*purL*	Phosphoribosylformylglycinamidine synthase, synthetase subunit	Purine metabolism
Chromosome	1,262,421	1,262,660	+	*purS*	Phosphoribosylformylglycinamidine synthase, PurS subunit	Purine metabolism
Chromosome	1,262,657	1,263,334	+	*purQ*	Phosphoribosylformylglycinamidine synthase, glutamine amidotransferase subunit	Purine metabolism
Chromosome	5,926,967	5,928,157	+	*purK*	Phosphoribosylaminoimidazole carboxylase ATPase subunit	Purine metabolism
Chromosome	5,928,150	5,928,668	+	*purE*	Phosphoribosylamidazole carboxylase catalytic subunit	Purine metabolism
Chromosome	1,246,819	1,247,703	+	*purC*	Phosphoribosylaminoimidazole-succinocarboxamide synthase	Purine metabolism
Chromosome	1,238,668	1,240,089	+	*purB*	Adenylosuccinate lyase	Purine metabolism
Chromosome	5,130,476	5,132,032	+	*purH*	Phosphoribosylaminoimidazolecarboxamide formyltransferase	Purine metabolism
Chromosome	5,097,130	5,098,419	+	*purA*	Adenylosuccinate synthetase	Purine metabolism
Chromosome	1,238,668	1,240,089	+	*purB*	Adenylosuccinate lyase	Purine metabolism
Chromosome	5,767,055	5,767,600	+	*adk1*	Adenylate kinase	Purine metabolism

Chromosome	3,300,576	3,301,157	+	*adk2*	Adenylate kinase	Purine metabolism
Chromosome	2,519,189	2,519,608	+	*ndk*	Nucleoside diphosphate kinase	Purine and pyrimidine metabolism
Chromosome	3,499,983	3,499,465	-	*gmk*	Guanylate kinase	Purine metabolism
Chromosome	5,823,892	5,825,463	+	*guaA*	GMP synthase	Purine metabolism
Chromosome	3,359,549	3,360,985	+	*guaB1*	Inosine-5’-monophosphate dehydrogenase	Purine metabolism
Chromosome	4,127,515	4,127,069	-	*guaB2*	Inosine-5’-monophosphate dehydrogenase	Purine metabolism
Chromosome	5,819,152	5,820,675	+	*guaB3*	Inosine-5’-monophosphate dehydrogenase	Purine metabolism
Chromosome	5,820,702	5,821,841	+	*guaB4*	Inosine-5’-monophosphate dehydrogenase	Purine metabolism
Chromosome	843,891	841,156	-	*xdhA1*	Xanthine dehydrogenase, molybdenum binding subunit	Purine metabolism
Chromosome	1,475,762	1,478,485	+	*xdhA2*	Xanthine dehydrogenase, molybdenum binding subunit	Purine metabolism
Chromosome	844,697	843,888	-	*xdhB1*	Xanthine dehydrogenase, FAD binding subunit	Purine metabolism
Chromosome	1,474,938	1,475,765	+	*xdhB2*	Xanthine dehydrogenase, FAD binding subunit	Purine metabolism
Chromosome	845,696	844,767	-	*pucL*	Uricase (urate oxidase)	Purine metabolism
Chromosome	866,473	865,529	-	*puuE*	Uricase (urate oxidase)	Purine metabolism
Chromosome	846,028	845,699	-	*pucM*	5-Hydroxyisourate hydrolase	Purine metabolism
Chromosome	846,540	846,028	-	*uraD*	2-oxo-4-hydroxy-4-carboxy--5-ureidoimidazoline (OHCU) decarboxylase	Purine metabolism
Chromosome	867,465	866,470	-	*alc*	Allantoicase	Purine metabolism
Chromosome	3,505,899	3,504,739	-	*carA*	Carbamoyl-phosphate synthase large chain	Pyrimidine metabolism
Chromosome	3,504,739	3,501,371	-	*carB*	Carbamoyl-phosphate synthase small chain	Pyrimidine metabolism
Chromosome	3,507,772	3,506,456	-	*pyrC*	Dihydroorotase	Pyrimidine metabolism
Chromosome	3,508,743	3,507,799	-	*pyrR*	Aspartate carbamoyltransferase	Pyrimidine metabolism
Chromosome	2,979,743	2,978,673	-	*pyrD*	Dihydroorotate dehydrogenase	Pyrimidine metabolism
Chromosome	5,063,466	5,063,999	+	*pyrE*	Orotate phosphoribosyltransferase	Pyrimidine metabolism
Chromosome	3,501,374	3,500,535	-	*pyrF*	Orotidine 5’-phosphate decarboxylase	Pyrimidine metabolism
Chromosome	6,185,523	6,186,251	+	*pyrH*	Uridine monophosphate kinase	Pyrimidine metabolism
Chromosome	2,943,519	2,945,288	+	*pyrG*	CTP synthase	Pyrimidine metabolism
Chromosome	6,061,354	6,063,537	+	*nrdE*	Ribonucleotide reductase, class Ib, alpha subunit	Pyrimidine metabolism
Chromosome	6,063,615	6,064,580	+	*nrdF*	Ribonucleotide reductase, class Ib, beta subunit	Pyrimidine metabolism
Chromosome	6,060,544	6,060,831	+	*nrdH*	Glutaredoxin-like protein NrdH, required for reduction of Ribonucleotide reductase class Ib	Pyrimidine metabolism
Chromosome	6,060,920	6,061,396	+	*nrdI*	Ribonucleotide reduction protein	Pyrimidine metabolism
Chromosome	4,849,022	4,849,591	+	*dcd*	Deoxycytidine triphosphate deaminase	Pyrimidine metabolism
Chromosome	6,347,206	6,347,652	+	*dut*	Deoxyuridine 5’-triphosphate nucleotide hydrolase	Pyrimidine metabolism
Chromosome	1,539,932	1,539,132	-	*thyA*	Thymidylate synthase	Pyrimidine metabolism
Chromosome	5,949,443	5,950,141	+	*tmk1*	Thymidylate kinase	Pyrimidine metabolism
Chromosome	5,373,059	5,375,026	+	*tmk2*	Thymidylate kinase	Pyrimidine metabolism

Chromosome	152,600	154,102	+	*fadD1*	Long-chain-fatty-acid--CoA ligase	Beta-oxidation
Chromosome	502,624	500,777	-	*fadD2*	Long-chain-fatty-acid--CoA ligase	Beta-oxidation
Chromosome	917,717	916,167	-	*fadD3*	Long-chain-fatty-acid--CoA ligase	Beta-oxidation
Chromosome	1,040,510	1,042,186	+	*fadD4*	Long-chain-fatty-acid--CoA ligase	Beta-oxidation
Chromosome	1,044,931	1,043,360	-	*fadD5*	Long-chain-fatty-acid--CoA ligase	Beta-oxidation
Chromosome	1,171,757	1,170,342	-	*fadD6*	Long-chain-fatty-acid--CoA ligase	Beta-oxidation
Chromosome	1,878,765	1,880,564	+	*fadD7*	Long-chain-fatty-acid--CoA ligase	Beta-oxidation
Chromosome	2,744,757	2,742,946	-	*fadD8*	Long-chain-fatty-acid--CoA ligase	Beta-oxidation
Chromosome	3,907,778	3,906,096	-	*fadD9*	Long-chain-fatty-acid--CoA ligase	Beta-oxidation
Chromosome	4,051,539	4,050,073	-	*fadD10*	Long-chain-fatty-acid--CoA ligase	Beta-oxidation
Chromosome	4,224,541	4,226,064	+	*fadD11*	Long-chain-fatty-acid--CoA ligase	Beta-oxidation
Chromosome	4,288,207	4,289,730	+	*fadD12*	Long-chain-fatty-acid--CoA ligase	Beta-oxidation
Chromosome	5,543,473	5,545,248	+	*fadD13*	Long-chain-fatty-acid--CoA ligase	Beta-oxidation
Chromosome	5,605,565	5,606,755	+	*fadD14*	Long-chain-fatty-acid--CoA ligase	Beta-oxidation
Chromosome	5,618,475	5,616,709	-	*fadD15*	Long-chain-fatty-acid--CoA ligase	Beta-oxidation
Chromosome	5,681,092	5,682,729	+	*fadD16*	Long-chain-fatty-acid--CoA ligase	Beta-oxidation
Chromosome	5,742,135	5,743,724	+	*fadD17*	Long-chain-fatty-acid--CoA ligase	Beta-oxidation
pRWAY01	606,992	605,433	-	*fadD*	Long-chain-fatty-acid--CoA ligase	Beta-oxidation
Chromosome	1,751,879	1,749,999	-	*acx*	Acyl-CoA oxidase	Beta-oxidation
Chromosome	202,429	203,574	+	*fadE1*	Butyryl-CoA dehydrogenase	Beta-oxidation
Chromosome	217,813	216,650	-	*fadE2*	Branched-chain acyl-CoA dehydrogenase	Beta-oxidation
Chromosome	500,744	499,575	-	*fadE3*	Butyryl-CoA dehydrogenase	Beta-oxidation
Chromosome	821,632	823,794	+	*fadE4*	Butyryl-CoA dehydrogenase	Beta-oxidation
Chromosome	823,877	824,917	+	*fadE5*	Acyl-CoA dehydrogenase	Beta-oxidation
Chromosome	824,902	826,053	+	*fadE6*	Acyl-CoA dehydrogenase	Beta-oxidation
Chromosome	913,176	912,118	-	*fadE7*	Butyryl-CoA dehydrogenase	Beta-oxidation
Chromosome	914,141	913,173	-	*fadE8*	Butyryl-CoA dehydrogenase	Beta-oxidation
Chromosome	915,298	914,138	-	*fadE9*	Butyryl-CoA dehydrogenase	Beta-oxidation
Chromosome	1,046,049	1,044,928	-	*fadE10*	Butyryl-CoA dehydrogenase	Beta-oxidation
Chromosome	1,135,137	1,133,836	-	*fadE11*	Butyryl-CoA dehydrogenase	Beta-oxidation
Chromosome	1,331,093	1,332,352	+	*fadE12*	Acyl-CoA dehydrogenase, short-chain specific	Beta-oxidation
Chromosome	1,852,221	1,853,411	+	*fadE13*	Butyryl-CoA dehydrogenase	Beta-oxidation
Chromosome	2,034,972	2,036,204	+	*fadE14*	Butyryl-CoA dehydrogenase	Beta-oxidation
Chromosome	2,054,090	2,055,238	+	*fadE15*	Butyryl-CoA dehydrogenase	Beta-oxidation
Chromosome	2,342,932	2,344,032	+	*fadE16*	Butyryl-CoA dehydrogenase	Beta-oxidation
Chromosome	2,839,029	2,840,972	+	*fadE17*	Butyryl-CoA dehydrogenase	Beta-oxidation
Chromosome	3,867,347	3,868,498	+	*fadE18*	Butyryl-CoA dehydrogenase	Beta-oxidation
Chromosome	4,055,055	4,053,814	-	*fadE19*	Butyryl-CoA dehydrogenase	Beta-oxidation

Chromosome	4,087,431	4,088,747	+	*fadE20*	Butyryl-CoA dehydrogenase	Beta-oxidation
Chromosome	4,184,512	4,185,747	+	*fadE21*	Butyryl-CoA dehydrogenase	Beta-oxidation
Chromosome	4,235,387	4,234,230	-	*fadE22*	Butyryl-CoA dehydrogenase	Beta-oxidation
Chromosome	4,354,022	4,351,809	-	*fadE23*	Acyl-CoA dehydrogenase	Beta-oxidation
Chromosome	4,390,385	4,389,225	-	*fadE24*	Butyryl-CoA dehydrogenase	Beta-oxidation
Chromosome	4,525,431	4,526,666	+	*fadE25*	Butyryl-CoA dehydrogenase	Beta-oxidation
Chromosome	6,031,073	6,029,661	-	*fadE26*	Butyryl-CoA dehydrogenase	Beta-oxidation
pRWAY02	154,775	155,296	+	*fadE*	Acyl-CoA dehydrogenase	Beta-oxidation
Chromosome	5,322,024	5,323,745	+	*acd*	Acyl-CoA dehydrogenase	Beta-oxidation
Chromosome	214,606	213,830	-	*paaF1*	Enoyl-CoA hydratase	Beta-oxidation
Chromosome	2,316,794	2,317,570	+	*paaF2*	Enoyl-CoA hydratase	Beta-oxidation
Chromosome	4,223,702	4,224,484	+	*paaF3*	Enoyl-CoA hydratase	Beta-oxidation
pRWAY01	684,077	683,265	-	*paaF*	Enoyl-CoA hydratase	Beta-oxidation
Chromosome	329,086	329,886	+	*echA1*	Enoyl-CoA hydratase	Beta-oxidation
Chromosome	643,577	644,368	+	*echA2*	Enoyl-CoA hydratase	Beta-oxidation
Chromosome	757,482	758,312	+	*echA3*	Enoyl-CoA hydratase	Beta-oxidation
Chromosome	1,007,248	1,006,472	-	*echA4*	Enoyl-CoA hydratase	Beta-oxidation
Chromosome	1,040,430	1,039,588	-	*echA5*	Enoyl-CoA hydratase	Beta-oxidation
Chromosome	1,210,711	1,211,499	+	*echA6*	Enoyl-CoA hydratase	Beta-oxidation
Chromosome	1,499,315	1,500,052	+	*echA7*	Enoyl-CoA hydratase	Beta-oxidation
Chromosome	3,559,761	3,560,525	+	*echA8*	Enoyl-CoA hydratase	Beta-oxidation
Chromosome	3,707,957	3,707,211	-	*echA9*	Enoyl-CoA hydratase	Beta-oxidation
Chromosome	4,052,432	4,051,536	-	*echA10*	Enoyl-CoA hydratase	Beta-oxidation
Chromosome	4,058,281	4,059,081	+	*echA11*	Enoyl-CoA hydratase	Beta-oxidation
Chromosome	4,606,554	4,607,624	+	*echA12*	Enoyl-CoA hydratase	Beta-oxidation
Chromosome	5,321,009	5,320,134	-	*echA13*	Enoyl-CoA hydratase	Beta-oxidation
Chromosome	5,604,662	5,605,486	+	*echA14*	Enoyl-CoA hydratase	Beta-oxidation
Chromosome	6,076,178	6,076,957	+	*echA15*	Enoyl-CoA hydratase	Beta-oxidation
Chromosome	332,634	333,395	+	*hadH1*	3-hydroxyacyl-CoA dehydrogenase	Beta-oxidation
Chromosome	399,565	398,741	-	*hadH2*	3-hydroxyacyl-CoA dehydrogenase	Beta-oxidation
Chromosome	1,106,236	1,105,472	-	*hadH3*	3-hydroxyacyl-CoA dehydrogenase	Beta-oxidation
Chromosome	2,037,476	2,038,243	+	*hadH4*	3-hydroxyacyl-CoA dehydrogenase	Beta-oxidation
Chromosome	4,072,974	4,072,195	-	*hadH5*	3-hydroxyacyl-CoA dehydrogenase	Beta-oxidation
Chromosome	4,239,395	4,240,159	+	*hadH6*	3-hydroxyacyl-CoA dehydrogenase	Beta-oxidation
Chromosome	4,689,368	4,690,132	+	*hadH7*	3-hydroxyacyl-CoA dehydrogenase	Beta-oxidation
pRWAY01	549,550	550,314	+	*hadH*	3-hydroxyacyl-CoA dehydrogenase	Beta-oxidation
Chromosome	603,355	604,569	+	*fadA1*	Acetyl-CoA acetyltransferase	Beta-oxidation
Chromosome	920,511	921,659	+	*fadA2*	Acetyl-CoA acetyltransferase	Beta-oxidation

Chromosome	1,127,499	1,128,644	+	*fadA3*	Acetyl-CoA acetyltransferase	Beta-oxidation
Chromosome	1,340,403	1,341,620	+	*fadA4*	Acetyl-CoA acetyltransferase	Beta-oxidation
Chromosome	1,370,895	1,372,112	+	*fadA5*	Acetyl-CoA acetyltransferase	Beta-oxidation
Chromosome	2,047,844	2,049,061	+	*fadA6*	Acetyl-CoA acetyltransferase	Beta-oxidation
Chromosome	3,621,192	3,622,406	+	*fadA7*	Acetyl-CoA acetyltransferase	Beta-oxidation
Chromosome	4,036,231	4,037,307	+	*fadA8*	Acetyl-CoA acetyltransferase	Beta-oxidation
Chromosome	4,061,756	4,063,015	+	*fadA9*	Acetyl-CoA acetyltransferase	Beta-oxidation
Chromosome	4,238,230	4,239,363	+	*fadA10*	Acetyl-CoA acetyltransferase	Beta-oxidation
Chromosome	4,580,184	4,581,530	+	*fadA11*	Acetyl-CoA acetyltransferase	Beta-oxidation
Chromosome	4,688,118	4,689,248	+	*fadA12*	Acetyl-CoA acetyltransferase	Beta-oxidation
Chromosome	5,092,662	5,091,493	-	*fadA13*	Acetyl-CoA acetyltransferase	Beta-oxidation
Chromosome	5,362,086	5,360,869	-	*fadA14*	Acetyl-CoA acetyltransferase	Beta-oxidation
Chromosome	6,374,989	6,373,781	-	*fadA15*	Acetyl-CoA acetyltransferase	Beta-oxidation
pRWAY01	610,582	609,365	-	*fadA1*	Acetyl-CoA acetyltransferase	Beta-oxidation
pRWAY01	926,547	927,758	+	*fadA2*	Acetyl-CoA acetyltransferase	Beta-oxidation
pRWAY02	393,598	392,452	-	*fadA*	Acetyl-CoA acetyltransferase	Beta-oxidation
Chromosome	2,036,222	2,037,421	+	*atoB1*	Acetyl-CoA acetyltransferase	Beta-oxidation
Chromosome	2,394,262	2,395,527	+	*atoB2*	Acetyl-CoA acetyltransferase	Beta-oxidation
Chromosome	4,452,922	4,451,729	-	*atoB3*	Acetyl-CoA acetyltransferase	Beta-oxidation
Chromosome	5,690,109	5,691,311	+	*atoB4*	Acetyl-CoA acetyltransferase	Beta-oxidation
pRWAY02	182,627	181,434	-	*atoB1*	Acetyl-CoA acetyltransferase	Beta-oxidation
pRWAY02	395,944	397,281	+	*atoB2*	Acetyl-CoA acetyltransferase	Beta-oxidation
Chromosome	6,115,406	6,117,001	+	*serA*	D-3-phosphoglycerate dehydrogenase	Serine metabolism
Chromosome	1,741,809	1,740,832	-	*serB1*	Phosphoserine phosphatase	Serine metabolism
Chromosome	6,069,566	6,070,786	+	*serB2*	Phosphoserine phosphatase	Serine metabolism
Chromosome	1,456,832	1,455,711	-	*serC*	Phosphoserine aminotransferase	Serine metabolism
Chromosome	665,396	666,661	+	*lysC*	Aspartokinase	Threonine, methionine and lysine metabolism
Chromosome	666,665	667,696	+	*asd*	Aspartate-semialdehyde dehydrogenase	Threonine, methionine and lysine metabolism
Chromosome	2,356,594	2,357,967	+	*hom*	Homoserine dehydrogenase	Threonine and methionine metabolism
Chromosome	2,359,046	2,360,035	+	*thrB*	Homoserine kinase	Threonine metabolism
Chromosome	2,357,964	2,359,046	+	*thrC*	Threonine synthase	Threonine metabolism
Chromosome	1,765,222	1,766,331	+	*cysK1*	Cysteine synthase B	Cysteine metabolism
Chromosome	2,414,983	2,415,945	+	*cysK2*	Cysteine synthase B	Cysteine metabolism
Chromosome	5,363,353	5,364,738	+	*cbs*	Cystathionine beta-synthase	Cysteine and methionine metabolism

Chromosome	5,365,344	5,366,516	+	*cth*	Cystathionine gamma-lyase	Cysteine metabolism
Chromosome	3,497,662	3,496,448	-	*metK*	S-adenosylmethionine synthetase	Cysteine and methionine metabolism
Chromosome	6,057,375	6,057,926	+	*pfs*	S-adenosylhomocysteine nucleosidase	Cysteine metabolism
Chromosome	1,681,725	1,684,019	+	*metE*	5-methyltetrahydropteroyltriglutamate--homocysteine methyltransferase	Methionine metabolism
Chromosome	3,006,977	3,010,546	+	*metH*	5-methyltetrahydrofolate--homocysteine methyltransferase	Methionine metabolism
Chromosome	5,947,902	5,949,386	+	*achy*	Adenosylhomocysteinase	Methionine metabolism
Chromosome	552,431	551,289	-	*cdm*	DNA (cytosine-5-)-methyltransferase	Methionine metabolism
Chromosome	6,111,821	6,113,629	+	*ilvB*	Acetolactate synthase large subunit	Valine/Isoleucine metabolism
Chromosome	6,114,168	6,115,181	+	*ilvC*	Ketol-acid reductoisomerase	Valine/Isoleucine metabolism
Chromosome	6,110,875	6,109,031	-	*ilvD*	Dihydroxy-acid dehydratase	Valine/Isoleucine metabolism
Chromosome	2,152,655	2,153,575	+	*ilvE1*	Branched-chain amino acid aminotransferase	Valine/Isoleucine metabolism
Chromosome	2,236,093	2,236,944	+	*ilvE2*	Branched-chain amino acid aminotransferase	Valine/Isoleucine metabolism
Chromosome	2,714,338	2,715,441	+	*ivlE3*	Branched-chain amino acid aminotransferase	Valine/Isoleucine metabolism
Chromosome	6,117,075	6,118,085	+	*leuB*	3-isopropylmalate dehydrogenase	Valine/Leucine/Isoleucine metabolism
Chromosome	196,085	197,446	+	*leuC1*	3-isopropylmalate dehydratase large subunit	Valine/Leucine/Isoleucine metabolism
Chromosome	4,332,542	4,331,142	-	*leuC2*	3-isopropylmalate dehydratase large subunit	Valine/Leucine/Isoleucine metabolism
Chromosome	6,123,892	6,125,313	+	*leuC3*	3-isopropylmalate dehydratase large subunit	Valine/Leucine/Isoleucine metabolism
Chromosome	197,453	198,070	+	*leuD1*	3-isopropylmalate dehydratase small subunit	Valine/Leucine/Isoleucine metabolism
Chromosome	4,331,105	4,330,509	-	*leuD2*	3-isopropylmalate dehydratase small subunit	Valine/Leucine/Isoleucine metabolism
Chromosome	6,125,341	6,125,949	+	*leuD3*	3-isopropylmalate dehydratase small subunit	Valine/Leucine/Isoleucine metabolism
Chromosome	629,417	627,609	-	*leuA*	2-isopropylmalate synthase	Leucine metabolism
Chromosome	1,660,395	1,661,798	+	*lpd1*	Dihydrolipoamide dehydrogenase	Valine/Leucine/Isoleucine metabolism
Chromosome	2,026,644	2,025,241	-	*lpd2*	Dihydrolipoamide dehydrogenase	Valine/Leucine/Isoleucine metabolism
Chromosome	3,670,350	3,668,971	-	*lpd3*	Dihydrolipoamide dehydrogenase	Valine/Leucine/Isoleucine metabolism

Chromosome	3,743,185	3,741,776	-	*lpd4*	Dihydrolipoamide dehydrogenase	Valine/Leucine/Isoleucine metabolism
Chromosome	5,885,786	5,887,189	+	*lpdA*	Dihydrolipoamide dehydrogenase	Valine/Leucine/Isoleucine metabolism
Chromosome	3,709,536	3,710,474	+	*dapA1*	4-hydroxy-tetrahydrodipicolinate synthase	Lysine metabolism
Chromosome	4,631,178	4,632,137	+	*dapA2*	4-hydroxy-tetrahydrodipicolinate synthase	Lysine metabolism
Chromosome	4,806,191	4,805,298	-	*dapA3*	4-hydroxy-tetrahydrodipicolinate synthase	Lysine metabolism
Chromosome	6,259,951	6,260,859	+	*dapA4*	4-hydroxy-tetrahydrodipicolinate synthase	Lysine metabolism
Chromosome	6,254,943	6,255,701	+	*dapB*	4-hydroxy-tetrahydrodipicolinate reductase	Lysine metabolism
Chromosome	5,540,747	5,539,794	-	*dapD*	2,3,4,5-tetrahydropyridine-2,6-dicarboxylate N-succinyltransferase	Lysine metabolism
Chromosome	2,916,585	2,917,784	+	*argD*	Acetylornithine aminotransferase	Lysine and arginine metabolism
Chromosome	5,526,715	5,527,815	+	*dapC*	N-succinyl-L,L-diaminopimelate aminotransferase alternative	Lysine and arginine metabolism
Chromosome	5,540,915	5,541,988	+	*dapE*	N-succinyl-L,L-diaminopimelate desuccinylase	Lysine metabolism
Chromosome	6,288,450	6,289,319	+	*dapF*	Diaminopimelate epimerase	Lysine metabolism
Chromosome	2,355,176	2,356,597	+	*lysA*	Diaminopimelate decarboxylase	Lysine metabolism
Chromosome	2,914,424	2,915,665	+	*argJ*	N-acetylglutamate synthase	Arginine metabolism
Chromosome	2,915,662	2,916,588	+	*argB*	Acetylglutamate kinase	Arginine metabolism
Chromosome	2,913,372	2,914,427	+	*argC*	N-acetyl-gamma-glutamyl-phosphate reductase	Arginine metabolism
Chromosome	2,917,781	2,918,728	+	*argF*	Ornithine carbamoyltransferase	Arginine metabolism
Chromosome	2,919,291	2,920,490	+	*argG*	Argininosuccinate synthase	Arginine metabolism
Chromosome	2,920,493	2,921,911	+	*argH*	Argininosuccinate lyase	Arginine metabolism
Chromosome	2,526,075	2,527,175	+	*proB*	Glutamate 5-kinase	Proline metabolism
Chromosome	2,537,372	2,538,649	+	*proA*	Gamma-glutamyl phosphate reductase	Proline metabolism
Chromosome	1,737,053	1,737,868	+	*proC*	Pyrroline-5-carboxylate reductase	Proline metabolism
Chromosome	3,013,375	3,014,226	+	*hisG*	ATP phosphoribosyltransferase	Histidine metabolism
Chromosome	3,013,040	3,013,321	+	*hisE*	Phosphoribosyl-ATP pyrophosphatase	Histidine metabolism
Chromosome	2,849,242	2,849,586	+	*hisI*	Phosphoribosyl-AMP cyclohydrolase	Histidine metabolism
Chromosome	2,847,041	2,847,634	+	*hisA*	Phosphoribosylformimino-5-aminoimidazole carboxamide ribotide isomerase	Histidine metabolism
Chromosome	2,848,472	2,849,245	+	*hisF*	Imidazole glycerol phosphate synthase cyclase subunit	Histidine metabolism
Chromosome	2,843,989	2,844,603	+	*hisB*	Imidazoleglycerol-phosphate dehydratase	Histidine metabolism
Chromosome	2,842,850	2,843,992	+	*hisC*	Histidinol-phosphate aminotransferase	Histidine metabolism
Chromosome	2,841,459	2,842,853	+	*hisD*	Histidinol dehydrogenase	Histidine metabolism
Chromosome	1,643,543	1,645,090	+	*hutH*	Histidine ammonia-lyase	Histidine metabolism
Chromosome	1,001,825	1,000,152	-	*hutU*	Urocanate hydratase	Histidine metabolism
Chromosome	1,000,142	998,937	-	*hutI*	Imidazolonepropionase	Histidine metabolism

Chromosome	998,940	997,987	-	*hutG*	Formiminoglutamase	Histidine metabolism
Chromosome	2,854,221	2,855,030	+	*trpC*	Indole-3-glycerol phosphate synthase	Tryptophan metabolism
Chromosome	2,734,500	2,735,603	+	*trpD*	Anthranilate phosphoribosyltransferase	Tryptophan metabolism
Chromosome	2,851,829	2,853,427	+	*trpE*	Anthranilate synthase, aminase component	Tryptophan metabolism
Chromosome	4,523,978	4,523,388	-	*trpF*	Phosphoribosylanthranilate isomerase	Tryptophan metabolism
Chromosome	32,654	33,307	+	*trpG*	Anthranilate synthase, amidotransferase component	Tryptophan metabolism
Chromosome	2,855,093	2,856,415	+	*trpB*	Tryptophan synthase beta chain	Tryptophan metabolism
Chromosome	2,856,412	2,857,215	+	*trpA*	Tryptophan synthase alpha chain	Tryptophan metabolism
Chromosome	5,117,800	5,117,504	-	*csm*	Chorismate mutase I	Phenylalanine, tyrosine and tryptophan metabolism
Chromosome	430,018	430,935	+	*pheA*	Prephenate dehydratase	Phenylalanine metabolism
Chromosome	5,006,667	5,005,414	-	*aspC*	Aspartate aminotransferase	Phenylalanine, tyrosine and tryptophan metabolism
Chromosome	555,293	554,343	-	*tyrA*	Prephenate dehydrogenase	Phenylalanine, tyrosine and tryptophan metabolism
Chromosome	541,975	540,899	-	*tat1*	Tyrosine aminotransferase	Phenylalanine, tyrosine and tryptophan metabolism
Chromosome	4,430,232	4,429,150	-	*tat2*	Tyrosine aminotransferase	Phenylalanine, tyrosine and tryptophan metabolism
Chromosome	1,753,343	1,754,551	+	*hppD1*	4-hydroxyphenylpyruvate dioxygenase	Tyrosine and phenylalanine metabolism
Chromosome	3,791,827	3,793,032	+	*hppD2*	4-hydroxyphenylpyruvate dioxygenase	Tyrosine and phenylalanine metabolism
Chromosome	2,083,970	2,084,818	+	*kynA*	Tryptophan 2,3-dioxygenase	Tryptophan metabolism
Chromosome	2,084,815	2,086,050	+	*kynU*	Kynureninase	Tryptophan metabolism
**Central aromatic metabolism**						
Chromosome	2,500,526	2,499,885	-	*pcaG*	Protocatechuate 3,4-dioxygenase alpha chain	Beta-ketoadipate
Chromosome	2,501,245	2,500,526	-	*pcaH*	Protocatechuate 3,4-dioxygenase beta chain	Beta-ketoadipate
Chromosome	2,499,862	2,498,516	-	*pcaB*	3-carboxy-cis,cis-muconate cycloisomerase	Beta-ketoadipate
Chromosome	2,498,519	2,497,326	-	*pcaC*	4-carboxymuconolactone decarboxylase	Beta-ketoadipate
Chromosome	345,521	346,303	+	*pcaD1*	Beta-ketoadipate enol-lactone hydrolase	Beta-ketoadipate
Chromosome	1,067,030	1,067,833	+	*pcaD2*	Beta-ketoadipate enol-lactone hydrolase	Beta-ketoadipate
Chromosome	3,589,424	3,590,290	+	*pcaD3*	Beta-ketoadipate enol-lactone hydrolase	Beta-ketoadipate
Chromosome	3,693,989	3,694,861	+	*pcaD4*	Beta-ketoadipate enol-lactone hydrolase	Beta-ketoadipate
Chromosome	6,450,972	6,450,100	-	*pcaD5*	Beta-ketoadipate enol-lactone hydrolase	Beta-ketoadipate
Chromosome	2,214,986	2,215,750	+	*pcaI*	Succinyl-CoA:3-ketoacid-coenzyme A transferase subunit A	Beta-ketoadipate

Chromosome	2,215,747	2,216,415	+	*pcaJ*	Succinyl-CoA:3-ketoacid-coenzyme A transferase subunit B	Beta-ketoadipate
Chromosome	4,154,203	4,155,045	+	*catA*	Catechol 1,2-dioxygenase	Beta-ketoadipate, catechol ortho-cleavage
Chromosome	4,155,075	4,156,196	+	*catB*	Muconate cycloisomerase	Beta-ketoadipate, catechol ortho-cleavage
Chromosome	4,156,212	4,156,493	+	*catC*	Muconolactone isomerase	Beta-ketoadipate, catechol ortho-cleavage
pRWAY01	74,049	74,930	+	*catE*	Catechol 2,3-dioxygenase	Catechol meta-cleavage
pRWAY02	198,132	197,251	-	*catE*	Catechol 2,3-dioxygenase	Catechol meta-cleavage
Chromosome	121,449	122,234	+	*bphE1*	2-keto-4-pentenoate hydratase	2-hydroxypentadienoate metabolism
Chromosome	881,759	882,544	+	*bphE2*	2-keto-4-pentenoate hydratase	2-hydroxypentadienoate metabolism
Chromosome	3,892,276	3,891,557	-	*bphE3*	2-keto-4-pentenoate hydratase	2-hydroxypentadienoate metabolism
pRWAY01	42,998	43,816	+	*bphE*	2-keto-4-pentenoate hydratase	2-hydroxypentadienoate metabolism
pRWAY02	191,841	192,785	+	*bphE*	2-keto-4-pentenoate hydratase	2-hydroxypentadienoate metabolism
Chromosome	123,186	124,244	+	*bphF1*	4-hydroxy-2-oxovalerate aldolase	2-hydroxypentadienoate metabolism
Chromosome	883,464	884,492	+	*BphF2*	4-hydroxy-2-oxovalerate aldolase	2-hydroxypentadienoate metabolism
Chromosome	3,890,592	3,889,582	-	*bphF3*	4-hydroxy-2-oxovalerate aldolase	2-hydroxypentadienoate metabolism
pRWAY01	44,802	45,821	+	*bphF1*	4-hydroxy-2-oxovalerate aldolase	2-hydroxypentadienoate metabolism
pRWAY01	66,000	65,809	-	*BphF2*	4-hydroxy-2-oxovalerate aldolase	2-hydroxypentadienoate metabolism
pRWAY01	66,562	66,059	-	*bphF3*	4-hydroxy-2-oxovalerate aldolase	2-hydroxypentadienoate metabolism
pRWAY02	193,768	194,787	+	*bphF*	4-hydroxy-2-oxovalerate aldolase	2-hydroxypentadienoate metabolism
Chromosome	122,246	123,148	+	*bphG1*	Acetaldehyde dehydrogenase	2-hydroxypentadienoate metabolism

Chromosome	882,556	883,458	+	*bphG2*	Acetaldehyde dehydrogenase	2-hydroxypentadienoate metabolism
Chromosome	3,891,560	3,890,589	-	*bphG3*	Acetaldehyde dehydrogenase	2-hydroxypentadienoate metabolism
pRWAY01	43,822	44,805	+	*bphG1*	Acetaldehyde dehydrogenase	2-hydroxypentadienoate metabolism
pRWAY01	66,900	66,571	-	*bphG2*	Acetaldehyde dehydrogenase	2-hydroxypentadienoate metabolism
pRWAY02	192,791	193,771	+	*bphG1*	Acetaldehyde dehydrogenase	2-hydroxypentadienoate metabolism
pRWAY02	205,797	206,407	+	*bphG2*	Acetaldehyde dehydrogenase	2-hydroxypentadienoate metabolism
Chromosome	4,144,636	4,143,254	-	*benA*	Benzoate 1,2-dioxygenase alpha subunit	Benzoate metabolism
Chromosome	4,143,239	4,142,730	-	*benB*	Benzoate 1,2-dioxygenase beta subunit	Benzoate metabolism
Chromosome	4,142,692	4,141,532	-	*benC*	benzoate dioxygenase, ferredoxin reductase component	Benzoate metabolism
Chromosome	4,141,535	4,140,741	-	*benD*	1,2-dihydroxycyclohexa-3,5-diene-1-carboxylate dehydrogenase	Benzoate metabolism
Chromosome	4,623,722	4,622,622	-	*gdoA1*	Gentisate 1,2-dioxygenase	Gentisate metabolism
Chromosome	4,661,780	4,660,668	-	*gdoA2*	Gentisate 1,2-dioxygenase	Gentisate metabolism
pRWAY01	73,562	74,041	+	*gdoA*	Gentisate 1,2-dioxygenase	Gentisate metabolism
pRWAY02	198,619	198,140	-	*gdoA*	Gentisate 1,2-dioxygenase	Gentisate metabolism
Chromosome	4,219,043	4,220,242	+	*hmgA*	Homogentisate 1,2-dioxygenase	Homogentisate metabolism

Table S6. Genes involved in the peripheral metabolism of *Rhodococcus* sp. WAY2

Replicon	Start	End	Strand	Gene name	Predicted function	Cellular function
pRWAY01	509,217	507,868	-	*bphA1a*	Biphenyl dioxygenase, alpha subunit	Biphenyl/PCBs metabolism
pRWAY01	507,833	507,297	-	*bphA2a*	Biphenyl dioxygenase, alpha subunit	Biphenyl/PCBs metabolism
pRWAY01	506,547	506,209	-	*bphA3*	Biphenyl dioxygenase ferredoxin subunit	Biphenyl/PCBs metabolism
pRWAY01	506,124	504,889	-	*bphA4*	Ferredoxin reductase component of biphenyl dioxygenase	Biphenyl/PCBs metabolism
pRWAY01	511,414	510,599	-	*bphB*	2,3-dihydroxy-2,3-dihydro-biphenyl dehydrogenase	Biphenyl/PCBs metabolism
pRWAY01	510,569	509,682	-	*bphC*	2,3-dihydroxybiphenyl 1,2-dioxygenase	Biphenyl/PCBs metabolism
pRWAY01	504,851	503,994	-	*bphD*	2-hydroxy-6-oxo-6-phenylhexa-2,4-dienoate hydrolase	Biphenyl/PCBs metabolism
pRWAY01	503,862	503,449	-	*bphE*	2-keto-4-pentenoate hydratase	Biphenyl/PCBs metabolism
pRWAY01	60,245	58,839	-	*bphA1b*	Biphenyl dioxygenase, alpha subunit	Biphenyl/PCBs metabolism
pRWAY01	58,835	58,317	-	*bphA2b*	Biphenyl dioxygenase, alpha subunit	Biphenyl/PCBs metabolism
pRWAY01	58,025	57,210	-	*bphB*	2,3-dihydroxy-2,3-dihydro-biphenyl dehydrogenase	Biphenyl/PCBs metabolism
pRWAY01	70,437	71,297	+	*bphD*	2-hydroxy-6-oxo-6-phenylhexa-2,4-dienoate hydrolase	Biphenyl/PCBs metabolism
pRWAY02	156,099	157,481	+	*etbA1a*	Ethylbenzene dioxygenase, large subunit	Ethylbenzene/biphenyl/PCBs metabolism
pRWAY02	157,504	158,052	+	*etbA2a*	Ethylbenzene dioxygenase, small subunit	Ethylbenzene/biphenyl/PCBs metabolism
pRWAY02	158,072	158,989	+	*etbC*	1,2-dihydroxyethylbenzene dioxygenase	Ethylbenzene/biphenyl/PCBs metabolism
pRWAY02	150,062	150,874	+	*bphB*	2,3-dihydroxy-2,3-dihydro-biphenyl dehydrogenase	Ethylbenzene/biphenyl/PCBs metabolism
pRWAY02	159,112	159,969	+	*bphD*	2-hydroxy-6-oxo-6-phenylhexa-2,4-dienoate hydrolase	Ethylbenzene/biphenyl/PCBs metabolism
pRWAY02	160,020	160,823	+	*bphE*	2-oxo-hepta-3-ene-1,7-dioic acid hydratase	Ethylbenzene/biphenyl/PCBs metabolism
pRWAY02	160,835	161,605	+	*bphF*	2,4-dihydroxyhept-2-ene-1,7-dioic acid aldolase	Ethylbenzene/biphenyl/PCBs metabolism
pRWAY02	167,930	169,312	+	*etbA1b*	Ethylbenzene dioxygenase, large subunit	Ethylbenzene/biphenyl/PCBs metabolism
pRWAY02	169,335	169,883	+	*etbA2b*	Ethylbenzene dioxygenase, small subunit	Ethylbenzene/biphenyl/PCBs metabolism

pRWAY02	169,913	170,278	+	*etbA3*	ferredoxin	Ethylbenzene/biphenyl/PCBs metabolism
pRWAY02	201,744	200,884	-	*bphD*	2-hydroxy-6-oxo-6-phenylhexa-2,4-dienoate hydrolase	Ethylbenzene/biphenyl/PCBs metabolism
pRWAY02	216,173	217,579	+	*nahA1*	Naphthalene dioxygenase alpha subunit	Naphthalene/biphenyl/PCBs metabolism
pRWAY02	217,583	218,101	+	*nahA2*	Naphthalene dioxygenase beta subunit	Naphthalene/biphenyl/PCBs metabolism
pRWAY02	218,115	218,387	+	*nahC*	dihydrodiol dehydrogenase	Naphthalene/biphenyl/PCBs metabolism
pRWAY02	218,393	219,208	+	*nahB*	cis-naphthalene dihydrodiol dehydrogenase	Naphthalene/biphenyl/PCBs metabolism
pRWAY01	15,833	15,171	-	*tmoF*	Oxidoreductase FAD-binding domain-containing protein	Toluene metabolism
pRWAY01	17,197	16,160	-	*tmoE*	Toluene-4-monooxygenase, subunit	Toluene metabolism
pRWAY01	17,495	17,199	-	*tmoD*	monooxygenase component	Toluene metabolism
pRWAY01	17,882	17,514	-	*tmoC*	Toluene-4-monooxygenase, subunit	Toluene metabolism
pRWAY01	18,124	17,879	-	*tmoB*	Toluene monooxygenase	Toluene metabolism
pRWAY01	19,655	18,141	-	*tmoA*	Toluene-4-monooxygenase, subunit	Toluene metabolism
Chromosome	942,988	944,622	+	*mmoX*	Methane monooxygenase component A alpha chain	Methane metabolism
Chromosome	944,707	945,750	+	*mmoC*	Methane monooxygenase component C	Methane metabolism
Chromosome	945,801	946,907	+	*mmoY*	Methane monooxygenase component A beta chain	Methane metabolism
Chromosome	946,904	947,245	+	*mmoB*	Methane monooxygenase regulatory protein B	Methane metabolism
pRWAY02	238,905	240,443	+	*mmoX1*	Methane monooxygenase component A alpha chain	Methane metabolism
pRWAY02	240,443	241,534	+	*mmoY1*	Methane monooxygenase component A beta chain	Methane metabolism
pRWAY02	241,531	241,848	+	*mmoB1*	Methane monooxygenase regulatory protein B	Methane metabolism
pRWAY02	241,860	242,891	+	*mmoC1*	Methane monooxygenase component C	Methane metabolism
pRWAY02	274,460	276,040	+	*mmoX2*	Methane monooxygenase component A alpha chain	Methane metabolism
pRWAY02	276,239	277,456	+	*mmoY2*	Methane monooxygenase component A beta chain	Methane metabolism
pRWAY02	277,859	277,996	+	*mmoB2*	Methane monooxygenase regulatory protein B	Methane metabolism
pRWAY02	278,372	279,394	+	*mmoC2*	Methane monooxygenase component C	Methane metabolism
pRWAY02	279,463	279,981	+	*mmoZ*	Methane monooxygenase component A gamma chain	Methane metabolism
pRWAY02	318,630	317,356	-	*pmoB*	Particulate methane monooxygenase B-subunit	Methane metabolism
pRWAY02	319,492	318,641	-	*pmoA*	Particulate methane monooxygenase A-subunit	Methane metabolism
pRWAY02	320,498	319,620	-	*pmoC*	Particulate methane monooxygenase C-subunit	Methane metabolism
Chromosome	5,945,530	5,946,753	+	*alkB*	Alkane 1-monooxygenase	Alkane degradation
Chromosome	357,092	358,192	+	*ladA1*	Long-chain alkane monooxygenase	Alkane degradation
Chromosome	358,189	359,343	+	*ladA2*	Long-chain alkane monooxygenase	Alkane degradation
Chromosome	4,017,878	4,019,047	+	*ladA3*	Long-chain alkane monooxygenase	Alkane degradation

Table S7. Genes involved in stress responses found in *Rhodococcus* sp. WAY2

Replicon	Start	End	Strand	Gene name	Predicted function	Function, pathway
**Storage compounds metabolism**						
Chromosome	5,214,900	5,215,832	+	*galU*	UTP--glucose-1-phosphate uridylyltransferase	Glycogen biosynthesis
Chromosome	5,552,557	5,553,753	+	*glgC*	Glucose-1-phosphate adenylyltransferase	Glycogen biosynthesis
Chromosome	5,552,416	5,551,247	-	*glgA*	Glycogen synthase, ADP-glucose transglucosylase	Glycogen biosynthesis
Chromosome	2,400,678	2,398,480	-	*glgB*	1,4-alpha-glucan (glycogen) branching enzyme	Glycogen biosynthesis
Chromosome	2,402,696	2,400,678	-	*glgE*	Putative glucanase	Glycogen degradation
Chromosome	2,402,921	2,405,512	+	*glgP*	Glycogen phosphorylase	Glycogen degradation
Chromosome	294,183	292,977	-	*glgX*	Glycogen debranching enzyme	Glycogen degradation
Chromosome	6,039,652	6,041,283	+	*pgm*	Phosphoglucomutase	Glycogen degradation
Chromosome	1,654,022	1,654,951	+	*ppk1*	Polyphosphate kinase 2	Polyphosphate metabolism
Chromosome	1,691,319	1,690,459	-	*ppk2*	Polyphosphate kinase 2	Polyphosphate metabolism
Chromosome	2,218,391	2,219,335	+	*ppk3*	Polyphosphate kinase 2	Polyphosphate metabolism
Chromosome	6,130,051	6,127,847	-	*ppk4*	Polyphosphate kinase	Polyphosphate metabolism
Chromosome	1,732,260	1,733,129	+	*ppx1*	Exopolyphosphatase	Polyphosphate metabolism
Chromosome	5,347,847	5,348,791	+	*ppx2*	Exopolyphosphatase	Polyphosphate metabolism
Chromosome	2,268,854	2,270,167	+	*aft1*	Wax ester synthase/acyl-CoA:diacylglycerol acyltransferase	Triacylglycerol biosynthesis
Chromosome	3,330,846	3,329,479	-	*aft2*	Wax ester synthase/acyl-CoA:diacylglycerol acyltransferase	Triacylglycerol biosynthesis
Chromosome	3,534,989	3,536,398	+	*aft3*	Wax ester synthase/acyl-CoA:diacylglycerol acyltransferase	Triacylglycerol biosynthesis
Chromosome	3,537,839	3,536,439	-	*aft4*	Wax ester synthase/acyl-CoA:diacylglycerol acyltransferase	Triacylglycerol biosynthesis
Chromosome	4,910,843	4,909,449	-	*aft5*	Wax ester synthase/acyl-CoA:diacylglycerol acyltransferase	Triacylglycerol biosynthesis
Chromosome	5,960,187	5,961,608	+	*aft6*	Wax ester synthase/acyl-CoA:diacylglycerol acyltransferase	Triacylglycerol biosynthesis
Chromosome	6,377,931	6,376,519	-	*aft7*	Wax ester synthase/acyl-CoA:diacylglycerol acyltransferase	Triacylglycerol biosynthesis
Chromosome	223,037	224,734	+	*phaC1*	Polyhydroxyalkanoic acid synthase	Polyhydroxyalkanoic acid synthesys
Chromosome	496,107	495,013	-	*phaC2*	Polyhydroxyalkanoic acid synthase	Polyhydroxyalkanoic acid synthesys
Chromosome	498,367	496,676	-	*phaC3*	Polyhydroxyalkanoic acid synthase	Polyhydroxyalkanoic acid synthesys

Chromosome	757,362	756,295	-	*phaC4*	Polyhydroxyalkanoic acid synthase	Polyhydroxyalkanoic acid synthesys
Chromosome	2,922,022	2,925,006	+	*phaC5*	Polyhydroxyalkanoic acid synthase	Polyhydroxyalkanoic acid synthesys
**Lithoautotrophy**						
Chromosome	921,751	922,080	+	*hypA*	[NiFe] hydrogenase nickel incorporation protein	Hydrogenase
Chromosome	922,086	922,853	+	*hypB*	[NiFe] hydrogenase nickel incorporation-associated protein	Hydrogenase
Chromosome	923,004	924,059	+	*hyaA*	Uptake hydrogenase small subunit	Hydrogenase
Chromosome	924,140	925,882	+	*hyaB*	Uptake hydrogenase large subunit	Hydrogenase
Chromosome	929,598	930,098	+	*hypD1*	Hydrogenase maturation protease	Hydrogenase
Chromosome	987,965	986,835	-	*hypD2*	[NiFe] hydrogenase metallocenter assembly protein	Hydrogenase
Chromosome	930,102	930,362	+	*hypC1*	Hydrogenase assembly chaperone	Hydrogenase
Chromosome	988,251	987,979	-	*hypC2*	[NiFe] hydrogenase metallocenter assembly protein	Hydrogenase
Chromosome	989,377	988,277	-	*hypE*	[NiFe] hydrogenase metallocenter assembly protein	Hydrogenase
Chromosome	993,154	990,848	-	*hypF*	[NiFe] hydrogenase metallocenter assembly protein	Hydrogenase
Chromosome	1,446,069	1,445,215	-	*coxM1*	Carbon monoxide dehydrogenase medium subunit	Carbon monoxide utilization
Chromosome	3,082,256	3,081,351	-	*coxM2*	Carbon monoxide dehydrogenase medium subunit	Carbon monoxide utilization
Chromosome	1,448,441	1,446,066	-	*coxL1*	Carbon monoxide dehydrogenase large chain	Carbon monoxide utilization
Chromosome	3,080,767	3,078,365	-	*coxL2*	Carbon monoxide dehydrogenase large chain	Carbon monoxide utilization
Chromosome	1,448,971	1,448,438	-	*coxS1*	Carbon monoxide dehydrogenase small chain	Carbon monoxide utilization
Chromosome	3,081,348	3,080,767	-	*coxS2*	Carbon monoxide dehydrogenase small chain	Carbon monoxide utilization
Chromosome	1,450,183	1,449,068	-	*coxE1*	Carbon monoxide dehydrogenase E protein	Carbon monoxide utilization
Chromosome	2,539,619	2,541,079	+	*coxE2*	Carbon monoxide dehydrogenase E protein	Carbon monoxide utilization
Chromosome	3,075,906	3,074,692	-	*coxE3*	Carbon monoxide dehydrogenase E protein	Carbon monoxide utilization
Chromosome	5,645,254	5,646,783	+	*coxE4*	Carbon monoxide dehydrogenase E protein	Carbon monoxide utilization
pRWAY02	326,248	327,774	+	*coxE*	Carbon monoxide dehydrogenase E protein	Carbon monoxide utilization
Chromosome	1,451,082	1,450,189	-	*coxD1*	Carbon monoxide oxidation accessory protein	Carbon monoxide utilization
Chromosome	3,077,445	3,076,558	-	*coxD2*	Carbon monoxide oxidation accessory protein	Carbon monoxide utilization
Chromosome	5,644,145	5,645,251	+	*coxD3*	Carbon monoxide oxidation accessory protein	Carbon monoxide utilization
pRWAY02	325,024	326,244	+	*coxD*	Carbon monoxide dehydrogenase D protein	
Chromosome	3,076,544	3,075,903	-	*coxG1*	Carbon monoxide oxidation accessory protein	Carbon monoxide utilization
Chromosome	3,290,423	3,289,977	-	*coxG2*	Carbon monoxide oxidation accessory protein	Carbon monoxide utilization
Chromosome	3,078,335	3,077,442	-	*coxF*	Aerobic carbon monoxide dehydrogenase molybdenum cofactor insertion protein	Carbon monoxide utilization
Chromosome	3,083,474	3,082,332	-	*coxI*	Xanthine and CO dehydrogenases maturation factor	Carbon monoxide utilization
Chromosome	794,324	794,956	+	*cynT*	Carbonic anhydrase	Carbon dioxide
Chromosome	4,365,422	4,367,500	+		Carbonic anhydrase	Carbon dioxide

Chromosome	6,032,559	6,032,065	-		Carbonic anhydrase	Carbon dioxide
Chromosome	6,136,323	6,136,844	+		Carbonic anhydrase	Carbon dioxide
pRWAY03	189,067	186,755	-		Carbonic anhydrase	Carbon dioxide
**Cold shock response**						
Chromosome	447,655	445,517	-		Cold shock protein	Cold shock response
Chromosome	715,068	715,271	+	*cspA*	Cold shock protein CspA	Cold shock response
Chromosome	911,575	912,108	+		Cold shock protein	Cold shock response
Chromosome	1,433,797	1,434,207	+	*cspC*	Cold shock protein CspC	Cold shock response
Chromosome	3,282,441	3,282,686	+	*cspA*	Cold shock protein CspA	Cold shock response
Chromosome	3,767,091	3,766,795	-	*cspA*	Cold shock protein CspA	Cold shock response
Chromosome	5,256,431	5,256,634	+	*cspA*	Cold shock protein CspA	Cold shock response
Chromosome	6,437,612	6,437,815	+	*cspA*	Cold shock protein CspA	Cold shock response
pRWAY01	220,873	221,223	+	*cspA*	Cold shock protein CspA	Cold shock response
pRWAY01	828,313	828,909	+		Cold shock protein	Cold shock response
**Compatible solutes**						
Chromosome	78,433	81,045	+		Trehalose-6-phosphate phosphatase	Compatible solutes
Chromosome	820,735	818,291	-		Trehalose-6-phosphate phosphatase	Compatible solutes
Chromosome	932,586	930,382	-	*treS*	Trehalose synthase	Compatible solutes
Chromosome	1,062,110	1,060,518	-		Alpha,alpha-trehalose-phosphate synthase [UDP-forming]	Compatible solutes
Chromosome	1,192,837	1,194,078	+	*proP*	L-Proline/Glycine betaine transporter ProP	Compatible solutes
Chromosome	1,334,567	1,335,913	+		Proline/betaine transporter	Compatible solutes
Chromosome	2,132,772	2,131,186	-	*betA*	Choline dehydrogenase	Compatible solutes
Chromosome	2,529,380	2,528,982	-	*ectC*	L-ectoine synthase	Compatible solutes
Chromosome	2,530,677	2,529,400	-	*ectB*	Diaminobutyrate-pyruvate aminotransferase	Compatible solutes
Chromosome	2,531,103	2,530,705	-	*ectA*	L-2,4-diaminobutyric acid acetyltransferase	Compatible solutes
Chromosome	2,535,912	2,534,443	-	*proP*	L-Proline/Glycine betaine transporter ProP	Compatible solutes
Chromosome	2,697,119	2,696,127	-	*proX*	L-proline glycine betaine binding ABC transporter protein ProX / Osmotic adaptation	Compatible solutes
Chromosome	2,699,097	2,697,886	-	*proV*	L-proline glycine betaine ABC transport system permease protein ProV	Compatible solutes
Chromosome	2,699,746	2,699,090	-		Glycine betaine/carnitine/choline ABC transporter, permease protein	Compatible solutes
Chromosome	2,809,617	2,807,914	-	*treZ*	Malto-oligosyltrehalose trehalohydrolase	Compatible solutes
Chromosome	2,816,988	2,814,601	-	*treY*	Malto-oligosyltrehalose synthase	Compatible solutes
Chromosome	3,318,110	3,316,680	-		Alpha,alpha-trehalose-phosphate synthase [UDP-forming]	Compatible solutes
Chromosome	4,017,746	4,016,421	-	*proP*	L-Proline/Glycine betaine transporter ProP	Compatible solutes

Chromosome	4,447,309	4,445,984	-	*proP*	L-Proline/Glycine betaine transporter ProP	Compatible solutes
Chromosome	4,625,092	4,623,719	-	*proP*	L-Proline/Glycine betaine transporter ProP	Compatible solutes
Chromosome	4,746,029	4,747,372	+	*proP*	L-Proline/Glycine betaine transporter ProP	Compatible solutes
Chromosome	4,844,175	4,845,503	+	*proP*	L-Proline/Glycine betaine transporter ProP	Compatible solutes
Chromosome	5,164,191	5,162,467	-	*betT*	High-affinity choline uptake protein BetT	Compatible solutes
Chromosome	5,241,917	5,243,590	+	*betT*	High-affinity choline uptake protein BetT	Compatible solutes
Chromosome	5,464,351	5,465,793	+	*betB*	Betaine aldehyde dehydrogenase	Compatible solutes
Chromosome	5,579,197	5,578,478	-	*proZ*	Glycine betaine ABC transport system permease protein	Compatible solutes
Chromosome	5,579,844	5,579,194	-	*proW*	L-proline glycine betaine ABC transport system permease protein ProW	Compatible solutes
Chromosome	5,581,006	5,579,849	-	*proV*	L-proline glycine betaine ABC transport system permease protein ProV	Compatible solutes
Chromosome	5,581,994	5,581,017	-	*proX*	L-proline glycine betaine binding ABC transporter protein ProX	Compatible solutes
Chromosome	5,582,253	5,583,119	+	*lpqZ*	Substrate-binding region of ABC-type glycine betaine transport system	Compatible solutes
Chromosome	5,624,334	5,626,169	+	*treS*	Trehalose synthase	Compatible solutes
Chromosome	5,670,137	5,671,612	+	*betA*	Choline dehydrogenase	Compatible solutes
Chromosome	6,310,486	6,311,850	+	*proP*	L-Proline/Glycine betaine transporter ProP	Compatible solutes
pRWAY01	147,880	149,148	+	*ectB*	Diaminobutyrate-pyruvate aminotransferase	Compatible solutes
pRWAY01	244,656	248,291	+		Trehalose-6-phosphate phosphatase	Compatible solutes
pRWAY01	544,245	542,944	-	*proP*	L-Proline/Glycine betaine transporter ProP	Compatible solutes
pRWAY01	768,642	769,964	+	*proP*	L-Proline/Glycine betaine transporter ProP	Compatible solutes
pRWAY01	797,450	798,835	+	*proP*	L-Proline/Glycine betaine transporter ProP	Compatible solutes

Table S8. Genetic islands (GIs) identified in *Rhodococcus* sp. WAY2

Replicon	GI coordinates	GI length (bp)	GC%	Prediction method	Gene start	Gene end	Strand	Product
Chromosome	72,878 .. 77,309	4,431	66.49	SIGI-HMM	72,878	73,279	-	Hypothetical protein
					73,582	75,363	-	Biotin carboxylase of acetyl-CoA carboxylase
					75,391	75,690	-	Hypothetical protein
					75,687	77,309	-	Methylcrotonyl-CoA carboxylase carboxyl transferase subunit
Chromosome	149,687 .. 154,102	4,415	58.44	SIGI-HMM	148,428	149,690	+	Methylmalonate-semialdehyde dehydrogenase
					149,687	150,157	+	Hypothetical protein
					150,330	151,199	+	3-oxoacyl-[acyl-carrier protein] reductase
					152,189	152,311	-	Hypothetical protein
					152,600	154,102	+	Acetoacetyl-CoA synthetase
Chromosome	778,268 .. 785,448	7,180	64.15	IslandPath-DIMOB	778,268	778,801	+	Hypothetical protein
					778,798	779,220	+	Hypothetical protein
					779,291	780,802	+	Lysyl-tRNA synthetase (class II)
					780,942	781,295	+	Histone protein Lsr2
					782,088	782,339	-	Hypothetical protein
					782,328	784,802	+	ATP-dependent Clp protease
					784,867	785,448	-	Hypothetical protein
Chromosome	1,333,138 .. 1,350,980	17,842	64.01	IslandPath-DIMOB	1,333,138	1,334,163	+	Predicted aminoglycoside phosphotransferase
					1,334,372	1,334,491	-	Hypothetical protein
					1,334,567	1,335,913	+	Proline/betaine transporter
					1,336,229	1,336,360	+	Hypothetical protein
					1,336,507	1,337,889	+	Membrane transport protein
					1,337,991	1,338,803	-	Transcriptional regulator, TetR family
					1,339,617	1,340,255	-	Transcriptional regulator, MerR family
					1,340,403	1,341,620	+	3-ketoacyl-CoA thiolase
					1,341,641	1,342,372	+	3-oxoacyl-[acyl-carrier protein] reductase
					1,342,404	1,344,200	+	3-methylmercaptopropionyl-CoA dehydrogenase (DmdC)
					1,344,216	1,344,977	+	3-oxoacyl-[acyl-carrier protein] reductase
					1,345,058	1,346,808	-	Mobile element protein
					1,347,469	1,347,636	-	Hypothetical protein
					1,347,667	1,347,903	+	Nodulation protein N
					1,348,043	1,348,180	+	Hypothetical protein
					1,348,486	1,348,623	+	Hypothetical protein
					1,349,154	1,349,453	-	Hypothetical protein

					1,349,434	1,349,562	+	Hypothetical protein
					1,349,578	1,349,712	+	Hypothetical protein
					1,349,835	1,350,980	-	Butyryl-CoA dehydrogenase
Chromosome	1,564,659 .. 1,597,326	32,667	68.15	IslandPath-DIMOB	1,564,659	1,566,191	+	Peptide synthetase
					1,566,351	1,575,512	+	Hypothetical protein
					1,575,544	1,580,037	+	Polyketide synthase
					1,580,034	1,580,876	+	3-hydroxybutyryl-CoA dehydrogenase
					1,580,911	1,592,397	+	Non-ribosomal peptide synthetase
					1,592,460	1,593,419	-	Probable 85 complex protein, A85 antigen family
					1,593,511	1,593,852	+	Possible inner membrane protein
					1,593,908	1,594,915	+	F420-dependent glucose-6-phosphate dehydrogenase
					1,595,045	1,596,358	+	Mobile element protein
					1,596,412	1,597,326	-	Hypothetical protein
Chromosome	1,885,277 .. 1,908,080	22,803	64.04	IslandPath-DIMOB	1,885,277	1,885,531	-	Hypothetical protein
					1,885,667	1,886,146	-	Cytosine deaminase
					1,886,150	1,886,959	-	Transcriptional regulator, TetR family
					1,887,132	1,888,481	+	Hypothetical protein
					1,888,478	1,889,092	+	Putative secreted protein
					1,889,155	1,889,643	-	Conserved Hypothetical protein SCF43.06
					1,889,801	1,890,250	-	Hypothetical protein
					1,890,300	1,890,455	+	Hypothetical protein
					1,890,553	1,891,770	+	Hypothetical protein
					1,891,799	1,892,092	-	Sporulation regulatory protein WhiD
					1,892,476	1,893,723	+	Alpha/beta hydrolase fold-containing protein
					1,893,797	1,895,632	-	3-methylmercaptopropionyl-CoA dehydrogenase (DmdC)
					1,895,992	1,897,689	-	putative cholesterol oxidase
					1,897,803	1,897,961	+	Hypothetical protein
					1,898,581	1,900,590	-	Possible electron transfer protein FdxB
					1,900,680	1,900,835	+	Hypothetical protein
					1,901,225	1,901,419	-	Hypothetical protein
					1,901,495	1,901,707	+	Hypothetical protein
					1,901,731	1,902,981	+	Hypothetical protein
					1,903,219	1,904,961	+	Acyl-CoA dehydrogenase
					1,904,998	1,905,117	-	Hypothetical protein
					1,905,118	1,905,510	+	Aldehyde dehydrogenase
					1,905,691	1,906,116	+	Putative membrane protein
					1,906,549	1,907,031	+	Transcriptional regulator, AraC family
					1,907,612	1,907,746	-	Hypothetical protein
					1,907,817	1,908,080	+	Hypothetical protein
Chromosome	2,188,167 .. 2,195,802	7,635	59.57	SIGI-HMM	2,188,167	2,188,541	-	CoA-binding domain protein
					2,190,033	2,190,596	-	Hypothetical protein
					2,190,998	2,192,689	-	ATP/GTP binding protein

					2,193,626	2,193,895	-	Hypothetical protein
					2,194,342	2,195,802	-	Hypothetical protein
Chromosome	2,906,194 .. 2,910,316	4,122	65.26	IslandPath-DIMOB	2,906,194	2,906,610	-	RecA/RadA recombinase
					2,906,701	2,906,826	+	Hypothetical protein
					2,907,064	2,907,597	+	Translation initiation factor 3
					2,907,657	2,907,851	+	LSU ribosomal protein L35p
					2,907,916	2,908,305	+	LSU ribosomal protein L20p
					2,908,344	2,909,153	+	rRNA methylase
					2,909,234	2,910,316	+	Phenylalanyl-tRNA synthetase alpha chain
Chromosome	3,157,288 .. 3,200,524	43,237	62.75	IslandPath-DIMOB, SIGI-HMM	3,157,288	3,157,683	-	Nitrilotriacetate monooxygenase component B
					3,157,888	3,158,643	-	Creatinine amidohydrolase
					3,158,652	3,160,535	-	Long-chain-fatty-acid--CoA ligase
					3,160,562	3,161,788	-	Branched-chain amino acid ABC transporter, amino acid-binding protein
					3,161,945	3,162,664	-	Branched-chain amino acid transport ATP-binding protein LivF
					3,162,651	3,163,436	-	Branched-chain amino acid transport ATP-binding protein LivG
					3,163,436	3,164,386	-	Branched-chain amino acid transport system permease protein LivM
					3,164,422	3,165,288	-	High-affinity branched-chain amino acid transport system Permease protein LivH
					3,165,299	3,166,093	-	Enoyl-CoA hydratase
					3,166,097	3,166,963	-	Enoyl-CoA hydratase
					3,166,960	3,168,153	-	Luciferase family protein
					3,168,507	3,169,094	+	Transcriptional regulator, TetR family
					3,169,453	3,169,791	+	FIG00828522: Hypothetical protein
					3,169,826	3,170,653	-	Formate dehydrogenase chain D
					3,170,653	3,172,956	-	Putative formate dehydrogenase oxidoreductase protein
					3,173,038	3,173,952	-	Hydrogen peroxide-inducible genes activator
					3,174,286	3,177,594	+	Hypothetical protein
					3,177,626	3,178,207	-	Hypothetical protein
					3,178,433	3,179,152	-	transcriptional regulator, LuxR family
					3,179,429	3,179,647	+	Hypothetical protein
					3,179,696	3,179,812	+	Hypothetical protein
					3,179,816	3,181,612	+	Mobile element protein
					3,182,109	3,182,579	+	Hypothetical protein
					3,182,591	3,183,394	+	Possible flavodoxin (beta-lactamase-like)
					3,183,526	3,184,392	-	Probable 5-valerolactone hydrolase
					3,184,660	3,186,291	-	Cyclohexanone monooxygenase
					3,186,338	3,187,954	-	Long-chain-fatty-acid--CoA ligase
					3,187,951	3,189,024	-	Probable acyl-CoA dehydrogenase

					3,189,027	3,190,211	-	Butyryl-CoA dehydrogenase
					3,190,213	3,191,055	-	Enoyl-CoA hydratase
					3,191,598	3,192,848	+	Possible transcriptional regulator
					3,193,250	3,193,591	+	Hypothetical protein
					3,193,836	3,194,327	+	Hypothetical protein
					3,194,740	3,195,585	+	Oxetanocin A resistance protein
					3,195,638	3,196,075	+	Hypothetical protein
					3,196,122	3,196,328	-	Hypothetical protein
					3,196,799	3,197,950	+	Epoxide hydrolase
					3,198,018	3,199,058	-	Alcohol dehydrogenase
					3,199,167	3,199,628	-	Transcriptional regulator, MerR family
					3,199,829	3,200,524	-	Transcriptional regulator, GntR family
Chromosome	4,251,782 .. 4,277,261	25,479	65.16	IslandPath-DIMOB	4,251,782	4,252,654	+	3-oxoacyl-[acyl-carrier protein] reductase
					4,252,669	4,252,902	+	Probable ferredoxin FdxD
					4,252,909	4,254,138	+	Putative cytochrome P450 hydroxylase
					4,254,334	4,254,912	+	Transcriptional regulator, TetR family
					4,255,378	4,256,436	+	Methionine ABC transporter ATP-binding protein
					4,256,514	4,257,326	+	Conserved Hypothetical integral membrane protein YrbE1A
					4,257,328	4,258,188	+	ABC-type transport system involved in resistance to organic solvents, permease component
					4,258,250	4,259,281	+	Mammalian cell entry related domain protein
					4,259,299	4,260,291	+	MCE-family protein Mce1B
					4,260,324	4,261,304	+	Virulence factor mce family protein
					4,261,306	4,262,337	+	MCE-family protein Mce1D
					4,262,339	4,263,346	+	Possible mce-family lipoprotein lprl
					4,263,343	4,264,302	+	Putative Mce family protein
					4,264,372	4,264,995	+	Hypothetical protein
					4,265,040	4,265,642	-	Possible transcriptional regulator, TetR family
					4,265,714	4,266,895	+	Large subunit aromatic oxygenase
					4,266,892	4,267,653	+	Hypothetical protein
					4,267,695	4,268,369	+	Hypothetical protein
					4,268,366	4,269,598	+	Alpha-methylacyl-CoA racemase
					4,269,834	4,271,375	+	O-succinylbenzoic acid--CoA ligase
					4,271,440	4,272,891	+	4-hydroxybenzoate transporter
					4,272,895	4,275,024	+	Hypothetical protein
					4,275,021	4,277,261	+	Formate dehydrogenase-O, major subunit
Chromosome	4,685,575 .. 4,719,883	34,308	63.37	IslandPath-DIMOB	4,685,575	4,686,354	+	amidohydrolase family protein
					4,686,439	4,687,224	+	Enoyl-CoA hydratase
					4,687,262	4,688,017	+	2-deoxy-D-gluconate 3-dehydrogenase
					4,688,118	4,689,248	+	3-ketoacyl-CoA thiolase
					4,689,368	4,690,132	+	3-hydroxyacyl-CoA dehydrogenase
					4,690,129	4,690,257	-	Hypothetical protein

					4,690,333	4,691,124	+	Enoyl-CoA hydratase
					4,691,211	4,692,332	-	Alpha-methylacyl-CoA racemase
					4,692,414	4,693,082	-	Transcriptional regulator, TetR family
					4,693,174	4,694,385	-	Isobutyryl-CoA dehydrogenase
					4,694,637	4,696,043	+	4-hydroxybenzoate transporter
					4,696,166	4,697,059	+	L-malyl-CoA/beta-methylmalyl-CoA lyase
					4,697,158	4,697,910	-	Enoyl-CoA hydratase
					4,698,021	4,698,191	-	Alcohol dehydrogenase
					4,698,308	4,700,092	-	Mobile element protein
					4,700,116	4,700,901	-	Hypothetical protein
					4,701,495	4,702,571	+	F420-dependent N(5),N(10)-methylenetetrahydromethanopterin reductase
					4,702,954	4,704,252	+	4-hydroxybenzoate transporter
					4,704,289	4,704,495	+	Hypothetical protein
					4,704,503	4,705,213	+	Transcriptional regulator, TetR family
					4,705,389	4,706,855	-	Aldehyde dehydrogenase
					4,707,454	4,708,587	+	2,4-dienoyl-CoA reductase [NADPH]
					4,708,680	4,709,414	+	Hypothetical protein
					4,709,722	4,710,687	+	Alcohol dehydrogenase
					4,710,700	4,711,257	+	Gamma-BHC dehydrochlorinase
					4,711,397	4,712,143	-	Hypothetical protein
					4,712,158	4,713,642	-	Drug resistance transporter EmrB/QacA subfamily
					4,713,685	4,715,094	-	Nitrilotriacetate monooxygenase component A
					4,715,299	4,715,889	+	Transcriptional regulator, TetR family
					4,715,935	4,716,048	+	Hypothetical protein
					4,716,578	4,717,723	+	Transporter, putative
					4,717,817	4,718,830	-	Quinone oxidoreductase
					4,718,894	4,719,883	-	Vanillate O-demethylase oxidoreductase
Chromosome	4,795,058 .. 4,799,299	4,241	66.71	SIGI-HMM	4,795,058	4,796,110	-	Transposase
					4,796,159	4,797,073	-	Probable taurine catabolism dioxygenase
					4,797,112	4,798,086	-	Quinone oxidoreductase
					4,798,289	4,799,299	+	Transcriptional regulator, AraC family
Chromosome	4,868,035 .. 4,902,842	34,807	66.45	IslandPath-DIMOB	4,868,035	4,868,562	-	Putative small integral membrane protein
					4,868,603	4,871,473	-	High-affinity carbon uptake protein Hat/HatR
					4,871,472	4,871,618	+	Hypothetical protein
					4,871,688	4,871,843	-	Fic protein family protein
					4,872,371	4,873,846	+	Putative membrane transport protein
					4,873,855	4,874,487	+	Transcriptional regulator, TetR family
					4,874,624	4,875,523	+	Probable hydrolase
					4,875,770	4,876,969	-	Fic family protein
					4,877,143	4,877,892	+	Hypothetical protein
					4,877,933	4,880,113	-	Arsenical pump-driving ATPase

					4,880,134	4,880,535	-	Arsenate reductase
					4,880,566	4,880,958	-	Arsenate reductase
					4,881,006	4,882,118	-	Arsenate reductase
					4,882,115	4,882,474	-	Arsenical resistance operon repressor
					4,882,593	4,882,937	+	Transcriptional regulator, ArsR family
					4,882,928	4,883,638	+	Putative membrane protein
					4,883,707	4,884,702	-	Arsenical-resistance protein ACR3
					4,884,801	4,885,550	-	Mercuric resistance operon regulatory protein
					4,885,653	4,886,507	-	Possible phosphinothricin N-acetyltransferase
					4,886,759	4,887,949	+	Probable secreted protein
					4,888,179	4,888,316	+	Hypothetical protein
					4,888,487	4,889,416	+	ABC transporter, ATP-binding component
					4,889,413	4,890,225	+	ABC transporter, family 2
					4,890,424	4,890,633	+	Hypothetical protein
					4,890,674	4,891,333	+	RNA polymerase sigma factor SigZ
					4,891,523	4,891,636	+	Hypothetical protein
					4,891,720	4,891,833	-	Hypothetical protein
					4,892,075	4,892,293	+	Hypothetical protein
					4,892,372	4,893,517	-	Chaperone protein DnaJ
					4,893,524	4,893,682	-	Hypothetical protein
					4,894,633	4,894,806	+	Hypothetical protein
					4,894,877	4,896,373	+	Adenylate cyclase
					4,896,494	4,897,702	-	Possible linoleoyl-coa desaturase (delta(6)-desaturase)
					4,897,870	4,898,676	-	Metal cation transporting ATPase, P-type ATPase superfamily
					4,898,670	4,899,122	-	Metal cation transporting ATPase, P-type ATPase superfamily
					4,899,810	4,901,615	+	Mobile element protein
					4,901,793	4,902,122	-	Hypothetical protein
					4,902,213	4,902,842	-	DNA-binding response regulator KdpE
Chromosome	4,947,132 .. 4,965,372	18,240	57.42	SIGI-HMM	4,947,132	4,947,296	-	Hypothetical protein
					4,947,981	4,949,444	+	Hypothetical protein
					4,949,983	4,952,109	-	O-antigen acetylase
					4,952,747	4,952,914	-	Hypothetical protein
					4,953,464	4,953,616	+	Hypothetical protein
					4,953,761	4,954,084	+	Hypothetical protein
					4,955,166	4,956,686	+	Undecaprenyl-phosphate galactosephosphotransferase
					4,957,408	4,958,685	+	Hypothetical protein
					4,960,656	4,961,972	+	Coenzyme F420-dependent oxidoreductase
					4,964,305	4,965,372	-	Alpha-D-GlcNAc alpha-1,2-L-rhamnosyltransferase
Chromosome	6,267,813 .. 6,289,319	21,506	66.17	IslandPath-DIMOB	6,267,813	6,268,337	-	Acetyltransferase, GNAT family

					6,268,539	6,269,117	+	CDP-diacylglycerol--glycerol-3-phosphate 3-phosphatidyltransferase
					6,269,114	6,269,614	+	C-terminal domain of CinA type S
					6,269,685	6,270,047	+	Hypothetical protein
					6,270,239	6,271,024	+	Phage shock protein A (IM30), suppresses sigma54-dependent transcription
					6,271,125	6,271,871	+	Alanine-rich, phage-related, membrane protein
					6,272,063	6,272,251	+	Hypothetical protein
					6,272,268	6,272,984	+	Hypothetical protein
					6,273,020	6,273,166	+	Hypothetical protein
					6,273,198	6,273,821	+	Probable transcriptional regulator, TetR family
					6,273,818	6,275,350	+	Glycerol-3-phosphate dehydrogenase
					6,275,371	6,276,525	+	Alanine rich transferase
					6,276,561	6,276,755	+	Hypothetical protein
					6,277,018	6,278,061	+	RecA protein
					6,278,072	6,278,587	+	Regulatory protein RecX
					6,278,649	6,279,551	-	Glutamate permease
					6,279,548	6,280,228	-	Putative glutamate transporter
					6,280,317	6,281,150	-	Glutamate-binding protein of ABC transporter system
					6,281,239	6,281,967	-	Putative glutamate uptake system ATP-binding protein
					6,282,176	6,283,693	+	tRNA-i(6)A37 methylthiotransferase
					6,283,690	6,284,295	+	Possible membrane protein Rv2732c
					6,284,314	6,285,726	-	ATPase involved in DNA repair
					6,285,893	6,286,618	+	Hypothetical protein
					6,286,615	6,287,544	+	tRNA dimethylallyltransferase
					6,287,541	6,288,389	+	Possible rRNA methylase
					6,288,450	6,289,319	+	Diaminopimelate epimerase
Chromosome	6,568,016 .. 6,574,590	6,574	65.48	IslandPath-DIMOB	6,568,016	6,568,684	+	Probable oxidoreductase, short chain dehydrogenase/ reductase family
					6,568,857	6,569,198	+	Hypothetical protein
					6,569,202	6,569,852	-	Maleylpyruvate isomerase, mycothiol-dependent
					6,570,023	6,570,580	+	TolA protein
					6,570,609	6,571,286	+	TolA protein
					6,571,283	6,571,426	-	Transposase and inactivated derivatives-like
					6,571,498	6,572,100	-	Transcriptional regulator, TetR family
					6,572,376	6,573,608	+	Beta-lactamase class C and other penicillin binding proteins
					6,573,610	6,574,590	-	Hypothetical protein
pRWAY01	267,189 .. 276,334	9,145	58.12	IslandPath-DIMOB, SIGI-HMM	267,189	267,866	+	Phosphoserine phosphatase
					268,257	268,550	+	Hypothetical protein
					268,695	269,087	+	Hypothetical protein
					269,220	269,606	+	Hypothetical protein
					269,690	270,148	+	Hypothetical protein

					270,253	271,698	+	Nicotinamide phosphoribosyltransferase
					271,814	271,936	-	Hypothetical protein
					272,619	272,741	+	Transposase, mutator type
					272,982	274,337	-	Coenzyme F420-0:L-glutamate ligase
					274,586	275,521	-	2-phospho-L-lactate guanylyltransferase
					275,534	276,334	-	6-phosphogluconolactonase
					276,331	278,802	-	7,8-didemethyl-8-hydroxy-5-deazariboflavin synthase subunit 1
pRWAY01	344,870 .. 365,930	21,060	69.67	IslandPath-DIMOB	344,870	345,668	-	ABC-type Fe3+-siderophore transport system, ATPase component
					345,712	346,824	-	ABC-type Fe3+-siderophore transport system, permease 2 component
					346,821	347,852	-	ABC-type Fe3+-siderophore transport system, permease component
					348,026	348,595	-	Possible transcriptional regulator
					348,636	348,788	-	Hypothetical protein
					348,805	349,008	+	Hypothetical protein
					349,011	349,529	+	Hypothetical protein
					349,559	350,980	-	Phage peptidoglycan binding endopeptidase
					351,144	351,950	-	Cytochrome c-type biogenesis protein CcdA
					351,947	352,552	-	Thiol:disulfide oxidoreductase related to ResA
					352,549	353,154	-	Possible thioredoxin
					353,189	353,401	+	Hypothetical protein
					353,515	353,895	+	Transcriptional regulator, MecI family
					353,892	354,854	+	Peptidase M48, Ste24p precursor
					354,970	355,359	+	Hypothetical protein
					355,466	356,578	+	Cysteine synthase
					356,578	357,867	+	Probable conserved integral membrane transport protein
					357,994	359,541	+	Multicopper oxidase
					359,640	360,458	+	Hypothetical protein
					360,465	362,405	+	Copper resistance protein CopC / Copper resistance protein CopD
					362,530	362,979	+	Hypothetical protein
					363,075	363,752	+	Putative lipoprotein
					363,874	365,109	+	Probable conserved lipoprotein LppS
					365,106	365,930	+	DedA family protein paralog
pRWAY01	381,580 .. 399,369	17,789	62.01	IslandPath-DIMOB	381,580	381,696	+	Hypothetical protein
					382,075	382,455	+	Hypothetical protein
					382,602	382,748	+	Hypothetical protein
					382,849	383,562	+	Plasmid partitioning protein ParA
					383,562	384,428	+	Chromosome (plasmid) partitioning protein ParB
					384,807	385,478	+	Mobile element protein

					385,552	385,695	-	Hypothetical protein
					385,709	386,551	+	Hypothetical protein
					386,981	387,541	+	Pentapeptide repeat family protein
					387,884	388,036	+	Hypothetical protein
					388,042	388,308	+	Hypothetical protein
					388,376	388,489	-	Hypothetical protein
					388,492	389,076	-	Hypothetical protein
					389,492	390,688	-	Phage excisionase
					390,894	391,049	-	Hypothetical protein
					391,340	392,128	-	Chromosome (plasmid) partitioning protein ParA
					392,467	392,634	-	Hypothetical protein
					393,021	393,881	-	Metallo-beta-lactamase superfamily protein
					393,878	395,797	-	Hypothetical protein
					395,956	396,084	-	Hypothetical protein
					396,344	397,195	+	O-Methyltransferase involved in polyketide biosynthesis
					397,173	398,156	-	Hypothetical protein
					398,153	398,713	-	Cinorf13 protein
					398,728	399,162	-	Hypothetical protein
					399,169	399,369	-	Hypothetical protein
					453,960	454,526	-	Resolvase
					455,205	455,396	+	Hypothetical protein
					456,542	456,727	-	Hypothetical protein
					456,782	457,060	+	Hypothetical protein
					457,292	460,009	+	Type II restriction enzyme, methylase subunit YeeA
pRWAY01	453,960 .. 466,850	12,890	59.95	IslandPath-DIMOB	460,006	461,991	+	DNA helicase, restriction/modification system component YeeB
					461,988	463,181	+	YeeC-like protein
					464,971	465,741	-	Hypothetical protein
					466,199	466,459	-	Hypothetical protein
					466,554	466,850	-	Hypothetical protein
					483,867	484,511	-	Organomercurial lyase
					484,583	485,026	-	Regulatory protein, MerR
					485,045	486,484	-	Mercuric ion reductase
					486,613	487,002	+	Mercuric resistance operon regulatory protein
					487,002	487,301	+	Cytochrome c-type biogenesis protein DsbD, protein-Disulfide reductase
pRWAY01	483,867 .. 491,147	7,280	71.44	SIGI-HMM	487,298	487,804	+	Hypothetical protein
					487,801	488,697	+	Cytochrome c biogenesis protein, transmembrane region
					488,694	490,073	+	PF00070 family, FAD-dependent NAD(P)-disulphide oxidoreductase
					490,191	491,147	+	Putative integrase/recombinase
pRWAY01	657,119 .. 692,928	35,809	66.09	IslandPath-DIMOB	657,119	658,012	+	Short-chain dehydrogenase/reductase SDR

					658,125	658,274	-	Hypothetical protein
					658,285	658,521	-	Probable transposase for insertion sequence element IS1533
					658,804	659,511	-	Alcohol dehydrogenase
					659,566	659,949	+	Hypothetical protein
					659,922	661,118	-	Amidohydrolase
					661,481	661,729	+	Hypothetical protein
					661,828	661,950	-	Hypothetical protein
					662,129	662,680	+	Dipeptide-binding ABC transporter, periplasmic substrate-binding component
					662,741	663,601	+	Sugar phosphate isomerases/epimerases
					664,518	666,494	-	Membrane transport protein
					666,509	666,877	-	Hypothetical protein
					666,926	668,413	-	Aldehyde dehydrogenase
					668,477	668,596	+	Hypothetical protein
					668,693	669,088	-	Hypothetical protein
					669,220	670,850	-	Hypothetical protein
					671,037	672,185	-	Amidohydrolase 2
					672,381	673,577	-	3-ketoacyl-CoA thiolase (EC 2.3.1.16)
					673,649	674,155	-	Transcriptional regulator, MarR family
					674,225	675,340	-	Oligopeptide transport ATP-binding protein OppF
					675,337	676,374	-	Oligopeptide transport system permease protein OppB
					676,377	677,198	-	Dipeptide transport system permease protein DppC
					677,267	678,217	-	Dipeptide transport system permease protein DppB
					678,287	678,502	+	Hypothetical protein
					678,595	679,383	+	Oxidoreductase, short-chain dehydrogenase/reductase family
					679,440	680,282	-	Enoyl-CoA hydratase
					680,279	681,250	-	Aminopeptidase
					681,345	682,325	-	Vanillate O-demethylase oxidoreductase
					682,443	683,219	-	3-oxoacyl-[acyl-carrier protein] reductase
					683,265	684,077	-	Enoyl-CoA hydratase
					684,197	684,610	+	Mobile element protein
					684,861	685,103	+	Mobile element protein
					685,100	685,999	-	Hypothetical protein
					686,275	686,816	-	GTP cyclohydrolase I
					687,052	687,795	-	Transcriptional regulator, GntR family
					687,963	689,540	-	Dipeptide-binding ABC transporter, periplasmic substrate-binding component
					689,839	691,056	-	BarH
					691,143	691,811	+	Short-chain dehydrogenase/reductase SDR
					691,819	692,724	-	2-hydroxy-3-oxopropionate reductase
					692,800	692,928	-	Hypothetical protein
prRWAY01	706,432 .. 711,574	5,142	66.86	SIGI-HMM	706,432	706,641	-	Hypothetical protein

					706,712	707,164	+	Hypothetical protein
					707,924	709,246	+	Citrate synthase
					709,355	709,507	-	Hypothetical protein
					709,926	710,135	-	Hypothetical protein
					710,336	711,574	-	Hypothetical protein
pRWAY01	833,420 .. 839,974	6,554	61.95	SIGI-HMM	833,420	834,766	-	Possible Rep protein
					834,829	834,942	-	Hypothetical protein
					835,122	835,277	+	Hypothetical protein
					835,457	835,951	-	Hypothetical protein
					837,083	837,469	+	Putative involvement in replication/partition
					837,802	838,134	-	Hypothetical protein
					838,382	839,974	+	Putative acetyltransferase
pRWAY02	392,452 .. 399442	6,990	66.59	IslandPath-DIMOB	392,452	393,598	-	3-ketoacyl-CoA thiolase
					393,947	394,531	+	Protein of unknown function
					394,867	395,307	-	Hypothetical protein
					395,359	395,793	+	Formate hydrogenlyase subunit 7
					395,790	395,933	+	Hypothetical protein
					395,944	397,281	+	3-ketoacyl-CoA thiolase
					397,726	398,361	+	Mobile element protein
					398,443	398,592	-	Hypothetical protein
					398,678	399,442	-	Mobile element protein
pRWAY02	441,129 .. 448,022	6,893	67.31	IslandPath-DIMOB	441,129	442,102	-	Mobile element protein
					442,506	442,640	-	Transposase
					442,867	443,523	-	Hypothetical protein
					443,523	444,236	-	Hypothetical protein
					444,373	444,543	+	Hypothetical protein
					444,647	444,913	-	Hypothetical protein
					445,488	447,515	+	Tellurium resistance protein TerD
					447,873	448,022	-	Hypothetical protein
pRWAY03	22,115 .. 28,334	6,219	65.35	SIGI-HMM	20,757	22,118	+	Beta-lactamase domain protein
					22,115	23,413	+	Major facilitator superfamily (MFS) transporter
					23,441	23,899	-	Hypothetical protein
					23,899	24,624	-	Putative uncharacterized protein
					24,862	25,128	+	Hypothetical protein
					25,195	25,509	+	Hypothetical protein
					25,901	27,250	+	Hypothetical protein
					27,256	28,044	-	Alpha/beta hydrolase fold
					28,146	28,334	-	Hypothetical protein
pRWAY03	132,522 .. 137,582	5,060	66.60	SIGI-HMM	132,522	132,728	+	Copper chaperone
					132,760	132,942	+	Hypothetical protein
					132,973	135,240	+	Lead, cadmium, zinc and mercury transporting ATPase; Copper-translocating

					135,230	135,454	-	Hypothetical protein
					135,532	135,858	+	Repressor CsoR of the copZA operon
					135,883	136,266	-	Putative transcriptional regulator, ArsR family
					136,854	137,582	+	Protein of unknown function
pRWAY03	253,769 .. 259,031	5,262	67.43	SIGI-HMM	252,879	253,772	-	Mobile element protein
					253,769	254,110	-	Mobile element protein
					254,103	255,197	+	Mobile element protein
					255,209	256,468	-	Hypothetical protein
					256,465	257,553	-	ATPase associated with various cellular activities
					257,751	257,948	-	Hypothetical protein
					258,101	258,526	-	Hypothetical protein
					258,591	258,818	+	Hypothetical protein
					258,864	259,031	+	Hypothetical protein
pRWAY03	304,048 .. 312,080	8,032	60.01	IslandPath-DIMOB	304,048	304,380	-	Hypothetical protein
					304,449	305,978	-	Hypothetical protein
					306,136	306,576	+	Putative involvement in replication/partition
					306,600	306,959	-	Hypothetical protein
					307,218	307,760	-	Unknown
					309,927	310,223	+	Hypothetical protein
					310,747	310,935	+	Hypothetical protein
					311,613	312,080	+	Hypothetical protein
